# A tailored CoA ligase–*N*-acyltransferase cascade for on-DNA amide bond formation gives access to broad substrate scope

**DOI:** 10.1038/s41929-026-01576-x

**Published:** 2026-07-17

**Authors:** Daniela Schaub, Alice Lessing, Fabian Meyer, Peter Stockinger, Miquel Estévez-Gay, Michael Eichenberger, Gerlis von Haugwitz, Andreas Gloger, Jörg Scheuermann, Rebecca Buller

**Affiliations:** 1 Competence Center for Biocatalysis, Zurich University of Applied Sciences, Wädenswil, Switzerland; 2 Center for Functional Protein Assemblies (CPA) and Department of Bioscience, TUM School of Natural Sciences, Technical University of Munich (TUM), Garching, Germany; 3 Department of Chemistry, Biochemistry and Pharmaceutical Sciences, University of Bern, Bern, Switzerland; 4 Institute of Pharmaceutical Sciences, Department of Chemistry and Applied Biosciences, ETH Zurich, Zurich, Switzerland; 5 Present Address: Chemical and Analytical Development, Novartis Pharma AG, Basel, Switzerland

**Keywords:** Biocatalysis, Enzymes, Combinatorial libraries

## Abstract

DNA-encoded chemical library (DEL) technology is a powerful tool in early-stage drug discovery. Although widely applied in industry and academia, challenges persist in generating DELs with high quality and chemical diversity. Low yields in building-block incorporation, limited selectivity and, most importantly, DNA damage from harsh reaction conditions compromise library quality, reduce signal-to-noise in affinity selections and ultimately hinder drug discovery. Here we show that tailored enzymes can be harnessed for the effective construction of molecular diversity on DNA under mild conditions. Targeting amide bond formation, we designed a cascade of complementary coenzyme A ligases and rationally tailored *N*-acyltransferases to access a broad amide scope on-DNA (>120 examples), identifying structural elements that optimize the biocatalysts’ DNA compatibility in the process. Integrating the enzymatic cascade with chemical synthesis led to the construction of a diverse DEL without damage to the DNA barcode, highlighting the biocatalysts’ applicability for early scaffold construction and late-stage functionalization.

**Figure:**
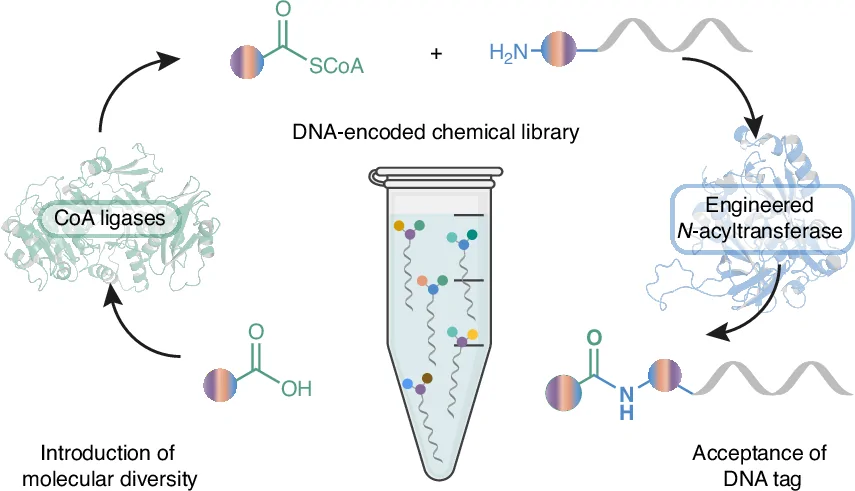

## Abstract (web summary)

DNA-encoded library (DEL) synthesis can be constrained by DNA damage and limited reaction scope under conventional chemistry. Here a complementary CoA ligase–*N*-acyltransferase cascade is engineered to enable high yield, DNA-compatible amide formation, expanding substrate scope and DEL quality.

## Main

**Fig. 1:**
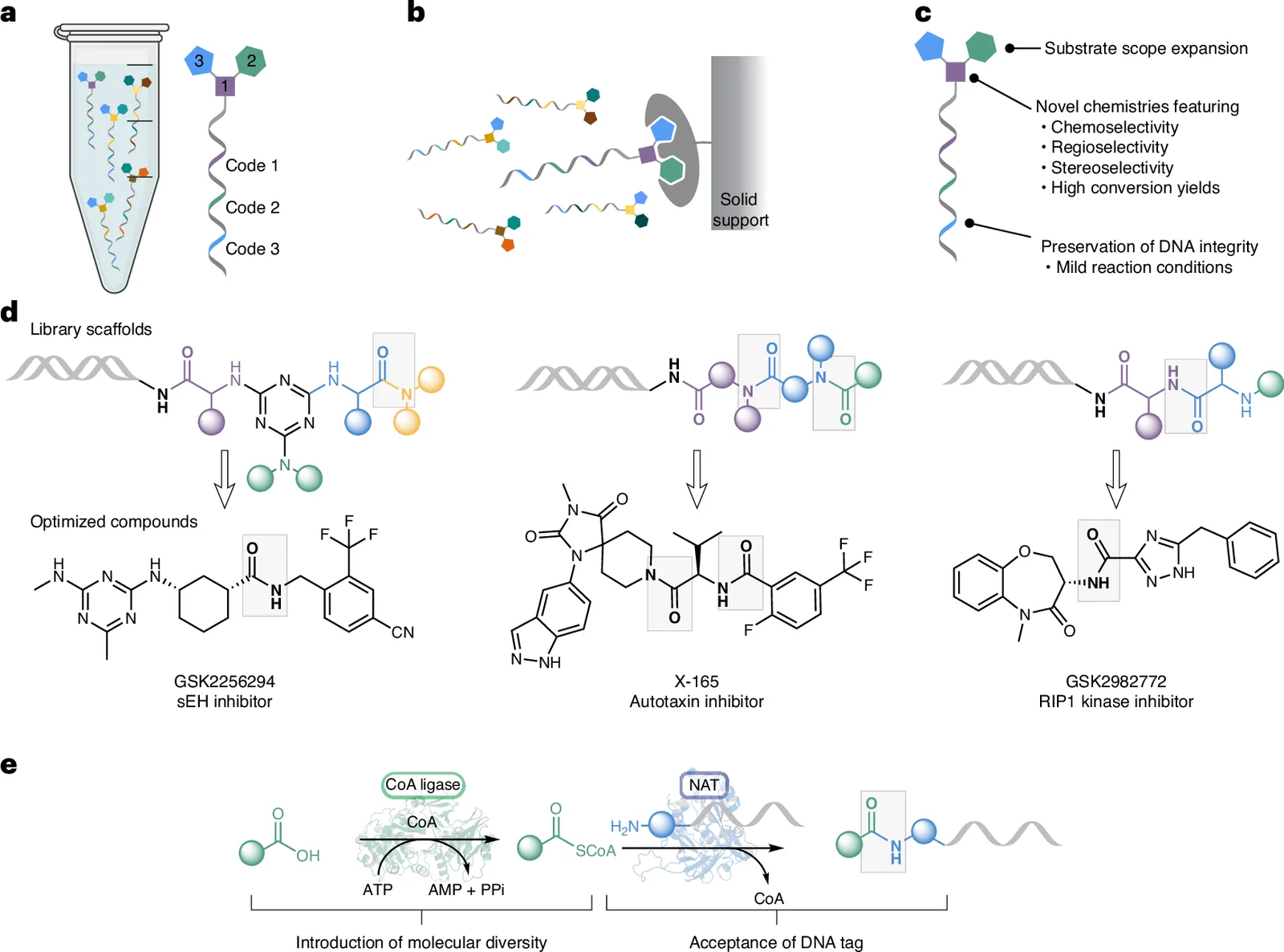
Concept of DELs and enzymatic on-DNA amide bond formation. **a**–**c**, Schematic representation of a DEL structure (**a**), affinity-based selection against pharmaceutically relevant protein targets (**b**) and advantages of biocatalysis-aided DEL synthesis (**c**). **d**, Optimized clinical candidates derived from screening amide bond containing DELs^11,22–24^. **e**, Enzymatic cascade consisting of complementary CoA ligases (CLs) and a NAT with broad substrate scope for on-DNA amide bond formation.

The discovery and development of new therapeutic modalities remains both time-consuming and costly^1,2^. While technologies for the generation of specific antibodies are well established^3–5^, the identification of high-affinity and specific small-molecule drugs continues to pose a major challenge. To address this bottleneck, barcoded compound collections, most notably DNA-encoded (chemical) libraries (DELs)^6^, have emerged as powerful and cost-efficient discovery platforms^7–10^. Today, DEL technology is widely applied and further developed across academia and industry (Fig. 1a–d), including in nearly all major pharmaceutical companies, where it is fuelling clinical pipelines^11–14^. DELs are generated through iterative split-and-pool combinatorial synthesis, in which each chemical reaction step is recorded by ligating a unique DNA tag^15–17^ (Fig. 1a). This strategy enables the creation of vast libraries, typically ranging from 10^6^ to 10^9^ library members. These libraries can be screened in parallel against protein targets of pharmaceutical interest by affinity-based selection^10,18–20^ (Fig. 1b). Enriched binders are then identified by polymerase chain reaction (PCR) amplification of the DNA tags and next-generation DNA sequencing^21^.

Since its introduction in the early 2000s, DEL technology has been continuously improved and delivered numerous clinical drug candidates^11,22–24^ (Fig. 1d). Nevertheless, fundamental limitations persist. The unavoidable presence of DNA during library synthesis imposes strict constraints on reaction conditions, as base modifications or even complete degradation of the DNA tag can occur, leading to code alterations or loss of PCR amplifiability^25,26^. As a consequence, chemical transformations must always be optimized for DNA compatibility and only a restricted subset of organic chemical conversions can, so far, be used for synthesizing productive DELs^27^. Recent innovations, such as solid-phase based synthesis to circumvent aqueous conditions or micellar reaction compartmentalization, have expanded the toolkit^28,29^, but the identification of high-yielding, diversity-generating and DNA-compatible transformations remains the central challenge in DEL technology^30^. At present, DEL construction is limited to a low number of split-and-pool cycles (typically 2–4) as otherwise the accumulation of truncated products^31,32^ rapidly degrades library quality and complicates hit identification during affinity selections by reducing signal-to-noise ratios^28^.

To address these limitations, we turned our attention to biocatalysis-aided DEL synthesis, as mild enzymatic transformations hold the promise to be DNA preserving while simultaneously providing products with excellent chemoselectivity, regioselectivity and stereoselectivity at high yields (Fig. 1c). Intrigued by early examples describing the use of enzymes for on-DNA aldol reactions (aldolases)^33^ and carbohydrate couplings (glycosyltransferases)^34^ we proposed that the development of engineered biocatalysts could broaden the hitherto moderate substrate spectrum and improve product yields, providing more effective tools for building molecular diversity in the presence of a DNA tag and leading to enhanced DEL quality and scope. In addition, we predicted that the tailor-made enzymes could easily be combined with existing chemical methods and, theoretically, be used in any of the consecutive split-and-pool cycles needed for library synthesis^35^.

As the value of a reaction for DEL synthesis is largely determined by its applicability across a wide range of substrates^36^, transformations that make use of commercially available building blocks are especially attractive^37^. Analyses of supplier catalogues show that amines and carboxylic acids are by far the most widely available building blocks^38,39^, followed by less diverse and chemically less stable compound classes such as aryl halides, aldehydes and ketones^40^. As a consequence, investigating the potential of enzymatic amide bond formation on DNA was considered particularly valuable, given that this transformation represents a fundamental reaction in pharmaceutical chemistry and underlies the synthesis of a broad spectrum of small-molecule therapeutics^41^. While chemical procedures to couple carboxylic acids and amines have been established in DEL technology, these methods often require large molar excesses of building blocks and coupling reagents, lead to varying coupling efficiency and potentially damage the DNA tag^25,42^. By contrast, enzymatic coupling of carboxylic acids and amines can generate amides with excellent selectivity from a diverse set of amines and carboxylic acids under mild reaction conditions^43–46^.

When assessing the suitability (on the basis of substrate scope as well as reaction conditions) of amide bond forming enzymes for DEL technology (for example, ATP-dependent amide bond forming enzymes^44,45,47^; lipases^48^; nitrile hydratases^49^), we were particularly intrigued by the versatility of an enzymatic cascade consisting of coenzyme A (CoA) ligases and *N*-acyltransferases (NATs)^43^. We predicted that by applying such a cascade, we could harness complementary wild-type CoA ligases to activate a broad set of carboxylic acids in an ATP-dependent manner, while only the NATs, applied in the amide forming step, would need to be specifically tailored for on-DNA chemistry (Fig. 1e).

Here we show how to implement this concept by designing an enzymatic cascade consisting of complementary CoA ligases and engineered NATs, giving access to a wide portfolio of on-DNA amides (>120 examples). The enzymatic method affords excellent yields on-DNA (on average >91% conversion in the enzymatically constructed DEL) while fully preserving the integrity of the encoding DNA tag. Applying a structure-guided engineering approach as well as a rational exchange of structural elements (loop swap), we identified key motifs important for on-DNA chemistry that could be transferred to distant enzyme scaffolds (27% sequence identity) effectively improving on-DNA activity. Combining the enzymatic cascade with chemical steps, a DEL consisting of 72 individual compounds was obtained, highlighting the versatility of our biocatalysis-aided DEL construction approach, which can be applied for initial scaffold building as well as for late-stage functionalization.

## Results

### Enzyme selection and assessment of substrate scope

To enable access to broad molecular diversity in the presence of DNA, we selected seven wild-type CoA ligases and NATs on the basis of their reported substrate scope^43,50^, origin and sequence diversity (Supplementary Table 1 and Supplementary Fig. 1). The CoA ligase panel comprised two eukaryotic, four bacterial and one archaeal enzyme, selected primarily for their complementary carboxylic acid substrate scopes^43,50^. For the NATs, intended for unprecedented on-DNA activity, the selection encompassed several different families (BAHD family, GCN5-related *N*-acetyltransferase (GNAT) family and arylamine NATs), each characterized by a distinct catalytic mechanism (BAHD family: HXXXD motif; GNAT family: catalytic tyrosine; arylamine NATs: Cys-His-Asp motif) and protein architecture (CATh IDs^51^: chloramphenicol acetyltransferase-like domain (BAHD): 3.30.559.10, GNAT: 3.40.630.30, *N*-arylamine acyltransferase: 3.30.2140.10)^52–54^.

Synthetic genes were cloned into a pET28b vector containing an N-terminal His-tag (Supplementary Table 2), transformed into *Escherichia coli* (*E. coli*) BL21(DE3) and expressed to produce the corresponding enzyme. Soluble production could be achieved for four CoA ligases, namely bacterial ipfF, LcCL and GtheCoAlg as well as the archaeal MsedCoAlg2 and for all selected NATs except for eukaryotic 11AtAT from *Arabidopsis thaliana* (Supplementary Fig. 2). Using ipfF from *Sphingomonas* sp. *Ibu-2* (UniProt A1E027), with its broad aromatic substrate scope, as a representative CoA ligase in initial activity tests, crude enzyme preparations were used to evaluate whether modifications to reported amine substrates, that is the addition of synthetic handles intended for the subsequent coupling to the DNA barcodes (carboxylic acid or methyl ester moieties), would be tolerated by the NATs (Supplementary Fig. 3). In detail, we screened our NAT panel in cascade with CoA ligase ipfF for the amide bond formation between 4-fluorophenylacetic acid (**1**) and freestanding amines **A**–**D**. Product formation was monitored using liquid chromatography coupled to mass spectrometry (LC-MS) by following the *m/z* ratios of the expected products (Supplementary Fig. 3).

For enzymatic cascades including NATs 05PaAT from *Pseudomonas aeruginosa* (UniProt Q9HUY3) or 42GmAT from *Gibberella moniliformis* (UniProt B7SP66), successful formation of products **1A**–**1D** or **1A**–**1C** was observed, respectively, providing opportunities for further substrate modification. In addition, a side product with a mass corresponding to the acetylated amine was detected in some reactions, probably resulting from a competing amide bond forming reaction with acetyl-CoA from the lysate. All lysate reactions yielding product formation were validated using assays with enzymes purified by Ni-NTA affinity chromatography (Supplementary Table 3 and Supplementary Figs. 2 and 4) and oligomeric states of key enzymes were determined by size-exclusion chromatography (Supplementary Fig. 5 and Supplementary Table 4).

**Fig. 2:**
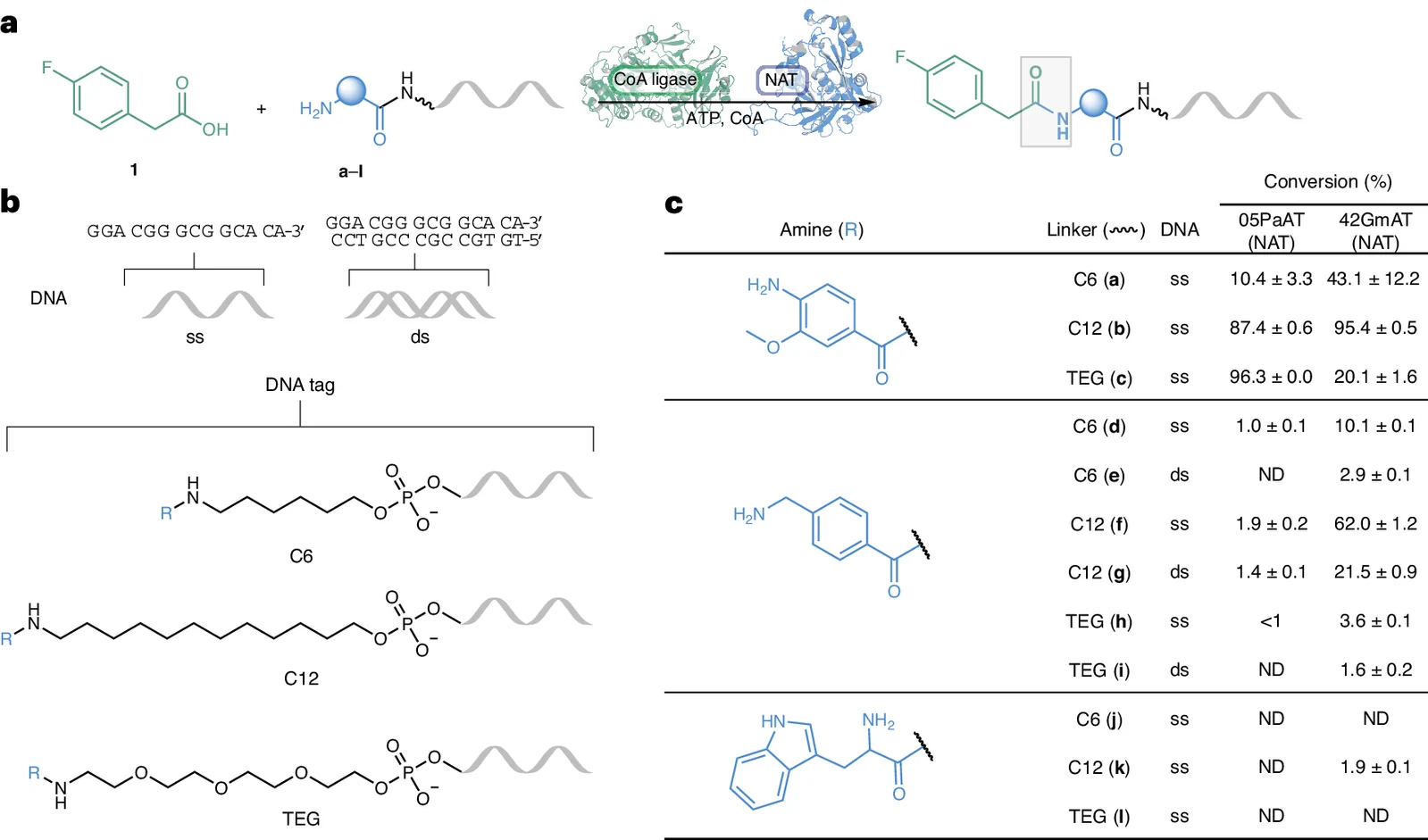
Enzymatic on-DNA amide bond formation. **a**,**b**, Enzymatic amide coupling reactions (**a**) between carboxylic acid **1** and a set of amines (**a**–**l**) carrying DNA 14-mers (**b**, single stranded, ss, or double stranded, ds) conjugated via a set of strategically chosen linkers (C6, C12, TEG) using CoA ligase ipfF and NATs 05PaAT or 42GmAT, respectively. **c**, Conversions are reported as mean values ± s.d. of three technical replicates (*n* = 3). ND, not detectable. Source data

Building on the successful results from the amine screening, on-DNA substrates were constructed (Fig. 2a). Examples from DEL^33,55^ and PROTAC^56^ literature highlight that the nature of the linker moiety can be decisive for ligand binding and recognition; in particular, length and hydrophobicity of the chosen tether can have an impact on affinity. For example, ligands conjugated to a double-stranded headpiece-DNA were shown to exhibit lower binding affinity against target proteins compared with the same ligands conjugated to a single-stranded DNA via a C6-linker^55,57^. To explore this effect and ensure to work with the most suitable linker architecture, molecular tethers of varying length and properties (C6, C12 and TEG, Fig. 2b) were installed at the 5′ end of a 14-mer oligonucleotide (GGA CGG GCG GCA CA-3′) and the resulting DNA tags were coupled to amines **A**, **C** and **D** (Supplementary Figs. 6 and 7 and Supplementary Table 5). The used oligomer was designed to prevent the formation of secondary structures such as hairpins or self-dimers and to form a stable duplex with a melting temperature of 59.4 °C. To test the influence of double-stranded DNA on enzyme performance, the single-stranded conjugates attached to molecule **C** were hybridized with the complementary strand (*T*_m_ = 59.4 °C, Supplementary Fig. 8).

Proof-of-concept biocatalytic on-DNA reactions were conducted with the established enzymatic cascade (Fig. 2a) consisting of purified CoA ligase ipfF (*c* = 20 µM) and NATs 05PaAT or 42GmAT (*c* = 20 µM), using 0.1 mM carboxylic acid **1** and 0.05 mM DNA-conjugated amines (**a**–**l**) in aqueous Tris buffer (pH 8.0) at 25 °C. For DNA-tagged substrates, product formation was monitored by ion-pairing LC-MS through detection of the expected *m/z* values. As the DNA oligonucleotide is the dominant chromophore (Supplementary Fig. 9), quantification was achieved by measuring the peak areas at a wavelength of 260 nm in accordance with established DEL protocols^10,42,58^. To closely mimic DEL synthesis conditions^17,59–61^ already from this early development stage, the reactions were carried out in a volume of 20 µl on a nanomolar scale. To our delight, successful product formation was observed for DNA-conjugated amines **a**–**I** and **k**, and up to 95% conversion was achieved for amides **1b** and **1c**, even under non-optimized reaction conditions (Fig. 2c). Further analysing the on-DNA experiments, we found that longer, hydrophobic linkers worked better in conjunction with the active NATs. Reactions containing C12 mostly showed higher conversions than those with C6-linked or TEG-linked amines (Fig. 2c), giving particularly good results with NAT 42GmAT. We did not observe any DNA degradation in the recorded chromatograms underscoring the DNA compatibility of the enzymatic reactions, while the negative control (without any enzyme) allowed us to exclude the occurrence of non-enzymatic background (Supplementary Fig. 10). Attempts to invert the coupling strategy, for example, activating DNA-coupled carboxylic acids with CoA ligase ipfF did not yield on-DNA CoA thioesters (Supplementary Figs. 11 and 12).

### Effect of DNA tag on enzymatic activity

Notably, we found that the type of DNA oligomer used (double stranded versus single stranded) had a clear impact on enzyme performance. For example, NAT 42GmAT exhibited only 44% relative activity for amines tethered to double-stranded DNA (**e**,**g**,**i**) compared with the identical substrates tagged with single-stranded oligonucleotide (**d**,**f**,**h**) (Fig. 2c). To investigate this effect, we used the cofolding models Boltz-2 and physics-corrected Boltz-2x^62,63^, both having a rich set of double-stranded DNA structures in their training data, to simulate 42GmAT with single-stranded (**f**) and double-stranded (**g**) DNA-tagged substrate. In these analyses, we found that in combination with the single-stranded DNA tag, amino acid Y229 consistently engaged in *π*–*π* stacking with the initial guanine of the oligo (vide infra), potentially guiding substrate positioning. Enzyme interactions with the double-stranded DNA tag, however, could not correctly be captured by either of the models. While the Boltz-2x model of the enzyme–substrate complex favoured a single-stranded binding mode of the DNA tag, the Boltz-2 model predicted an incorrectly paired and distorted double-stranded helix (Supplementary Fig. 13). Further analysis by NCIPLOT^64^, a computational tool to visualize and analyse non-covalent interactions, revealed pronounced repulsive interactions between the double-stranded DNA-tagged substrate and the enzyme (Supplementary Fig. 14). Although modelling artefacts caused by pairing substrate-modified with unmodified DNA strands cannot be excluded, the convergence of experimental activity data and structural modelling supports a steric origin of the reduced conversion of double-stranded DNA-tagged substrates and the improved acceptance of those carrying a single DNA-strand.

**Extended Data Fig. 1:**
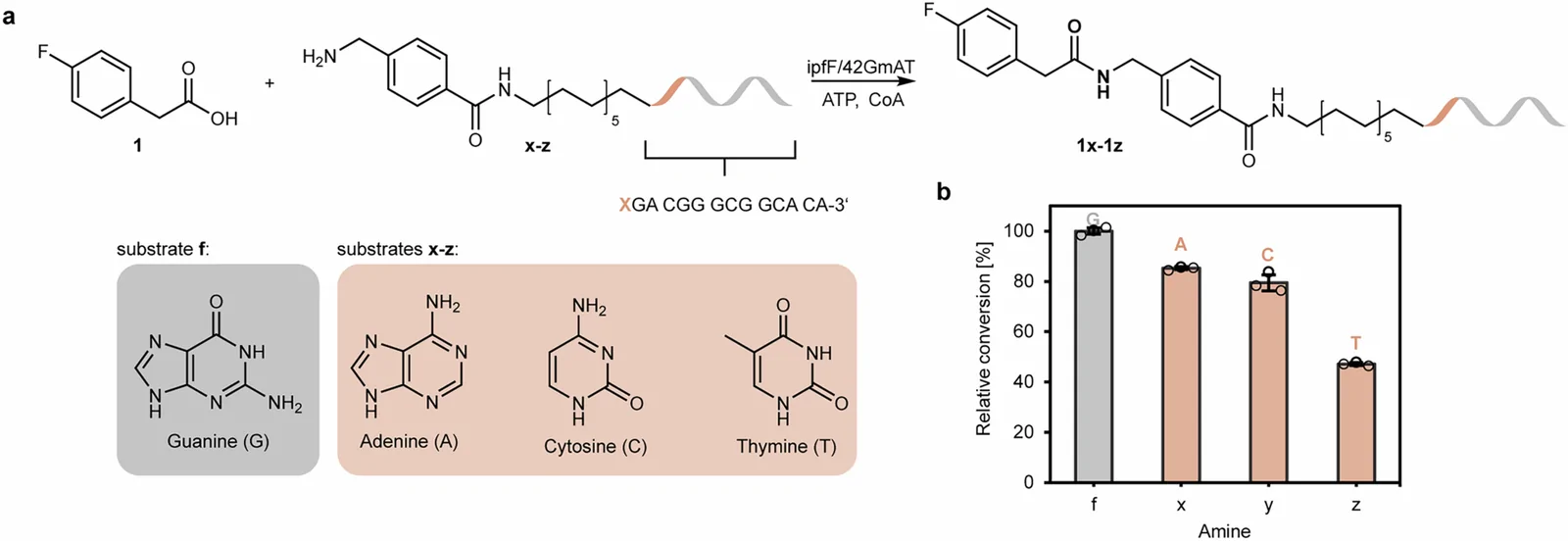
Base exchange. **a**, To test the influence of DNA sequence on enzyme performance, the initial base in substrate **f** was replaced with adenine (**x**), cytosine (**y**), and thymine (**z**). **b**, The barplot shows relative conversion of the enzymatic coupling reactions between carboxylic acid **1** and the on-DNA amines **f**, **x**–**z**. Reaction conditions: 20 µM ipfF, 20 µM 42GmAT, 0.1 mM carboxylic acid **1**, 0.05 mM DNA-conjugated amines (**f**, **x**-**z**) in aqueous Tris buffer (pH 8.0) at 25 °C. Data shown in the barplot are the mean values ± s.d. (error bars) of three technical replicates (n = 3). Source data

Guided by the computational insights, we explored whether the DNA sequence could be used to modulate enzyme activity. For this purpose, we replaced guanine—serving as the first base in the standard single-stranded 14-mer oligo—with adenine, cytosine and thymine, and coupled the resulting DNA strands via a C12 linker onto amine **C** (Supplementary Fig. 15). Cascade reactions with CoA ligase ipfF and NAT 42GmAT with this customized substrate set highlighted a preference of the enzyme for the purine bases guanine (100% relative activity), followed by adenine (85.2% relative activity) and the pyrimidine cytosine (79.5% relative activity), while thymine was less well accepted (47.2% relative activity) (Extended Data Fig. 1). The energy landscape of the system, as analysed by metadynamics simulations and xTB-GFN2-based^65^ binding energy calculations, corroborated the experimentally observed conversions, indicating that the differential enzymatic activities are largely governed by differences in binding affinity towards the first base (Supplementary Table 6). Taken together, these results pointed to exploitable interactions between enzyme and DNA tag and identified the single-stranded 14-mer oligo (GGA CGG GCG GCA CA-3′) coupled via its 5′ end to a C12 linker as the optimal set-up for all further enzyme engineering, substrate scoping and DEL construction experiments.

**Fig. 3:**
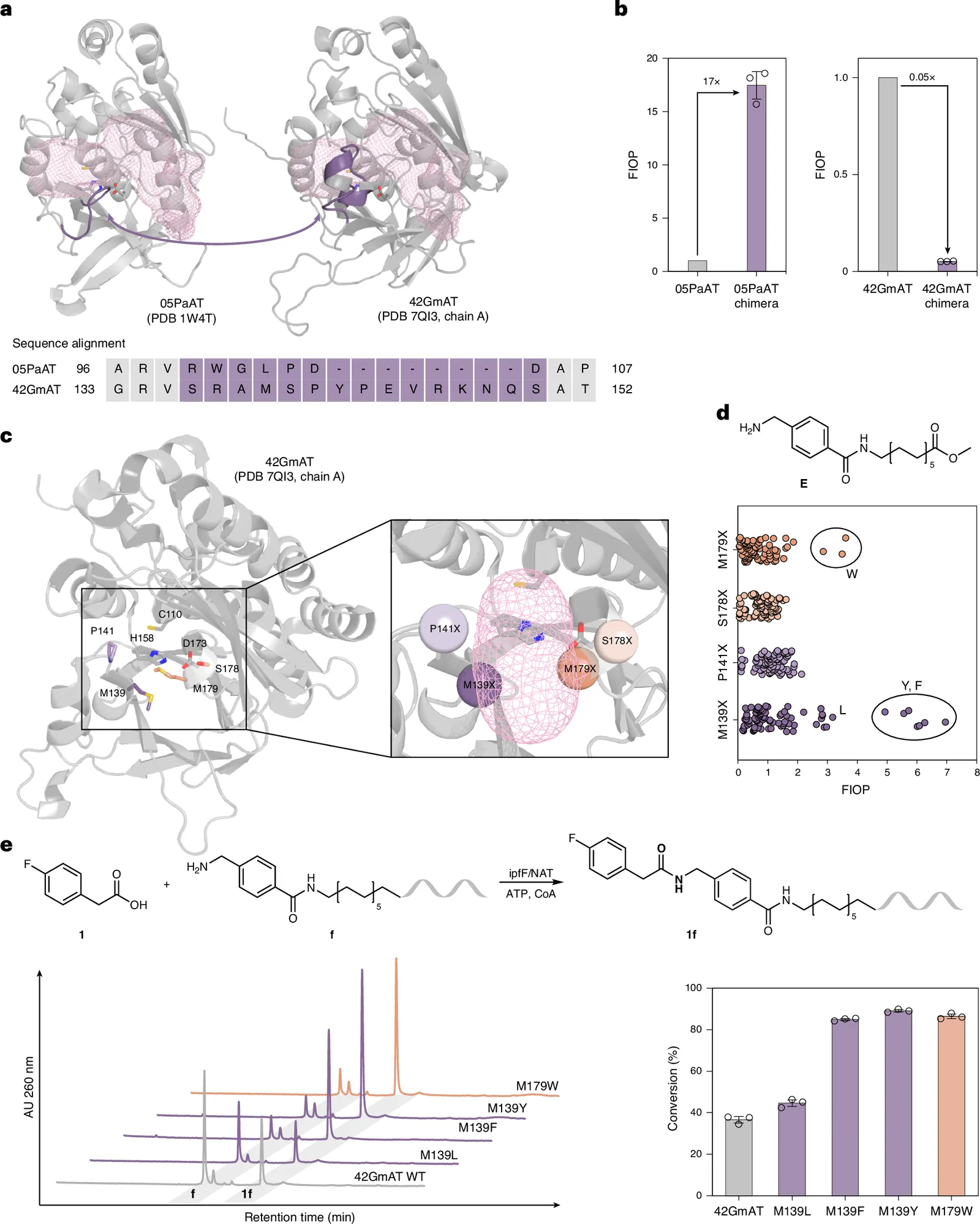
Engineering NAT 42GmAT. **a**, Representation of the loop swap between NATs 05PaAT and 42GmAT. Structure and sequence of the exchanged protein motif is highlighted in purple, while the binding pocket is indicated by a pink mesh. **b**, FIOP of 05PaAT chimera and 42GmAT chimera when screened for the amide bond forming reaction between carboxylic acid **1** and DNA-conjugated amine **f** to the DNA-conjugated product **1f**. **c**, Overall structure of NAT 42GmAT (PDB 7QI3) and a zoom-in view of the active site highlighting the substrate entrance tunnel (pink mesh), which was identified using the bioinformatic tool CAVER^69^. Residues selected for sites-saturation mutagenesis are shown as coloured spheres, while the catalytic triad is shown as grey sticks (C110, H158, D173). **d**, FIOP of 42GmAT variants screened for the amide bond forming reaction between the screening substrate **E** (no DNA tag) and carboxylic acid **1**. Since the terminal ester group of screening substrate **E** was susceptible to hydrolysis in *E. coli* lysate (empty vector control: 90% hydrolysis of substrate), only the major hydrolysed product was considered for calculations. Top performing variants are annotated. **e**, High-performance liquid chromatograms of amide bond forming reactions between carboxylic acid **1** and DNA-conjugated amine **f** to the DNA-conjugated product **1f** by best-performing 42GmAT variants and the wild-type enzyme. The accompanying bar plot shows the corresponding enzymatic conversion to product **1f** (*c*_NAT_ = 10 µM). Variant data in **b**,**e** are the mean values ± s.d. of three technical replicates (*n* = 3). Source data

Even though being consistently less active in the off-DNA amine screening (Supplementary Table 3), our experimental data (Fig. 2c) demonstrated that 42GmAT generally outperformed 05PaAT in on-DNA amide coupling reactions, triggering us to conduct structural analyses to uncover underlying principles. Both NATs belong to the family of arylamine NATs and are characterized by relatively large and accessible binding pockets. Sequence-independent protein structure comparison (TM-align^66^) of the experimental structures with the Protein Data Bank (PDB) IDs 7QI3 (42GmAT) and 1W4T (05PaAT) showed high structural agreement between the active NATs (TM score 0.73) despite low sequence similarity (27%, Supplementary Fig. 1). Using pyKVFinder^67^, a Python-based tool for the detection and characterization of protein cavities, we found, however, that 42GmAT featured a larger pocket volume (1,517 ± 56 Å^3^) than 05PaAT (1,068 Å^3^) and, concomitantly was characterized by a larger surface area (42GmAT: 1,036 ± 6 Å^2^ versus 05PaAT: 812 Å^2^) suggesting binding site accessibility as a potential determinant for 42GmAT’s improved on-DNA performance (Fig. 3a).

**Extended Data Fig. 2:**
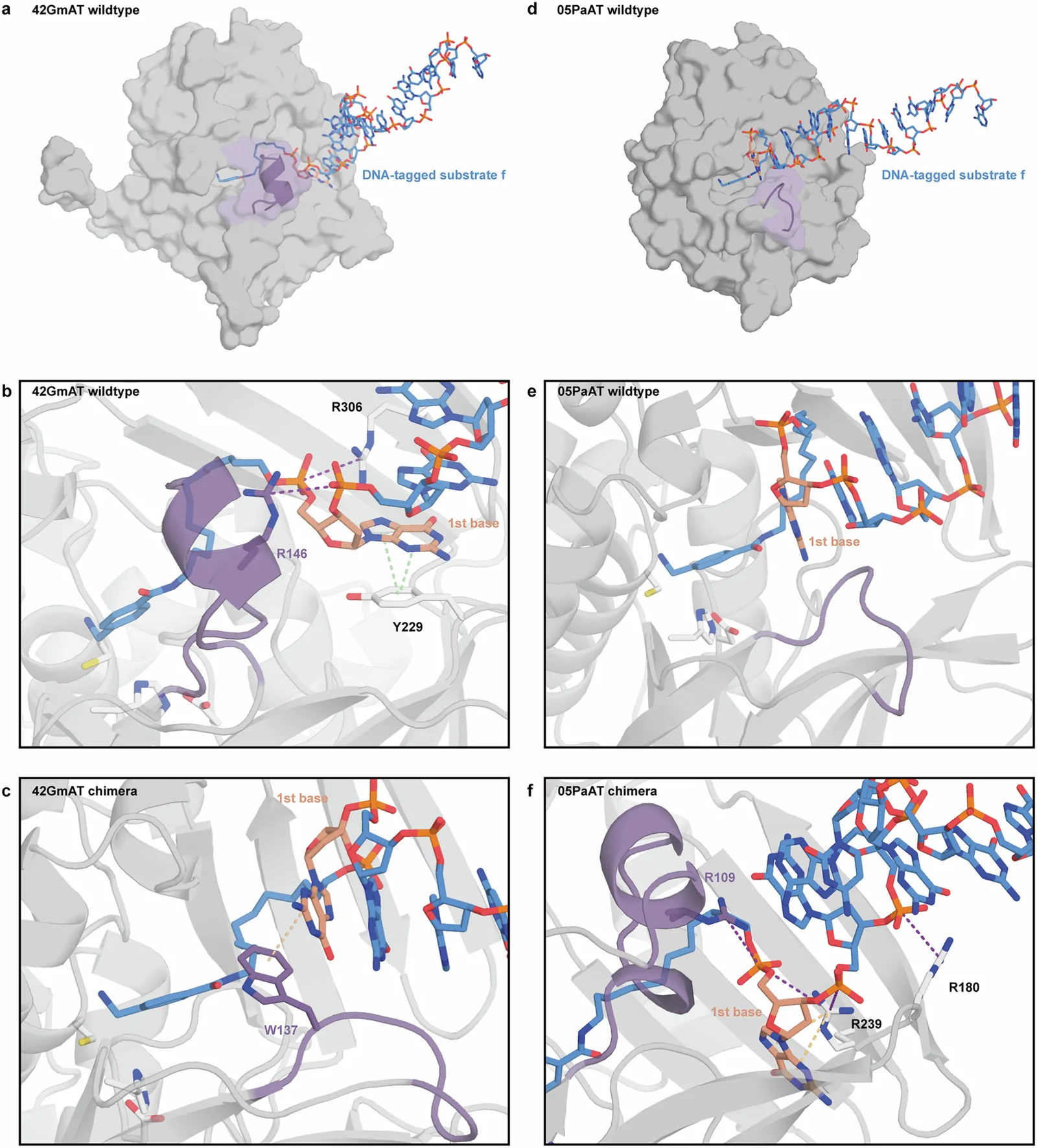
Substrate-bound structures of wild-type and chimera enzymes as predicted by Boltz-2. **a**, Predicted structure of 42GmAT with DNA-conjugated amine **f** (in blue). The helix-loop-motif (residues 136–150) is highlighted in purple. **b**, Snapshot of the active site showcasing several stabilizing interactions between the protein and the first base of the 14-mer oligonucleotide: Residues R146 and R306 form salt bridges with the DNA backbone (purple dash), whereas amino acid Y229 engages in π-π stacking (palegreen) with the first guanine base. **c**, Snapshot of the active site of the 42GmAT chimera, in which the π-π stacking and salt bridge cannot be formed to the engineered loop, while a new π-cation interaction between W137 and the first base was detected. **d**, Predicted structure of 05PaAT with DNA-conjugated amine f (in blue). The loop motif (residues 99–105) is highlighted in purple. **e**, Snapshot of the active site showing interactions detected between the protein and the DNA-tag. Notably, the native loop (purple) does not engage in interaction with the DNA-tag. **f**, Snapshot of the active site of the 05PaAT chimera, indicating the formation of a salt bridge between residue R109, located on the engineered loop, and the DNA backbone, in addition to several additional salt bridge and π-cation interactions (purple and wheat colored dashes) with residues R239 and R180.

Analysing the structures further, we also found that 42GmAT’s binding pocket architecture is partially defined by a helix-loop-helix motif (residues 136–150) spanning the active site (Fig. 3a). This structural element is absent in the less DNA-accepting NATs 05PaAT. Here the active site is covered by a flexible loop (residues 99–105). Helix-turn-helix motifs are structural elements often found in DNA-binding domains, such as in transcription factors^68^. Thus, to explore this element’s role in on-DNA catalysis, we performed a loop swap between 42GmAT and 05PaAT (Supplementary Tables 7 and 8). Both desired chimeric enzymes could be obtained in soluble form (Supplementary Fig. 16) and were tested in on-DNA amide bond forming reactions. The 05PaAT-based chimera incorporating the helix-loop-helix motif from 42GmAT exhibited a 17-fold improvement over wild-type for the amide bond formation between carboxylic acid **1** and on-DNA amine **f**, whereas the activity of the 42GmAT-based chimera harbouring the 05PaAT loop dropped by 20-fold (Fig. 3b). Applying Boltz-2x^62,63^ we modelled the two wild-type NATs and their corresponding chimeras in the presence of the DNA-conjugated amine **f** (Extended Data Fig. 2). In the 42GmAT wild-type model (Extended Data Fig. 2a), several stabilizing interactions between the protein and the first base of the 14-mer oligonucleotide were detected: Residue R146, which is part of the helix-loop-helix element, as well as residue R306 formed salt bridges with the DNA backbone, whereas amino acid Y229 engaged in *π*–*π* stacking with the initial guanine (Extended Data Fig. 2b). Such stabilizing interactions were reduced in the 42GmAT chimera, where salt bridges with R146 and R306 were no longer observed (Extended Data Fig. 2c). Analogously, the binary model of wild-type 05PaAT (Extended Data Fig. 2d) did not show any interactions between its native loop and the DNA-bound substrate (Extended Data Fig. 2e), while in the structure of the more active chimeric enzyme a salt bridge between the helix residue R109 (analogous to R146 in 42GmAT) as well as interactions between R180 and R239 and the DNA backbone were detected (Extended Data Fig. 2f), potentially facilitating on-DNA catalysis.

### 42GmAT engineering to improve on-DNA performance

Considering the superior activity of NAT 42GmAT for DNA-tagged amines (Fig. 2b), we opted to tailor this enzyme for improved activity for on-DNA amide bond formation in context of the entire enzymatic cascade using CoA ligase ipfF with substrate **f** for the carboxylic acid activation step. Guided by our structural analysis^67,69^ and the experimental results highlighting the importance of linker-type and initial DNA base, we chose four positions (M139, P141, S178, M179) at the tunnel entrance potentially in contact with the linker–DNA as targets for full site saturation (Fig. 3c). The libraries were constructed using NNK codon containing primers (Supplementary Table 7) and full genes were assembled by overlap extension PCRs^70^ (Supplementary Table 9). To ensure sufficient oversampling of the site-saturation libraries^71^, each randomized position was screened on a 96-well microtiter plate, including the wild-type enzyme and appropriate negative controls. Due to the DNA-tagged substrates’ limited stability in *E. coli* lysates (Supplementary Fig. 17), the screening work was carried out with substrate **E**, dubbed ‘screening substrate’, in which the target amine was linked to the C12 linker but not the DNA tag (Fig. 3d). Following the biocatalytic reactions, fold improvement over parent (FIOP) was quantified for each variant by comparing relative amide product formation in individual wells to that of the wild-type enzyme. This analysis identified several improved variants from positions M139, M179 and P141, with FIOPs exceeding 3 (M179W) and 5 (M139F, M139Y), whereas more modest improvements (FIOPs of roughly 2) were observed for variants M139L and P141I. Notably, none of the variants from the library S178NNK exhibited improved (FIOP < 2) amide bond forming activity (Fig. 3d).

In a next step, the plasmids harbouring the five hit variants (M139L, M139F, M139Y, P141I, M179W) were sequenced, retransformed into *E. coli* BL21(DE3) and the corresponding enzymes were produced and purified (Supplementary Fig. 16) When testing these enzyme preparations with on-DNA amine **f** (reduced enzyme load of 10 µM), improved activity of variants M139Y (FIOP 2.4), M139F (FIOP 2.3) and M179W (FIOP 2.4) was confirmed (Fig. 3e). A further combination of successful mutations at position 139 (L, F, Y) with M179W, however, did not result in any improvement beyond that observed for the single mutants (Supplementary Table 10).

To assess the catalytic improvements gained during evolution, we determined the steady-state kinetic parameters for the amide bond formation between the CoA thioester of carboxylic acid **1** (**CoA-1**, Supplementary Fig. 18) and free 4-(aminomethyl)benzoic acid (**C**) or on-DNA amine **f**. In off-DNA reactions (Supplementary Fig. 19), substrate saturation could not be achieved for either enzyme variant and thus catalytic efficiency was used to compare enzyme performance in the different settings (Supplementary Fig. 20). In off-DNA reactions, engineered 42GmAT M139Y only slightly outperformed the wild-type enzyme (*k*_cat_/*K*_m_ (42GmAT) = 2.75 ± 0.21 × 10^−3^ min^−1^ mM^−1^ (mean ± s.d.) versus *k*_cat_/*K*_m_ (42GmAT M139Y) = 4.02 ± 0.23 × 10^−3^ min^−1^ mM^−1^, where *k*_cat_ is the catalytic turnover number, *K*_m_ the Michaelis constant and *k*_cat_/*K*_m_ denotes catalytic efficiency). On-DNA reactions proceeded far more efficiently: The wild-type enzyme formed on-DNA amide **1f** with a *k*_cat_ of 5.15 ± 0.1 × 10^−2 ^min^−1^, a *K*_m_ of 0.71 ± 0.06 mM and a *k*_cat_/*K*_m_ of 7.93 ± 2.70 × 10^−2 ^min^−1^ mM^−1^ whereas the engineered 42GmAT M139Y was substantially more performant and characterized by a *k*_cat_ of 5.99 ± 0.04 × 10^−1^ min^−1^ and a *K*_m_ of 1.17 ± 0.15 mM, overall leading to a >150-fold improved catalytic efficiency for the on-DNA substrate (*k*_cat_/*K*_m_ 4.32 ± 0.01 × 10^−1 ^min^−1^ mM^−1^) compared with the parental enzyme for off-DNA reactions (Table 1).

**Table 1:** Biochemical characterization of 42GmAT wild type (WT) and 42GmAT M139Y (M139Y) variants for the biocatalytic production of **1C** (off-DNA) and **1f** (on-DNA)

Entry	Enzyme	Substrate	Apparent *k*_cat_ (min^−1^)	Apparent *K*_m_ (mM)	Apparent *k*_cat_/*K*_m_ (min^−1^ mM^−1^)	(*k*_cat_/*K*_m_)/ (*k*_cat_/*K*_m_)^WT, off‒DNA^
1	WT	**C** (off-DNA)	ND	ND	2.75 × 10^−3^ ± 0.21 × 10^−3^	1
2^a^	WT	**f** (on-DNA)	5.15 ± 0.1 × 10^−2^	0.71 ± 0.06	7.93 × 10^−2^ ± 2.70 × 10^−2^	29
3	M139Y	**C** (off-DNA)	ND	ND	4.02 × 10^−3^ ± 0.23 × 10^−3^	1.5
4	M139Y	**f** (on-DNA)	5.99 ± 0.5 × 10^−1^	1.17 ± 0.15	4.32 × 10^−1^ ± 0.01 × 10^−1^	156

Values are mean ± s.d. of three independent measurements (*n* = 3)

^a^Values are derived from two independent measurements (*n* = 2).

Further insight into the role of mutation M139Y was obtained through comparative molecular modelling of wild-type 42GmAT and the 42GmAT M139Y variant in complex with the screening substrate **E**. The small-molecule substrate was selected because, like the on-DNA substrates, it showed improved conversion with the enzyme variant while allowing more reliable modelling. Boltz-2–based cofolding simulations revealed that for wild-type 42GmAT, 29 out of 30 poses displayed a non-productive substrate orientation, in which the amine group pointed away from the catalytic pocket. In contrast, the 42GmAT M139Y variant more frequently adopted a catalytically competent substrate orientation, with 5 of 30 poses representing putative productive conformations (Supplementary Fig. 21). As expected from the activity data, size-exclusion chromatography of variant 42GmAT M139Y variant highlighted that the oligomeric state of the NATs had not been affected by the mutation (Supplementary Fig. 5 and Supplementary Table 4).

### Carboxylic acid scope of the enzymatic cascade

**Fig. 4:**
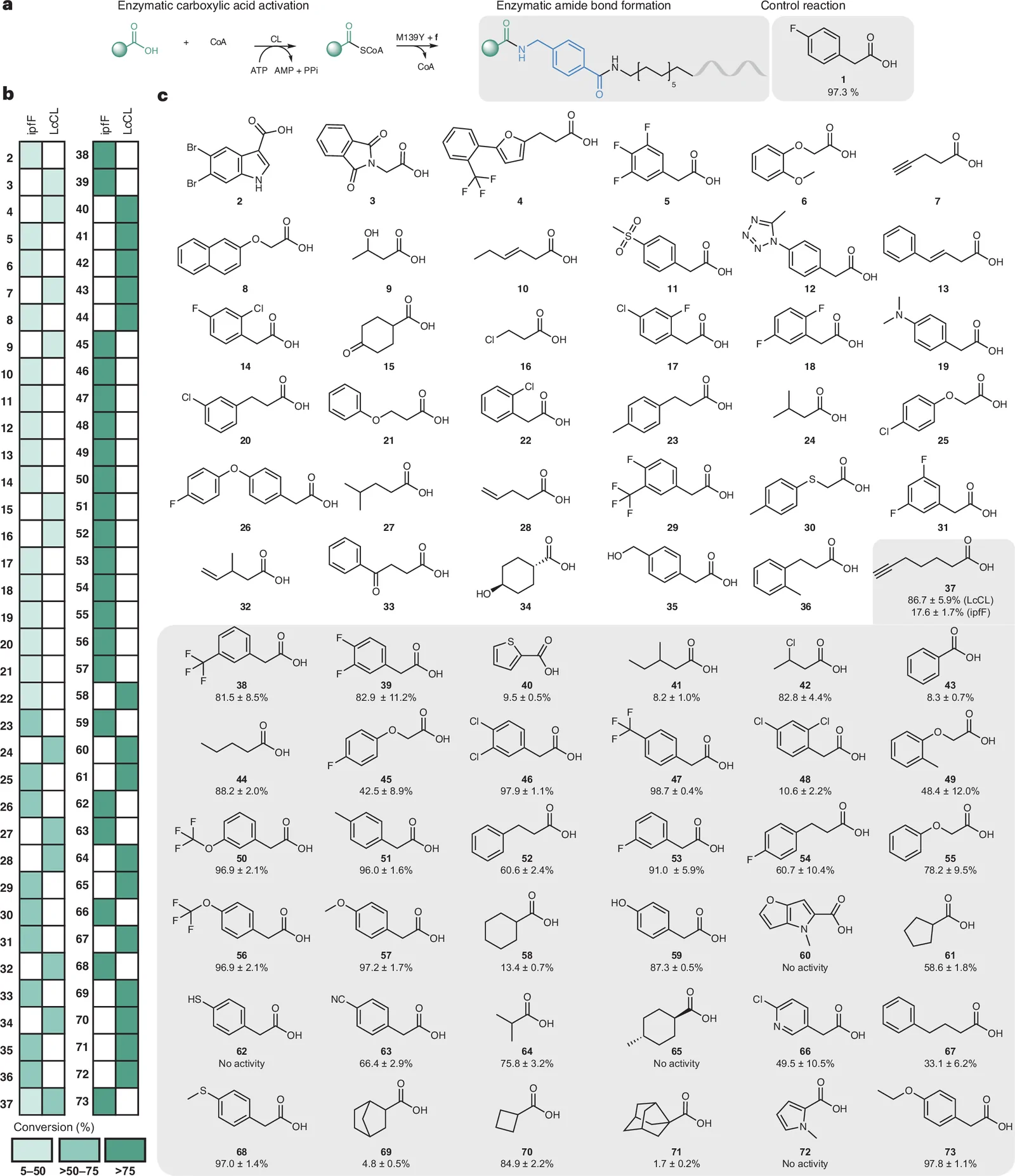
Carboxylic acid substrate scope. **a**, Reaction steps of enzymatic amide bond formation including carboxylic acid activation by CoA ligases, followed by coupling of the activated carboxylic acid to DNAconjugated amine f by N-acyltransferase variant 42GmAT M139Y. **b**, Heat map showing conversions of carboxylic acid activation by CoA ligases ipfF and LcCL as quantified by Malachite Green assay (*t* = 90 min, 2 μM CoA ligase, 0.4 U ml^−1^ pyrophosphatase, 0.1 mM carboxylic acid, 1 mM ATP, 1 mM CoA and 5 mM MgCl2 in 50 mM Tris (pH 8)). **c**, Carboxylic acid substrate scope of CoA ligases ipfF and LcCL. For carboxylic acids **37**–**73** (highlighted in grey) the full enzymatic cascade was tested for on-DNA amide bond formation (reaction scheme as depicted in **a**; carboxylic acid = 0.25 mM). Conversions to on-DNA amides are given below the structures. Data in **c** are the mean values ± s.d. of three technical replicates. Source data

**Extended Data Fig. 3:**
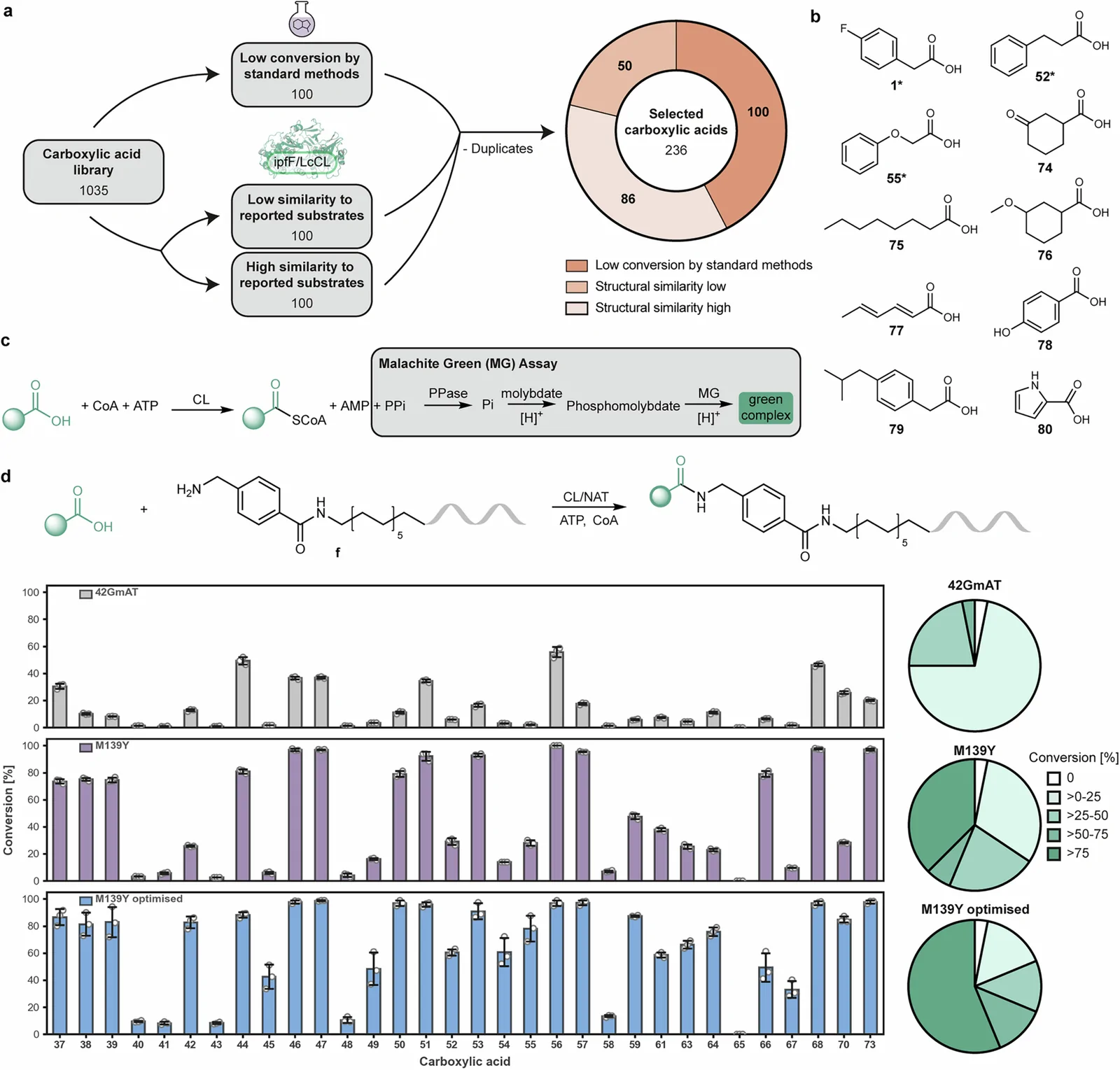
Carboxylic acid scope. **a**, A library of 236 carboxylic acids was assembled from an in-house collection ( > 1,000 compounds) to assess CoA ligase substrate scope. Compounds were selected based on Morgan fingerprint similarity to reported ipfF substrates using Tanimoto similarity, yielding 100 high- and 100 low-similarity substrates. Additionally, 100 carboxylic acids previously identified as challenging for on-DNA amidation ( < 50% conversion) were included. After removal of duplicates, 236 unique compounds remained. **b**, Reported substrates of CoA ligase ipfF which were considered for similarity calculations. **c**, Representation of the carboxylic acid activation reaction catalysed by CoA ligases and the Malachite Green assay reaction. Pyrophosphatase (PPase) was used to transform pyrophosphate, which is liberated during the enzymatic activation reaction, to detectable ortho-phosphate. **d**, Comparison of conversion rates for 42GmAT and the variant M139Y for amide bond formation with DNA-conjugated amine **f** and different carboxylic acids using in cascade with CoA ligase ipfF or LcCL (non-optimised conditions, c_carboxylic acid_ = 0.1 mM, optimised conditions, c_carboxylic acid_ = 0.25 mM). Barplots show the conversion to amide product with 42GmAT (grey) and 42GmAT M139Y (purple and blue). Accompanying pie charts shows the distribution of conversion achieved by the two *N*-acyltransferase. Data shown bar plots are the mean values ± s.d (error bars) of three technical replicates (n = 3). Source data

To supplement the improved on-DNA activity of tailored NATs 42GmAT M139Y (Supplementary Table 11), we opted to explore the substrate scope of CoA ligases ipfF from *Sphingomonas* sp. *Ibu-2* and complementary LcCL from *Leptothrix cholodnii* (UniProt B1Y4L8) with the goal of introducing additional molecular diversity on DNA. Going beyond known examples, we compiled a customized carboxylic acid collection containing a balanced mixture of acids with high (*n* = 86) or low (*n* = 50) structural similarity to the reported ipfF substrate scope^43^ (Extended Data Fig. 3a,b) as measured by the Tanimoto index^72^. The collection also comprised carboxylic acids (*n* = 100) that were previously reported as demanding building blocks for on-DNA amide bond formation, each giving less than 50% yield in chemical coupling reactions using DMT-MM or EDC/HOAt/DIPEA^73^. This led to a library consisting of 236 acids (Extended Data Fig. 3a), which was screened using purified ipfF, known for its acceptance of aromatic carboxylic acid, and the complementary enzyme LcCL, which converts aliphatic substrates. Enzyme activity was quantified using a spectrophotometric pyrophosphate detection assay (Malachite Green assay) based on measuring free phosphate concentration (Fig. 4a, Extended Data Fig. 3c, Supplementary Fig. 22).

The colorimetric screening highlighted the broad substrate scope and complementarity of the two chosen CoA ligases (Fig. 4b and Supplementary Table 12). While ipfF accepted 46 carboxylic acids, LcCL was able to activate 27 compounds, including only one common structure (**37**). Notably, we identified 22 compounds from the subset of acids with poor chemical reactivity that were activated when incubated with our selected CoA ligases, including 6-chloro-3-pyridineacetic acid (**66**), 3,4-dichlorophenylacetic acid (**46**) and 3-(trifluoromethoxy)phenylacetic acid (**50**)^73^. Attempts to directly activate Fmoc-protected amino acids, however, were unsuccessful (Supplementary Fig. 23). On the basis of the Malachite Green assay results, 32 acids were selected for coupling with on-DNA amine **f** using the full CoA ligase–NAT cascade, including either wild-type 42GmAT or 42GmAT M139Y. The NATs and its variant exhibited good compatibility with the structurally diverse carboxylic acids, yielding products in all cases except for compound **65** (Supplementary Table 13, *c*_Carboxylic acid_ = 0.1 mM). Notably, the engineered enzyme outperformed the wild-type 42GmAT across nearly all DNA-conjugated substrates and carboxylic acids tested (Extended Data Fig. 3d and Supplementary Tables 11 and 13) highlighting mutations M139Y role as a general enabler for on-DNA activity increase without negatively affecting substrate promiscuity. To avoid conditions that favour individual enzymes, reaction conditions were optimized for the full enzymatic cascade, aiming to ensure broad applicability across a wide substrate range as required for split-and-pool synthesis. The investigated reaction parameters included concentration *c*_CoA_ = 0.025–1 mM, *c*_ATP_ = 0.1–1 mM, *c*_Carboxylic acid_ = 0.1–0.5 mM, *c*_NAT_ = 20–60 µM, *T* = 20–35 °C, pH 6–8.5, reaction time 1–20 hours and *c*_DMSO_ = 0–15% (Supplementary Table 14). In particular, we found that increasing the carboxylic acid concentration to 0.25 mM had a positive effect on amide bond formation in several instances (Supplementary Table 14 and Extended Data Fig. 3d). The optimized fivefold stoichiometric excess of carboxylic acid is well below what is typically required for chemical amide bond formation on DNA (>100-fold excess)^17,59,73^, mitigating potential solubility constraints in the presence of enzyme and DNA. The optimized conditions were subsequently used to couple 37 different carboxylic acids (>75% conversion Malachite Green assay) to on-DNA amine **f** using enzymatic cascades consisting of ipfF or LcCL with 42GmAT M139Y giving access to a wide portfolio of on-DNA amides with excellent conversions (up to 98%) and purity (Fig. 4c and Supplementary Fig. 24).

### Enzymatic construction of a DEL

**Fig. 5:**
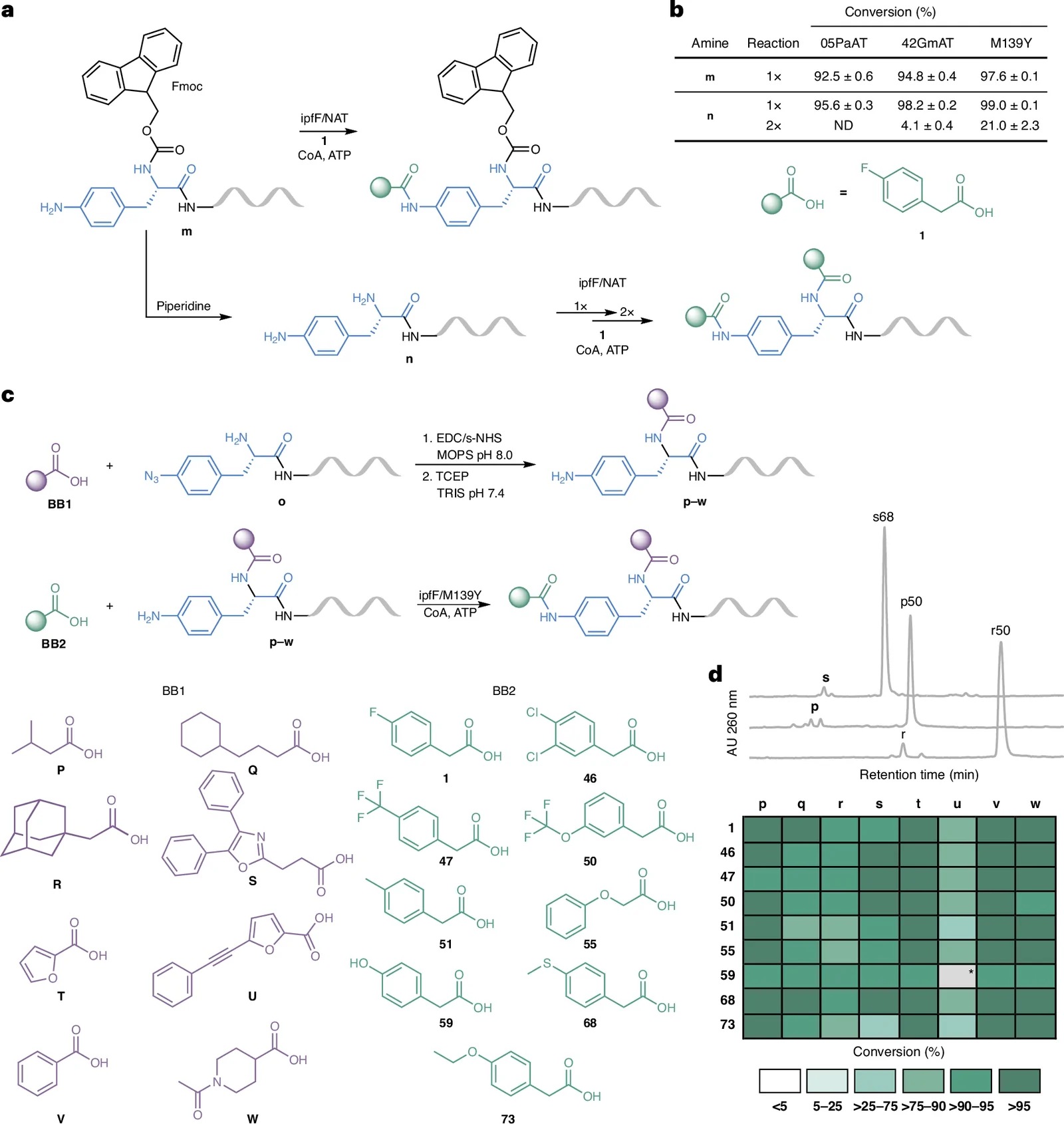
Enzymatic construction of a DEL. **a**, Enzymatic modification of trifunctional scaffolds **m** (with Fmoc protecting group) and **n** (without the Fmoc protecting group) with carboxylic acid **1** using enzymatic cascades consisting of CoA ligase ipfF and NATs 05PaAT, 42GmAT or 42GmAT M139Y, respectively. **b**, The conversion yields obtained for each enzymatic cascade. **c**, Construction scheme of the chemo-enzymatic DEL. Following chemical coupling of building blocks 1 (BB1, **P**–**W**) to amine **o**, nine carboxylic acids (shown in green) were used for the late-stage functionalization of the obtained DNA conjugates (**p**–**w**) by applying the best-performing enzymatic cascade (CoA ligase ipfF/NAT 42GmAT M139Y, carboxylic acid = 0.25 mM). **d**, Chromatograms of selected biocatalytic reactions highlight the purity of the obtained DNA-coupled products while observed conversions are indicated in the accompanying grid chart (on average 91%, *n* = 1). The asterisk denotes coeluting substrate and product peaks preventing accurate calculation of conversion values. Data in **b** are the mean values ± s.d. of three technical replicates (*n* = 3). All amines were conjugated to DNA via a C12 linker, which has been omitted in the structures for clarity. BB2: building block 2. Source data

Expanding complexity, we next assayed a trifunctional scaffold installed on the C12-linked oligomer either bearing two primary amines (**n**) or including a Fmoc protecting group (**m**) with an enzymatic cascade containing NATs 05PaAT, 42GmAT or 42GmAT M139Y (Fig. 5a). While NATs 05PaAT showed high specificity to form the mono-amidated product when assayed with amine **n**, 42GmAT M139Y could also modify the more hindered amine group in the vicinity of the DNA tag (Fig. 5a). Each enzymatic cascade afforded excellent conversion for both substrates (92–99%), including substrate **m** bearing the bulky Fmoc protecting group (Fig. 5b), opening the door for the enzymatic construction of complex DELs containing multiple building blocks.

On the basis of this promising outcome, we set out to chemo-enzymatically construct a DEL with a substrate set curated to consider both structural diversity and high conversion rates (Fig. 5c). In detail, we chemically installed eight structurally diverse carboxylic acids (building block 1) on the alpha-amine of substrate **o** (Supplementary Fig. 25 and 26, and Supplementary Table 15), before applying the best-performing enzymatic cascade (CoA ligase ipfF/NAT 42GmAT M139Y) for late-stage functionalization of the obtained DNA conjugates with a set of nine carboxylic acids (building block 2) (Fig. 5c). When evaluating library construction performance, we observed uniform incorporation and excellent conversions of all building blocks 2 (on average >91% yield), despite the structural variability and bulkiness of pre-installed fragments **p**–**w** (Fig. 5d and Supplementary Table 16).

### DNA integrity

**Extended Data Fig. 4:**
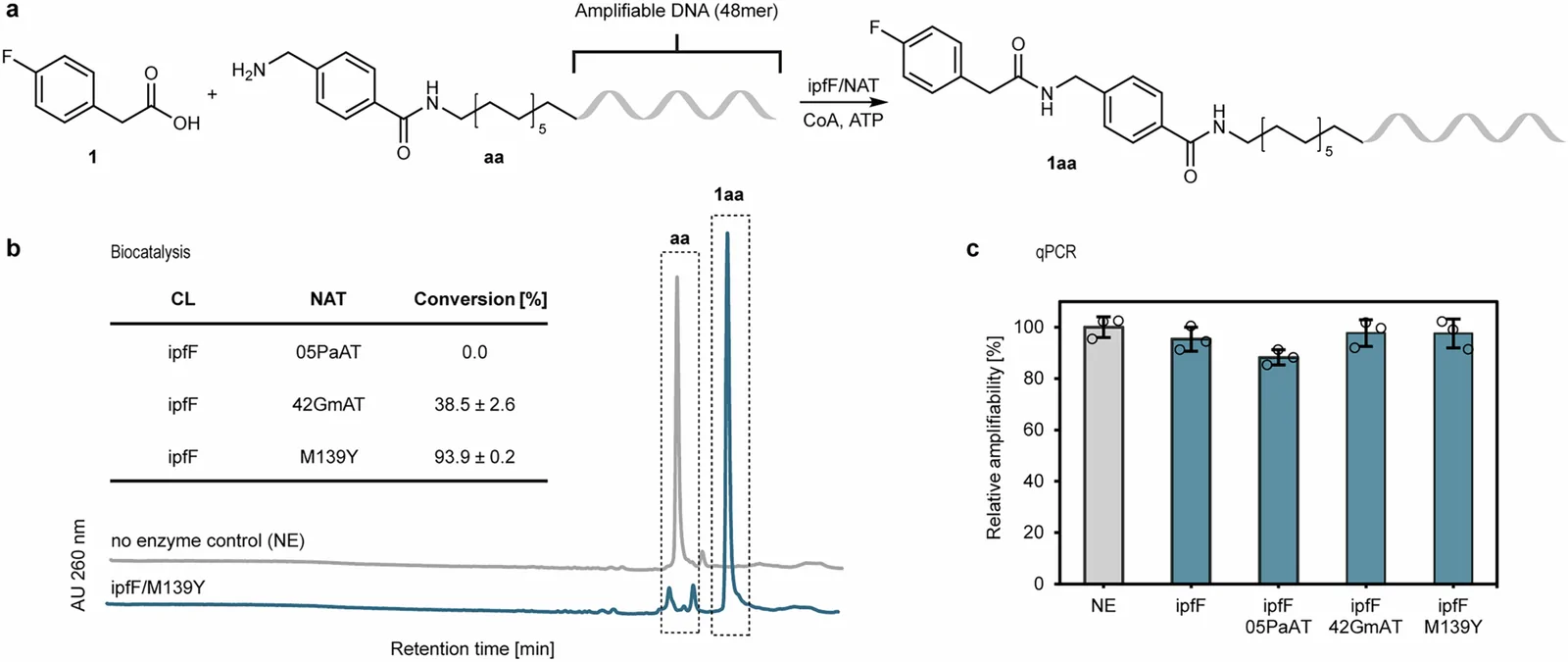
DNA integrity. **a**, Enzymatic amide bond formation between amine (**aa**), carrying an amplifiable 48mer oligonucleotide sequence, and carboxylic acid **1**. **b**, Conversions achieved by the enzymatic cascades (ipfF and 05PaAT, 42GmAT and 42GmAT M139Y) for the reaction depicted in **a**. In addition, the chromatogram of the reaction mixture comprising *N*-acyltransferase 42GmAT M139Y (blue) is compared to the chromatogram of the no enzyme control (grey). Substrate (**aa**) and product (**1aa**) peaks are indicated with boxes. **c**, Barplot depicting relative amplifiability of the DNA code post-reaction compared to a no enzyme control (NE). Data shown in **b** and **c** are the mean values ± s.d. (in **c** shown as error bars) of three technical replicates (n = 3). Source data

Beyond the uniformity and yield of on-DNA conversions, DNA integrity is an additional key performance indicator when assessing methods applied in DEL construction^25,28,74^. Complementing our LC-MS analysis, which showed no evidence of DNA degradation or off target acylation (Supplementary Fig. 27), DNA compatibility of the enzymatic cascade was further evaluated by quantitative PCR (qPCR)^25,28,74^. Using an amine linked to an amplifiable 48-mer DNA tag (**aa**), representative in length for further DEL synthesis^75^, and carboxylic acid **1** as substrates, we achieved almost quantitative conversion to amide product **1aa** (93.9%) when applying the tailored enzymatic cascade (ipfF/42GmAT M139Y) (Extended Data Fig. 4a,b) underlining the independence of enzyme performance from DNA length and composition beyond the first base (used 48-oligomer and 14-oligomer only have the first three bases in common). Following the biocatalytic reactions, DNA content was quantified by qPCR, indicating no decrease in amplifiability for any of the tested enzyme cascades compared to the no enzyme control (Extended Data Fig. 4c, Supplementary Table 17 and Supplementary Figs. 28 and 29). In addition, to complete the use tests for enzymatic DEL construction, we investigated T4 DNA ligase-mediated ligation efficiency after conducting enzymatic reactions, which proceeded to completion (Supplementary Fig. 30).

## Discussion

A critical factor in generating a successful DEL is that the chosen transformations must maintain the integrity of the DNA tag while leading to high conversions for a broad set of substrates. Unfortunately, reaction conditions routinely used in synthetic chemistry, such as low pH or the application of metal catalysts, can lead to depurinations^76,77^ and base transversions^26^ or promote DNA cleavage^25,78^. Against this backdrop, enzymes are emerging as attractive additions to the DEL-reaction toolkit^33,34,79–82^. Although, on first glance, the bulky DNA tag might be perceived as an impediment for enzymatic substrate acceptance, our increasing ability to discover, design and tailor proteins on demand using different approaches^83,84^ is helping to address this challenge, leading to highly promiscuous catalysts that can be applied in early but also late library generation. In fact, we postulate that the DNA barcode may serve as an additional substrate recognition element and, in appropriately engineered enzymes, can facilitate catalysis. This insight is not only important for DEL synthesis but might also play a role in DEL screening as the linker–DNA construct potentially influences binding affinity^55,57^. Finally, our study on tailored enzyme cascades for DEL construction shows that harnessing customized enzymes for DEL synthesis allows to achieve excellent reaction yields for a broad substrate scope using a substantially lower excess of educts paving the way for using biocatalysis also for other reaction types. Ultimately, the resulting diverse and high-quality DELs may accelerate our ability to discover new medicines.

## Methods

### Materials and reagents

Commercial enzymes, chemicals, solvents, kits and materials are listed in Supplementary Data 2. Customized oligonucleotides were purchased from LGC Biosearch Technologies, Microsynth AG and Hitgen, analysed by LC-MS and quantified with a Nanodrop 2000c spectrophotometer. Water was purified with a Millipore Milli-Q system (MQ, Merck). Gene synthesis was performed by Twist Bioscience. Sequencing service was provided by Microsynth AG.

### Cloning, protein production and purification

Genes encoding CoA ligases or NATs containing an N-terminal His-tag were purchased in a pET28b(+) vector. The plasmids were transformed into *E. coli* BL21(DE3), and cells were plated on a Luria-Bertani (LB) agar plate containing 50 µg ml^−1^ kanamycin (kan). A single colony of freshly transformed cells was incubated at 37 °C overnight in 3 ml of LB medium containing 50 µg ml^−1^ kan. Glycerol stocks (30% glycerol in LB) of BL21(DE3) harbouring the target genes were stored at −80 °C until further use.

To produce the proteins of interest, 20 ml of LB medium containing 50 µg ml^−1^ kan was inoculated from the corresponding glycerol stock in a 100-ml shaking flask. The cultures were incubated overnight using 120 rpm and 37 °C (Infors HT multitron, 50 mm shaking amplitude). Next, 10 ml of the overnight cultures were used to inoculate 190 ml of Zym-5052 autoinduction medium containing 50 µg ml^−1^ kan in a 1-l shaking flask. The culture was incubated for at least 24 h at 20 °C and 120 rpm (Infors HT multitron, 50 mm shaking amplitude). The cell pellet was collected by centrifugation (15,000*g*, 15 min) in a 50-ml falcon tube and was stored at −20 °C for at least 12 h (overnight) before lysis. For the lysis, the cell pellet was thawed on ice and resuspended in 5 ml of buffer A (50 mM Tris-Cl, pH 7.4, 20 mM imidazole, 500 mM NaCl). Cells were lysed by sonication (2 × 1 min, 2-s pulses, 40% amplitude, on ice: Sonopuls, Bandelin). The solid cell material was removed by centrifugation (15,000*g*, 15 min, 4 °C) and the supernatant was filtered by passing it through a 0.45-µm polysulfone filter membrane. Enzyme purification was conducted on an ÄKTA pure system (Cytiva Life Sciences) by applying the clarified lysate to a 5-ml equilibrated HisTrap-column (Cytiva Life Sciences). The column was washed with at least 5 column volumes of buffer A and 5 column volumes of 10% buffer B (50 mM Tris-Cl, pH 7.4, 200 mM Imidazole and 500 mM NaCl) to separate any low-affinity binding proteins before eluting the protein of interest with 100% buffer B. The target protein-containing fraction was concentrated by ultrafiltration (Amicon Ultra, molecular weight cut-off 10 kDa) and desalted in 50 mM Tris buffer (pH 8.0) using a HiTrap desalting column (Cytiva Life Sciences) before snap-freezing in liquid nitrogen. The enzyme stocks were stored at −80 °C until further use.

### Synthesis of on-DNA substrates

To synthesize on-DNA substrates **a**–**aa**, corresponding activation solutions were prepared by mixing 175 µl of dimethylsulfoxide (DMSO), 32 µl of amine **A**, **C**, **D**, **F** solution (200 mM in DMSO), 32 µl of 1-ethyl-3-carbodiimide (EDC; 100 mM in DMSO) and 160 µl of s-NHS solution (100 mM in 2:1 DMSO: water). The activation solution was shaken at 37 °C for 30 min and added to a solution of 100 nmol 14-mer or 48-mer amino-modified oligonucleotide in 100 µl of water and 100 µl MOPS buffer (pH 8, 100 mM MOPS, 1 M NaCl). The reaction mixture was shaken overnight at 37 °C before ethanol precipitation was carried out. For larger scales (up to 500 nmol), a linear scale-up was performed.

For Fmoc deprotection, an aqueous solution of the Fmoc-protected oligonucleotide conjugates (100–200 µl) was added to an equal volume of 20% (v/v) piperidine in MQ water. The solution was shaken at 400 rpm at 30 °C for 2 h (HCM100-Pro Thermo Mix, DLAB Scientific). Volatile components were evaporated in a vacuum concentrator; the residual solids were redissolved in water and oligonucleotide conjugates were subjected to ethanol precipitation before further use.

For azide reduction, 100–500 nmol of the relevant DNA conjugates were dissolved in 100 µl of water before 100 µl of Tris(2-carboxyethyl)phosphine solution (200 mM in 500 mM Tris.HCl, pH 7.4) was added and the mixture was shaken at 400 rpm and 37 °C for 5 h (HCM100-Pro Thermo Mix, DLAB Scientific). Following the reaction, the samples were ethanol precipitated before further use.

DNA conjugates were purified by preparative reverse-phase-HPLC and desalted using centrifugal filter tubes (Amicon Ultra, molecular weight cut-off 3 kDa) before biocatalysis.

Double-stranded DNA conjugates were prepared by mixing DNA conjugates in an equimolar ratio with the complementary 14-mer oligomer. Hybridization was triggered by heating to 75 °C for 10 min, followed by slow cool-down to room temperature. Analysis of successful hybridization was performed by agarose gel electrophoresis (4% (w/v) agarose).

### Enzymatic on-DNA amide bond formation

Standard enzymatic reactions to form on-DNA amides contained 2.5 mM ATP (100 mM stock in water pH 7.5), 0.025 mM CoA (2.5 mM stock in water), 0.1 mM carboxylic acid (200 mM stock in DMSO or 20 mM stock in water), 0.05 mM on-DNA amine (1 mM stock in water), 20 µM CoA ligase and 20 µM NAT in 50 mM Tris buffer (pH 8.0). The final reaction volume was 20 µl. Biocatalytic reactions were carried out for 20 h at 25 °C and 450 rpm (Thermomixer, Eppendorf). Reactions were quenched by heat shock (90 °C, 5 min) and diluted with water:acetonitrile (ACN) and centrifuged (15,000*g*, 30 min) before LC-MS analysis.

### LC-MS analysis of oligonucleotides

Oligonucleotide conjugates were analysed by electrospray ionization-time of flight-mass spectrometry on a Waters Xevo G2-XS QTof instrument after separation on a Waters Acquity UPLC H-Class System fitted with an Acquity Premier Oligonucleotide BEH C18 column (130 Å 1.7 µM, 2.1 × 50 mm) used for analysis of DNA conjugates and an Acquity UPLC Peptide BEH C18 column (300 Å 1.7 µM, 2.1 × 50 mm) used for analysis of biocatalysis samples, both from Waters. LC separation was performed at 60 °C and a flow rate of 0.5 ml min^−1^. Eluent A consisted of 0.4 M hexafluoroisopropanol and 5 mM triethylamine in water; methanol was used as eluent B. Two gradients were used. The standard method started with 100% eluent A for 0.5 min, followed by a linear increase to 50% eluent B over 6 min and a further increase to 90% eluent B over 0.5 min, then a return to 100% eluent A over 0.5 min, which was maintained for 0.5 min. A second method was used for analysing more hydrophobic compounds: after starting with 100% eluent A for 0.5 min, a linear increase to 70% eluent B over 6.5 min and a further increase to 95% eluent B over 0.9 min followed, ending with a return to 100% eluent A over 0.1 min, which was maintained for 0.9 min. Conversions to product were determined from peak areas measured at a wavelength of 260 nm.

### Molecular analysis of 05PaAT and 42GmAT

To rationalize why 42GmAT’s activity towards on-DNA substrate was superior to 05PaAT, we investigated and compared substrate binding site characteristics of both enzymes. At first, we characterized the pocket size and volume using pyKVFinder Python package (v.0.8.2)^67^. Crystal structures of 05PaAT and 42GmAT (PDB 1W4T and 7QI3, respectively) were used as input. Before cavity detection, MDTraj (v.1.10.3) was used to remove water and ions, as well as to separate duplicate chains in 7QI3. Open-source PyMol (v.3.1.0) was used in a Python script, using the cmd module, to superpose the structures onto 1W4T and ensure comparable protein orientation. Cavity detection was performed with adapted probe out and volume cut-offs (8 Å and 50 Å, respectively) to ensure that only pockets with significant volume were detected. All other parameters were set to the default values. The detected pockets were manually investigated to select the cavity that comprises the known catalytic site. Finally, volume and surface area of this cavity were extracted, and the substrate binding pocket was visualized in mesh representation using PyMol (v.3.1.0)^85^.

Next, we opted to model the biomolecular complex of the NATs with DNA-tagged substrate **f** using Boltz-2 (v.2.2.1)^63^. For this purpose, yaml files containing protein sequences and substrate SMILES were prepared and the prediction was performed with 30 diffusion samples (-diffusion_samples 30) using ColabFold^86^ multiple sequence alignment server (-use_msa_server) and adding inference-time potentials to improve the physical plausibility (-use_potentials) when applying Boltz-2x. The 30 resulting complexes were superposed clustered by their root mean square deviations using MDTraj (v.1.10.3)^87^ and scikit-learn (v.1.7.2)^88^ Python libraries. Although the default Boltz-2 workflow returns a single conformation, we increased the sampling depth to 30 thoroughly probe conformational heterogeneity and to stabilize ensemble-derived structural descriptors. The value of *N* = 30 was chosen on the basis of common statistical practice and computational efficiency and validated by a retrospective analysis (Supplementary Figs. 31 and 32). Cofolding of screening substrate **E** and double-stranded DNA substrate **g** was performed analogously.

### 42GmAT and 05PaAT chimera engineering

FastCloning PCR^89^ was used to generate 42GmAT and 05PaAT chimera enzymes (loop swap) with primer pairs 42GmAT_05PaAT or 05PaAT_42GmAT (Supplementary Table 7) and pET28b plasmids harbouring 42GmAT or 05PaAT as templates. Following PCR conditions were used: 10 µl of Q5 reaction buffer, 4 µl of dNTPs (10 mM), 2.5 µl (+)primer (10 µM), 2.5 µl (−)primer (10 µM), 0.5 µl of template and 0.5 µl of Q5 polymerase, with the addition of MQ water to reach a final volume of 50 µl. The PCR was performed according to the manufacturer’s recommendation using an annealing temperature of 60 °C and 30 cycles (Supplementary Table 8). Following DpnI digest (1 µl of DpnI, at 37 °C for 1 h 40 min, deactivation for 20 min at 80 °C), PCR products were transformed into *E. coli* NEB10-β. Sequences were validated by Sanger sequencing.

### Creation of 42GmAT site-saturation libraries

NNK site-saturation libraries of 42GmAT were constructed by overlap extension PCR as previously reported^90^ using primers pET28(+), pET28(−) and the corresponding NNK primer pairs (M139NNK, P141NNK, S178NNK, M179NNK, Supplementary Table 7). The libraries were subcloned into a pET28b vector containing the negative selection marker *sacB* using restriction sites for XhoI and NcoI and ligated with T4 ligase according to the supplier’s instructions. Subsequently, the libraries were transformed into *E. coli* NEB10-β before plating on LB-Agar (50 µg ml^−1^ kan, 5% (w/v) sucrose, 1 mM isopropyl-β-D-thiogalactoside). Library quality was confirmed by Sanger sequencing. Following library plasmid isolation with NucleoSpin Plasmid Easy Pure Kit, *E. coli* BL21(DE3) cells were transformed with the genetic material. Per library, 89 single colonies were picked to inoculate 1 ml of LB media (50 µg ml^−1^ kan) in a 96-deep well plate. Three wells on the plate were inoculated with cells carrying the empty vector only (no gene of interest) and three wells with cells harbouring wild-type NAT 42GmAT. In addition, one position was carried forward without any cell material (no enzyme control). In parallel to NAT library production, a plate uniformly inoculated with CoA ligase ipfF was generated. The plates were incubated overnight at 37 °C and 300 rpm (Duetz system, 50 mm shaking amplitude). As a next step, 50 µl of each well from the preculture plate was used to inoculate 950 µl of Zym-5052 on the main culture plate, which was incubated at 20 °C for 24 h. Plates were centrifuged (3,428*g*, 20 min at 4 °C) and the supernatant was discarded before the main culture plate was being stored for at least 12 h (overnight) at −20 °C.

For library screening, plates were thawed at room temperature, and lysis was initiated by adding 50 µl per well lysis buffer (50 mM Tris pH 8.0), 1 mg ml^−1^ lysozyme, 0.5 mg ml^−1^ polymyxin B and DNAseI) to the site-saturation plates and 200 µl per well lysis buffer to the CoA ligase plate. Lysis was carried out for 20 min at 25 °C and 300 rpm. After combining 50 µl of lysate of each NNK library variant with 50 µl of ipfF lysate, the biocatalytic reaction was initiated by adding 100 µl of 50 mM Tris buffer (pH 8.0) containing ATP, CoA, amine **E** and carboxylic acid **1** yielding the following final concentrations: 0.625 mM amine **E**, 1.25 mM carboxylic acid **1**, 2.5 mM ATP and 0.025 mM CoA. After 20 h of incubation at 300 rpm and 25 °C, the reaction was quenched by the addition of 800 µl of ACN (5 min of incubation, 25 °C). Reaction plates were centrifuged (15 min, 3,428*g*) and each well’s supernatant was analysed with reverse-phase chromatography on a 1290 Infinity II Agilent system fitted with a single quadrupole (electrospray ionization). A Poroshell 120 EC-C18 column (2.7 µm, 2.1 × 50 mm) from Agilent was used for analysis. LC separation was performed at 40 °C and a flow rate of 0.8 ml min^−1^. Eluent A consisted of 5% (v/v) ACN with 0.2% (v/v) formic acid. Eluent B consisted of 100% ACN with 0.2% (v/v) formic acid. The method started with 100% eluent A for 0.15 min, followed by a linear increase to 95% eluent B over 2 min, which was maintained for 0.35 min, followed by a return to 100% eluent A over 0.5 min. Substrates and products were detected by mass spectrometry scan (*m/z* 300–510) and single ion monitoring (SIM) ([M+H]+), targeting the expected *m/z* for products and substrates.

### Construction of combinatorial 42GmAT libraries

Site-directed mutagenesis PCR was used to combine mutations M139L, M139F and M139Y with M179W. To this end, pET28b plasmids harbouring *42GmAT* genes with L, F or Y at position 139 were used as templates and amplified with primers containing the M179W mutation (M179(+) and (−), (Supplementary Table 7) using the following PCR conditions: 10 µl of Q5 reaction buffer, 3 µl of dNTPs (10 mM), 2.5 µl of (+)primer (10 µM), 2.5 µl of (−)primer (10 µM), 0.5 µl of template, 0.5 µl of Q5 polymerase, with the addition of MQ water to reach a final volume of 50 µl. The PCR was performed according to manufacturer’s recommendation using an annealing temperature of 61 °C and 25 cycles (Supplementary Table 9). Following DpnI digest (1 µl of DpnI, at 37 °C for 1 h 40 min, deactivation for 20 min at 80 °C), PCR products were transformed into *E. coli* NEB10-β. Sequences were validated by Sanger sequencing.

### Malachite Green assay

To assess CoA ligase substrate scope, a library of carboxylic acids was assembled from an in-house collection containing >1,000 molecules. As a first step, molecular fingerprints (Morgan fingerprints)^91^ of all available in-house carboxylic acids were generated and compared to the fingerprints of the literature-reported substrates of CoA ligase ipfF^43^. Following calculation of Tanimoto similarity^72^, the 100 most and least similar molecules to reported enzymatic substrates were selected and labelled as ‘high similarity substrates’ and ‘low-similarity substrates’, respectively. In addition, 100 carboxylic acids difficult to couple chemically to amino-modified 14-mer oligonucleotides (reported conversions <50%)^73^ were chosen and labelled as ‘low conversion substrates’. After removal of duplicates, this afforded the final set of 236 carboxylic acids.

The activity of CoA ligases ipfF and LcCL for the set of 236 carboxylic acids was determined via the Malachite Green assay^92^. Reactions were carried out in a 96-well plate, with each well containing 2 µM CoA ligase, 0.4 U ml^−1^ pyrophosphatase, 0.1 mM carboxylic acid (200 mM stock in DMSO), 1 mM ATP, 1 mM CoA and 5 mM MgCl_2_ in 25 µl of 50 mM Tris (pH 8.0). The reactions were incubated at 25 °C for 90 min. Following reaction, 2.5 µl of the reaction mix was diluted with 50 mM Tris (pH 8.0), to a volume of 50 µl in a clear F-bottom Greiner plate. The Malachite Green assay was carried out according to the supplier’s instructions: 10 µl of Malachite Green Reagent A was added to the 50-µl sample and shaken for 10 min at room temperature before adding 10 µl of Malachite Green Reagent B. The plate was loaded on the Tecan Spark plate reader and shaken for 15 s to mix components before incubating without shaking for 20 min at 30 °C. Absorbance was measured at a wavelength of 630 nm with 15 flashes per well at 30 °C. All reactions were performed as three technical replicates and corrected against a general blank reaction containing no CoA ligase. The phosphate calibration curve (Supplementary Fig. 22) was generated according to the supplier’s instructions and was used to quantify phosphate concentration that served as a measure for carboxylic acid activation.

### qPCR experiments

For qPCR experiments, 7.5 µl of 50 mM Tris buffer (pH 8.0) containing enzyme (ipfF; ipfF/05PaAT; ipfF/42GmAT; ipfF/42GmAT M139Y) as well as no enzyme control (7.5 µl of 50 mM Tris buffer, pH 8.0) preincubated at 25 °C were added to 7.5 µl of 50 mM Tris buffer (pH 8.0) containing ATP, CoA, carboxylic acid **1** and amine **aa**, including a amplifiable DNA tag (48-mer oligonucleotide, Supplementary Table 5 entry 13). Final reaction concentrations were; 2.5 mM ATP, 0.025 mM CoA, 0.05 mM on-DNA amine **aa** and 0.1 mM carboxylic acid **1**. The reactions were incubated for 20 h at 25 °C (HCM100-Pro Thermo Mix, DLAB Scientific) and quenched by heat shock (90 °C, 1 min). Solutions were diluted to a concentration of 1 µM and directly used for the qPCR experiments. All reactions were prepared in triplicate.

Quantitative PCR (qPCR) of the control samples was performed using PowerTrack SYBR Green Master Mix (Applied Biosystems) on a QuantStudio 7 Flex Real-Time PCR System (Applied Biosystems) in 10 µl sample volume and according to the manufacturer’s instructions with an annealing temperature of 60 °C using 40 cycles (Supplementary Tables 17). A standard curve based on a serial dilution (10^−3^–10^−6^ µM) of the substrate was prepared. All qPCR reactions were prepared and measured in quadruplicate for each of the enzymatic reaction samples.

### Reporting summary

Further information on research design is available in the Nature Portfolio Reporting Summary linked to this article.

## Supplementary information

Supplementary Information Supplementary Methods, Tables 1–17, Figs. 1–32 and Appendix.

Reporting Summary.

Peer Review File

Supplementary Data 1 Source Data for Supplementary Fig. 20.

Supplementary Data 2 List of commercial chemicals, enzymes, materials and solvents including supplier and catalogue number.

Supplementary Data 3 Supplementary Table 12 with chemical structures embedded.

## Source data

Source Data Fig. 2 Source Data for Fig. 2.

Source Data Fig. 3 Source Data for Fig. 3.

Source Data Fig. 4 Source Data for Fig. 4.

Source Data Fig. 5 Source Data for Fig. 5.

Source Data Extended Data Fig. 1 Source Data for Extended Data Fig. 1.

Source Data Extended Data Fig. 3 Source Data for Extended Data Fig. 3.

Source Data Extended Data Fig. 4 Source Data for Extended Data Fig. 4.

## Data Availability

Nucleotide and amino acid sequences of enzymes as well as UniProt accession codes can be found in the Supplementary Information. The crystal structures used in bioinformatic experiments can be accessed via PDB ID 7QI3 (42GmAT) and 1W4T (05PaAT). Input data required to reproduce the Boltz-2 modelling and binding energy analyses, as well as the corresponding output data, are available via GitHub at https://github.com/Buller-Lab/EnzyDEL_NATs. Experimental raw data and methods are provided with this paper, the supplementary information or are available from the authors upon request. Source data are provided with this paper.

## Code availability

All Python scripts used for the bioinformatic analysis are available via GitHub at https://github.com/Buller-Lab/EnzyDEL_NATs and via Zenodo at 10.5281/zenodo.20138090 (ref. ^93^).
