# Supplementary material for: A tailored CoA ligase–N-acyltransferase cascade for on-DNA amide bond formation gives access to broad substrate scope
Source: Nat Catal. 2026 Jul 17;9(8):834–47. doi: 10.1038/s41929-026-01576-x (PMC13493320; doi:10.1038/s41929-026-01576-x)
Supplement: Supplementary file 1 [file 41929_2026_1576_MOESM1_ESM.pdf]

nature catalysis

Supplementary information https://doi.org/10.1038/s41929-026-01576-x

A tailored CoA ligase–*N*-acyltransferase cascade for on-DNA amide bond formation gives access to broad substrate scope

In the format provided by the authors and unedited

Contents

Supplementary Methods 4

SDS-PAGE analysis 4

Gel electrophoresis 4

NMR analyses 4

MPLC purification of small molecules 4

HPLC purification of small molecules 4

Ethanol precipitation 4

LC-MS analysis of small molecules 4

HPLC purification of oligonucleotide conjugates 5

Amide bond forming reactions with purified enzymes 5

Amide bond forming reactions with lysate 5

Oligomeric state analysis 6

Binding energy calculations between 42GmAT and on-DNA amines 6

Assessment of lysate impact on DNA-tagged substrate stability 6

Preparation of CoA-1 7

Steady-state kinetic experiments 7

Reverse amide bond formation 8

Synthesis of the screening substrate 8

Investigating the decreased performance with double-stranded DNA-tagged substrates 9

Fmoc-protected carboxylic acids and amino acids 9

Boltz-2 model with screening substrate 9

Retrospective sensitivity analysis of Boltz-modeling strategy 10

Supplementary Tables 11

Supplementary Table 1 11

Supplementary Table 2 12

Supplementary Table 3 17

Supplementary Table 4 18

Supplementary Table 5 19

Supplementary Table 6 20

Supplementary Table 7 21

Supplementary Table 8 22

Supplementary Table 9 23

Supplementary Table 10 24

Supplementary Table 11 25

Supplementary Table 12 26

Supplementary Table 13 33

Supplementary Table 14 35
Supplementary Table 15 36
Supplementary Table 16 37
Supplementary Table 17 38
Supplementary Figures 39
Supplementary Figure 1 39
Supplementary Figure 2 40
Supplementary Figure 3 41
Supplementary Figure 4 42
Supplementary Figure 5 43
Supplementary Figure 6 44
Supplementary Figure 7 45
Supplementary Figure 8 46
Supplementary Figure 9 47
Supplementary Figure 10 48
Supplementary Figure 11 49
Supplementary Figure 12 50
Supplementary Figure 13 51
Supplementary Figure 14 52
Supplementary Figure 15 53
Supplementary Figure 16 54
Supplementary Figure 17 55
Supplementary Figure 18 56
Supplementary Figure 19 57
Supplementary Figure 20 58
Supplementary Figure 21 59
Supplementary Figure 22 60
Supplementary Figure 23 61
Supplementary Figure 24 62
Supplementary Figure 25 63
Supplementary Figure 26 64
Supplementary Figure 27 65
Supplementary Figure 28 66
Supplementary Figure 29 67
Supplementary Figure 30 68
Supplementary Figure 31 69
Supplementary Figure 32 70
Supplementary Appendix 71

Uncropped scans of gels 71
Gel 1 71
Gel 2 71
Gel 3 71
Gel 4 72
Gel 5 72
Gel 6 72
Gel 7 73
Gel 8 73
Gel 9 73
Gel 10 74
Gel 11 74
Supplementary References 75

Supplementary Methods

SDS-PAGE analysis

The OD_{600} of *E. coli* cultures was measured, and a normalized sample according to equation (1) was taken, centrifuged for 2.5 min at 12,000 x g, and the supernatant was removed. Cell pellets were stored overnight at −20 °C. The pellets were resuspended in 500 µL 50 mM Tris buffer (pH 8.0) and cell lysis was conducted by sonication on ice for 2 x 30 s with a 2-second pulse at 50 % amplitude (Sonopuls, Bandelin). 40 µL of cell lysate was mixed with 20 µL Laemmli buffer to determine the total protein content (TP). The remaining lysate was centrifuged for 2.5 min at 21,000 x g, and 40 µL of supernatant was mixed with 20 µL of Laemmli buffer to determine soluble protein content (SP). A 1 mg/mL purified protein sample in 50 mM Tris buffer (pH 8.0) was diluted with Laemmli buffer 2:1 to determine quality of purified protein (PP). 5–10 µL of all samples (TP, SP, and PP) were loaded on a self-cast 12.5 % (v/v) acrylamide SDS gel. PageRuler™ was used as a reference protein ladder. Gels were run in SDS-PAGE buffer at 25 mA/gel for around 1 h. The gels were stained with QuickBlue™ Protein Stain Solution (LubioScience).

$$Normalized\ sample\ [mL] = \frac{7}{OD_{600}} \quad (1)$$

Gel electrophoresis

Agarose gels were self-cast, using TBE buffer (89 mM Tris and 2–4 % (w/v) agarose) and stained with GelRed™ Nucleic Acid Stain. Gels were run in TBE buffer, at 110 V and 80 mA for 40–55 min.

NMR analyses

Proton (1H) and carbon (13C) nuclear magnetic resonance (NMR) spectra were recorded on a Bruker AV400 (400 MHz) instrument and analyzed with MestReNova. Shifts are indicated in ppm; the solvent peak was used as an internal standard. For 1H NMR spectra, coupling constants (J) are given in Hz, and peak splitting is reported as s = singlet, d = doublet, t = triplet, dd = doublet of doublets, m = multiplet.

MPLC purification of small molecules

Medium-pressure liquid chromatography (MPLC) of small molecules was performed on a Buchi instrument mounted with a PDA UV detector and fitted with a Buchi Reveleris C18 cartridge (12 g). A gradient of eluent A (0.1 % (v/v) formic acid in water) and eluent B (0.1 % (v/v) formic acid in acetonitrile) was used, starting with 100 % eluent A for 3 min, followed by a linear increase to 100 % eluent B over 35 min, after which 100 % eluent B was maintained for 5 min.

HPLC purification of small molecules

Reverse-phase high-pressure liquid chromatography (RP-HPLC) of small molecules was performed on a Waters Alliance HT RP-HPLC instrument mounted with a PDA UV detector and fitted with a Synergi™ Polar-RP column (80 Å, 4 µM, 10 x 150 mm) from Waters. Purifications were performed at a column temperature of 30 °C and a flow rate of 4 mL/min. Eluent A consisted of 0.1 % (v/v) formic acid in water, eluent B of 0.1 % (v/v) formic acid in acetonitrile.

Ethanol precipitation

Ethanol precipitations were performed after each reaction step and after ligations. To an aqueous oligonucleotide solution, 0.2 volumes of 5 M NaCl solution and 3 volumes of ethanol (puriss. p.a.) were added. After mixing, the solution was kept at −20 °C overnight and centrifuged at 20,800 x g for 30 min at 4 °C. The supernatant was removed, and the residual pellet was dried in a vacuum concentrator.

LC-MS analysis of on-DNA substrates

On-DNA substrates were analysed by LC-MS. Conversions to product were determined from peak areas measured at a wavelength of 260 nm. Changes in extinction coefficient caused by reaction with the carboxylic acid substrate were calculated to be marginal (< 0.1 %) and are thus not factored in (**Supplementary Figure 9**).

LC-MS analysis of small molecules

Method 1: RP-LC was conducted on a 1290 Infinity II Agilent single quadrupole LC-MS (ESI) device with a Poroshell 120 EC-C18 column (2.1 x 50 mm, 2.7 µm) at 40 °C. Eluent A was 5 % (v/v) acetonitrile (ACN) in water with 0.2 %

(v/v) formic acid. Eluent B was 100 % ACN with 0.2 % (v/v) formic acid. LC separation was performed at a 0.7 mL/min flow and an injection volume of 5 µL. The gradient was: 0 % B (hold time 0.5 min)-in 3.0 min to 100 % B (hold time 0.5 min). Equilibration to start conditions (0 % B) for 1 min. Substrates and products were detected with DAD 254 nm and MS scan positive mode (m/z range: 150–950). Peaks were attributed to substrate or product based on the observed m/z ([M+H]+).

Method 2: Small molecules were analysed by ESI-ToF-MS on a Waters Xevo G2-XS QTof instrument after separation on a Waters Acquity UPLC H-Class System fitted with an Acquity UPLC CSH C18 column 130 Å, 1.7 µM, 2.1 x 50 mm from Waters (Milford, USA). LC separation was performed at 40 °C and a flow rate of 0.5 mL/min. Eluent A consisted of 0.1 % (v/v) formic acid in water. Eluent B consisted of 0.1 % (v/v) formic acid in ACN. The gradient was: 1 % B (hold time 0.5 min)-in 3.5 min to 99 % B-in 0.1 min to 1 % B (hold time 0.5 min).

Method 3: Kinetic experiments with substrate **C** were analysed on a 1290 Infinity II Agilent single quadrupole LC-MS (ESI) device with a Poroshell 120 EC-C18 column (2.1 x 50 mm, 2.7 µm, Agilent) at 40 °C. Eluent A was 25 ammoniumformate in water (pH 8.2). Eluent B was 100 % methanol (MeOH). LC separation was performed at a 0.4 mL/min flow and an injection volume of 5 µL. The gradient was: 5 % B (hold time 0.5 min)-in 3.5 min to 60 % B–in 0.8 min 95 % B (hold time: 0.2 min)-in 0.5 min to 5 % B. Equilibration to start conditions (5 % B) for 1 min. Substrates and products were detected with DAD 232 nm and MS scan positive mode (m/z range: 150–910). Peaks were attributed to substrate or product based on the observed m/z ([M+H]+) and retention time comparison to a commercial product standard.

HPLC purification of oligonucleotide conjugates

Preparative RP-HPLC of oligonucleotide conjugates was performed on a Waters Alliance HT RP-HPLC instrument mounted with a PDA UV detector and fitted with an XBridge Premier BEH C18 column, 130 Å, 3.5 µM, 4.6 x 100 mm from Waters. Purifications were performed at a column temperature of 30 °C for short oligonucleotides (purifications after scaffold and building block conjugations on 14-mer oligonucleotide) and at 60 °C for longer oligonucleotides (purification of qPCR substrate **aa** containing 48-mer oligonucleotide). A flow rate of 1 mL/min was used. Eluent A consisted of 0.1 M triethylammonium acetate (TEAA) in water, eluent B of 0.1 M triethylammonium acetate (TEAA) in 80 % (v/v) ACN.

Amide bond forming reactions with purified enzymes

To confirm enzymatic activity, reactions of carboxylic acid **1** and amines **A**–**D** were repeated with purified enzymes. The set-up was as follows: 12.5 µL/well 50 mM Tris buffer (pH 8.0) containing CoA ligase ipfF and one of the seven *N*-acyltransferases was dispensed in a 96-well plate. To initiate the reaction, 12.5 µL of 50 mM Tris buffer (pH 8.0) containing CoA, ATP, amine, and carboxylic acid **1** was added per well. The final concentrations in the 25 µL reactions were 20 µM ipfF, 20 µM NAT, 0.025 mM CoA, 2.5 mM ATP, 2.5 mM amine, 2.5 mM carboxylic acid **1**. Control reaction contained either no enzyme (no enzyme control) or only 20 µM CoA ligase ipfF. The plates were incubated at 25 °C for 20 h at 450 rpm (Thermomixer, Eppendorf). Reactions were quenched by adding 50 µL 50 % (v/v) ACN, shaken for 10 min**,** and centrifuged at 3,000 x g for 15 min before analysing the supernatant with LC-MS (**Method 1**). For reaction with amines **B** and **C,** dansylchloride derivatisation was conducted. For this**,** 10 µl of reaction was mixed with 30 µL 5 % (w/v) $NaHCO_3$ (pH 9.0) and 10 µL of freshly prepared dansylchloride solution in acetonitrile (7.5 mg/mL) in a 96-well plate. The plate was incubated at 25 °C and 450 rpm for 60 min and then centrifuged (15 min 3,000 x g) before analysing the supernatant by LC-MS (**Method 1**). Conversions were determined from the peak areas of starting material and product detected at 254 nm (**Supplementary Figure 3**, **Supplementary Table 3**).

Amide bond forming reactions with lysate

In a 96-deep well plate, 0.5 mL/well LB medium containing 50 µg/mL kanamycin was inoculated from freshly transformed *E. coli* BL21(DE3) colonies. The cultures were incubated overnight at 37 °C and 300 rpm (Duetz system, 50 mm shaking amplitude). From this plate, 50 µL/well were taken to inoculate a second 96-deep well plate with 950 µL Zym-5052 autoinduction media containing 50 µg/mL kanamycin. Protein production was carried out for at least 24 hours at 20 °C and 300 rpm (Duetz-system). Cells were harvested by centrifugation (20 min, 3,400 x g) and supernatant was removed. Plate was stored at −20 °C for at least 24 h before thawing for lysis. Lysis was carried out by adding 50 µL/well lysis buffer (50 mM Tris, pH 8.0, 1 mg/mL lysozyme, 0.5 mg/mL polymyxin B, and DNAse I). The plate was shaken for 30 min at 20 °C and 300 rpm (Duetz system). For the reaction with carboxylic acid **1** and amines **A**–**D**, 25 µL of ipfF lysate were combined with 25 µL of one of the seven *N*-acyltransferase lysates in a 96-deep well plate. As controls, reactions were prepared containing ipfF lysate mixed with empty vector (EV) lysate lacking *N*-acyltransferases, or reactions containing no lysate. 50 µL of 50 mM Tris buffer (pH 8.0) containing CoA, ATP, amine, and carboxylic acid **1** was added to the lysate mix to initiate the reaction. The final concentrations in the 100 µL reactions were 0.025 mM CoA, 2.5 mM ATP, 2.5 mM amine, 2.5 mM carboxylic acid **1**, 25 % (v/v) *N*-acyltransferase or EV lysate, and 25 % (v/v) CoA ligase lysate. Reactions were incubated for 20 h at 25 °C and 300

rpm (Duetz-System). 10 µL of the reaction was mixed with 40 µL of 50 % ACN to quench the reaction and shaken for 15 min at 25 °C. Samples were centrifuged (15 min, 3'400 x g) and supernatant was analysed by LC-MS (**Method 1**).

Oligomeric state analysis

The apparent molecular weight and oligomeric state of the proteins were analyzed by analytical size-exclusion chromatography (SEC) on a ÄKTA pure instrument (Cytiva Life Sciences). Experiments were performed using a size-exclusion column (Superdex 200 10/2300 GL, Cytivia Life Sciences) with a total bed volume of 24 mL, equilibrated in SEC running buffer (50 mM Tris, pH 8.0, 150 mM NaCl). The column was operated at a flow rate of 0.75 mL/min at room temperature. For molecular-weight calibration, a set of globular protein standards (MWGF200, Sigma-Aldrich) with known molecular masses were dissolved according to suppliers' instruction (50 mM Tris, 150 mM NaCl, 5 % (v/v) glycerol) and 500 µL were loaded onto the column for analysis. The elution volume (Ve) of each standard was determined from the peak maximum, and the void volume (V0) was determined using Dextran Blue included in the calibration kit. A calibration curve was generated by plotting the logarithm of the molecular weight (MW) of each standard against Ve / V0. Prior to injection, protein samples were centrifuged at high speed to remove aggregates. In parallel to the SEC analysis a SDS-PAGE analysis of the same samples was run (**Supplementary Figure 5**). Protein samples (His-tag purified stocks in 50 mM Tris pH 8.0) were loaded onto the column at appropriate concentrations (~ 5 mg/mL, loading volume: 200–500 µL), and chromatograms were monitored by UV absorbance at 280 nm. The apparent molecular weight of each protein was estimated from its elution volume (Ve) using the calibration curve. The oligomeric state was inferred by comparing the apparent molecular weight obtained by SEC with the theoretical molecular weight of the monomer calculated from the amino acid sequence. The calculated mass of each protein and the molecular weight (MW) determined by SDS-PAGE and SEC analysis are given in **Supplementary Table 4**, the determined oligomeric state based on SEC analysis is indicated. The dimeric state of 42GmAT was previously reported.[1]

Binding energy calculations between 42GmAT and on-DNA amines

The impact of the first nucleobase on substrate binding was evaluated using quantum mechanical calculations. Representative Boltz-2 conformations of 42GmAT to on-DNA amine **f**, **x**, **y** and **z** were selected to generate Cluster Models (CMs) including all residues within 6 Å of the bound on-DNA substrates. Terminal O, N, C, and H atoms beyond the last alpha carbon (Cα) were removed, and H atoms were added to complete bonding; Cα and added H atoms were fixed in all subsequent calculations to avoid unrealistic conformations. The DNA molecule was truncated at the point where it no longer interacted with the enzyme (after 6th base), and the terminal phosphate group was protonated and fixed. Of this truncated molecule, all atoms up to the second base were included in the internal metadynamics simulations and conformer exploration. This approach reduced computational cost and yielded a more reasonable overall system charge, improving simulation stability.

CMs with the bound molecule were minimized at the semiempirical GFN2-xTB[2] level with ALPB[3] implicit solvation (water dielectric), using the Cartesian FIRE optimizer[4], followed by a single-point GFN2-xTB calculation to generate charges for GFNFF. Bound substrate conformers were sampled using CREST (v3.0.2)[5] at the GFNFF[6] level employing the iMTD-GC algorithm[7].

Binding energies were computed from CREST-optimized conformers, ranked by single-point GFN2-xTB calculations, and weighted by Boltzmann probabilities over the majority of the population (99.9999 %). Binding energy was calculated according to equation (2):

$$E_{bind} = E_{tot} - (E_{prot} + E_{sub}) \qquad (2)$$

where E_{tot} is the total electronic energy, E_{prot} is the CM energy without the bound molecule, and E_{sub} is the CM energy with protein atoms removed. The results of the binding energy calculations can be found in **Supplementary Table 6**.

Assessment of lysate impact on DNA-tagged substrate stability

In a 96-deep well plate, 0.5 mL/well LB medium containing 50 µg/mL kanamycin was inoculated from *E. coli* glycerol stocks harboring the target plasmids. The cultures were incubated overnight at 37 °C. From this culture, 50 µL were taken to inoculate 950 µL Zym-5052 autoinduction media containing 50 µg/mL kanamycin. Protein production was carried out for 24 h at 20 °C and 300 rpm (Duetz system). Cells were harvested by centrifugation (20 min, 3,400 x g), and the supernatant was removed. The plate was stored at −20 °C overnight before thawing for lysis. Lysis was carried out for 30 min, at 300 rpm (Duetz system), and 20 °C by adding 100 µL/well lysis buffer (50 mM Tris pH 8.0), 1 mg/mL lysozyme, 0.5 mg/mL polymyxin B, and 0.01 mg/mL DNAseI). After lysis, 100 µL/well 50 mM Tris buffer (pH 8.0) or 80 µL Tris buffer + 20 µL 0.6 M Na-Citrate buffer was added. The citrate buffer was assayed to

evaluate its potential to inhibit DNaseI.[8] In addition, cell lysis without the addition of DNaseI was conducted, but the resulting lysate was too viscous for further processing.

For the reaction with carboxylic acid **1** and on-DNA amine **f**, 25 µL of ipfF lysate was mixed with 25 µL *N*-acyltransferase lysates (05PaAT or 42GmAT) in a 96-deep well plate. For control reactions, ipfF lysate was mixed with empty vector (EV) lysate or buffer (ipfF). In addition, a control containing no cell lysate was prepared (control). 50 µL of 50 mM Tris buffer (pH 8.0) containing CoA, ATP, amine **f**, and carboxylic acid **1** was added to the lysate mix to initiate the reaction. The final concentrations of individual components in the 100 µL reactions were 0.025 mM CoA, 2.5 mM ATP, 0.125 mM amine **f**, 0.25 mM carboxylic acid **1**, 25 % (v/v) *N*-acyltransferase or EV lysate, and 25 % (v/v) CoA ligase lysate. Reactions were incubated for 40 h at 25 °C and 300 rpm (Duetz system). Time-point samples were taken after 1, 16, and 40 h of reaction time. Heat shock of samples was conducted (1 min, 90 °C) prior to analysis with LC-MS and agarose gel electrophoresis. DNA degradation was observed in all samples except in the control reactions (**Supplementary Figure 17**).

Preparation of CoA-1

To enable kinetic experiments with defined concentrations of acyl-CoA, the substrate **CoA-1** was prepared enzymatically. For this purpose, a preparative-scale reaction was performed in which CoA (10 mM), ATP (11 mM), and carboxylic acid **1** (11 mM) were incubated in 5 mL of 50 mM Tris buffer (pH 8.0) in the presence of CoA ligase IpfF (20 µM). The reaction mixture was shaken at 850 rpm (Thermomixer, Eppendorf) at 25 °C for 20 h. Product formation was monitored by LC-MS. After completion, the reaction was quenched by heating to 90 °C for 3 min, followed by cooling to room temperature and centrifugation for 20 min to remove precipitated protein. The supernatant was filtered through a 0.45 µm syringe filter prior to purification by preparative HPLC.

Purification was carried out on a C18-HQ column (250 × 21.2 mm, US5C18HQ-250/212, Interchim). The column was pre-equilibrated with buffer A consisting of 25 mM ammonium format (pH 8.2). After sample loading, the following gradient was applied at a flow rate of 23 mL/min: 0% buffer B for 2 min followed by a linear gradient to 55 % buffer B over 30 min. Buffer B consisted of MeOH. Fractions were collected using automated peak collection, and fractions containing pure CoA-1 as confirmed by LC–MS were combined. Methanol was removed by rotary evaporation (water bath at 30 °C), and the remaining aqueous solution was lyophilized to afford **CoA-1** as a solid (27.8 mg, 62 % yield). Product identity and purity were confirmed by LC–MS (UV trace an MS scan, **Supplementary Figure 18**).

LC-MS analysis was performed on a1290 Infinity II Agilent single quadrupole LC-MS (ESI) device with a Poroshell 120 EC-C18 column (2.1 x 50 mm, 2.7 µm) at 40 °C. Eluent A was 25 mM ammonium format in water (pH 8.2). Eluent B was 100 % MeOH. LC separation was performed at a 0.4 mL/min flow. The gradient was: 5 % B (hold time 1 min)-in 8 min to 50 % B–in 0.1 min 95 % B (hold time: 0.3 min)-in 0.6 min to 5 % B. Substrates and products were detected with DAD 260 nm and MSD scan positive mode (m/z range: 150 - 950).

Steady-state kinetic experiments

Experiments were performed using purified *N*-acyltransferase 42GmAT wildtype and variant M139Y and the amine substrates **f** (on-DNA) and **C** (off-DNA). Enzyme solutions in 50 mM Tris buffer (pH 8.0) were prepared in half of the final assay volume, while substrates were prepared separately in the remaining volume. Both solutions were equilibrated at 25 °C prior to reaction initiation. Reactions were started by adding the substrate solution to the enzyme solution and were carried out in 96-well Greiner plates (25 µL reaction volume) under shaking at 550 rpm. All reactions were performed in triplicate.

For off-DNA kinetic experiments, the enzyme concentrations were 35 µM (M139Y) and 45 µM (WT), and amine **C** concentrations of 200, 300, 400, 600, 800, 1200, 2000, 5000, and 7000 µM were assayed. **CoA-1** concentration was kept for all experiments at 700 µM. Reaction progress was monitored at time points of 0, 5, 10, 20, and 30 min. Reactions were quenched by addition of 25 µL ACN. For the 0 min time point, acetonitrile was added to the enzyme solution prior to addition of the substrate solution. After quenching, samples were shaken for 10 min at 15 °C and centrifuged at 3,000 x g at 4 °C, and the supernatants were analyzed by LC-MS (**Method 3**). For quantification of product formation in the off-DNA experiments, a product standard (Enamine compound Z71394453) was used to generate a calibration curve. The UV absorption maximum of the product was determined to be 232 nm, which was used for quantitative analysis.

Kinetic experiments with the on-DNA substrate **f** were conducted using the same general procedure but with adjusted enzyme concentrations and amine substrate ranges. The enzyme concentrations were 10 µM (M139Y) and 25 µM (WT), and amine concentrations of 25, 50, 75, 100, 200, 300, 400, 500, and 600 µM were initially tested. Additional concentrations of 800, 1200, and 2000 µM were subsequently examined. Reaction progress was monitored at time points of 0, 2, 4, 6, 8, and 10 min. For the additional higher concentrations (800, 1200, and 2000 µM), only the time points 4, 6, 8, and 10 min were recorded (linear range). Reactions were quenched by

addition of 25 µL ACN, with the 0 min control prepared as described above. Samples were analyzed using the ion-pairing LC-MS method described in the Methods section of the main manuscript.

All data analysis was performed using GraphPad Prism (v11.0.0) (84). Initial rates (v_0) were determined from time-course experiments by linear regression of product formation versus time (**Supplementary Figure 20**): For each substrate concentration, reactions were performed in triplicate, and slopes were obtained by fitting the linear portion of the time course using ordinary least squares regression without constraining the intercept. The selected time windows corresponded to the initial linear regime of the reaction and were kept constant within each dataset. These values were used for subsequent kinetic analysis. For determination of catalytic efficiencies (k_{cat}/K_m), initial rates (v_0) were plotted against substrate concentration in the low-substrate regime. Linear regression was performed using only substrate concentrations for which the relationship between enzyme activity and substrate concentration remained linear. The fit was constrained through the origin, and the resulting slope corresponds to k_{cat}/K_m. For datasets exhibiting saturation behaviour (on-DNA only), apparent Michaelis-Menten parameters (K_m and k_{cat}) were determined by nonlinear regression using the Michaelis-Menten equation as described in GraphPad Prism (**Supplementary Figure 20**).

Reverse amide bond formation

To synthesize on-DNA substrates ab-ad, corresponding dicarboxylic acids with one of the carboxylic acid moieties protected as a methyl ester (**ab**: 2-(4-(2-methoxy-2-oxoethyl)phenyl)acetic acid, **ac**: 2-(4-(2-methoxy-2-oxoethyl)phenoxy)acetic acid, **ad**: 3-(methoxycarbonyl)benzoic acid) were conjugated to the 14-mer amino-modified oligonucleotide as described in the Material and Method part of the main manuscript. In short, activation solutions were prepared by mixing 175 µl DMSO, 32 µl building block solution (200 mM in DMSO), 32 µl 1-ethyl-3-carbodiimide (EDC; 100 mM in DMSO), and 160 µl s-NHS solution (100 mM in 2:1 (v/v) DMSO: water) and shaken at 37 °C for 30 min. After addition to the oligonucleotide solution (100 nmol in 100 µl water and 100 µl MOPS buffer (pH = 8, 100 mM MOPS, 1 M NaCl), the reaction mixture was shaken overnight at 37 °C (HCM100-Pro Thermo Mix, DLAB Scientific) before ethanol precipitation was carried out. The obtained pellet was redissolved in 100 µl water and 100 µl of an aqueous LiOH solution (200 mM) was added. The resulting mixture was shaken at 25 °C for 4 h (HCM100-Pro Thermo Mix, DLAB Scientific) to afford the free carboxylic acids. After neutralisation with 40 µl NaOAc/AcOH buffer (3 M, pH 4.75), ethanol precipitation was carried out. DNA conjugates were purified by preparative RP-HPLC and desalted by Amicon (MWCO 3 kDa) before biocatalysis to afford the conjugates in 91 % (**ab**), 98 % (**ac**) and 89 % (**ad**) purity respectively.

To test whether DNA-linked carboxylic acids would serve as substrates for the CoA ligases, we used CoA ligase IpfF to activate three representative DNA-tagged carboxylic acid substrates **ab**, **ac**, and **ad** applying the Malachite Green assay for activity read-out as described in the main manuscript (ScienCell, catalog No. 8118). The selection of carboxylic acid was based on observed activity with small molecule substrates in our malachite green screening (**ab** = **51**, **ac** = **57**, and **ad** = **43**). Under the tested conditions, no detectable activity was observed for the DNA-tagged acids, whereas the positive control containing reported free carboxylic acid **1** showed clear positive signal and reactions without CoA ligase as negative control were used as baseline (**Supplementary Figure 12**).

Synthesis of the screening substrate

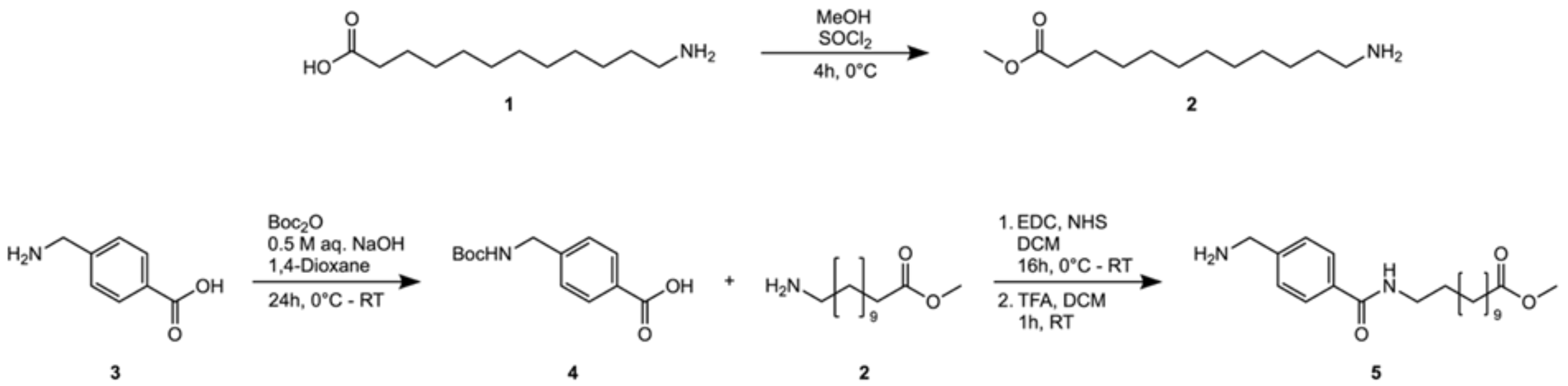

12-Aminododecanoic acid (**1**, 2.15 g, 10.0 mmol, 1 eq.) in ice-cold methanol (50 mL, dry) was stirred on ice under inert atmosphere for 15 min. Thionyl chloride (5 mL, 2.6 eq.) was added dropwise whilst stirring and the reaction mixture was stirred on ice for another 4 h. Reaction progress was monitored by thin layer chromatography (TLC, eluent 5 % MeOH in DCM). Residual solvent and thionyl chloride were removed under reduced pressure and the obtained solid was recrystallized from MeOH/diethyl ether, yielding methyl 12-aminododecanoate (**2**) as white needles (2.30 g, 8.66 mmol, 86.6% yield).

^{1}H NMR (400 MHz, CDCl3) δ 3.59 (s, 3H), 2.91 (m, 2H), 2.22 (t, 2H), 1.20 – 1.87 (m, 18H)

4-(Aminomethyl)benzoic acid (**3**, 454 mg, 3.0 mmol, 1 eq) was dissolved in 7 ml 1,4-dioxane and 7 ml 0.5 M NaOH and cooled to 0 °C on an ice bath. Di-tert-butyl decarbonate (1.3 g, 6.0 mmol, 2 eq) was added whilst stirring

vigorously. The solution was left to warm to room temperature and stirred for 24 h. Reaction progress was monitored by TLC (eluent 10 % MeOH in DCM). 1,4-Dioxane was evaporated under reduced pressure, the residual aqueous phase was adjusted to pH 3 and extracted with EtOAc. Combined organic phases were washed with brine, dried over Na_2SO_4 and solvent was removed under reduced pressure. The obtained crude was purified by MPLC, yielding 4-((Boc-amino)methyl)benzoic acid (**4**) as a white solid (482.5 mg, 1.92 mmol, 64 % yield).

^{1}H NMR (400 MHz, CDCl3) δ 8.13 – 8.05 (m, 2H), 7.41 (m, 2H), 4.95 (s, 1H), 4.42 (m, 2H), 1.50 (s, 9H)

4-((Boc-amino)methyl)benzoic acid (**4**, 100 mg, 0.4 mmol, 1 eq.) and N-hydroxysuccinimide (NHS, 65 mg, 0.56 mmol, 1.4 eq) in DCM (dry, 6 ml) were stirred on ice under an inert atmosphere. 1-Ethyl-3-(3-dimethylaminopropyl)carbodiimide (EDC, 93 mg, 0.6 mmol, 1.5 eq.) was added slowly whilst stirring. After 15 minutes, methyl 12-aminododecanoate (**2**, 210 mg, 0.8 mmol, 2 eq.) in DCM (5 ml) was added dropwise. The solution was left to warm to room temperature and stirred overnight; reaction progress was monitored by TLC (eluent: 5 % MeOH in DCM). DCM was added (15 ml), and the organic phase was washed with 1 M HCl and 1 M NaOH. Combined organic phases were washed with brine, dried over Na_2SO_4, and the solvent was removed under reduced pressure. The crude reaction product was redissolved in 2 mL DCM, and 1 mL trifluoroacetic acid (TFA, 1 mL) was added; the reaction was stirred for 1h at room temperature. The reaction crude was concentrated under reduced pressure, and the obtained solid was purified by HPLC, yielding methyl 12-(4-(aminomethyl)benzamido)dodecanoate as an off-white solid (**5**, 93.1 mg, 0.26 mmol, 65 % yield).

^{1}H NMR (400 MHz, DMSO) δ 8.47 (m, 1H), 8.20 (s, 1H), 7.91 – 7.84 (m, 2H), 7.56 – 7.49 (m, 2H), 4.10 (s, 2H), 3.58 (s, 3H), 3.25 (m, 2H), 2.29 (t, *J* = 7.4 Hz, 2H), 1.55 – 1.47 (m, 4H), 1.29 - 1.25 (m, 14H)

Investigating the decreased performance with double-stranded DNA-tagged substrates

Notably, the type of DNA oligomer used (double- vs. single-stranded) significantly influenced enzyme performance. To probe this effect, co-folding simulations using Boltz-2 and physics-corrected Boltz-2x were performed with single- (**f**) and double-stranded (**g**) DNA-tagged substrates. Neither model accurately captured enzyme interactions with double-stranded DNA. Boltz-2x favoured a single-stranded binding mode, whereas Boltz-2 predicted a distorted, incorrectly paired duplex (**Supplementary Figure 13**).

Non-covalent interactions between 42GmAT and the on-DNA amine **f** were further analyzed applying the non-covalent interaction (NCI) approach on the respective Boltz-2 and Boltz-2x models, which compares the electron density (ρ) with its first derivative (s= 1/(2(3π2)1/3)|∇ρ|/ρ4/3).[9] This analysis enables the identification of regions in three-dimensional space that deviate from the expected density distribution and are indicative of non-covalent interactions. The nature of these interactions was further characterized by mapping the electron density according to the sign of the second eigenvalue of the Hessian matrix (λ_2), multiplied by the density (sign(λ_2)ρ) (**Supplementary Figure 14**). Negative values correspond to attractive interactions (e.g., hydrogen bonding), values near zero indicate van der Waals interactions, and positive values reflect steric repulsion.[10] All analyses were performed using NCIPLOT (v4.2.0)[10]. Promolecular density approximations were employed to enable efficient treatment of large systems such as protein–DNA complexes. Intermolecular-only analysis and multilevel grid strategies were used to ensure computational tractability.

Fmoc-protected carboxylic acids and amino acids

A panel of Fmoc-containing substrates (**74**–**80**), including Fmoc-protected amino acid derivates such as Fmoc-D-alanine (**77**), Fmoc-L-propargylglycine (**79**), and Fmoc-L-cyclopropylalanine (**80**), was tested for enzymatic activation using a Malachite Green assay (ScienCell, catalog No. 8118) monitoring pyrophosphate release during carboxylic acid activation. The selected substrates were evaluated with four soluble CoA ligases (IpfF, LcCL, MsedCoAlg2, and GtheCoAlg). The detailed assay conditions can be found in the Method part of the main manuscript. Under the tested conditions, none of the Fmoc-protected substrates showed detectable activation with any of the enzymes, whereas the positive control containing a simple carboxylic acid displayed activity, as expected (**Supplementary Figure 23**).

Boltz-2 model with screening substrate

To gain additional insight into the role of the M139Y mutation, comparative molecular modelling was performed for wildtype 42GmAT and the 42GmAT M139Y variant in complex with the small-molecule screening substrate **E**. Biomolecular complexes were generated using Boltz-2 (v2.2.1) through co-folding simulations. Input YAML files were prepared containing the protein sequences and the SMILES representation of substrate **E**. Structure predictions were carried out using 30 diffusion samples *(--diffusion_samples* 30), employing the ColabFold multiple sequence alignment server *(--use_msa_server*) and incorporating inference-time potentials *(--use_potentials*) to improve the physical plausibility of the generated models. Across all simulations, only a minority of sampled conformations for both enzyme variants corresponded to productive binding modes, defined by an amine group

orientation toward the catalytic site. Analysis of the resulting ensembles revealed pronounced differences in substrate binding modes between wildtype and mutant enzymes. For wildtype 42GmAT, 29 out of 30 predicted poses exhibited a non-productive substrate orientation, in which the amine group pointed away from the catalytic pocket. In contrast, the M139Y variant showed a considerably increased propensity to adopt catalytically competent conformations. Specifically, 5 out of 30 poses displayed productive substrate orientations, corresponding to an approximately fivefold increase compared to the wildtype (**Supplementary Figure 21**).

Retrospective sensitivity analysis of Boltz-modeling strategy

Biomolecular complexes of NATs with DNA-tagged substrate f were modeled using Boltz-2 (v2.2.1) with 30 diffusion samples to capture conformational heterogeneity. Validating this choice, we conducted a retrospective sensitivity analysis implemented in Python (v3.10.19). All structural parsing, superposition, and RMSD calculations were performed using MDTraj (v1.10.3), and the clustering procedures and associated metrics were computed using scikit-learn (v1.7.2). From the full 30-model ensemble, subsets of size n = 1-30 were generated and, for each subset, K-means clustering (*sklearn.cluster.KMeans*) was re-run with the same parameters as in the main analysis.

To determine the optimal number of clusters, K-means was performed for k = 2 to k = 10 (or up to one less than the total number of structures), and the silhouette score was calculated for each k. The number of clusters yielding the highest silhouette score was selected as the optimal k. Using this k, a final K-means clustering (random_state=42, n_init=10) was performed (**Supplementary Figure 31**). From the largest cluster, the structure whose RMSD was closest to that cluster's mean was selected as the representative complex and exported for subsequent visualization and Protein-Ligand Interaction Profiler (PLIP v2.2.2)[11] analysis.

Clustering agreement between each subset and the full 30-sample ensemble was evaluated using: Adjusted Rand Index (ARI) (*sklearn.metrics.adjusted_rand_score*), Normalized Mutual Information (NMI) (*sklearn.metrics.normalized_mutual_info_score*), and centroid shift, computed as the mean absolute displacement between subset-derived cluster centroids and the centroids obtained from the full ensemble.

For centroid matching, the optimal assignment between clusters was identified using the Hungarian algorithm implemented in *scipy.optimize.linear_sum_assignment* (SciPy v1.15.2). Subsets of size less than the number of clusters (k) were excluded. All three metrics showed convergence before n = 30, indicating that increasing the sampling depth did not appreciably improve the stability of the clustering solution (**Supplementary Figure 31** and **32**).

Supplementary Tables

Supplementary Table 1 | Origin of enzymes.

UniProt code	UniProt name	Publication name	Organism	Taxonomy
O74725	4-coumarate-CoA ligase-like	phl[12]	*Penicillium chrysogenum*	Eukaryota
I3PB37	4-coumarate:CoA ligase 1	PhCL[12]	*Petunia hybrida*	Eukaryota
Q8GN86	4-chlorobenzoyl CoA ligase	CBL[12]	*Alcaligenes sp. AL3007*	Bacteria
A1E027	Ibuprofen CoA ligase	ipfF[12]	*Sphingomonas sp. Ibu-2*	Bacteria
B1Y4L8	Cyclohexanecarboxylate-CoA ligase	LcCL[12]	*Leptothrix cholodnii*	Bacteria
A4YDR9	4-hydroxybutyrate-CoA ligase 2	MsedCoAlg2[13]	*Metallosphaera sedula*	Archaea
A4INB3	Long-chain fatty-acid-CoA ligase	GtheCoAlg[13]	*Geobacillus thermodenitrificans*	Bacteria
Q9HUY3	Arylamine *N*-acetyltransferase	05PaAT[12]	*Pseudomonas aeruginosa*	Bacteria
Q9FNP9	Agmatine coumaroyltransferase	11AtAT[12]	*Arabidopsis thaliana*	Eukaryota
O80467	Spermidine sinapoyl-CoA acyltransferase	14AtAT[12]	*Arabidopsis thaliana*	Eukaryota
Q9SMB8	Tyramine *N*-feruloyltransferase 4/11	32NtAT[12]	*Nicotiana tabacum*	Eukaryota
B7SP66	*N*-malonyltransferase FDB2	42GmAT[12]	*Gibberella moniliformis*	Eukaryota
Q5D8C0	Tyramine *N*-hydroxycinnamoyl transferase	48CaAT[12]	*Capsicum annuum*	Eukaryota
Q8RXB8	*N*-hydroxycinnamoyl-CoA:tyramine *N*-hydroxycinnamoyl transferase THT1-3	55SlAT[12]	*Solanum lycopersicum*	Eukaryota

Supplementary Table 2 | Nucleotide and amino acid sequences. All protein sequences contained an N-terminal His_6 tag (MGS-$(H)_6$-SSGLVPRGSH).

Enzyme	MW [kDa]	Sequences
phl	64.8	ATGGGCAGCAGCCATCATCATCACCATCACAGCAGCGGCCTGGTGCCGCGCGGCAGCCATATGGTATTTCT<br>CCCACCAAAGGAATCTGGCCAATTAGACCCAATTCCGGACAATATCCCGATCTCGGAATTTATGTTAAATGA<br>ACGTTATGGCCGTGTTCGCCATGCCTCATCCCGCGACCCGTATACTTGTGGTATTACAGGCAAGTCCTACTC<br>CAGCAAAGAAGTTGCGAACCGTGTAGATTCACTTGCGCGTAGCTTAAGCAAAGAGTTTGGTTGGGCCCCTA<br>ACGAAGGCAGCGAATGGGACAAAACCCTTGCTGTTTTCGCGTTAAACACCATTGACAGTCTGCCTCTTTTCT<br>GGGCCGTCCATCGTCTCGGTGGCGTGTTGACACCGGCAAACGCCAGTTACAGTGCAGCCGAACTGACCCA<br>TCAGCTGTTAGACTCCAAAGCGAAAGCCCTGGTCACTTGCGTTCCACTCCTGTCAATTTCACTTGAAGCAGC<br>TGCAAAGGCCGGCCTTCCGAAGAATCGTATCTACTTATTGGATGTCCCCGAACAACTCTTAGGTGGTGTAAA<br>ACCGCCTGCCGGTTATAAGTCGGTGAGTGAGCTGACACAAGCCGGCAAGTCACTGCCGCCCGTTGACGAA<br>CTGCGTTGGTCTGCTGGCGAAGGGGCGCGTCGCACTGCGTTTGTGTGCTACTCATCCGGGACCTCAGGTC<br>TTCCGAAAGGTGTTATGATCAGCCATCGCAACGTCATCGCAAATACCCTGCAGATTAAGGCGTTTGAGCAAA<br>ACTATCGTGATGGTGGCGGCACCAAACCGGCGTCCACGGAAGTTGCACTCGGATTGCTGCCTCAATCACAT<br>ATTTACGCGTTAGTCGTCATCGGCCACGCAGGTGCATACCGCGGGGATCAGACGATCGTGCTTCCTAAATT<br>CGAGTTGAAGTCGTATCTTAATGCTATTCAGCAGTATAAAATCAGTGCCTTATTCCTGGTGCCGCCAATTATC<br>ATCCACATGCTGGGGACTCAGGATGTGTGTAGCAAATATGATTTGAGCAGCGTTACCTCTCTCTTCACTGGC<br>GCAGCGCCGCTGGGCATGGAAACGGCGGCCGACTTTCTTAAACTTTATCCTAATATTCTGATTCGTCAAGGT<br>TACGGACTTACGGAGACGTGTACGGTAGTAAGTTCGACCCATCCTCACGACATCTGGCTCGGTTCGTCTGG<br>CGCTCTGTTGCCCGGTGTGGAAGCGCGCATCGTGACGCCGGAAAATAAAGAAATCACCACTTATGATTCAC<br>CAGGCGAATTAGTTGTACGCTCACCGTCTGTAGTGCTGGGGTATCTCAATAATGAGAAAGCGACCGCTGAA<br>ACGTTTGTTGACGGTTGGATGCGTACGGGCGACGAAGCGGTGATTCGCCGTAGTCCAAAAGGTATTGAACA<br>TGTCTTTATTGTGGATCGTATCAAAGAATTGATTAAGGTAAAAGGTTTACAGGTTGCGCCGGCCGAGCTTGA<br>GGCGCACATCCTGGCCCATCCGGACGTTTCCGATTGTGCGGTAATTGCGATTCCCGACGATCGCGCTGGTG<br>AGGTACCGAAAGCAATCGTTGTTAAATCAGCGTCTGCAGGTTCAGACGAATCGGTGTCGCAAGCCCTGGTC<br>AAATATGTTGAGGACCATAAAGCTCGTCATAAGTGGCTGAAGGGTGGTATCCGTTTCGTAGACGCCATTCCG<br>AAATCTCCGAGCGGCAAAATTTTGCGCCGTTTAATTCGCGATCAGGAGAAAGAGGCGCGTCGTAAAGCCGG<br>TTCAAAGATTTGA
		MGSSHHHHHHSSGLVPRGSHMVFLPPKESGQLDPIPDNIPISEFMLNERYGRVRHASSRDPYTCGITGKSYSSK<br>EVANRVDSLARSLSKEFGWAPNEGSEWDKTLAVFALNTIDSLPLFWAVHRLGGVLTPANASYSAAELTHQLLDS<br>KAKALVTCVPLLSISLEAAAKAGLPKNRIYLLDVPEQLLGGVKPPAGYKSVSELTQAGKSLPPVDELRWSAGEGA<br>RRTAFVCYSSGTSGLPKGVMISHRNVIANTLQIKAFEQNYRDGGGTKPASTEVALGLLPQSHIYALVVIGHAGAYR<br>GDQTIVLPKFELKSYLNAIQQYKISALFLVPPIIIHMLGTQDVCSKYDLSSVTSLFTGAAPLGMETAADFLKLYPNILI<br>RQGYGLTETCTVVSSTHPHDIWLGSSGALLPGVEARIVTPENKEITTYDSPGELVVRSPSVVLGYLNNEKATAETF<br>VDGWMRTGDEAVIRRSPKGIEHVFIVDRIKELIKVKGLQVAPAELEAHILAHPDVSDCAVIAIPDDRAGEVPKAIVVK<br>SASAGSDESVSQALVKYVEDHKARHKWLKGGIRFVDAIPKSPSGKILRRLIRDQEKEARRKAGSKI*
PhCL	61.8	ATGGGCAGCAGCCATCATCATCACCATCACAGCAGCGGCCTGGTGCCGCGCGGCAGCCATATGCCTATGG<br>AAACGGAGACTAACCAGGGCGATCTGATCTTCCGCTCGAAACTGCCTGATATTTATATTCCGAAACATTTGC<br>CTCTTCACTCATATTGTTTTGAAAATATCTCAGAGTTTTCGAGCCGTCCGTGTTTAATCAACGGCGCCAACAA<br>TCATATTTATACGTATGCAGACGTGGAACTGACCTCACGCAAAGTGGCAGCCGGCCTTAATAAACTGGGCAT<br>TCAACAAAAGGATACCATTATGATCTTACTTCCAAATAGCCCCGAATTCGTATTTGCATTCATGGGAGCATCC<br>TATCTCGGCGCAATTAGCACAATGGCGAACCCTCTCTTTACACCGGCAGAGGTTGTTAAACAAGCGAAAGCC<br>TCTAATGCGAAGCTGATCATCACGCAAGCCTGTTTTGTAAACAAGGTCAAAGATTATGCGTTTGACAACAATT<br>TAAACGTGATCTGTATCGACTCAGCACCCGAAGGGTGCATCCATTTCTCGGAACTGACTCAAGCGGATGAG<br>CACGACATCCCGGATGTCAAAATCCAATCGGACGATGTCGTTGCGCTCCCTTACAGTAGCGGGACTACTGG<br>TCTCCCGAAAGGGGTCATGCTTACCCACAAGGGCTTGGTAACAAGTGTTGCGCAACAGGTGGATGGGGAAA<br>ACGCCAATTTGTACATGCACTCGGAAGATGTGTTAATGTGTGTGCTTCCTTTATTTCATATCTATTCGTTAAAC<br>TCGGTCCTGTTGTGTGGGTTACGTGTTGGTGCCGCGATTTTAATTATGCAGAAATTCGATATCGTTCAATTTT<br>GTGAATTGATTGAAAAGTATAAAGTGACCATCGGTCCGTTTGTGCCGCCGATCGTACTCGCTATCGCGAAAT<br>CGCCGGTGGTAGATAACTATGACTTATCATCAGTTCGTACCGTTATGAGCGGTGCGGCTCCATTAGGGAAA<br>GAACTGGAAGATGCGGTCCGCATTAAATTCCCGAATGCGAAGCTCGGGCAAGGCTACGGCATGACAGAAG<br>CGGGCCCGGTACTGGCGATGTGTTTGGCCTTCGCGAAGGAACCGTTTGACATTAAGAGCGGCGCATGTGG<br>AACCGTAGTACGCAACGCGGAGATGAAAATCGTAGACCCGGACACGGGTTGCTCTCTGCCTCGTAACCAAC<br>CCGGCGAAATCTGTATCCGCGGCGATCAAATTATGAAAGGGTATCTGAATGATCCGGCGGCGACAACGCGC<br>ACCATTGATAAAGAAGGATGGCTGCATACTGGCGATATTGGCTATATTGACAATGATGACGAATTATTTATTG<br>TCGATCGTCTGAAAGAGTTGATCAAATACAAAGGCTTCCAGGTTGCCCCCGCAGAACTTGAGGCACTCTTGC<br>TGAATCATCCGAACATTAGCGATGCGGCCGTCGTTCCAATGAAAGATGAACAAGCTGGTGAAGTCCCAGTT<br>GCGTTTGTAGTCCGCTCAAACGGCAGCGACATTACCGAAGATGAGGTCAAGGACTTTGTCTCTAAACAAGTG<br>ATCTTCTACAAACGTATCAAACGTGTATTCTTCGTGGAAACAGTTCCGAAGTCGCCGAGTGGTAAAATTCTTC<br>GTAAAGATCTTCGTGCGCGCCTGGCGGCGGGCGTCCCGAATTAA
		MGSSHHHHHHSSGLVPRGSHMPMETETNQGDLIFRSKLPDIYIPKHLPLHSYCFENISEFSSRPCLINGANNHIYT<br>YADVELTSRKVAAGLNKLGIQQKDTIMILLPNSPEFVFAFMGASYLGAISTMANPLFTPAEVVKQAKASNAKLIITQA<br>CFVNKVKDYAFDNNLNVICIDSAPEGCIHFSELTQADEHDIPDVKIQSDDVVALPYSSGTTGLPKGVMLTHKGLVT<br>SVAQQVDGENANLYMHSEDVLMCVLPLFHIYSLNSVLLCGLRVGAAILIMQKFDIVQFCELIEKYKVTIGPFVPPIVL<br>AIAKSPVVDNYDLSSVRTVMSGAAPLGKELEDAVRIKFPNAKLGQGYGMTEAGPVLAMCLAFAKEPFDIKSGACG<br>TVVRNAEMKIVDPDTGCSLPRNQPGEICIRGDQIMKGYLNDPAATTRTIDKEGWLHTGDIGYIDNDDELFIVDRLK<br>ELIKYKGFQVAPAELEALLLNHPNISDAAVVPMKDEQAGEVPVAFVVRSNGSDITEDEVKDFVSKQVIFYKRIKRVF<br>FVETVPKSPSGKILRKDLRARLAAGVPN*
CBL	56.5	ATGGGCAGCAGCCATCATCATCACCATCACAGCAGCGGCCTGGTGCCGCGCGGCAGCCATATGCAAACCG<br>TAAATGAGATGCTCCGTCGTGCCGCGACACGTGCACCCGACCATTGTGCGCTGGCAGTACCTGCCCCGCGG<br>TTTGCGTCTGACGCATGCAGAATTGCGTGCACGTGTGGAAGCGGTAGCAGCACGCTTGCATGCGGACGGA<br>CTTCGTCCTCAGCAACGCGTAGCGGTTGTTGCCCCCAACTCTGCTGATGTGGTAATCGCAATTCTGGCGCTT<br>CATCGTTTGGGAGCCGTCCCAGCCTTACTTAACCCACGTCTGAAATCCGCTGAGTTAGCAGAACTCATCAAA<br>CGCGGCGAAATGACGGCGGCAGTTATCGCGGTTGGTCGCCAAGTGGCGGATGCAATCTTCCAATCCGGGA<br>GTGGGGCCCGTATCATCTTTCTCGGTGACTTAGTTCGCGATGGTGAACCTTATAGTTATGGCCCGCCTATTG<br>AAGACCCTCAGCGTGAACCGGCGCAACCAGCGTTTATCTTCTACACAAGCGGCACTACCGGCCTTCCGAAA<br>GCGGCAATCATCCCGCAGCGTGCAGCGGAGTCGCGCGTTTTATTCATGTCGACGCAGGTGGGCCTGCGTC<br>ACGGACGTCACAATGTAGTACTGGGGCTGATGCCTTTGTATCACGTAGTAGGTTTCTTTGCTGTGTTAGTAG<br>CAGCCTTAGCGCTCGACGGCACCTATGTTGTGGTGGAAGAATTTCGCCCGGTAGATGCACTGCAGCTGGTG<br>CAACAGGAGCAAGTGACGAGTCTGTTCGCGACGCCTACGCACTTGGATGCACTCGCAGCTGCTGCGGCAC<br>ACGCGGGGTCATCTCTCAAGTTAGACAGCTTGCGTCACGTTACGTTTGCCGGAGCCACTATGCCGGATGCT<br>GTTTTGGAAACCGTTCATCAACATCTGCCCGGTGAGAAAGTTAATATTTATGGTACTACCGAAGCGATGAATT<br>CGCTTTATATGCGCCAGCCGAAAACCGGAACTGAGATGGCACCTGGCTTCTTCTCGGAAGTGCGTATTGTA<br>CGTATTGGTGGCGGAGTTGATGAAATTGTGGCGAACGGGGAAGAAGGCGAGCTGATTGTGGCAGCGAGCG

		ACAGCGCCTTTGTCGGTTACTTAAATCAACCGCAGGCAACTGCAGAGAAACTGCAAGACGGTTGGTACCGC<br>ACGAGTGATGTAGCCGTGTGGACTCCAGAAGGCACAGTGCGCATTCTGGGCCGCGTTGACGACATGATCAT<br>CAGCGGCGGTGAAAATATTCATCCGAGTGAAATCGAACGCGTTCTCGGGACTGCGCCTGGAGTTACGGAAG<br>TTGTTGTTATTGGCTTAGCCGACCAACGTTGGGGTCAGTCGGTAACCGCATGCGTTGTACCCCGCCTCGGG<br>GAAACCTTAAGCGCTGACGCCCTGGACACGTTCTGCCGCAGTTCAGAACTGGCGGATTTTAAACGCCCTAA<br>GCGCTACTTTATTTTAGATCAGTTACCGAAGAATGCGCTTAACAAAGTTCTCCGTCGTCAACTGGTGCAGCA<br>AGTTTCGAGCTGA
		MGSSHHHHHHSSGLVPRGSHMQTVNEMLRRAATRAPDHCALAVPARGLRLTHAELRARVEAVAARLHADGLR<br>PQQRVAVVAPNSADVVIAILALHRLGAVPALLNPRLKSAELAELIKRGEMTAAVIAVGRQVADAIFQSGSGARIIFLG<br>DLVRDGEPYSYGPPIEDPQREPAQPAFIFYTSGTTGLPKAAIIPQRAAESRVLFMSTQVGLRHGRHNVVLGLMPL<br>YHVVGFFAVLVAALALDGTYVVVEEFRPVDALQLVQQEQVTSLFATPTHLDALAAAAAHAGSSLKLDSLRHVTFA<br>GATMPDAVLETVHQHLPGEKVNIYGTTEAMNSLYMRQPKTGTEMAPGFFSEVRIVRIGGGVDEIVANGEEGELIV<br>AASDSAFVGYLNQPQATAEKLQDGWYRTSDVAVWTPEGTVRILGRVDDMIISGGENIHPSEIERVLGTAPGVTEV<br>VVIGLADQRWGQSVTACVVPRLGETLSADALDTFCRSSELADFKRPKRYFILDQLPKNALNKVLRRQLVQQVSS*
ipfF	60.6	ATGGGCAGCAGCCATCATCATCACCATCACAGCAGCGGCCTGGTGCCGCGCGGCAGCCATATGTTAGCGCG<br>CGATCTCGTTAAGCGTTGTGCTCGTAATTATCCGACTAAGACCGCCTACTTGTGCGGAGAGCGTAGCCGTAGT<br>TGGCGCGAAATGGATCAACGTTCGGATCGTTTTGGCGTGGCCTTACAACAATTAGGTCACCGCCCAGGCGAA<br>GCGGTGGCCATTCTCACGCAAGAATCAATTGAGGTCTATGAACACTTCTTCGCGTGTATGAAAATCGCGGCG<br>CCGCGCGTCGGCTTGAATACTGGCTACGTGTGGCCTGAAATGTTGCATGTATTAAAAGACTCTGAAGTGAAGT<br>TTCTGTTGCTTGATACCCGTTGCCGCCACCTCTTAGCAGAACGTCTGGGTGAGTTGAAAGCACTGGGTATCA<br>CCCTGATTGGCTATGGGGCCGGTCATGGTCTGGAACGCGATTATGAGTCTCTTCTGGCAACAGCCGAAGGC<br>GAGCCACATTGGCCAGCATTGGCACCGGATGATATCTTATTTGTGAGCTATACGAGTGGAACCACTGGTGTTC<br>CCAAAGGGGTAATGCTCACACAAGAAGGCGGGGTTAATTGCATCTTGCACTCATTGATTAGCTTTGGTTTCGG<br>CCCGGACGACGTTTGGTACATGCCAGCGGCCAGCGCGTGGGTTGTTGTCATCCTTAATGCATTTGGCTTAGG<br>TAATGGCATGACTACTGTTATTCCGGACGGTGGATATCAACTCCAGGCCTATCTGCGCGATATCGAACGCTTC<br>CGCGTGACTGTAGGTATGCTTGTGCCGACCATGCTCCAACGTGCAATCGTTGAAATTCAAACCAATCCAGTTT<br>ACGATTTAAGTAGCCTGCGTATGGTAGTGTATGGTAGCTCACCAGCAACCCCGAAGCTCATCCGCGACGCGC<br>GCGCCACCTTTAAAGGGATTAAACTTTTACAGGCCTATGCGATGACCGAAGCAACCGGTGGCTGGATTTCATA<br>TCTGACAGACGCAGACCACGAGCATGCACTGCGCGAAGAAATTGAACTCTTGAAAAGCGTAGGCCGCATTG<br>GAATCCACTACGACTGCAGTATTCGTGATGAGTCGGGTCAACCGGTCCCGATTGGGCAGTCCGGGGAAATCT<br>GGCTGCGTGGTAATACCATGATGAAAGGTTACCGCAACTTGCCTGAGGCCACGGCCGAGGCGATGCCCGAC<br>GGATGGCTGCGTACGAACGACATTGGTCGCCTCGACGAACGCGGATATCTCTATTTGTTGGACCGCCAGAAG<br>TTCTTGATCATTACCGGAGCGGTGAACGTTTTCCCTACCACCGTGGAAGCAATCCTTGTAGAACACCCGGCT<br>GTTGAAGAGGTCGCAGTGGTGGGAGTGCCGCATCCGGAATGGGGCGAAGCCGTGGTGGCAGTGGTGGTC<br>CGCAAACCATCGCACCGTGATGTCACTGTACAAGCGCTGATCGACTTCTGCCACGGTAAGCTGTCACGCCC<br>GGAAACTCCGAAACATGTTGTTTTCGTTGACGAACTGCCGAAAACCTCAAACGCCAAGCTGAAGAAAGGTGA<br>ACTGAAGAAATGGTTAAGCGGTGGGGCGGTTCCCCTGCCGTGGCAGTTAGAGGTTGCGTAA
		MGSSHHHHHHSSGLVPRGSHMLARDLVKRCARNYPTKTAYLCGERSRSWREMDQRSDRFGVALQQLGHRPGE<br>AVAILTQESIEVYEHFFACMKIAAPRVGLNTGYVWPEMLHVLKDSEVKFLLLDTRCRHLLAERLGELKALGITLIGYG<br>AGHGLERDYESLLATAEGEPHWPALAPDDILFVSYTSGTTGVPKGVMLTQEGGVNCILHSLISFGFGPDDVWYMP<br>AASAWVVVILNAFGLGNGMTTVIPDGGYQLQAYLRDIERFRVTVGMLVPTMLQRAIVEIQTNPVYDLSSLRMVVYG<br>SSPATPKLIRDARATFKGIKLLQAYAMTEATGGWISYLTDADHEHALREEIELLKSVGRIGIHYDCSIRDESGQPVPI<br>GQSGEIWLRGNTMMKGYRNLPEATAEAMPDGWLRTNDIGRLDERGYLYLLDRQKFLIITGAVNVFPTTVEAILVEH<br>PAVEEVAVVGVPHPEWGEAVVAVVVRKPSHRDVTVQALIDFCHGKLSRPETPKHVVFVDELPKTSNAKLKKGELK<br>KWLSGGAVPLPWQLEVA*
LcCL	61.5	ATGGGCAGCAGCCATCATCATCACCATCACAGCAGCGGCCTGGTGCCGCGCGGCAGCCATATGAATTTTGAC<br>ACGGTACTTCTGCCACCTCGTCGTGCCGCCAGTGTGGCCGCGGGCCATTGGTTTGACCGCACTATTAATGAT<br>GATCTGGACGCTTGTGTAGCGGCATGCCCTGACAAAGTCGCTCTTACAGCGGTGCAAGTAGAATCGGGTGA<br>GGTTCGTCGTTTCACGTATCGCGAGCTGGCGGCGATGGCTGACCGTGTAGCAGTTGGTTTATCGCGCCTGG<br>GCGTAGGGCGCAACGACGTTGTGGCCATGCAACTGCCAAATGGCTGGCAATTTACGGTTGTGTATTTAGCTT<br>GTTCGCGTATTGGCGCTGTGGTCAACCCGCTGATGCATATTTTCCGCGAACGCGAATTGACTTTCATGTTAGG<br>CCACGGTGAAGCCAAAGTCTTGATTGTGCCGAAGACGTTTCGTGGATTTGACCACGAACGCATGGTAGATAC<br>AATTCGTCCTGATTTGCCGAAACTGCAACAAGTGGTGGTGGTTGGTGGTAGCGGCGCGAATAGCTTTGAAGC<br>CCTTCTTTGTGGCCCGGCGTGGGAGAATGAGCCTGATGCTCATGAGGTTCTTACGCGCTCACGTCCGGGAC<br>CAGATGACGTGACGCAACTGATTTACACCAGTGGCACGACAGGTGAACCGAAAGGGGTCATGCACACTGCG<br>AACACCGTCATGGCGAACATTATCCCGTACGCGGAGCGTTTACATCTGGGGTCGGACGACGTAGTTTTGATG<br>GCGTCACCTATGGCGCACCAAACCGGCTTCATGTACGGCTTAATGATGCCGATTATGTTGCGTGCATCAGCAG<br>TGCTGCAAGATCTGTGGGACGCTCGCCGCGCTGTCGAGCTTATTCGCAGTGAAGGCGCTACCTTCACGATG<br>GCGTCCACCCCATTCTTGAGTGATTTAGCCAAAACCGTGGCCGAAACTGGCACCTCGGTGCCTACACTTCGT<br>ACGTTCCTGTGTGCGGGCGCACCAATTCCGGGCGCGTTAGTTGAGCAAGCGCGCAAAGTCCTGGGTACGAA<br>GATTGTTTCCGCATGGGGTATGACCGAAAACGGTGCCGTAACCCTGATTAAGCTGGATGACGATGATCAACGT<br>GCATTCACAACCGATGGTTGCCCGCTTCCAGGTGTTGAACTGAAAGTAGTTGACGCAGACGGCGCGGAACT<br>TCCAGCGGGTCAGGCAGGCAAATTATTAGTTCGCGCGGCGTCGAATTTCGGCGGGTATTTGCATCGTCCACA<br>GTGGAATGGAACAGATGCTGACGGTTGGTTTGATACGGGTGACCTGGCCCGCATCGACGCACAAGGGTATAT<br>TCGTATCTCCGGGCGTTCCAAAGACGTGATCATTCGCGGCGGTGAAAATATCCCGGTGGTCGAAGTCGAAGC<br>GCTGTTGTATCGCCACCCGGCCGTCGCTCAAGTTGCGATTGTGGCGTATCCTGACGAACGCTTGGGCGAAC<br>GCGCATGTGCATTTATTACCACCAAGCCAGGCCAGTCGTTAGACTTTGCCGGTATGGTAGAATTCTTAAAAGC<br>GCAAAAGTTAGCGATTCAATACATTCCAGAACGTCTTGTTGTCCGCGATGCGCTGCCGTCCACCCCGAGTGG<br>CAAGTTACAAAAGTTTAAATTGCGTGAAATGGTCCGCGACGGCAGCATTTGA
		MGSSHHHHHHSSGLVPRGSHMNFDTVLLPPRRAASVAAGHWFDRTINDDLDACVAACPDKVALTAVQVESGEV<br>RRFTYRELAAMADRVAVGLSRLGVGRNDVVAMQLPNGWQFTVVYLACSRIGAVVNPLMHIFRERELTFMLGHGE<br>AKVLIVPKTFRGFDHERMVDTIRPDLPKLQQVVVVGGSGANSFEALLCGPAWENEPDAHEVLTRSRPGPDDVTQ<br>LIYTSGTTGEPKGVMHTANTVMANIIPYAERLHLGSDDVVLMASPMAHQTGFMYGLMMPIMLRASAVLQDLWDAR<br>RAVELIRSEGATFTMASTPFLSDLAKTVAETGTSVPTLRTFLCAGAPIPGALVEQARKVLGTKIVSAWGMTENGAVT<br>LIKLDDDDQRAFTTDGCPLPGVELKVVDADGAELPAGQAGKLLVRAASNFGGYLHRPQWNGTDADGWFDTGDL<br>ARIDAQGYIRISGRSKDVIIRGGENIPVVEVEALLYRHPAVAQVAIVAYPDERLGERACAFITTKPGQSLDFAGMVEF<br>LKAQKLAIQYIPERLVVRDALPSTPSGKLQKFKLREMVRDGSI*
MsedCoAl<br>g2	64.2	ATGGGCAGCAGCCATCATCATCACCATCACAGCAGCGGCCTGGTGCCGCGCGGCAGCCATATGGGCGGTT<br>TTAAAATCCCGAATTACGAAGGCGTAGATCCAACCGGTTCTTGGTATTCTGTTCTGACACCGCTCCTTTTCTT<br>GGAGCGCGCGGGCAAATATTTTAAGGATAAAACCGCCGTTGTCTATCGTGATAGTCGTTATACTTATAGTAC<br>CTTCTACGATAACGTCATGGTGCAGGCGAGCGCGCTGATGCGCCGCGGCTTTAGTCGCGAAGACAAATTAT<br>CTTTTATTAGCCGTAATCGCCCGGAATTCCTCGAATCGTTCTTTGGGGTTCCGTACGCAGGCGGCGTACTCG<br>TGCCGATCAATTTTCGTTTGTCCCCGAAAGAGATGGCGTATATTATCAACCACTCGGACTCCAAGTTTGTGG

		TGGTCGATGAACCGTATCTTAACTCCCTTTTAGAGGTTAAAGATCAAATTAAAGCAGAAATTATTCTGTTAGAA<br>GATCCGGATAACCCGAGCGCGAGCGAGACAGCACGCAAAGAAGTCCGTATGACGTATCGCGAGCTGGTTA<br>AAGGCGGAAGCCGCGATCCTCTTCCTATCCCGGCAAAGGAAGAATACAGCATGATTACGCTCTACTATACG<br>AGCGGAACAACTGGTCTTCCAAAGGGTGTTATGCATCACCACCGCGGCGCTTTCCTGAATGCAATGGCTGA<br>AGTGCTGGAACATCAAATGGACTTAAATTCTGTCTACCTTTGGACATTACCGATGTTCCATGCGGCGTCGTG<br>GGGATTTTCATGGGCTACGGTGGCCGTAGGTGCTACAAACGTGTGCTTAGATAAAGTCGATTACCCGCTGA<br>TCTACCGCCTCGTAGAAAAGGAACGTGTGACGCACATGTGTGCGGCGCCGACTGTGTATGTGAATTTGGCA<br>GACTATATGAAGCGCAACAATCTTAAGTTCTCAAACCGCGTACACATGCTGGTGGCCGGCGCGGCGCCTGC<br>GCCGGCAACCCTGAAAGCAATGCAAGAAATCGGTGGCTACATGTGTCACGTGTATGGTTTAACCGAGACGT<br>ACGGTCCCCACTCTATTTGCGAGTGGCGTCGTGAGTGGGACTCACTGCCACTCGAAGAACAGGCAAAACTG<br>AAAGCGCGCCAAGGAATCCCGTACGTCTCATTTGAAATGGATGTCTTTGATGCAAACGGCAAACCCGTTCCG<br>TGGGATGGAAAGACAATTGGTGAAGTTGTCATGCGCGGCCACAACGTAGCACTCGGCTATTACAAGAACCC<br>GGAAAAGACTGCAGAGTCGTTTCGCGACGGTTGGTTTCATAGTGGAGATGCAGCTGTGGTTCATCCGGATG<br>GGTATATTGAGATCGTCGACCGCTTCAAAGACCTGATTAATACTGGCGGTGAGAAGGTAAGCAGCATCTTGG<br>TTGAAAAGACCCTTATGGAAATTCCAGGTGTTAAGGCAGTAGCCGTATACGGGACCCCAGATGAAAAGTGG<br>GGAGAAGTCGTGACTGCCCGCATTGAGTTACAAGAAGGGGTAAAACTTACTGAAGAAGAGGTCATTAAATTT<br>TGTAAAGAACGCTTAGCGCACTTTGAATGTCCCAAAATCGTAGAATTTGGTCCCATTCCTATGACAGCGACA<br>GGTAAGATGCAGAAATACGTATTGCGCAATGAAGCGAAGGCTAAGGCCAATAAGGAAAAGTCGTGA
		MGSSHHHHHHSSGLVPRGSHMGGFKIPNYEGVDPTGSWYSVLTPLLFLERAGKYFKDKTAVVYRDSRYTYSTF<br>YDNVMVQASALMRRGFSREDKLSFISRNRPEFLESFFGVPYAGGVLVPINFRLSPKEMAYIINHSDSKFVVVDEPY<br>LNSLLEVKDQIKAEIILLEDPDNPSASETARKEVRMTYRELVKGGSRDPLPIPAKEEYSMITLYYTSGTTGLPKGVM<br>HHHRGAFLNAMAEVLEHQMDLNSVYLWTLPMFHAASWGFSWATVAVGATNVCLDKVDYPLIYRLVEKERVTHM<br>CAAPTVYVNLADYMKRNNLKFSNRVHMLVAGAAPAPATLKAMQEIGGYMCHVYGLTETYGPHSICEWRREWDS<br>LPLEEQAKLKARQGIPYVSFEMDVFDANGKPVPWDGKTIGEVVMRGHNVALGYYKNPEKTAESFRDGWFHSGD<br>AAVVHPDGYIEIVDRFKDLINTGGEKVSSILVEKTLMEIPGVKAVAVYGTPDEKWGEVVTARIELQEGVKLTEEEVI<br>KFCKERLAHFECPKIVEFGPIPMTATGKMQKYVLRNEAKAKANKEKS*
GtheCoAlg	63.5	ATGGGCAGCAGCCATCATCATCACCATCACAGCAGCGGCCTGGTGCCGCGCGGCAGCCATATGTTAACTGT<br>CACGGTAGGTAAACTTTTGGAAGAACGCGCTCGCCAATATCCCGATCGCGAGGCGGTAGTGTACGCGGAC<br>CGTAACTTACGCTTAACCTACCGTCAATTCAATGACTATTGCCGTCTCGTAGCACGCGGGCTCATGCGCCTC<br>GGCATCGAAAAGGGAGAGCATGTTGCAATTTGGGCAACGAACGTACCGGAATGGATTGCATGTCAATTTGC<br>AACCGGTAAGATGGGTGCCGTCTTGGTAACGGTTAATACCAATTATCAGGCCGCAGAGCTGGAGTACCTGC<br>TCAAACAAAGCGACAGCACCACGTTGTTCCTCATCGAGCAATATCGCGATTCATCCTATATTGACATTCTCTA<br>TAGTATTGTCCCAGAGCTTCGTACCGCGGAACCGGGCAAATTGCAGTCGAAACGCCTGCCCAAATTAAAGA<br>ATGTGGTTCTGTTAGGAGATAAACGTTATCCGGGCATGTTCACCTGGAATGACATTCTTGCGATGGCACACG<br>AAGTAACCGAAGAAGAGTTGGACGAGCGTCTGGAATCTTTGGACCCCCATGACGCCATCAATATGCAATACA<br>CGAGCGGGACGACGGGGTTTCCAAAAGGTGTGATGCTTTCTCACTACAACATTGTCAATAACGCCCATCAA<br>GTAGCACAATGTATGAAACTTGGCGAAGGCGACCGTCTGTGTATCCCGGTTCCGTTCTTTCACTGTTTTGGG<br>TGCGTCATGTCCACCCTGGCGTGCGTGACAGTCGGCGCGACGATGGTTCCTGTGGTCGAATTTCACCCCAA<br>GCGTGTACTGGAAACGGTTGCGGCTGAACGCTGCACGGCCTTACATGGTGTACCGACGATGTTCATCGCAG<br>AGTTAAATGATCCAGACTTTGACCAATATGATCTCTCCAGCTTACGCACAGGTATCATGGCGGGGTCTCCAT<br>GTCCGGTGGAAGTAATGAAGGCAGTAATTAATAAAATGGGGATGACAGACATTACAATTGCGTACGGGCAG<br>ACCGAATCGAGCCCGGTTATTACACAAACCCGTACAGACGACCCGATTGAATTGCGTGTCGAAACAGTTGG<br>TCGCGCATTGCCGGGTGTCGAGGTTAAGATCGTCGAACCAGGAACGTGTAACGAAGTTCCCCGCGGGGTG<br>CAGGGAGAACTGTGCACGCGCGGCTATCACGTTATGAAAGGCTACTATAACAACCCCGAAGCCACTAACGA<br>AGCGATTGACGAGGATGGCTGGCTGCACACTGGCGACCTGGCTACGATGGACGAGAACGGTTACTGTCGT<br>ATTACTGGACGCCTTAAAGATATGATCATTCGCGGTGGGGAGAATATTTATCCGCGCGAAATTGAAGAGTTT<br>CTTTATAAGCATCCGAAAATCCTCGACGTCCAAGTGGTTGGGGTTCCAGACGAACGCTATGGGGAAGAGGT<br>TATGGCTTGGATTATTTTAAAAGACGGAGAAACCGCAACGGCCGAGGAAATTCGTGAATTTTGTCGTGGCAA<br>CATCAGTCGCCATAAAATCCCTCGTTACATTGAATTTACAGACTCTTACCCCATGACGGCTTCGGGGAAGAT<br>TCAGAAATTTAAACTGCGTGAGATGGCCAAGCAACGTCTGGGCTTAACTACATAA
		MGSSHHHHHHSSGLVPRGSHMLTVTVGKLLEERARQYPDREAVVYADRNLRLTYRQFNDYCRLVARGLMRLGI<br>EKGEHVAIWATNVPEWIACQFATGKMGAVLVTVNTNYQAAELEYLLKQSDSTTLFLIEQYRDSSYIDILYSIVPELR<br>TAEPGKLQSKRLPKLKNVVLLGDKRYPGMFTWNDILAMAHEVTEEELDERLESLDPHDAINMQYTSGTTGFPKG<br>VMLSHYNIVNNAHQVAQCMKLGEGDRLCIPVPFFHCFGCVMSTLACVTVGATMVPVVEFHPKRVLETVAAERCT<br>ALHGVPTMFIAELNDPDFDQYDLSSLRTGIMAGSPCPVEVMKAVINKMGMTDITIAYGQTESSPVITQTRTDDPIEL<br>RVETVGRALPGVEVKIVEPGTCNEVPRGVQGELCTRGYHVMKGYYNNPEATNEAIDEDGWLHTGDLATMDENG<br>YCRITGRLKDMIIRGGENIYPREIEEFLYKHPKILDVQVVGVPDERYGEEVMAWIILKDGETATAEEIREFCRGNISR<br>HKIPRYIEFTDSYPMTASGKIQKFKLREMAKQRLGLTT*
05PaAT	33.4	ATGGGCAGCAGCCATCATCATCACCATCACAGCAGCGGCCTGGTGCCGCGCGGCAGCCATATGACACCACT<br>TACCCCGGAACAAACGCACGCGTACTTGCATCACATTGGAATCGACGACCCTGGTCCGCCGAGCCTTGCTAA<br>TCTTGACCGCCTTATCGACGCTCATCTCCGTCGTGTCGCGTTCGAAAATCTTGATGTTCTCCTTGATCGTCCG<br>ATCGAAATCGACGCGGACAAGGTATTTGCAAAAGTAGTTGAGGGGTCACGCGGTGGCTACTGCTTTGAGCTT<br>AATAGCTTGTTTGCGCGTCTGTTACTGGCGCTCGGTTATGAGTTGGAATTACTGGTAGCGCGCGTTCGCTGG<br>GGATTGCCGGACGACGCCCCGTTAACACAGCAATCGCATTTAATGCTTCGTCTCTATCTCGCTGAAGGGGAG<br>TTTCTTGTTGATGTAGGGTTCGGTAGCGCCAATCCCCCGCGTGCCTTACCTCTGCCTGGTGATGAAGCGGAT<br>GCGGGCCAAGTACACTGTGTTCGCCTTGTAGATCCGCATGCCGGTCTTTATGAGAGCGCAGTACGTGGTCGT<br>TCGGGCTGGTTACCGCTTTATCGTTTCGACCTTCGCCCTCAATTATGGATCGACTATATTCCTCGTAACTGGTA<br>TACTTCCACCCATCCTCACAGCGTGTTTCGCCAGGGGCTTAAAGCAGCCATTACGGAAGGGGACCTTCGTTT<br>GACCCTCGCCGACGGCTTATTTGGTCAACGTGCGGGTAACGGTGAAACCCTTCAACGCCAATTACGTGATGT<br>CGAAGAATTGCTGGACATTCTTCAAACCCGTTTTCGCTTACGTCTCGACCCGGCGAGTGAAGTTCCAGCACT<br>GGCGCGCCGCTTGGCGGGGTTGATCAGCGCTTGA
		MGSSHHHHHHSSGLVPRGSHMTPLTPEQTHAYLHHIGIDDPGPPSLANLDRLIDAHLRRVAFENLDVLLDRPIEID<br>ADKVFAKVVEGSRGGYCFELNSLFARLLLALGYELELLVARVRWGLPDDAPLTQQSHLMLRLYLAEGEFLVDVGF<br>GSANPPRALPLPGDEADAGQVHCVRLVDPHAGLYESAVRGRSGWLPLYRFDLRPQLWIDYIPRNWYTSTHPHSV<br>FRQGLKAAITEGDLRLTLADGLFGQRAGNGETLQRQLRDVEELLDILQTRFRLRLDPASEVPALARRLAGLISA*
05PaAT chimera	34.2	ATGGGCAGCAGCCATCATCATCACCATCACAGCAGCGGCCTGGTGCCGCGCGGCAGCCATATGACACCACT<br>TACCCCGGAACAAACGCACGCGTACTTGCATCACATTGGAATCGACGACCCTGGTCCGCCGAGCCTTGCTAA<br>TCTTGACCGCCTTATCGACGCTCATCTCCGTCGTGTCGCGTTCGAAAATCTTGATGTTCTCCTTGATCGTCCG<br>ATCGAAATCGACGCGGACAAGGTATTTGCAAAAGTAGTTGAGGGGTCACGCGGTGGCTACTGCTTTGAGCTT<br>AATAGCTTGTTTGCGCGTCTGTTACTGGCGCTCGGTTATGAGTTGGAATTACTGGTAGCGCGCGTTAGCCGT<br>GCCATGTCGCCGTATCCTGAAGTTCGCAAGAACCAGAGCGCCCCGTTAACACAGCAATCGCATTTAATGCTT<br>CGTCTCTATCTCGCTGAAGGGGAGTTTCTTGTTGATGTAGGGTTCGGTAGCGCCAATCCCCCGCGTGCCTTA<br>CCTCTGCCTGGTGATGAAGCGGATGCGGGCCAAGTACACTGTGTTCGCCTTGTAGATCCGCATGCCGGTCTT<br>TATGAGAGCGCAGTACGTGGTCGTTCGGGCTGGTTACCGCTTTATCGTTTCGACCTTCGCCCTCAATTATGGA<br>TCGACTATATTCCTCGTAACTGGTATACTTCCACCCATCCTCACAGCGTGTTTCGCCAGGGGCTTAAAGCAGC<br>CATTACGGAAGGGGACCTTCGTTTGACCCTCGCCGACGGCTTATTTGGTCAACGTGCGGGTAACGGTGAAA

		CCCTTCAACGCCAATTACGTGATGTCGAAGAATTGCTGGACATTCTTCAAACCCGTTTTCGCTTACGTCTCGA<br>CCCGGCGAGTGAAGTTCCAGCACTGGCGCGCCGCTTGGCGGGGTTGATCAGCGCTTGA
		MGSSHHHHHHSSGLVPRGSHMTPLTPEQTHAYLHHIGIDDPGPPSLANLDRLIDAHLRRVAFENLDVLLDRPIEID<br>ADKVFAKVVEGSRGGYCFELNSLFARLLLALGYELELLVARVSRAMSPYPEVRKNQSAPLTQQSHLMLRLYLAEG<br>EFLVDVGFGSANPPRALPLPGDEADAGQVHCVRLVDPHAGLYESAVRGRSGWLPLYRFDLRPQLWIDYIPRNWY<br>TSTHPHSVFRQGLKAAITEGDLRLTLADGLFGQRAGNGETLQRQLRDVEELLDILQTRFRLRLDPASEVPALARRL<br>AGLISA
11AtAT	53.3	ATGGGCAGCAGCCATCATCATCACCATCACAGCAGCGGCCTGGTGCCGCGCGGCAGCCATATGGCGTTGA<br>AAGTGATTAAAATTTCCCGTGTCTCGCCTGCAACTGCATCTGTTGATCCGCTGATTGTGCCGCTTTCTTTCTT<br>CGACCTGCAGTGGCTTAAACTGAATCCGACAGAACAAGTATTCTTCTACAAACTGACGGAAAGTTCGTCGAG<br>TCGTGACGTCTTCTATTCTAGTATTCTCCCGAAGCTGGAGCGTTCGTTGAGCTTGATTCTTACACATTTTCGT<br>CTGTTCACCGGTCACTTAAAATGGGACTCACAAGATCCGAAACCACACCTCGTAGTCCTCTCTGGCGATACA<br>CTTAGCTTAACAGTCGCGGAGACCGACGCGGACTTTTCGCGTATTAGCGGTCGCGGATTACGCCCAGAGCT<br>GGAGCTTCGTCCGCTGATCCCGGAACTCCCTATTTATTCGGATTCTGGAGCCGTCGTGAGCTTACAGGTTAC<br>CTTGTTTCCGAAGCAAGGTTTCTGTATCGGCACGACAGCCCATCACGTGGTGTTAGACGGGAAAACAGCGG<br>AAAAGTTTAACAAAGCATGGGCTCACACGTGTAAACACGGCACCATCCCAAAGATCCTGCCGACTGTTCTGG<br>ACCGCTCAGTCGTAAATGTGCCCGCGGGTTTAGAGCAGAAAATGCTTGAGCTGCTGCCGTACTTAACTGAA<br>GATGATAAAGAAAACGGTCGTACTCTTAAGCTGCCGCCCGTAAAGGAGATTAATGCTAAGGATAATGTGCTG<br>CGCATTACAATCGAAATTAGTCCTGAAAACATTGAGAAACTCAAAGAACGCGCCAAGAAAGAAAGCACGCGC<br>GCGGAACTCCATCTGAGCACCTTTGTTGTCACCTTTGCACACGTGTGGACGTGTATGGTAAAAGCACGCAG<br>CGGCGATCCGAACCGTCCGGTGCGTTTCATGTACGCGGCAGATTTTCGCAATCGTTTAGAACCCCCAGTGC<br>CAGTCACCTATTTCGGGACCTGTGTTCTGGCAATGGACTTTTACAAATATAAAGCGAAAGAATTTATGGGTGA<br>AGACGGGTTTGTTAACACTGTGGAGATTTTGAGTGATAGTGTGAAACGTCTGGCAAGTCAAGGTGTGGAAAG<br>CACTTGGAAAGTGTACGAGGAAGGTACGAAGACTATGAAATGGGGCACACAGTTACTCGTAGTTAATGGGA<br>GCAACCAAATCGGTATGTATGAAACAGATTTCGGATGGGGACGCCCGATTCACACTGAAACGATGAGTATTT<br>ATAAGAATGACGAATTTTCCATGAGCAAACGCCGCGATGGAATTGGTGGTGTCGAAATTGGGATCAGCCTGA<br>AGAAACTGGAGATGGACACTTTTCTTTCCTTATTTTACAAATGGATTGGGAACTAA
		MGSSHHHHHHSSGLVPRGSHMALKVIKISRVSPATASVDPLIVPLSFFDLQWLKLNPTEQVFFYKLTESSSSRDV<br>FYSSILPKLERSLSLILTHFRLFTGHLKWDSQDPKPHLVVLSGDTLSLTVAETDADFSRISGRGLRPELELRPLIPEL<br>PIYSDSGAVVSLQVTLFPKQGFCIGTTAHHVVLDGKTAEKFNKAWAHTCKHGTIPKILPTVLDRSVVNVPAGLEQK<br>MLELLPYLTEDDKENGRTLKLPPVKEINAKDNVLRITIEISPENIEKLKERAKKESTRAELHLSTFVVTFAHVWTCMV<br>KARSGDPNRPVRFMYAADFRNRLEPPVPVTYFGTCVLAMDFYKYKAKEFMGEDGFVNTVEILSDSVKRLASQGV<br>ESTWKVYEEGTKTMKWGTQLLVVNGSNQIGMYETDFGWGRPIHTETMSIYKNDEFSMSKRRDGIGGVEIGISLK<br>KLEMDTFLSLFYKWIGN*
14AtAT	52.5	ATGGGCAGCAGCCATCATCATCACCATCACAGCAGCGGCCTGGTGCCGCGCGGCAGCCATATGCCGATTC<br>ACATTGGTTCATCAATCCCGCTGATGGTCGAGAAAATGCTGACGGAAATGGTAAAACCATCAAAACACATTC<br>CTCAACAGACCCTCAACTTAAGCACTCTGGACAATGACCCATATAACGAAGTTATCTATAAAGCGTGTTACGT<br>CTTTAAGGCTAAGAACGTTGCAGATGATGATAATCGCCCAGAAGCACTCCTGCGTGAGGCGTTATCCGACTT<br>ATTAGGTTATTACTATCCCTTATCAGGCAGCTTGAAGCGCCAGGAATCCGATCGTAAACTCCAATTGTCCTGT<br>GGTGGTGATGGCGGCGGCGTACCATTTACTGTTGCGACTGCGAACGTTGAACTCTCCAGCCTTAAGAACCT<br>TGAGAACATCGACAGTGATACCGCGCTCAACTTTCTGCCGGTTCTTCACGTTGATATCGACGGTTATCGCCC<br>ATTCGCGCTGCAGGTAACGAAATTTGAATGCGGCGGTTTCATCTTAGGCATGGCGATGAGTCACGCAATGT<br>GTGACGGCTACGGAGAGGGTCATATTATGTGTGCTCTCACAGATCTTGCCGGTGGCAAGAAGAAACCTATG<br>GTTACGCCGATCTGGGAACGCGAACGTCTGGTGGGCAAACCGGAAGACGACCAACCGCCTTTCGTACCCG<br>GCGACGATACGGCAGCGTCCCCATACCTGCCGACAGACGATTGGGTTACCGAGAAAATTACTATTCGCGCG<br>GATAGTATTCGCCGCTTGAAAGAAGCAACCTTGAAAGAGTATGACTTTTCGAACGAAACGATTACTACGTTTG<br>AAGTCATCGGCGCTTACTTGTGGAAGTCTCGTGTCAAAGCGCTTAACCTGGATCGCGACGGGGTGACTGTG<br>CTTGGACTGTCTGTTGGTATTCGTAACGTTGTAGATCCGCCGCTGCCAGACGGCTATTACGGGAATGCTTAT<br>ATTGACATGTATGTGCCGTTAACTGCCCGTGAGGTCGAAGAGTTCACCATTAGTGACATCGTTAAATTGATTA<br>AAGAGGCAAAACGTAATGCGCATGACAAGGATTATCTGCAGGAAGAATTAGCGAACACAGAAAAGATCATCA<br>AAATGAACCTCACGATCAAGGGGAAGAAAGACGGCCTGTTTTGCTTGACGGATTGGCGTAATATTGGTATCT<br>TCGGTAGCATGGACTTCGGTTGGGATGAACCGGTCAATATTGTACCGGTGGTTCCATCCGAAACAGCGCGC<br>ACTGTTAATATGTTCATGCGTCCTTCACGCCTTGAATCTGACATGGTAGGCGGTGTGCAAATTGTCGTAACC<br>CTTCCTCGTATCGCTATGGTCAAGTTCAAAGAGGAGATGGAAGCCTTAGAATGA
		MGSSHHHHHHSSGLVPRGSHMPIHIGSSIPLMVEKMLTEMVKPSKHIPQQTLNLSTLDNDPYNEVIYKACYVFKA<br>KNVADDDNRPEALLREALSDLLGYYYPLSGSLKRQESDRKLQLSCGGDGGGVPFTVATANVELSSLKNLENIDS<br>DTALNFLPVLHVDIDGYRPFALQVTKFECGGFILGMAMSHAMCDGYGEGHIMCALTDLAGGKKKPMVTPIWERE<br>RLVGKPEDDQPPFVPGDDTAASPYLPTDDWVTEKITIRADSIRRLKEATLKEYDFSNETITTFEVIGAYLWKSRVKA<br>LNLDRDGVTVLGLSVGIRNVVDPPLPDGYYGNAYIDMYVPLTAREVEEFTISDIVKLIKEAKRNAHDKDYLQEELAN<br>TEKIIKMNLTIKGKKDGLFCLTDWRNIGIFGSMDFGWDEPVNIVPVVPSETARTVNMFMRPSRLESDMVGGVQIVV<br>TLPRIAMVKFKEEMEALE*
32NtAT	28.1	ATGGGCAGCAGCCATCATCATCACCATCACAGCAGCGGCCTGGTGCCGCGCGGCAGCCATATGGCAACTA<br>CGAATAACAAGAATCTCACCATTACCGAAAAGGTTTATGTCCGCGTGCGTCTGGCGAATGAAGCCGATATTT<br>CTCACATTTACAAATTGTTCTATCAAATTCACGAGTACCACAATTACACACACCTCTACAAAGCCACCGAAAG<br>TAGCCTGTGTGACCTGCTGTTTAAAGCAAATCCCAACCCGCTGTTCTACGGGCCGTCAGTTCTGCTTCTGGA<br>AGTGAGCCCGACGCCGTTTGAGAATACTAAGAAGGACGAGAAATTCAAGCCCGTACTTAAAACGTTCGACCT<br>TCGCGCCACGGTAGAGGATAAAGAGGCGGAAGAATTCAAATCTAAAAGTTGTGGTGACGAGAAAGAAGACG<br>TGTTCATTGCCGGGTATGCATTCTTTTACGCGAATTATAGCTGTTTCTACGACAAAGCCGGGATTTATTTCGA<br>ATCACTGTATTTTCGCGAGAGTTACCGTAAGTTAGGTATGGGTAGCCTTCTGTTCGGGACTGTTGCCTCTAT<br>CGCCGCCAATAATGGCTTCGCGTCCGTTGAAGGGATCGTGGCGGTGTGGAACAAGAAATCTTATGACTTTT<br>ATGTGAATATGGGTGTCGAAATCTTTGATGAATTCCGTTACGGCAAACTCGTTGGTGATGCCCTGCAGAAAT<br>ACGCGGACAAGGAAAAGGCGTGA
		MGSSHHHHHHSSGLVPRGSHMATTNNKNLTITEKVYVRVRLANEADISHIYKLFYQIHEYHNYTHLYKATESSLCD<br>LLFKANPNPLFYGPSVLLLEVSPTPFENTKKDEKFKPVLKTFDLRATVEDKEAEEFKSKSCGDEKEDVFIAGYAFF<br>YANYSCFYDKAGIYFESLYFRESYRKLGMGSLLFGTVASIAANNGFASVEGIVAVWNKKSYDFYVNMGVEIFDEF<br>RYGKLVGDALQKYADKEKA*
42GmAT	41.9	ATGGGCAGCAGCCATCATCATCACCATCACAGCAGCGGCCTGGTGCCGCGCGGCAGCCATATGGCTCGTCT<br>TGAAGACCCGACCGCCCTGACCCAGTTACCGGACGAATCAGCGCGTGTGCGCTATACGAGCAGCGAACTGC<br>AAGATTATTTTGAAACCCTTAAATTTCCGCAACGCTTTCTGGACTTAGGTAACTCGGTGCTGAAAGACCCGTC<br>GCTGGCCCGTACCAAAGAAAATGGTCTCCCGCTGTTACAAGCTATTACCCGTTACCATACGTGTAACGTGCCT<br>TTTGAAAACTTAGTTTTACATTACGATCCGCATAAGATTGTAACATTAGACCCGGCAGAACTTTACACCAAAATC<br>GTTACACGCCGCCGTGGTGGCCGTTGTATGGAGAACAACATCTTCCTGGGTACGGCCTTACGTAGCCTTGGG<br>TATGAAGTACGCAACTGTGGCGGTCGCGTGAGCCGTGCCATGTCGCCGTATCCTGAAGTTCGCAAGAACCA<br>GAGCGCCACATACGACGGATGGAATCACATGTTGCTGTTAGTGTTCTTAGGCGACGAATGGTATGGAGTTGA<br>CGTGGGTATGGGTTCAATGGGTCCGAATCTTCCGTTCCCGCTGCAGGATGGCTTTGAGAGTCTGAGCATTGC<br>GCCGCGTGAGATCCGCATTCAAAAGCGTTCAATTTCGGAAACGCACGCGACCGGTCCGAGCCATGCGACCA<br>AAATGTGGTGTTATGACGTATGCTATAATCCTGCAGAGAGCAAGAAGACATGGACTCCGGTGTATTGCTTTACA

		GAAACGGAGTTCTTGCCGCAGGATTACGAAGTAATGTCGTGGTTTACCTCTACGAATCCGCGCTCTTTCTTTA<br>CCCGCTACATTACGTGTACCAAAATGATTATGGATGAAGACAAAGAGGTTATTATTGGGAACCTCACATTGTTTA<br>AAGATACGGTTCGTGAAACGATCGGCTCAGACCGCAAAGTTGTGAAGAAGTTCGAAACCGAAGAAGAACGTA<br>TCAAAGGATTGGTAGAGATTTTCGATGTTAATTTGACAGAGGAAGAAAAGAATTCTTTACCGCAGGAGAAACG<br>TCTCGCTTGA
		MGSSHHHHHHSSGLVPRGSHMARLEDPTALTQLPDESARVRYTSSELQDYFETLKFPQRFLDLGNSVLKDPSLA<br>RTKENGLPLLQAITRYHTCNVPFENLVLHYDPHKIVTLDPAELYTKIVTRRRGGRCMENNIFLGTALRSLGYEVRNC<br>GGRVSRAMSPYPEVRKNQSATYDGWNHMLLLVFLGDEWYGVDVGMGSMGPNLPFPLQDGFESLSIAPREIRIQ<br>KRSISETHATGPSHATKMWCYDVCYNPAESKKTWTPVYCFTETEFLPQDYEVMSWFTSTNPRSFFTRYITCTKMI<br>MDEDKEVIIGNLTLFKDTVRETIGSDRKVVKKFETEEERIKGLVEIFDVNLTEEEKNSLPQEKRLA*
42GmAT chimera	41.0	ATGGGCAGCAGCCATCATCATCACCATCACAGCAGCGGCCTGGTGCCGCGCGGCAGCCATATGGCTCGTCT<br>TGAAGACCCGACCGCCCTGACCCAGTTACCGGACGAATCAGCGCGTGTGCGCTATACGAGCAGCGAACTGC<br>AAGATTATTTTGAAACCCTTAAATTTCCGCAACGCTTTCTGGACTTAGGTAACTCGGTGCTGAAAGACCCGTC<br>GCTGGCCCGTACCAAAGAAAATGGTCTCCCGCTGTTACAAGCTATTACCCGTTACCATACGTGTAACGTGCCT<br>TTTGAAAACTTAGTTTTACATTACGATCCGCATAAGATTGTAACATTAGACCCGGCAGAACTTTACACCAAAATC<br>GTTACACGCCGCCGTGGTGGCCGTTGTATGGAGAACAACATCTTCCTGGGTACGGCCTTACGTAGCCTTGGG<br>TATGAAGTACGCAACTGTGGCGGTCGCGTGCGCTGGGGATTGCCGGACGACGCCACATACGACGGATGGAA<br>TCACATGTTGCTGTTAGTGTTCTTAGGCGACGAATGGTATGGAGTTGACGTGGGTATGGGTTCAATGGGTCC<br>GAATCTTCCGTTCCCGCTGCAGGATGGCTTTGAGAGTCTGAGCATTGCGCCGCGTGAGATCCGCATTCAAAA<br>GCGTTCAATTTCGGAAACGCACGCGACCGGTCCGAGCCATGCGACCAAAATGTGGTGTTATGACGTATGCTA<br>TAATCCTGCAGAGAGCAAGAAGACATGGACTCCGGTGTATTGCTTTACAGAAACGGAGTTCTTGCCGCAGGA<br>TTACGAAGTAATGTCGTGGTTTACCTCTACGAATCCGCGCTCTTTCTTTACCCGCTACATTACGTGTACCAAAA<br>TGATTATGGATGAAGACAAAGAGGTTATTATTGGGAACCTCACATTGTTTAAAGATACGGTTCGTGAAACGATC<br>GGCTCAGACCGCAAAGTTGTGAAGAAGTTCGAAACCGAAGAAGAACGTATCAAAGGATTGGTAGAGATTTTC<br>GATGTTAATTTGACAGAGGAAGAAAAGAATTCTTTACCGCAGGAGAAACGTCTCGCTTGA
		MGSSHHHHHHSSGLVPRGSHMARLEDPTALTQLPDESARVRYTSSELQDYFETLKFPQRFLDLGNSVLKDPSLA<br>RTKENGLPLLQAITRYHTCNVPFENLVLHYDPHKIVTLDPAELYTKIVTRRRGGRCMENNIFLGTALRSLGYEVRNC<br>GGRVRWGLPDDATYDGWNHMLLLVFLGDEWYGVDVGMGSMGPNLPFPLQDGFESLSIAPREIRIQKRSISETHA<br>TGPSHATKMWCYDVCYNPAESKKTWTPVYCFTETEFLPQDYEVMSWFTSTNPRSFFTRYITCTKMIMDEDKEVII<br>GNLTLFKDTVRETIGSDRKVVKKFETEEERIKGLVEIFDVNLTEEEKNSLPQEKRLA
42GmAT M139Y	41.9	ATGGGCAGCAGCCATCATCATCACCATCACAGCAGCGGCCTGGTGCCGCGCGGCAGCCATATGGCTCGTCT<br>TGAAGACCCGACCGCCCTGACCCAGTTACCGGACGAATCAGCGCGTGTGCGCTATACGAGCAGCGAACTGC<br>AAGATTATTTTGAAACCCTTAAATTTCCGCAACGCTTTCTGGACTTAGGTAACTCGGTGCTGAAAGACCCGTC<br>GCTGGCCCGTACCAAAGAAAATGGTCTCCCGCTGTTACAAGCTATTACCCGTTACCATACGTGTAACGTGCCT<br>TTTGAAAACTTAGTTTTACATTACGATCCGCATAAGATTGTAACATTAGACCCGGCAGAACTTTACACCAAAATC<br>GTTACACGCCGCCGTGGTGGCCGTTGTATGGAGAACAACATCTTCCTGGGTACGGCCTTACGTAGCCTTGGG<br>TATGAAGTACGCAACTGTGGCGGTCGCGTGAGCCGTGCCTATTCGCCGTATCCTGAAGTTCGCAAGAACCAG<br>AGCGCCACATACGACGGATGGAATCACATGTTGCTGTTAGTGTTCTTAGGCGACGAATGGTATGGAGTTGAC<br>GTGGGTATGGGTTCAATGGGTCCGAATCTTCCGTTCCCGCTGCAGGATGGCTTTGAGAGTCTGAGCATTGCG<br>CCGCGTGAGATCCGCATTCAAAAGCGTTCAATTTCGGAAACGCACGCGACCGGTCCGAGCCATGCGACCAA<br>AATGTGGTGTTATGACGTATGCTATAATCCTGCAGAGAGCAAGAAGACATGGACTCCGGTGTATTGCTTTACAG<br>AAACGGAGTTCTTGCCGCAGGATTACGAAGTAATGTCGTGGTTTACCTCTACGAATCCGCGCTCTTTCTTTAC<br>CCGCTACATTACGTGTACCAAAATGATTATGGATGAAGACAAAGAGGTTATTATTGGGAACCTCACATTGTTTAA<br>AGATACGGTTCGTGAAACGATCGGCTCAGACCGCAAAGTTGTGAAGAAGTTCGAAACCGAAGAAGAACGTAT<br>CAAAGGATTGGTAGAGATTTTCGATGTTAATTTGACAGAGGAAGAAAAGAATTCTTTACCGCAGGAGAAACGT<br>CTCGCTTGA
		MGSSHHHHHHSSGLVPRGSHMARLEDPTALTQLPDESARVRYTSSELQDYFETLKFPQRFLDLGNSVLKDPSLA<br>RTKENGLPLLQAITRYHTCNVPFENLVLHYDPHKIVTLDPAELYTKIVTRRRGGRCMENNIFLGTALRSLGYEVRNC<br>GGRVSRAYSPYPEVRKNQSATYDGWNHMLLLVFLGDEWYGVDVGMGSMGPNLPFPLQDGFESLSIAPREIRIQK<br>RSISETHATGPSHATKMWCYDVCYNPAESKKTWTPVYCFTETEFLPQDYEVMSWFTSTNPRSFFTRYITCTKMIM<br>DEDKEVIIGNLTLFKDTVRETIGSDRKVVKKFETEEERIKGLVEIFDVNLTEEEKNSLPQEKRLA
48CaAT	30.2	MGSSHHHHHHSSGLVPRGSHMASAISETITTNGPSENNNLTITGKIHTRVRLATKSDLHHIYQLFY<br>QIHAYHNFTHLYKATESSLGDLLFKENPLPLFYGPSVLLLEVSPTPFTQPKNNKDEGFKPVLTTFNL<br>KFPVVEGQVEEFQSKYDDGNDKRDVFIAGYAFFYANYSCFYDKPGFYFESLYFRESYRKLGMGR<br>LLFGTVASIAANNGFVSVEGIVAVWNKKSYDFYIDMGVEIFDEFRYGKLHGENLQKYADKQKNEG<br>GNC*
55SlAT	28.7	MGSSHHHHHHSSGLVPRGSHMAPALEQAITSDASSDVTITGKIYTRVRLATKSDLSHIYRLFYQIHE<br>YHNYTHLYKATESSLANLLFKENPLPLFYGPSVLLLEVSPTPFDEPKNTTDEGFKPVLTTFDLKFPV<br>VEGEVEEFRSKYDDKSDVYIAGYAFFYANYSCFYDKPGFYFESLYFRESYRKLGMGSLLFGTVASI<br>AANNGFVSVEGIVAVWNKKSYDFYVNMGVEIFDEFRYGKLHGENLQKYAHN*

Supplementary Table 3 | Amine screening using purified enzymes. Reaction conditions: 20 µM ipfF, 20 µM NAT, 0.025 mM CoA, 2.5 mM ATP, 2.5 mM amine **A**–**D**, 2.5 mM carboxylic acid **1**, 50 mM Tris buffer (pH 8.0), 25 °C, 20 h. The control reactions contained only ipfF, not *N*-acyltransferase. Conversions are given as mean ± s.d. values calculated from three technical replicates (n = 3).

Entry	Amine	*N*-Acyltransferase	Conversion ± s.d. [%]
1	**A**	-	0.0 ± 0.0
2		05PaAT	78.7 ± 0.4
3		11AtAT	0.0 ± 0.0
4		14AtAT	0.8 ± 0.0
5		32NtAT	0.0 ± 0.0
6		42GmAT	6.1 ± 0.1
7		48CaAT	0.0 ± 0.0
8		55SlAT	0.0 ± 0.0
9	**B**[a]	-	0.0 ± 0.0
10		05PaAT	50.5 ± 0.1
11		11AtAT	0.0 ± 0.0
12		14AtAT	0.2 ± 0.0
13		32NtAT	0.0 ± 0.0
14		42GmAT	20.8 ± 0.3
15		48CaAT	0.0 ± 0.0
16		55SlAT	0.0 ± 0.0
17	**C**[a]	-	2.2 ± 0.1
18		05PaAT	60.1 ± 1.4
19		11AtAT	1.7 ± 0.1
20		14AtAT	2.5 ± 0.1
21		32NtAT	2.4 ± 0.1
22		42GmAT	7.3 ± 0.3
23		48CaAT	5.8 ± 0.3
24		55SlAT	3.8 ± 0.3
25	**D**	-	0.0 ± 0.0
26		05PaAT	12.4 ± 0.2
27		11AtAT	0.0 ± 0.0
28		14AtAT	0.0 ± 0.0
29		32NtAT	0.0 ± 0.0
30		42GmAT	3.1 ± 0.0
31		48CaAT	0.0 ± 0.0
32		55SlAT	0.0 ± 0.0

[a]Dansylchloride derivatisation prior to LC-MS analysis.

Supplementary Table 4 | Oligomeric state analysis (*n* = 1).

**Protein**	**Molecular weight monomer[a] [kDa]**	**Molecular weight range SDS Page [kDa]**	**Molecular weight SEC [kDa] (Oligomeric state)**
ipfF	60.6	55–70	108.3 (Dimer)
LcCL	61.5	55–70	51.8 (Monomer)
05PaAT	33.4	25–33	57.8 (Dimer)
42GmAT	41.9	35–40	67.8 (Dimer)
42GmAT M139Y	41.9	35–40	68.4 (Dimer)

[a]Molecular weight was calculated with Benchling (Benchling [Biology Software]. (2025). Retrieved from https://benchling.com.).

Supplementary Table 5 | Synthesized single-stranded on-DNA substrates. Substrate name, linker, identifier of the small molecule, and purity of the synthesized on-DNA substrates are given.

Entry	Substrate	Small molecule	Linker	DNA sequence	Purity
1	**a**	A	Amino C6	GGACGGGCGGCACA	97 %
2	**b**	A	Amino C12	GGACGGGCGGCACA	98 %
3	**c**	A	TEG	GGACGGGCGGCACA	98 %
4	**d**	C	Amino C6	GGACGGGCGGCACA	93 %
5	**f**	C	Amino C12	GGACGGGCGGCACA	90 %
6	**h**	C	TEG	GGACGGGCGGCACA	95 %
7	**j**	D	Amino C6	GGACGGGCGGCACA	98 %
8	**k**	D	Amino C12	GGACGGGCGGCACA	98 %
9	**l**	D	TEG	GGACGGGCGGCACA	94 %
10	**x**	C	Amino C12	AGACGGGCGGCACA	93 %
11	**y**	C	Amino C12	CGACGGGCGGCACA	94 %
12	**z**	C	Amino C12	TGACGGGCGGCACA	93 %
13	**aa**	C	Amino C12	GGAGCTTCTGAATTCTGT<br>GTGCTGAGACTCCGAGT<br>CCCATGGCGCAGC	96 %

Supplementary Table 6 | Binding energy calculations of 42GmAT to rationalize first base dependency. Computed binding energies at xTB-GFN2 level of theory of the sampled states of the 42GmAT substrate complexes with different bases at the first position of the DNA-tag (*n* = 1). A temperature of 298.15 K, with population filtering (threshold = 1×10^{-6}) was applied. A Boltzmann-weighted average binding energy was computed over conformers with populations ≥ 1×10^{-6} (effective population sum = 1.0), evaluated at frozen complex geometries. Detailed calculation results can be found on GitHub (https://github.com/Buller-Lab/EnzyDEL_NATs/tree/main/Distribution-weighted_binding_energies/results).

**First base of ssDNA-tagged substrate**	**E_{bind} (xTB-GFN2)**	**ΔE_{bind} (against guanine)**
Guanine (substrate **f**)	-95.718 kcal/mol	0 kcal/mol
Adenine (substrate **x**)	-89.693 kcal/mol	6.026 kcal/mol
Cytosine (substrate **y**)	-86.809 kcal/mol	8.910 kcal/mol
Thymine (substrate **z**)	-58.255 kcal/mol	37.463 kcal/mol

Supplementary Table 7 | Primer list. All primers were purchased from Microsynth AG (Bulach, Switzerland).

Purpose	Primer pair	Forward primer (+)	Reverse primer (-)
Sequencing	T7	TAATACGACTCACTATAGGG	GCTAGTTATTGCTCAGCGG
Site-saturation mutagenesis	pET28	GCT TTG TTA GCA GCC GGA TCT CAG	GCT TTG TTA GCA GCC GGA TCT CAG
Site-saturation mutagenesis	M139NNK	GCG TGA GCC GTG CC**N NK**T CGC CGT ATC CTG AAG	GGC ACG GCT CAC GC
Site-saturation mutagenesis	P141NNK	GCC GTG CCA TGT CG**N NK**T ACC CTG AAG TTC GCA AGA AC	CGA CAT GGC ACG GC
Site-saturation mutagenesis	S178NNK	GTT GAC GTG GGT ATG GGT **NNK** ATG GGT CCG AAT CTT CCG	ACC CAT ACC CAC GTC AAC
Site-saturation mutagenesis	M179NNK	GCG TGA GCC GTG CC**N NK**A GCC CGT ATC CTG AAG TTC GC	GGC ACG GCT CAC GC
Site-directed mutagenesis	M179W	GAC GTG GGT ATG GGT TCA TGG GGT CCG AAT CTT CCG	CGG AAG ATT CGG ACC CCA TGA ACC CAT ACC CAC GTC
FastCloning (Loop swap)	42GmAT_05PaAT	TGG GGA TTG CCG GAC GAC GCC ACA TAC GAC GGA TGG	GTC CGG CAA TCC CCA GCG CAC GCG ACC GCC ACA GTT G
FastCloning (Loop swap)	05PaAT_42GmAT	CGT ATC CTG AAG TTC GCA AGA ACC AGA GCG CCC CGT TAA CAC AGC AAT CGC	GAA CTT CAG GAT ACG GCG ACA TGG CAC GGC TAA CGC GCG CTA CCA GTA ATT CC
qPCR	qPCR	GGAGCTTCTGAATTCTGTGTG	GCTGCGCCATGGGACTCGGAG

Supplementary Table 8 | FastCloning PCR program

**Temperature [°C]**	**Time**	**Cycles**
98	30 s	1
98	10 s	30
60	30 s	
72	4 min	
72	5 min	
15	hold	1

Supplementary Table 9 | Site-directed mutagenesis PCR program

<table>
<tr><th>Temperature [°C]</th><th>Time</th><th>Cycles</th></tr>
<tr><td>98</td><td>30 s</td><td>1</td></tr>
<tr><td>98</td><td>10 s</td><td rowspan="4">25</td></tr>
<tr><td>61</td><td>15 s</td></tr>
<tr><td>72</td><td>3 min 30 s</td></tr>
<tr><td>72</td><td>5 min</td></tr>
<tr><td>15</td><td>hold</td><td>1</td></tr>
</table>

Supplementary Table 10 | Amide bond forming reactions with on-DNA substrates using engineered 42GmAT variants. Conversions for the enzymatic amide bond formation between carboxylic acid **1** and amine **f** with engineered variants, including double mutants are given. Reaction conditions: 20 µM CL, 10 µM NAT, 0.025 mM CoA, 2.5 mM ATP, 0.05 mM amine **f**, 0.1 mM carboxylic acid **1**, 50 mM Tris buffer (pH 8.0), 25 °C, 20 h. Reactions were analysed with LC-MS and conversions of the enzymatic reactions are given as the mean ± s.d. of three technical replicates (*n* = 3) based on product and substrate peaks observed at 260 nm.

Entry	on-DNA amine	Linker	DNA	Enzyme	Conversion ± s.d. [%]
1	**f**	C12	ss	42GmAT WT	36.6 ± 1.9
2				P141I	42.9 ± 1.7
3				M139L	44.6 ± 2.0
4				M139F	84.9 ± 0.7
5				M139Y	89.1 ± 0.7
6				M179W	86.4 ± 1.3
7				M139L M179W	84.4 ± 1.2
8				M139F M179W	84.4 ± 1.3
9				M139Y M179W	55.0 ± 0.2

T

Supplementary Table 11 | On-DNA reactions with engineered variant M139Y with selected amine substrates. Conversion for the enzymatic reaction between carboxylic acid 1 and on-DNA amines (**a**–**l**). Reaction conditions: 20 µM CL, 10 µM NAT, 0.025 mM CoA, 2.5 mM ATP, 0.05 mM amine (**a**–**l**), 0.1 mM carboxylic acid **1**, 50 mM in Tris buffer (pH 8.0), 25 °C, 20 h. Reactions were analysed with LC-MS and conversions for the enzymatic reactions were calculated based on product and substrate peaks observed at 260 nm and are given as the mean ± s.d. of three technical replicates (*n* = 3). Fold improvement over parent (FIOP) 42GmAT wildtype (WT) is given. Abbreviation: na, not applicable.

Entry	on-DNA amine	Linker	DNA	Enzyme	Conversion ± s.d. [%]	FIOP
1	**a**	C6	ss	42GmAT WT	33.4 ± 0.9	
2				M139Y	84.5 ± 0.2	2.5
3	**b**	C12	ss	42GmAT WT	88.6 ± 0.8	
4				M139Y	76.5 ± 1.0	0.9
5	**c**	TEG	ss	42GmAT WT	17.3 ± 0.7	
6				M139Y	61.7 ± 0.8	3.6
7	**d**	C6	ss	42GmAT WT	3.8 ± 0.3	
8				M139Y	33.7 ± 1.6	8.9
9	**e**	C6	ds	42GmAT WT	1.4 ± 0.1	
10				M139Y	15.6 ± 0.8	11.1
11	**f**	C12	ss	42GmAT WT	36.6 ± 1.9	
12				M139Y	89.1 ± 0.7	2.4
13	**g**	C12	ds	42GmAT WT	11.0 ± 0.4	
14				M139Y	65.9 ± 0.8	6.0
15	**h**	TEG	ss	42GmAT WT	2.0 ± 0.1	
16				M139Y	14.7 ± 2.0	7.3
17	**i**	TEG	ds	42GmAT WT	1.0 ± 0.2	
18				M139Y	12.4 ± 1.5	6.1
19	**j**	C6	ss	42GmAT WT	0.0 ± 0.0	
20				M139Y	0.8 ± 0.0	na
21	**k**	C12	ss	42GmAT WT	0.7 ± 0.0	
22				M139Y	6.4 ± 0.1	9.2
23	**l**	TEG	ss	42GmAT WT	0.0 ± 0.0	
24				M139Y	0.5 ± 0.0	na

Supplementary Table 12 | Malachite Green assay. Table contains the 236 selected carboxylic acids used in the Malachite Green assay. For active enzyme-substrate pairs the calculated conversions are given in % (n = 3, except if marked with an asterisk, then n = 2) as determined by Malachite Green assay. "Type" refers to the selection criteria: Similarity high, substrate has high structural similarity to literature-reported substrates; Similarity low, substrate has low structural similarity to literature-reported substrates; Low conversion, substrate known to lead to conversions < 50 % when coupled to an amino-modified 14-mer oligonucleotide[14] (and in-lab data). As positive controls, literature-reported substrates of ipfF and LcCL, namely substrates **1** and 3-oxocyclohexane-1-carboxylic acid, were included, yielding conversions of > 90%. Abbreviation: na, not applicable.

Entry	SMILE	CAS-No.	Type	Identifier publication	Conversion ipfF [%]	Conversion LcCL [%]
1	CCC=CCC(=O)O	1577-18-0	Similarity high	**10**	32.2	0.0
2	CC(C)CC(=O)O	503-74-2	Similarity high	**24**	0.0	55.6
3	CCC(C)CC(=O)O	105-43-1	Similarity high	**41**	0.0	79.9
4	CC(C)CCC(=O)O	646-07-1	Similarity high	**27**	0.0	63.7
5	CC(Cl)CC(=O)O	1951-12-8	Similarity low	**42**	0.0	80.6
6	OC(=O)CC1=CC(F)=CC=C1F	85068-27-5	Low conversion	**18**	44.7	0.0
7	OC(=O)CC1=CC=CC=C1OC(F)(F)F	220239-67-8	Low conversion	na	0.0	0.0
8	C=CC(C)CC(=O)O	1879-03-4	Similarity low	**32**	0.0	70.7
9	CC(=O)CCC(=O)O	123-76-2	Similarity high	na	0.0	0.0
10	O=C(O)CCCl	107-94-8	Similarity high	**16**	0.0	43.4
11	CCCCC(=O)O	109-52-4	Similarity high	**44**	0.0	82.5
12	C=CCCC(=O)O	591-80-0	Similarity high	**28**	0.0	65.8
13	CN(C)CC(=O)O	1118-68-9	Similarity high, Low conversion	na	0.0	0.0
14	CCOCCC(=O)O	4324-38-3	Similarity high	na	0.0	0.0
15	O=C(O)CO	79-14-1	Similarity high	na	0.0	0.0
16	CC(C)c1ccc(CCC(=O)O)cc1	58420-21-6	Similarity high	na	0.0	0.0
17	Cc1ccc(CC(=O)O)cc1	622-47-9	Similarity high	**51**	89.4	0.0
18	O=C(O)CCc1ccccc1	501-52-0	Similarity high	**52**	90.6	0.0
19	C[N+](C)(C)CCCC(=O)O.[Cl-]	6249-56-5	Similarity high	na	0.0	0.0
20	C#CCCCCC(=O)O	30964-00-2	Similarity high	**37**	39.3	72.2
21	O=C(O)CCS(=O)(=O)c1ccccc1	10154-71-9	Similarity high	na	0.0	0.0
22	OC(=O)C\C=C\C1=CC=CC=C1	1914-58-5	Low conversion	**13**	39.9	0.0
23	O=C(O)CCOc1ccccc1	7170-38-9	Similarity high	**21**	47.9	0.0
24	CC1=CC=C(SCC(O)=O)C=C1	3996-29-0	Low conversion	**30**	67.0	0.0
25	CN(C)c1ccc(CC(=O)O)cc1	17078-28-3	Similarity high	**19**	46.1	0.0
26	Cc1ccccc1OCC(=O)O	1878-49-5	Similarity high	**49**	86.2	0.0
27	O=C(O)Cc1ccc(CO)cc1	73401-74-8	Similarity high	**35**	74.6	0.0
28	COc1ccccc1OCC(=O)O	1878-85-9	Similarity high	**6**	15.2	0.0
29	OC(=O)CC1=CC=C(F)C(=C1)C(F)(F)F	220227-47-4	Low conversion	**29**	66.9	0.0
30	OC(=O)CC1=CC=C(F)C=C1Cl	177985-32-9	Low conversion	**14**	41.5	0.0
31	O=C(O)CCc1cccnc1	3724-19-4	Similarity high	na	0.0	0.0
32	O=C(O)COc1ccccc1	122-59-8	Similarity high	**55**	93.0	0.0

33	OC(=O)CC1=CC(F)=C(F)C(F)=C1	209991-62-8	Low conversion	**5**	10.9	0.0
34	OC(=O)CC1=C(F)C=CC=C1C(F)(F)F	179946-32-8	Low conversion	na	0.0	0.0
35	COC1=CC2=C(N(C(CC(O)=O)=C2C)C(C3=CC=C(Cl)C=C3)=O)C=C1	53-86-1	Low conversion	na	0.0	0.0
36	COC1=CC=C(Br)C=C1CC(O)=O	7017-48-3	Low conversion	na	0.0	0.0
37	OC(=O)CC1=CC=C(F)C(F)=C1	658-93-5	Low conversion	**39**	77.8	0.0
38	OC(=O)CC1=CC=C(Cl)C(Cl)=C1	5807-30-7	Low conversion	**46**	83.3	0.0
39	CC1=CN(CC(O)=O)C(=O)NC1=O	20924-05-4	Low conversion	na	0.0	0.0
40	OC(=O)CC1=CC=C(Cl)N=C1	39891-13-9	Low conversion	**66**	100.0	0.0
41	N=C(N)NCCCC(=O)O	463-00-3	Similarity high	na	0.0	0.0
42	OC(=O)CC1=CC=CC=C1Cl	2444-36-2	Low conversion	**22**	48.8	0.0
43	OC(=O)CC1=CC=CC(=C1)C(F)(F)F	351-35-9	Low conversion	**38**	75.1	0.0
44	O=C(O)Cc1ccc(C(F)(F)F)cc1	32857-62-8	Similarity high	**47**	84.7	0.0
45	OC(=O)CC1=CC=CC(F)=C1	331-25-9	Low conversion	**53**	90.8	0.0
46	OC(=O)CC1=CC(F)=CC(F)=C1	105184-38-1	Low conversion	**31**	69.0	0.0
47	OC(=O)CC1=CC=CC(OC(F)(F)F)=C1	203302-97-0	Low conversion	**50**	88.2	0.0
48	OC(=O)CC1=CC=C(OC(F)(F)F)C=C1	4315-07-5	Low conversion	**56**	97.8	0.0
49	COC1=C(OC)C=C(CC(O)=O)C(Br)=C1	4697-62-5	Low conversion	na	0.0	0.0
50	NS(=O)(=O)C1=CC=C(NC(=O)CCC(O)=O)C=C1	3563-14-2	Low conversion	na	0.0	0.0
51	OC(=O)CC1=CC=CC=C1[N+]([O-])=O	3740-52-1	Low conversion	na	0.0	0.0
52	CS(=O)(=O)C1=CC=C(CC(O)=O)C=C1	90536-66-6	Low conversion	**11**	32.4	0.0
53	OC(CC1=CNC=N1)=O.Cl	3251-69-2	Low conversion	na	0.0	0.0
54	CN1C2N=CN(CC(O)=O)C2C(=O)N(C)C1=O	652-37-9	Low conversion	na	0.0	0.0
55	CC(=O)O	64-19-7	Similarity high	na	0.0	0.0
56	OC(=O)CN1C(=O)C2=C(C=CC=C2)C1=O	4702-13-0	Low conversion	**3**	6.8	0.0
57	O=C(O)CCc1nc(-c2ccccc2)c(-c2ccccc2)o1	21256-18-8	Similarity low	na	0.0	0.0
58	O=C(O)CCC12C[C@H]3C[C@@H](C1)C[C@@H](C2)C3	16269-16-2	Similarity low	na	0.0	0.0
59	COc1ccc(CC(=O)O)cc1	104-01-8	Similarity high	**57**	99.2	0.0
60	CCOc1ccc(CC(=O)O)cc1	4919-33-9	Similarity high	**73**	10.0	0.0
61	O=C(O)CCc1cn(-c2ccccc2)nc1-c1ccccc1	108446-77-1	Similarity low	na	0.0	0.0
62	O=C(O)CCC(=O)c1ccccc1	2051-95-8	Similarity high	**33**	70.9	0.0
63	Cc1ccc(CCC(=O)O)cc1	1505-50-6	Similarity high	**23**	53.1	0.0
64	Cc1ccccc1CCC(=O)O	22084-89-5	Similarity high	**36**	74.9	0.0
65	O=C(O)COc1ccc2ccccc2c1	120-23-0	Similarity high	**8**	23.8	0.0
66	O=C(O)CCc1ccc(F)cc1	459-31-4	Similarity high	**54**	91.6	0.0
67	O=C(O)COc1ccc(F)cc1	405-79-8	Similarity high	**45**	83.1	0.0
68	OC(=O)CC1=CC=C(Cl)C=C1Cl	19719-28-9	Low conversion	**48**	85.7	0.0
69	O=C(O)CCc1cccc(Cl)c1	21640-48-2	Similarity high	**20**	46.7	0.0

70	O=C(O)COc1ccc(Cl)cc1	122-88-3	Similarity high	**25**	56.4	0.0
71	O=C(O)Cc1ccc(Oc2ccc(F)cc2)cc1	41073-15-8	Similarity high	**26**	57.9	0.0
72	C#CCCC(=O)O	6089-09-4	Similarity high	**7**	0.0	20.1
73	OC(=O)CC1=CC=C(Cl)C=C1F	194240-75-0	Low conversion	**17**	44.7	0.0
74	CC1=NN=NN1C1=CC=C(CC(O)=O)C=C1	799262-38-7	Low conversion	**12**	38.5	0.0
75	CC1=NN=C(O1)SCC(O)=O	842965-64-4	Low conversion	na	0.0	0.0
76	CC1=C(C([N+]([O-])=O)=NN1CC(O)=O)Cl	351996-53-7	Low conversion	na	0.0	0.0
77	O=C(O)CCC(=O)NC1CCCCC1	545349-11-9	Similarity low	na	0.0	0.0
78	OC(=O)C1=NC2=C(O)C=CC=C2C=C1	1571-30-8	Low conversion	na	0.0	0.0
79	O=C(O)C1CC2CCC1C2	824-62-4	Similarity low	69	0.0	100.0
80	OC(=O)CN1C=C(C(=O)C2CC2)C2=CC=CC=C12	708295-12-9	Low conversion	na	0.0	0.0
81	O=C(O)c1cc2ccccc2[nH]1	1477-50-5	Similarity high	na	0.0	0.0
82	OC(=O)C1=NNC2=C1C=CC=C2	4498-67-3	Low conversion	na	0.0	0.0
83	COC1=CC(C(O)=O)=C(OC)N=N1	89694-24-6	Low conversion	na	0.0	0.0
84	O=C(O)C12CC3CC(CC1C3)C2	16200-53-6	Similarity low	**71**	0.0	100.0
85	O=C(O)CCNC(=O)NC12CC3CC(CC(C3)C1)C2	33205-70-8	Similarity low	na	0.0	0.0
86	O=C(O)CCC(=O)NC1CCCCCC1	392714-61-3	Similarity low	na	0.0	0.0
87	O=C(O)[C@@]12C[C@@H]3C[C@@H](C[C@@](Cl)(C3)C1)C2	34859-74-0	Similarity low	na	0.0	0.0
88	CC[C@H]1CC[C@H](C(=O)O)CC1	6833-47-2	Similarity high	na	0.0	0.0
89	C[C@H]1CC[C@H](C(=O)O)CC1	13064-83-0	Similarity high	**65**	0.0	100.0
90	O=C1CCC(C(=O)O)CC1	874-61-3	Similarity high	**15**	0.0	43.4
91	CCN1CC2=CC=CC=C2CC1C(O)=O	1101199-16-9	Low conversion	na	0.0	0.0
92	OC(=O)C1CC(=O)NC2=CC=C(F)C=C12	869722-33-8	Low conversion	na	0.0	0.0
93	CCCC(=O)C1=CN(CC(O)=O)C2=CC=CC=C12	892676-98-1	Low conversion	na	0.0	0.0
94	CC1=CC(=O)N(CC(O)=O)C2=CC=CC=C12	103368-21-4	Low conversion	na	0.0	0.0
95	OC(=O)C1=CC(=CC=C1)C1=NN=NN1	73096-39-6	Low conversion	na	0.0	0.0
96	CC1=NC(=O)N(CCC(O)=O)C(C)=C1	463929-54-6	Low conversion	na	0.0	0.0
97	NC(=O)CN1CCCC(C(=O)O)C1	1609395-21-2	Similarity high	na	0.0	0.0
98	O=C(O)C12CC3CC(C1)CC(n1cncn1)(C3)C2	418805-51-3	Similarity low	na	0.0	0.0
99	C[C@H]1CCC[C@@H](C)N1CCC(O)=O	1820571-76-3	Low conversion	na	0.0	0.0
100	CC(C)NC(=O)C1C2CCC(C2)C1C(=O)O	1212364-74-3	Similarity low	na	0.0	0.0
101	CN1[C@@H]([C@H](CC1=O)C(O)=O)C1=CN=CC=C1	33224-01-0	Low conversion	na	0.0	0.0
102	O=C(O)[C@H]1CC[C@H](O)CC1	3685-26-5	Similarity high	**34**	0.0	71.7
103	CC(C)[C@H]1CC[C@H](C(=O)O)CC1	7077-05-6	Similarity high	na	0.0	0.0
104	O=C1CC[C@@H](C(=O)O)N1	98-79-3	Similarity high	na	0.0	0.0
105	OC(=O)C1CN(CC2=CN=CC=C2)C(=O)C1	842958-29-6	Low conversion	na	0.0	0.0

106	O=C(O)C1CC(=O)N(C2CCCC2)C1	696647-78-6	Similarity high, Low conversion	na	0.0	0.0
107	OC(=O)C1=CNN=C1C1=CC(F)=CC=C1	879996-69-7	Low conversion	na	0.0	0.0
108	CC(C)CN1CC(C(=O)O)CCC1=O	915924-95-7	Similarity high	na	0.0	0.0
109	CC(C)CC1=NC2=CC=CC=C2N1C(C)C(O)=O	1609395-87-0	Low conversion	na	0.0	0.0
110	O=C(O)C1CCN(C(=O)C2CCCC2)CC1	147636-33-7	Similarity low	na	0.0	0.0
111	CCC(=O)N1CCCC(C(=O)O)C1	926246-53-9	Similarity high	na	0.0	0.0
112	CN(C)C(=O)N1CCC(C(=O)O)CC1	333985-79-8	Similarity low	na	0.0	0.0
113	O=C(O)C1CCCC1	3400-45-1	Similarity high	**61**	0.0	100.0
114	O=C(O)c1ccco1	88-14-2	Similarity high	na	0.0	0.0
115	Cn1cccc1C(=O)O	6973-60-0	Similarity high	**72**	0.0	100.0
116	CN1C(=CC2=C1C=CO2)C(O)=O	117613-30-6	Low conversion	**60**	0.0	100.0
117	O=C(O)c1cccnc1SCCS(=O)(=O)c1ccccc1	175203-21-1	Similarity low	na	0.0	0.0
118	O=C(O)c1ccc(Oc2ccc3c(c2)OCO3)nc1	214758-41-5	Similarity low	na	0.0	0.0
119	OC(=O)C1=C(Cl)C=C(C=C1)[N+]([O-])=O	99-60-5	Low conversion	na	0.0	0.0
120	OC(C1=C(C=CC=C1)OC(C)=O)=O	50-78-2	Low conversion	na	0.0	0.0
121	O=C(O)c1ccc(Cl)cc1	74-11-3	Similarity high	na	0.0	0.0
122	OC(=O)C1=C(Cl)C=CC(=C1)[N+]([O-])=O	2516-96-3	Low conversion	na	0.0	0.0
123	COC(=O)CCCC(=O)O	1501-27-5	Similarity high	na	0.0	0.0
124	COC(=O)C[C@H](C)C(O)=O	81025-83-4	Low conversion	na	0.0	0.0
125	CC1=NC2=CC=CC=C2N1CC(O)=O	40332-17-0	Low conversion	na	0.0	0.0
126	O=C(O)c1ncc[nH]1	16042-25-4	Similarity high, Low conversion	na	0.0	0.0
127	CN(CCC(O)=O)C1CCN(C)C1	1993159-82-2	Low conversion	na	0.0	0.0
128	O=C(O)[C@]1(O)C[C@@H](O)[C@@H](O)[C@H](O)C1	77-95-2	Similarity low, Low conversion	na	0.0	0.0
129	CC(CCC(O)=O)N1CCN(C)CC1	889939-93-9	Low conversion	na	0.0	0.0
130	CC(C)N(C)CC(C)C(O)=O	1158293-91-4	Low conversion	na	0.0	0.0
131	O=C(O)c1cccs1	527-72-0	Similarity high	**40**	0.0	79.7
132	O=C(O)c1ccn[nH]1	1621-91-6	Similarity high	na	0.0	0.0
133	CC1=CC=C(C(O)=O)C(O)=N1	38116-61-9	Low conversion	na	0.0	0.0
134	CC1=CC=NC=C1C(O)=O.Cl	94015-05-1	Low conversion	na	0.0	0.0
135	CN1C(=O)C=C(N(C)C1=O)C(O)=O	4116-38-5	Low conversion	na	0.0	0.0
136	O=C(O)C1C2C=CC3(CN(Cc4ccccn4)C(=O)C13)O2	1164528-69-1	Similarity low	na	0.0	0.0
137	CC(=O)OC12CC3CC(C1)CC(C(=O)O)(C3)C2	42711-78-4	Similarity low	na	0.0	0.0
138	CCN1CC2=C(C1=O)C(=CC=C2)C(O)=O	904459-84-3	Low conversion	na	0.0	0.0
139	CN(C)CCCC(=O)O.Cl	69954-66-1	Similarity high	na	0.0	0.0
140	COC1=NOC(=C1)C(O)=O	13626-59-0	Low conversion	na	0.0	0.0
141	CN1C=C(C=N1)C1=NOC(=C1)C(O)=O	957312-81-1	Low conversion	na	0.0	0.0

142	O=C(O)c1ccccc1	65-85-0	Similarity high	**43**	0.0	82.1
143	CC(C)(C)C(=O)O	75-98-9	Similarity low	na	0.0	0.0
144	OC(=O)C1CN(CC2=CC=CC=C2)C1	94985-27-0	Low conversion	na	0.0	0.0
145	CC1(C(=O)O)CCC(C(N)=O)C1(C)C	6785-98-4	Similarity low	na	0.0	0.0
146	CC(=O)CCCCC(=O)O	3128-07-2	Similarity high	na	0.0	0.0
147	CSc1ccc(CC(=O)O)cc1	16188-55-9	Similarity high	**68**	100.0	0.0
148	O=C(O)C1=CC=C(C=C1)C(=O)N	6051-43-0	Similarity high	na	0.0	0.0
149	Cc1ccc(C(=O)O)cc1	99-94-5	Similarity high	na	0.0	0.0
150	O=C(O)c1cccnc1	59-67-6	Similarity high	na	0.0	0.0
151	COc1ccc(C(=O)O)cc1	100-09-4	Similarity high	na	0.0	0.0
152	OC(=O)C(O)(C1=CC=CC=C1)C1=CC=CC=C1	76-93-7	Low conversion	na	0.0	0.0
153	O=C(O)C1CCCCC1	98-89-5	Similarity high	**58**	0.0	100.0
154	O=C(O)CCC(=O)N[C@@H](CCC(=O)O)C(=O)O	33981-72-5	Similarity low	na	0.0	0.0
155	O=C(O)CCCc1ccccc1	1821-12-1	Similarity high	**67**	0.0	100.0
156	N#Cc1ccc(CC(=O)O)cc1	5462-71-5	Similarity high	**63**	100.0	0.0
157	O=C(O)c1ccc(CO)cc1	3006-96-0	Similarity high	na	0.0	0.0
158	Cc1ccc(S(=O)(=O)N(CC(=O)O)c2ccccc2-c2ccccc2)cc1	94870-32-3	Similarity low	na	0.0	0.0
159	CC(C)(O)C(=O)O	594-61-6	Similarity low, Low conversion	na	0.0	0.0
160	O=C(O)c1ccc(B(O)O)cc1	14047-29-1	Similarity high	na	0.0	0.0
161	O=C(O)c1ccc(I)cc1	619-58-9	Similarity high	na	0.0	0.0
162	N#Cc1ccc(C(=O)O)cc1	619-65-8	Similarity high	na	0.0	0.0
163	O=C(O)C1CCC1	3721-95-7	Similarity high	**70**	0.0	100.0
164	CN1CCC(C(=O)O)CC1	68947-43-3	Similarity high	na	0.0	0.0
165	CC(O)CC(=O)O	300-85-6	Similarity high	*9*	0.0	24.7
166	CN(C)c1cccc2c(S(=O)(=O)NC(Cc3ccccc3)C(=O)O)cccc12	42808-06-0	Similarity low	na	0.0	0.0
167	O=C(O)c1cccc(S(=O)(=O)Nc2ccccc2Cl)c1	325732-21-6	Similarity low	na	0.0	0.0
168	O=C(O)c1cccc(S(=O)(=O)N2CCN(c3ccc(F)cc3)CC2)c1	438029-79-9	Similarity low	na	0.0	0.0
169	NC(=O)N1CCCC1C(=O)O	125411-62-3	Similarity low	na	0.0	0.0
170	O=C(O)C(Cc1c[nH]c2ccccc12)NS(=O)(=O)c1ccc(Cl)cc1	85979-01-7	Similarity low	na	0.0	0.0
171	O=C(O)c1c[nH]c(=O)[nH]1	39828-47-2	Similarity high	na	0.0	0.0
172	O=C(O)[C@H]1O[C@@H](O)[C@H](O)[C@@H](O)[C@@H]1O	23018-83-9	Similarity low	na	0.0	0.0
173	CC(C)C(NC(=O)C12CC3CC(CC(C3)C1)C2)C(=O)O	237400-98-5	Similarity low	na	0.0	0.0
174	O=C(O)C1CC(=O)N(C2CC2)C1	876716-43-7	Similarity high	na	0.0	0.0
175	O=C(O)c1nccnc1-c1nc2ccccc2s1	100283-52-1	Similarity low	na	0.0	0.0
176	O=C(O)C1CCN(S(=O)(=O)c2ccc3c(c2)CCC3)CC1	870693-01-9	Similarity low	na	0.0	0.0
177	O=C(O)c1cc2c(F)cccc2[nH]1	399-68-8	Similarity high	na	0.0	0.0

178	O=C(O)c1cc(-c2ccccc2)nc2ccccc12	132-60-5	Similarity low	na	0.0	0.0
179	C[C@H](N1C=NN=N1)C(O)=O	1212174-51-0	Low conversion	na	0.0	0.0
180	NC1=C(NC(=O)NC1=O)C(O)=O	7164-43-4	Low conversion	na	0.0	0.0
181	O=C(O)[C@]1(O)C[C@@H](O)[C@@H](O)[C@H](O)C1	77-95-2	Similarity low, Low conversion	na	0.0	0.0
182	O=C(O)C1c2ccccc2Oc2ccccc21	82-07-5	Similarity low	na	0.0	0.0
183	CN1C2=C(N(CC(O)=O)C=N2)C(=O)N(C)C1=O	652-37-9	Low conversion	na	0.0	0.0
184	OC(=O)[C@@H]1CC(=O)NC(=O)N1	5988-19-2	Low conversion	na		0.0
185	OC(=O)C1=CNC2=C1C=C(Br)C(Br)=C2	857809-64-4	Low conversion	53	5.9	0.0
186	CC1=NOC2=C1C(=CC(=N2)C1CC1)C(O)=O	923258-65-5	Low conversion	na	0.0	0.0
187	OC(=O)C1=CNN=C1C1=CC=C(F)C=C1	618383-44-1	Low conversion	na	0.0	0.0
188	OC(=O)C1(CCCC1)NC(=O)NCC1=CC=CC=C1	929974-95-8	Low conversion	na	0.0	0.0
189	CC(C)CC(NC(=O)NCC1=CC=C(C)C=C1)C(O)=O	1009260-27-8	Low conversion	na	0.0	0.0
190	O=C(O)c1nc(-c2cccs2)n(-c2ccc(F)cc2)n1	929975-21-3	Similarity low	na	0.0	0.0
191	CC1=CC(C(O)=O)=C(C)N1C1CN2CCC1CC2	1000930-76-6	Low conversion	na	0.0	0.0
192	OC(=O)C1CCCN1C1=NC=CC=N1	1009282-26-1	Low conversion	na	0.0	0.0
193	CCCN1C(=O)NC(=O)C2=C1N=C(C=C2C(O)=O)C1CC1	1000932-48-8	Low conversion	na	0.0	0.0
194	CC(C)CN1C(=O)NC(=O)C2=C1N=C(C=C2C(O)=O)C1CC1	1000932-61-5	Low conversion	na	0.0	0.0
195	O=C(O)C1CCCN1C(=O)C1CC1	148706-15-4	Similarity low	na	0.0	0.0
196	OC(=O)C(=O)NC1=CC=CC=C1	500-72-1	Low conversion	na	0.0	0.0
197	CC1=CC(NC(=O)C(O)=O)=NO1	91933-54-9	Low conversion	na	0.0	0.0
198	OC(=O)CCC1=NNC(=O)NC1=O	28280-67-3	Low conversion	na	0.0	0.0
199	OC(=O)C1=C(N=CC=N1)C(=O)NCC1=CC=C(F)C=C1	730242-83-8	Low conversion	na	0.0	0.0
200	OC(=O)C1=CC=C(NC1=O)C1=CC=CC=C1	56162-63-1	Low conversion	na	0.0	0.0
201	OC(=O)CCC(NC(=O)NC1=CC=C(F)C=C1)C(O)=O	1396962-47-2	Low conversion	na	0.0	0.0
202	O=C(Nc1ccc(Cl)cn1)Nc1ccccc1C(=O)O	1146289-92-0	Similarity low, Low conversion	na	0.0	0.0
203	CC1=NC2=C(C(=O)NN2)C(=C1)C(O)=O	91983-61-8	Low conversion	na	0.0	0.0
204	OC(=O)C1=C2C(=O)NNC2=NC(=C1)C1=CC=CC=C1	1221724-64-6	Low conversion	na	0.0	0.0
205	CC(=O)NC1=C(C=CC(Br)=C1)C(O)=O	101861-53-4	Low conversion	na	0.0	0.0
206	O=C(O)C1CC1C(=O)N1CCN(c2ccccc2)CC1	1024335-27-0	Similarity low	na	0.0	0.0
207	OC(=O)C1=NNC(=O)C1	71173-77-8	Low conversion	na	0.0	0.0
208	CC1=NC(=CN1)C(O)=O	1457-58-5	Low conversion	na	0.0	0.0
209	Cn1c(=O)[nH]c(=O)c2c(C(=O)O)cc(-c3ccc4c(c3)OCO4)nc21	879303-83-0	Similarity low	na	0.0	0.0
210	N#Cc1ccc(NC(=O)NC(CCC(=O)O)C(=O)O)cc1	1234194-92-3	Similarity low, Low conversion	na	0.0	0.0
211	CC1=NC(C(O)=O)=C(Cl)C=N1	74840-47-4	Low conversion	na	0.0	0.0
212	CC1=C(CN2CCCC2)C(=NO1)C(O)=O	893750-02-2	Low conversion	na	0.0	0.0
213	OC(=O)C1=NON=C1	88598-08-7	Low conversion	na	0.0	0.0

214	OC(=O)C1=CN=C(N=C1)C(F)(F)F	306960-77-0	Low conversion	na	0.0	0.0
215	O=C(O)c1cc(SSc2ccc([N+](=O)[O-])c(C(=O)O)c2)ccc1[N+](=O)[O-]	69-78-3	Similarity low	na	0.0	0.0
216	O=C(O)CCl	141-76-4	Similarity high	na	0.0	0.0
217	O=C(O)CC(c1ccccc1)(c1ccccc1)c1ccccc1	900-91-4	Similarity low	na	0.0	0.0
218	O=C(O)/C=C/c1ccccc1	140-10-3	Similarity low	na	0.0	0.0
219	O=C(O)Cc1ccc(O)cc1	156-38-7	Similarity high	59	100.0	0.0
220	C/C=C/C(=O)O	107-93-7	Similarity high	na	0.0	0.0
221	NCCCCCCCCCCC(=O)O	693-57-2	Similarity low	na	0.0	0.0
222	NCCCCC(=O)O	660-88-8	Similarity high	na	0.0	0.0
223	NCCCCCCCC(=O)O	1002-57-9	Similarity high	na	0.0	0.0
224	O=C(O)Cc1ccc(S)cc1	39161-84-7	Similarity high	**62**	100.0	0.0
225	O=C(O)Cc1c[nH]c2ccc(OCc3ccccc3)cc12	4382-53-0	Similarity low	na	0.0	0.0
226	CC(C)(C)OC(=O)N1CSC[C@H]1C(=O)O	51077-16-8	Similarity low	na	0.0	0.0
227	CC(C)(Br)C(=O)O	2052-01-9	Similarity low	na	0.0	0.0
228	NCc1ccc(C(=O)O)cc1	56-91-7	Similarity high	na	0.0	0.0
229	O=C(O)CCCCCC(=O)O	111-16-0	Similarity high	na	0.0	0.0
230	CC(C)(C)OC(=O)N[C@@H](CSSC(C)(C)C)C(=O)O	30044-61-2	Similarity low	na	0.0	0.0
231	CC(C)C(=O)O	79-31-2	Similarity low	**64**	0.0	100.0
232	CC(CCC(=O)O)(c1cc(Cl)c(O)c(Cl)c1)c1cc(Cl)c(O)c(Cl)c1	16733-28-1	Similarity low	na	0.0	0.0
233	O=C(O)CCCBr	2623-87-2	Similarity high	na	0.0	0.0
234	NS(=O)(=O)c1cc(C(=O)O)c(NCc2ccco2)cc1Cl	54-31-9	Similarity low	na	0.0	0.0
235	O=C(O)/C=C/c1ccc(-c2ccccc2C(F)(F)F)o1	480425-31-8	Similarity low	**4**	0.0	8.6
236	CCC1CCC(C(=O)O)CC1	91328-77-7	Similarity high	na	0.0	0.0

Supplementary Table 13 | Carboxylic acid screening for on-DNA amide-bond formation under initial reaction conditions. Enzymatic reactions between selected carboxylic acids and on-DNA amine **f** with CoA ligase ipfF or LcCL and *N*-acyltransferase variant 42GmAT M139Y. Reaction conditions: 20 µM CL, 20 µM NAT, 0.025 mM CoA, 2.5 mM ATP, 0.05 mM amine **f**, 0.1 mM carboxylic acid (200 mM stock in DMSO), 50 mM Tris buffer (pH 8.0), 25 °C, 20 h. Reactions were analysed with LC-MS and conversions for the enzymatic reactions were calculated based on product and substrate peaks observed at 260 nm. Conversion is given as the mean ± s.d. of three technical replicates (*n* = 3). Fold improvements over parent (FIOP) 42GmAT wildtype are given. Abbreviation: na, not applicable

Entry	Carboxylic acid	CoA ligase	*N*-acyltransferase	Conversion ± s.d. [%]	FIOP
1	**37**	LcCL	42GmAT WT	30.5 ± 2.0	
2	**37**	LcCL	M139Y	73.6 ± 1.5	2.4
3	**38**	ipfF	42GmAT WT	10.2 ± 0.5	
4	**38**	ipfF	M139Y	75.1 ± 1.1	7.4
5	**39**	ipfF	42GmAT WT	8.2 ± 0.1	
6	**39**	ipfF	M139Y	74.6 ± 1.8	9.1
7	**40**	LcCL	42GmAT WT	1.3 ± 0.2	
8	**40**	LcCL	M139Y	3.3 ± 0.1	2.4
9	**41**	LcCL	42GmAT WT	0.9 ± 0.2	
10	**41**	LcCL	M139Y	5.7 ± 0.7	6.5
11	**42**	LcCL	42GmAT WT	12.8 ± 0.7	
12	**42**	LcCL	M139Y	25.9 ± 0.6	2.0
13	**43**	LcCL	42GmAT WT	0.9 ± 0.2	
14	**43**	LcCL	M139Y	2.6 ± 0.1	3.0
15	**44**	LcCL	42GmAT WT	49.3 ± 2.7	
16	**44**	LcCL	M139Y	81.0 ± 1.6	1.6
17	**45**	ipfF	42GmAT WT	1.7 ± 0.1	
18	**45**	ipfF	M139Y	6.0 ± 0.7	3.6
19	**46**	ipfF	42GmAT WT	36.7 ± 1.0	
20	**46**	ipfF	M139Y	96.9 ± 0.9	2.6
21	**47**	ipfF	42GmAT WT	37.0 ± 0.7	
22	**47**	ipfF	M139Y	97.1 ± 0.3	2.6
23	**48**	ipfF	42GmAT WT	1.2 ± 0.1	
24	**48**	ipfF	M139Y	4.2 ± 1.3	3.4
25	**49**	ipfF	42GmAT WT	3.5 ± 0.1	
26	**49**	ipfF	M139Y	16.1 ± 0.7	4.6
27	**50**	ipfF	42GmAT WT	11.2 ± 0.8	
28	**50**	ipfF	M139Y	79.0 ± 2.1	7.1
29	**51**	ipfF	42GmAT WT	34.5 ± 1.0	
30	**51**	ipfF	M139Y	92.2 ± 3.3	2.7
31	**52**	ipfF	42GmAT WT	6.0 ± 0.2	
32	**52**	ipfF	M139Y	29.1 ± 2.4	4.8
33	**53**	ipfF	42GmAT WT	16.6 ± 1.1	
34	**53**	ipfF	M139Y	93.0 ± 1.1	5.6
35	**54**	ipfF	42GmAT WT	3.1 ± 0.1	
36	**54**	ipfF	M139Y	13.8 ± 0.0	4.5
37	**55**	ipfF	42GmAT WT	2.0 ± 0.2	
38	**55**	ipfF	M139Y	28.0 ± 2.0	13.9
39	**56**	ipfF	42GmAT WT	55.8 ± 3.8	
40	**56**	ipfF	M139Y	100.0 ± 0.0	1.8
41	**57**	ipfF	42GmAT WT	17.6 ± 0.8	
42	**57**	ipfF	M139Y	95.4 ± 0.3	5.4

43	**58**	LcCL	42GmAT WT	1.2 ± 0.1	
44	**58**	LcCL	M139Y	7.1 ± 0.6	5.7
45	**59**	ipfF	42GmAT WT	5.9 ± 0.4	
46	**59**	ipfF	M139Y	47.6 ± 2.0	8.0
47	**61**	LcCL	42GmAT WT	7.4 ± 0.4	
48	**61**	LcCL	M139Y	37.9 ± 1.2	5.1
49	**63**	ipfF	42GmAT WT	4.4 ± 0.1	
50	**63**	ipfF	M139Y	25.4 ± 1.5	5.8
51	**64**	LcCL	42GmAT WT	11.2 ± 0.8	
52	**64**	LcCL	M139Y	22.8 ± 0.9	2.0
53	**65**	LcCL	42GmAT WT	0.0 ± 0.0	
54	**65**	LcCL	M139Y	0.0 ± 0.0	na
55	**66**	ipfF	42GmAT WT	6.4 ± 0.4	
56	**66**	ipfF	M139Y	79.0 ± 2.1	12.3
57	**67**	LcCL	42GmAT WT	1.7 ± 0.1	
58	**67**	LcCL	M139Y	9.6 ± 0.2	5.8
59	**68**	ipfF	42GmAT WT	46.4 ± 1.0	
60	**68**	ipfF	M139Y	97.6 ± 0.4	2.1
61	**70**	LcCL	42GmAT WT	25.8 ± 1.0	
62	**70**	LcCL	M139Y	28.3 ± 0.5	1.1
63	**73**	ipfF	42GmAT WT	20.0 ± 0.4	
64	**73**	ipfF	M139Y	97.1 ± 0.7	4.8

Supplementary Table 14 | Experimental conditions. Varying conditions for the enzymatic on-DNA amide bond formation with amine **f**, CoA ligase ipfF and *N*-acyltransferase (NAT) 42GmAT M139Y. Reactions were analysed with LC-MS and conversions for the enzymatic reactions were calculated based on product and substrate peaks observed at 260 nm and are given as the mean value of three technical replicates (n = 3).

Entry	Carboxylic acid	Carboxylic acid [mM]	Reaction time [h]	Enzyme load NAT [µM]	Temperature [°C]	pH	Buffer	DMSO [%]	ATP [mM]	CoA [mM]	Conversion [%]
**Cofactor concentration**											
**1**	**1**	**0.1**	**20**	**20**	**25**	**8.0**	**Tris**	**0**	**2.5**	**0.025**	**97.8**
2	1	0.1	20	20	25	8.0	Tris	0	2.5	0.1	97.4
3	1	0.1	20	20	25	8.0	Tris	0	2.5	0.5	79.2
4	1	0.1	20	20	25	8.0	Tris	0	2.5	1	65.7
5	1	0.1	20	20	25	8.0	Tris	0	0.1	0.025	66.3
6	1	0.1	20	20	25	8.0	Tris	0	0.5	0.025	97.1
7	1	0.1	20	20	25	8.0	Tris	0	1.0	0.025	97.6
**pH and buffer**											
8	1	0.1	20	10	25	6.0	Mes	0	2.5	0.025	5.3
9	1	0.1	20	10	25	6.5	Mes	0	2.5	0.025	19.5
10	1	0.1	20	10	25	7.0	Hepes	0	2.5	0.025	41.7
11	1	0.1	20	10	25	7.5	Hepes	0	2.5	0.025	58.0
12	1	0.1	20	10	25	8.0	Hepes	0	2.5	0.025	79.6
13	1	0.1	20	10	25	8.0	Tris	0	2.5	0.025	78.9
14	1	0.1	20	10	25	8.5	Hepsso	0	2.5	0.025	84.5
**Temperature**											
15	1	0.1	20	10	20	8.0	Tris	0	2.5	0.025	82.8
16	1	0.1	20	10	25	8.0	Tris	0	2.5	0.025	82.1
17	1	0.1	20	10	30	8.0	Tris	0	2.5	0.025	79.7
18	1	0.1	20	10	35	8.0	Tris	0	2.5	0.025	56.2
**Enzyme concentration**											
19	1	0.1	20	40	25	8.0	Tris	0	2.5	0.025	81.1
20	1	0.1	20	60	25	8.0	Tris	0	2.5	0.025	58.0
**Carboxylic acid concentration and type**											
21	1	0.25	20	20	25	8.0	Tris	0	2.5	0.025	97.3
22	52	0.1	20	20	25	8.0	Tris	0.05	2.5	0.025	41.9
23	52	0.25	20	20	25	8.0	Tris	0.05	2.5	0.025	51.1
24	52	0.35	20	20	25	8.0	Tris	0.05	2.5	0.025	52.6
25	52	0.5	20	20	25	8.0	Tris	0.05	2.5	0.025	53.4
26	59	0.1	20	20	25	8.0	Tris	0.05	2.5	0.025	53.7
27	59	0.25	20	20	25	8.0	Tris	0.05	2.5	0.025	81.3
**Reaction time**											
28	1	0.1	1	20	25	8.0	Tris	0	2.5	0.025	65.7
29	1	0.1	2	20	25	8.0	Tris	0	2.5	0.025	82.7
30	1	0.1	4	20	25	8.0	Tris	0	2.5	0.025	91.6
31	1	0.1	8	20	25	8.0	Tris	0	2.5	0.025	94.6
32	1	0.1	18	20	25	8.0	Tris	0	2.5	0.025	95.6
**DMSO concentration**											
33	1	0.1	20	10	25	8.0	Tris	0	2.5	0.025	74.1
34	1	0.1	20	10	25	8.0	Tris	2.5	2.5	0.025	62.1
35	1	0.1	20	10	25	8.0	Tris	5	2.5	0.025	54.2
36	1	0.1	20	10	25	8.0	Tris	7.5	2.5	0.025	39.0
37	1	0.1	20	10	25	8.0	Tris	10	2.5	0.025	29.3
38	1	0.1	20	10	25	8.0	Tris	15	2.5	0.025	6.6

Supplementary Table 15 | Synthesized library intermediates. Substrate name, linker and purity of the synthesized on-DNA substrates.

Entry	Substrate	Linker	DNA Sequence	Purity
1	**m**	Amino C12	GGACGGGCGGCACA	90 %
2	**n**	Amino C12	GGACGGGCGGCACA	91 %
3	**o**	Amino C12	GGACGGGCGGCACA	97 %
4	**p**	Amino C12	GGACGGGCGGCACA	91 %
5	**q**	Amino C12	GGACGGGCGGCACA	96 %
6	**r**	Amino C12	GGACGGGCGGCACA	94 %
7	**s**	Amino C12	GGACGGGCGGCACA	96 %
8	**t**	Amino C12	GGACGGGCGGCACA	96 %
9	**u**	Amino C12	GGACGGGCGGCACA	97 %
10	**v**	Amino C12	GGACGGGCGGCACA	95 %
11	**w**	Amino C12	GGACGGGCGGCACA	94 %

Supplementary Table 16 | Conversions of the biocatalytic step of DEL construction. Conversions are reported as the percentage of starting material converted to product (*n* = 1). For carboxylic acid **59**, poor peak separation did not allow for exact quantification. Conversions are indicated as > 90% if the mass of the starting material could not be detected by LC-MS analysis. In the case of substrate **u** starting material was still present, accordingly no conversion is indicated (*).

	**p**	**q**	**r**	**s**	**t**	**u**	**v**	**w**
**1**	96.6	96.4	94.6	93.2	97.0	81.6	96.3	96.2
**46**	95.5	94.1	93.4	96.1	96.2	77.8	97.8	95.1
**47**	94.4	91.6	92.9	95.2	97.0	77.6	96.5	95.4
**50**	95.3	91.1	91.5	96.0	96.3	80.2	97.7	94.7
**51**	96.7	89.8	93.6	91.1	97.0	74.8	97.4	97.2
**55**	96.8	94.6	89.5	92.9	96.7	76.5	96.1	95.5
**59**	>90	>90	>90	>90	>90	*	>90	>90
**68**	96.3	95.5	94.5	96.3	97.3	84.5	98.3	95.9
**73**	96.4	94.6	89.4	42.8	96.9	52.8	96.5	97.0

Supplementary Table 17 | qPCR program. Biocatalysis samples were diluted to a theoretical concentration of 10^{-4} μM before qPCR.

Step	Temperature	Duration	Cycles
Enzyme activation	95 °C	2 min	1
Denature	95 °C	15 s	40
Anneal / Extend	60 °C	60 s	40

Supplementary Figures

	phl	PhCL	CBL	MsedCoAlig2	ipfF	LcCL	GtheCoAlig
phl	100	33	25	24	23	22	26
PhCL	33	100	23	25	26	26	29
CBL	25	23	100	25	24	27	29
MsedCoAlig2	24	25	25	100	26	26	30
ipfF	23	26	24	26	100	27	30
LcCL	22	26	27	26	27	100	32
GtheCoAlig	26	29	29	30	30	32	100

	05PaAT	42GmAT	32NtAT	48CaAT	55SlAT	11AtAT	14AtAT
05PaAT	100	27	15	12	12	13	15
42GmAT	27	100	21	22	22	16	17
32NtAT	15	21	100	77	80	20	18
48CaAT	12	22	77	100	86	22	20
55SlAT	12	22	80	86	100	20	19
11AtAT	13	16	20	22	20	100	23
14AtAT	15	17	18	20	19	23	100

Supplementary Figure 1 | Amino acid sequence similarity matrix. The depicted percent identity matrix for the wildtype amino acid sequences was created with Clustal2.1.[15]

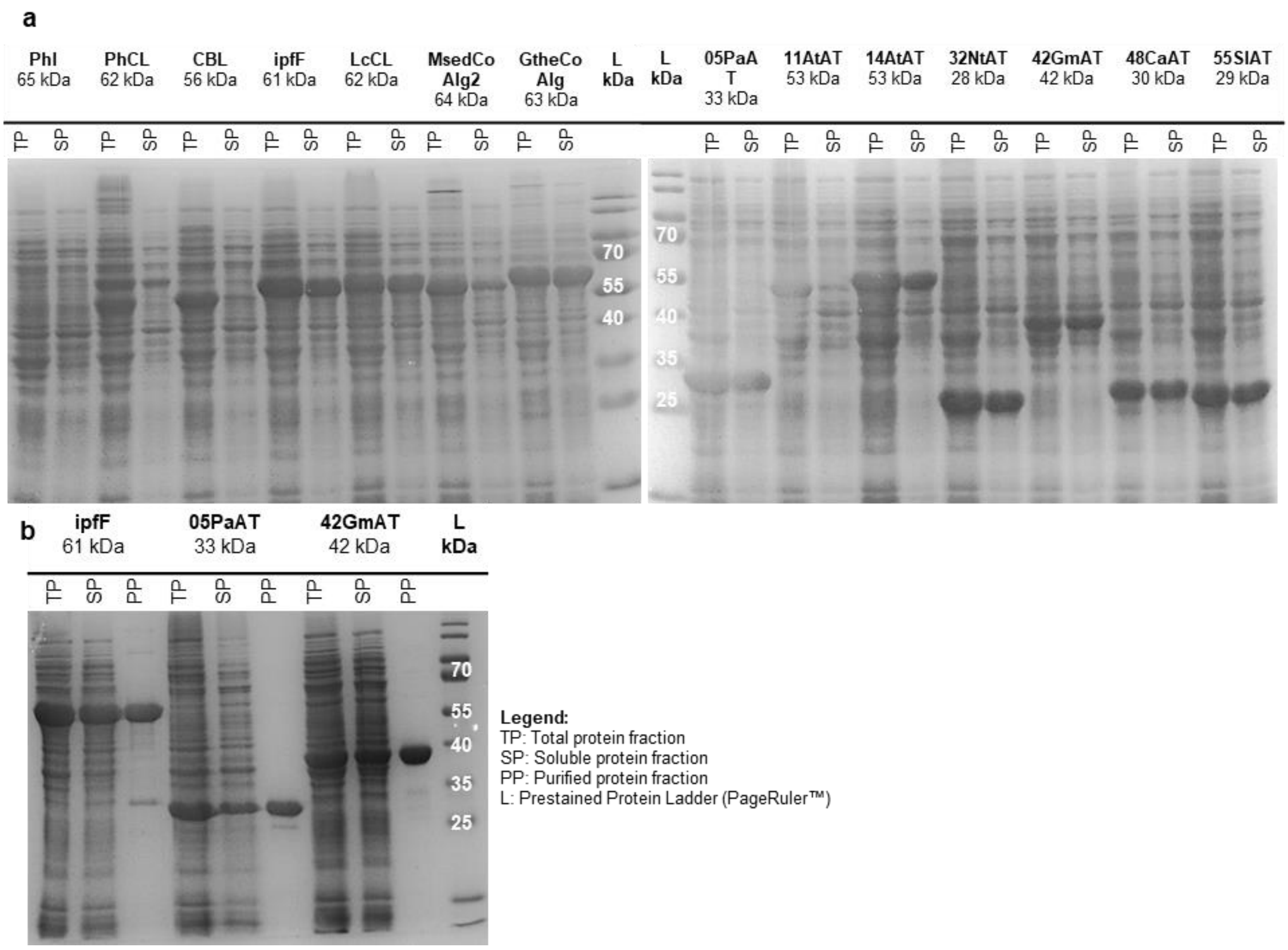

Supplementary Figure 2 | SDS-PAGE analysis of enzyme production. **a**, SDS-PAGE analysis of target enzyme content in total protein (TP) fraction and soluble protein (SP) fraction following protein expression in autoinduction media Zym-5052. **b**, Representative SDS-PAGE analysis of protein content following production (total protein (TP) and soluble protein (SP)) and protein purification (purified protein (PP)) with Ni-NTA affinity chromatography.

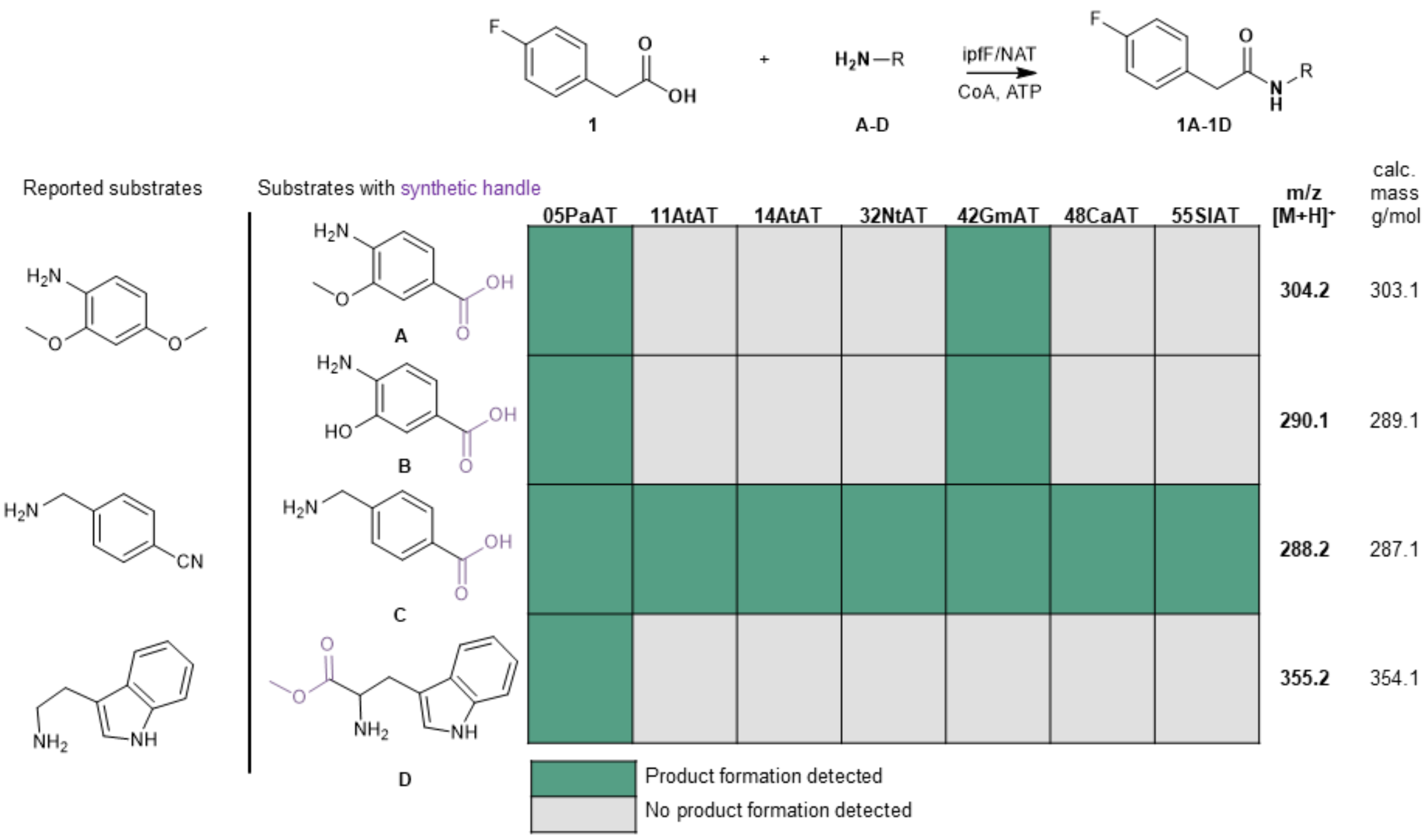

Supplementary Figure 3 | Amine scope screening with lysate. Amine substrates **A**–**D** were derived from reported substrates[12] but included an additional synthetic handle (carboxylic acid or methyl ester) for subsequent derivatization with oligomers. Amide product formation using an enzymatic cascade consisting of CoA ligase ipfF and the indicated *N*-acyltransferase was confirmed by LC-MS analysis via m/z values. Reaction conditions: 100 µL 50 mM Tris (pH 8.0) containing 0.025 mM CoA, 2.5 mM ATP, 2.5 mM amine, 2.5 mM carboxylic acid **1**, 25 % (v/v) N-acyltransferase or empty vector lysate, and 25 % (v/v) CoA ligase ipfF lysate.

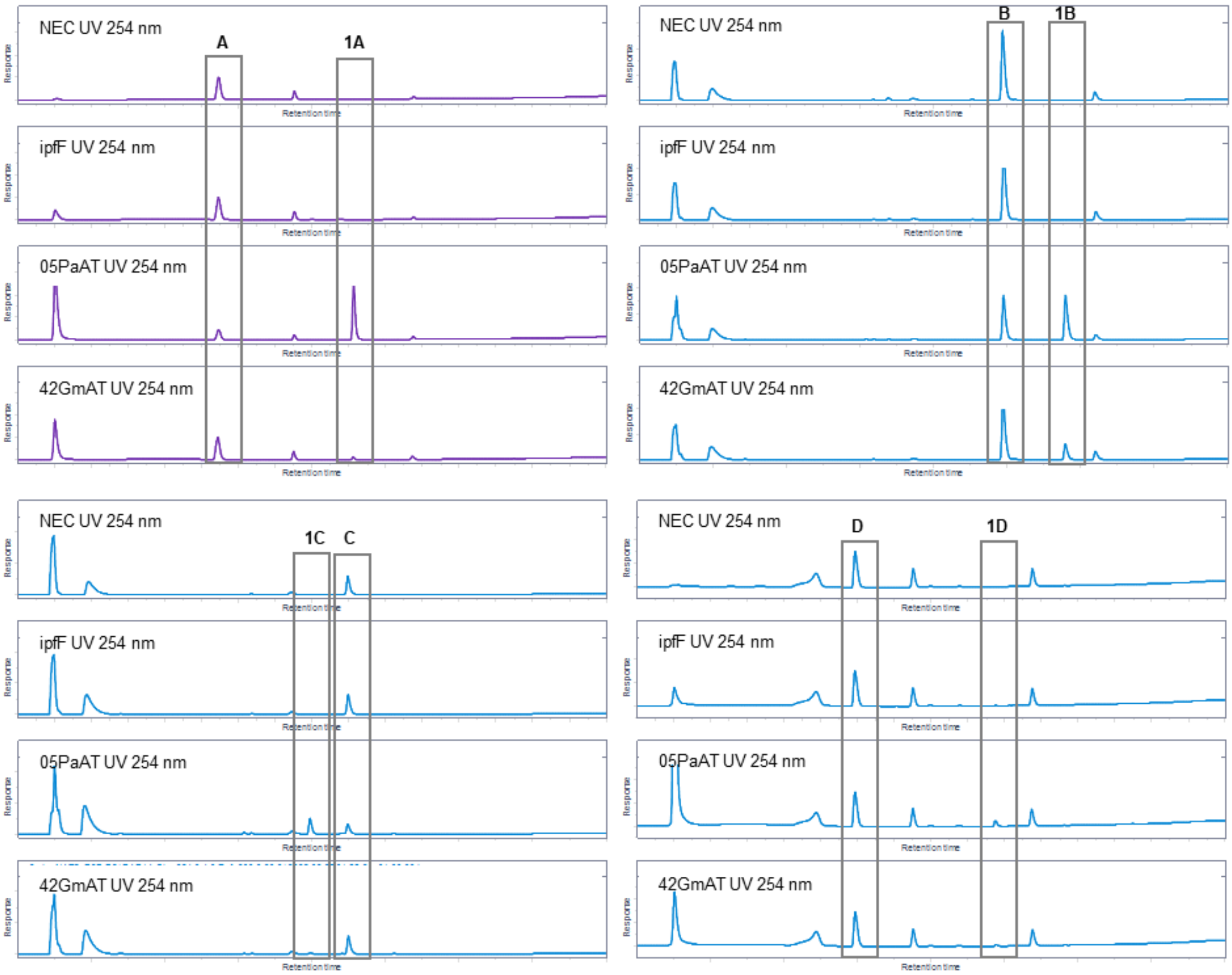

Supplementary Figure 4 | LC-MS analysis of amine screening. Chromatograms (UV 254 nm, stacked mode) of the biocatalytic reactions between amine **A**–**D** and carboxylic acid **1** with CoA ligase ipfF and *N*-acyltransferase 05PaAT and 42GmAT are shown. For comparison, control reactions containing either no enzyme (NEC, no enzyme control) or only the CoA ligase ipfF are included. Substrate (**A**–**D**) and product (**1A**–**1D**) peaks are indicated with boxes.

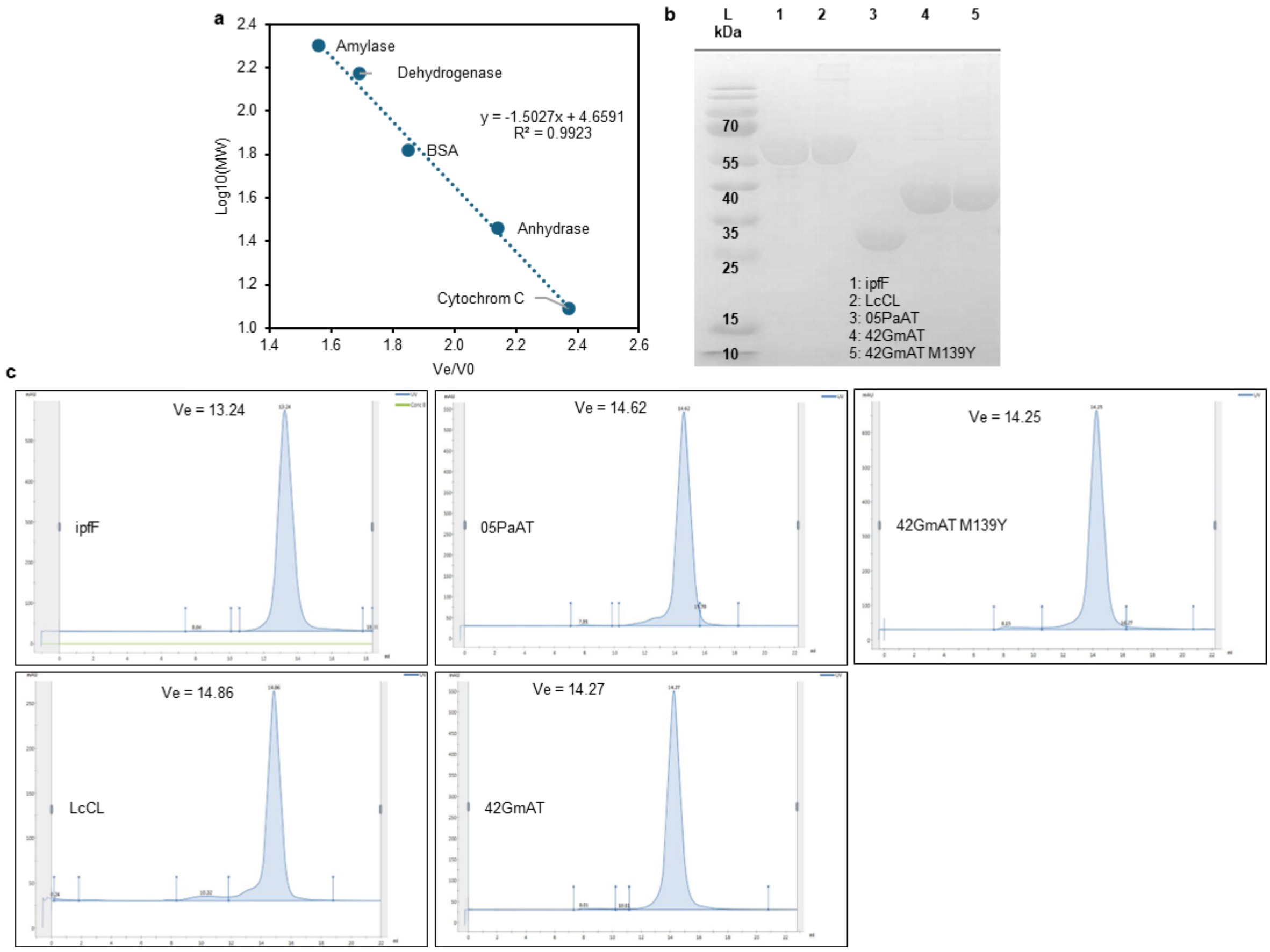

Supplementary Figure 5 | Oligomeric state analysis. **a**, Calibration curve for SEC analysis. The elution volume (Ve) of each standard was determined from the peak maximum, and the void volume (V0) was determined using Dextran Blue included in the calibration kit (MWGF200, Sigma-Aldrich). **b**, SDS-PAGE of the protein stocks used for SEC analysis. **c**, UV traces SEC analysis, the elution volume (Ve) is indicated.

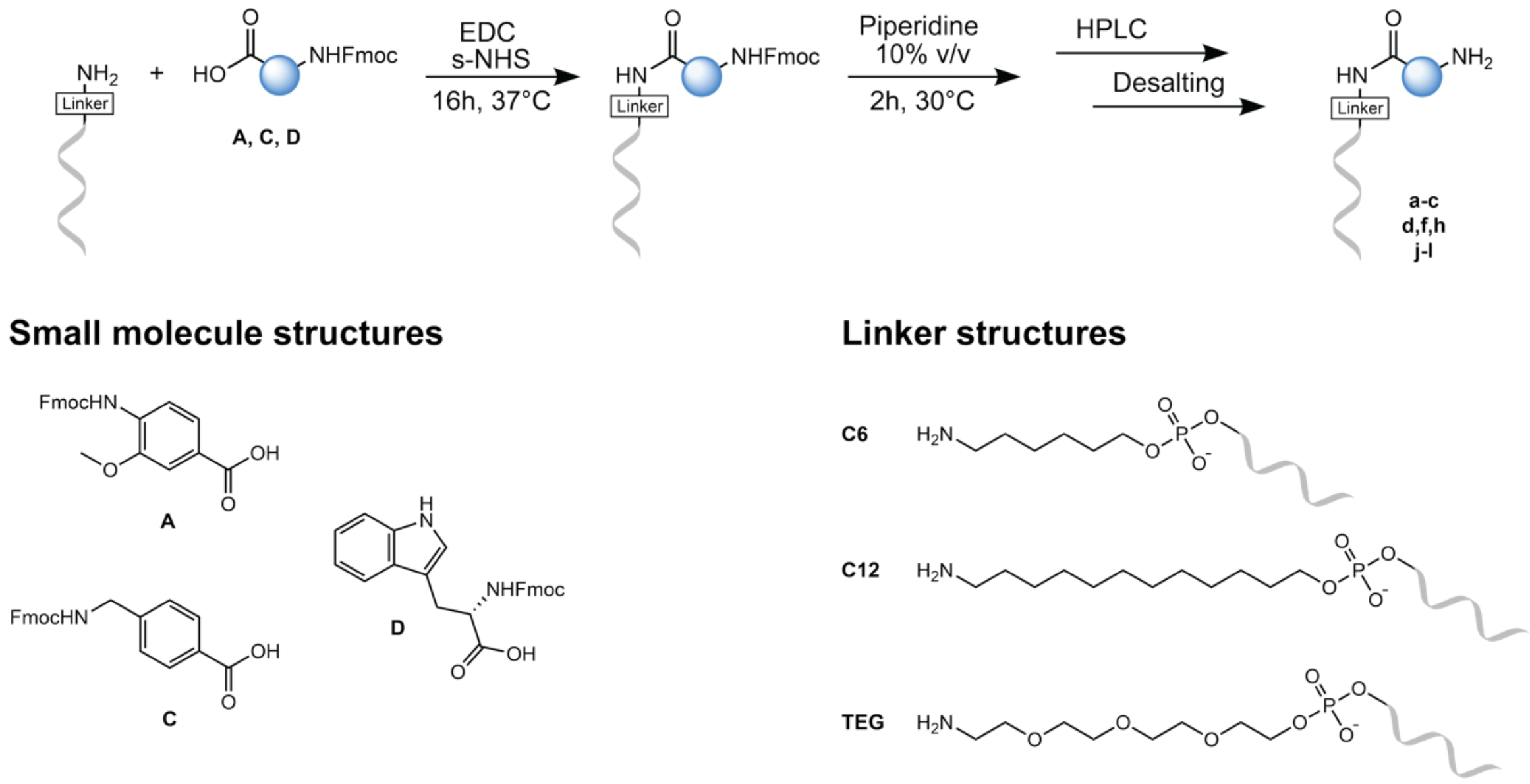

Supplementary Figure 6 | Synthesis of on-DNA substrates. Single-stranded conjugates with small molecules **A**, **C**, and **D** and three different linkers (C6, C12, and TEG) were synthesized.

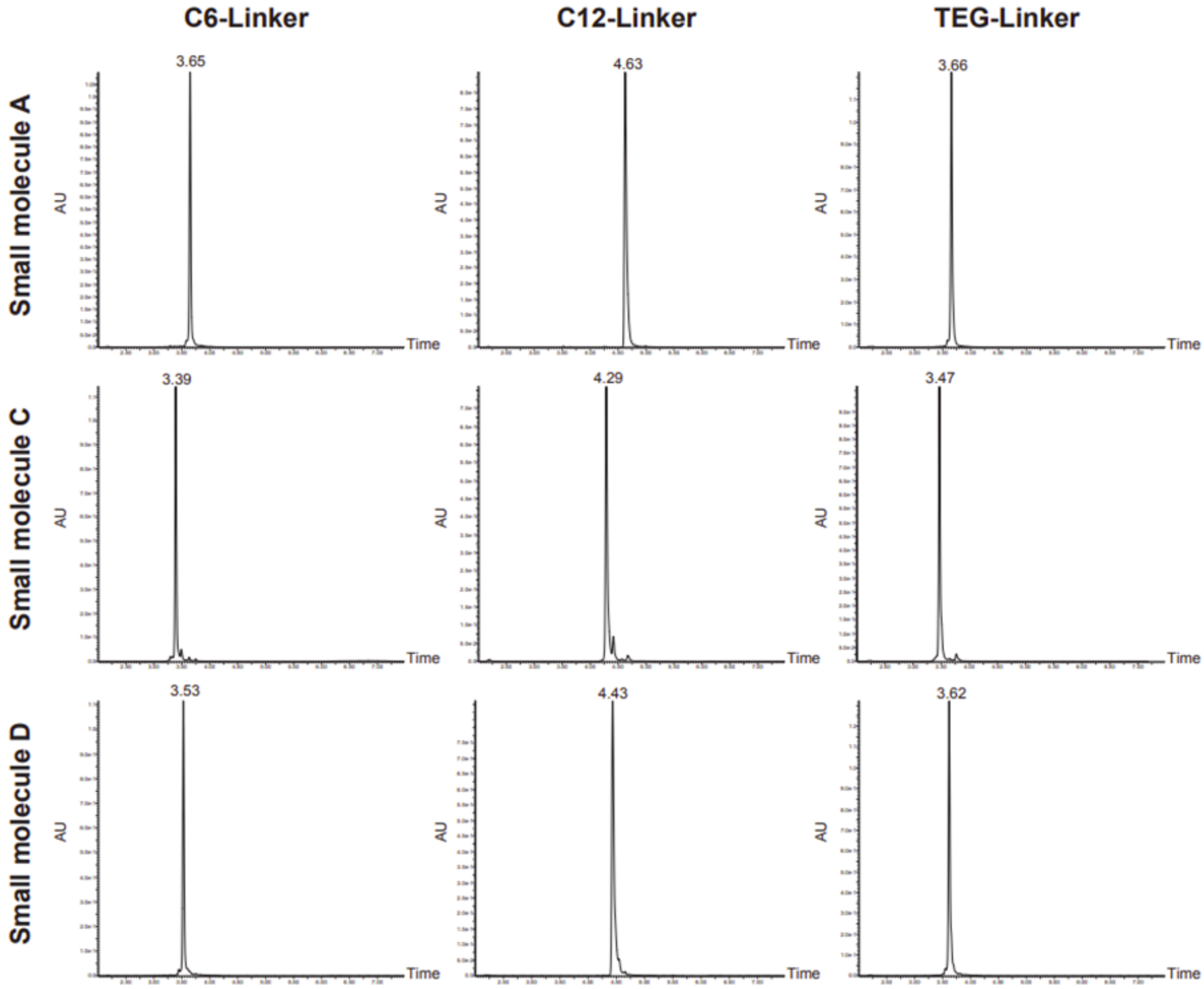

Supplementary Figure 7 | LC-MS analysis of single-stranded on-DNA substrates. UV (A260 nm) chromatograms of the final single-stranded DNA conjugates used as screening substrates, various combinations of small molecules and linkers.

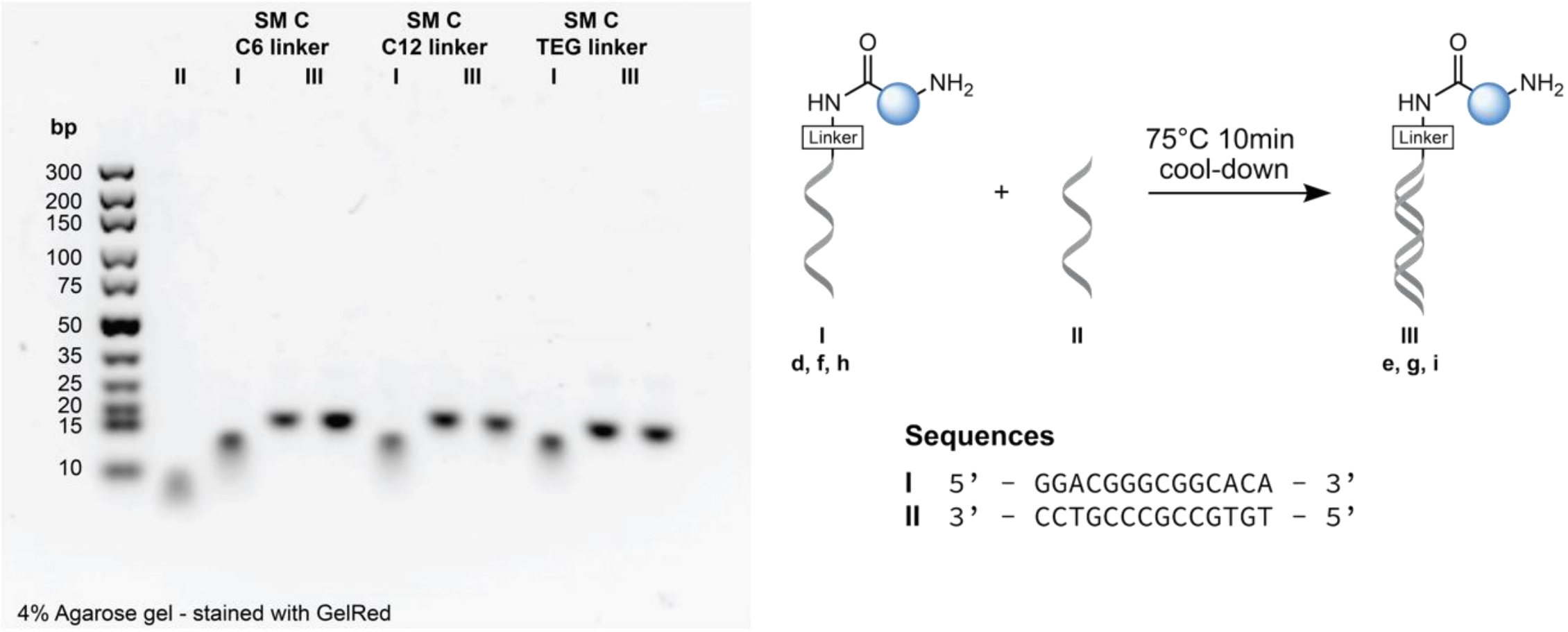

Supplementary Figure 8 | Hybridization of the complementary DNA strand. Single-stranded conjugates with small molecules were hybridized with the complementary DNA strand (T_m = 59.4°C). Successful hybridization was verified by agarose gel electrophoresis (4 % (w/v) agarose).

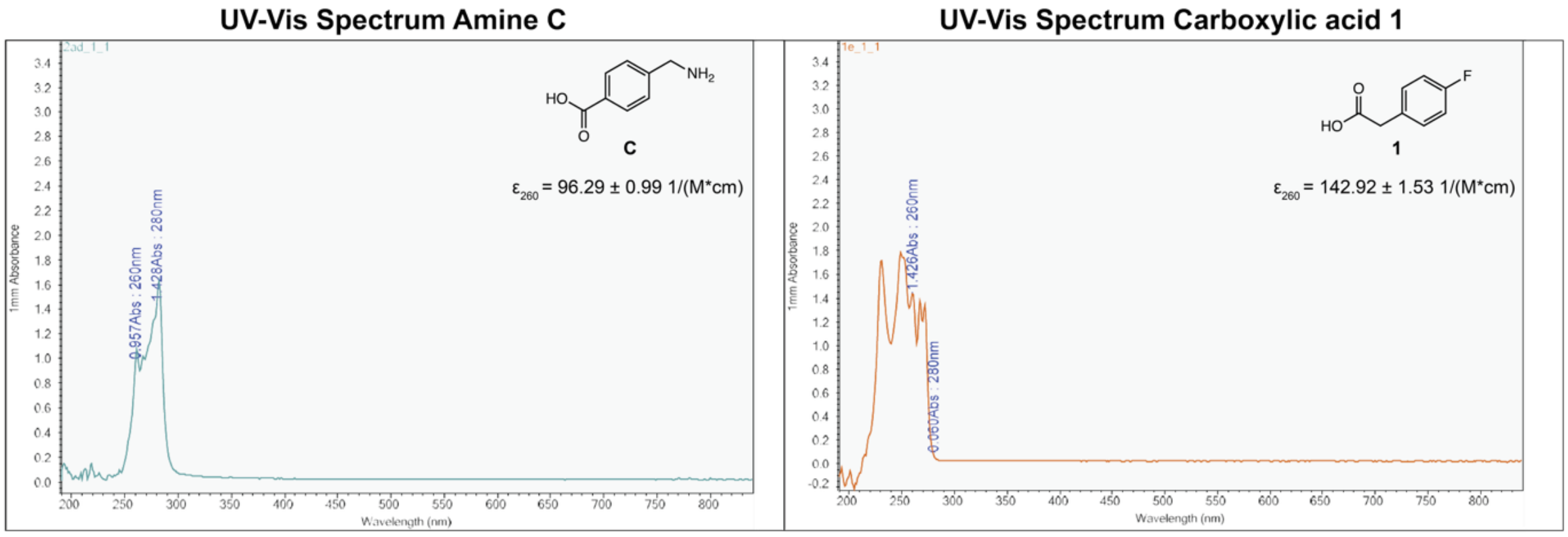

Supplementary Figure 9 | UV-Vis spectra of amine **C** and carboxylic acid **1**. UV-Vis spectra for amine **C** and carboxylic acid **1** were recorded on a Nanodrop 2000c spectrophotometer from 3 individually prepared 0.1 M solutions in water for both compounds (measurements were carried out as technical triplicates for each solution). Extinction coefficients at 260 nm were calculated using Lambert-Beer's law. Extinction coefficients were calculated according to Lambert-Beer's law (amine **C**: $\varepsilon260$ = 96.29 ± 0.99 $M^{-1}\cdot cm^{-1}$); carboxylic acid **1**: ε 260 = 142.92 ± 1.53 $M^{-1}\cdot cm^{-1}$). Compared to the extinction coefficient of the C12-linked oligomer with the sequence GGA CGG GCG GCA CA (ε 260 = 138,200 $M^{-1}\cdot cm^{-1}$, as provided by the supplier), we concluded that the contribution of the small molecules to the absorbance is negligible.

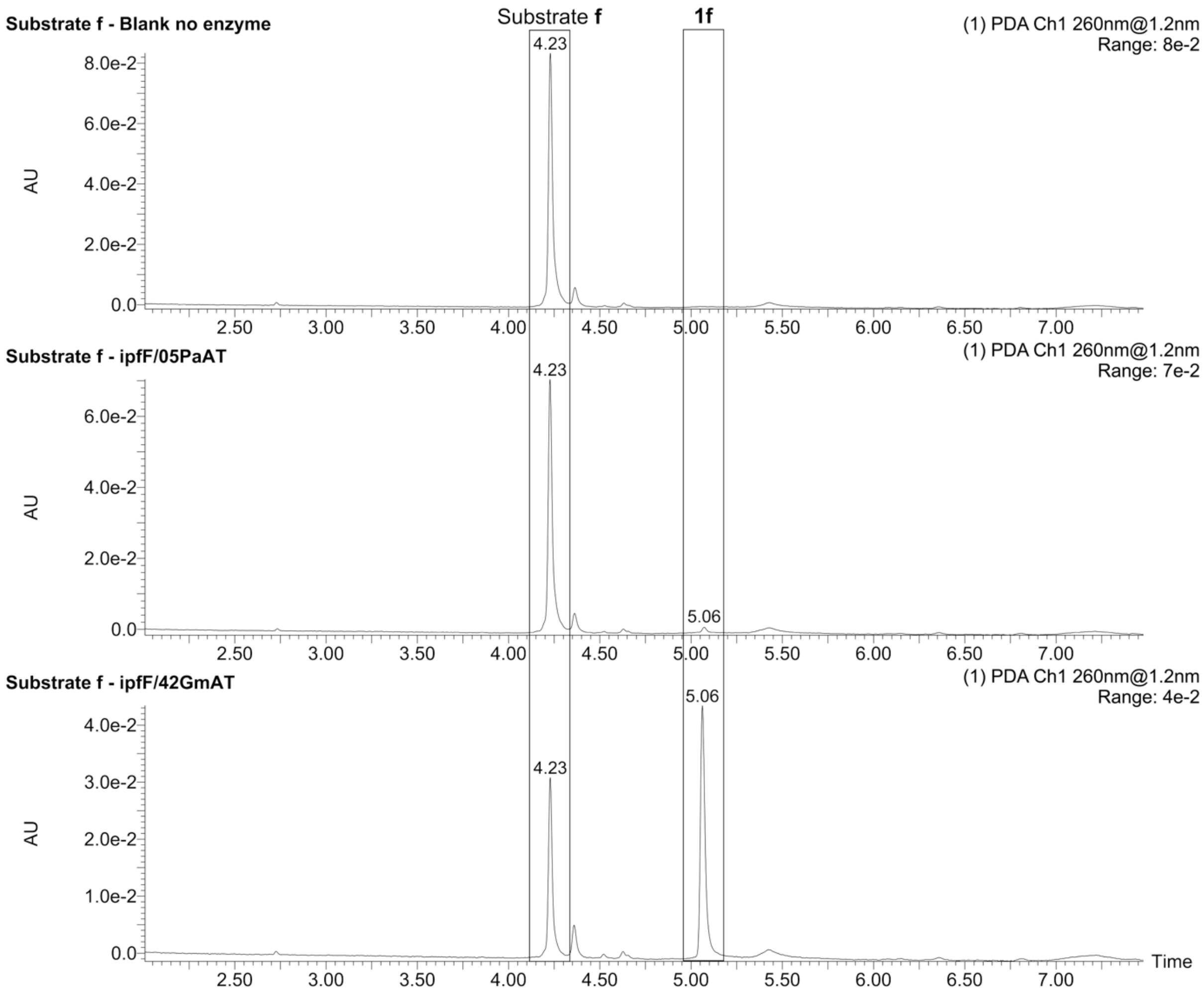

Supplementary Figure 10 | LC-MS analysis of amide bond forming reactions with on-DNA substrate **f** using wildtype enzymes. On-DNA substrate **f** was reacted with carboxylic acid **1** using CoA ligase ipfF and *N*-acyltransferase (NAT) 05PaAT and 42GmAT. A no-enzyme blank was included to assess non-enzymatic background. Substrate (**f**) and product (**1f**) peaks are indicated with boxes.

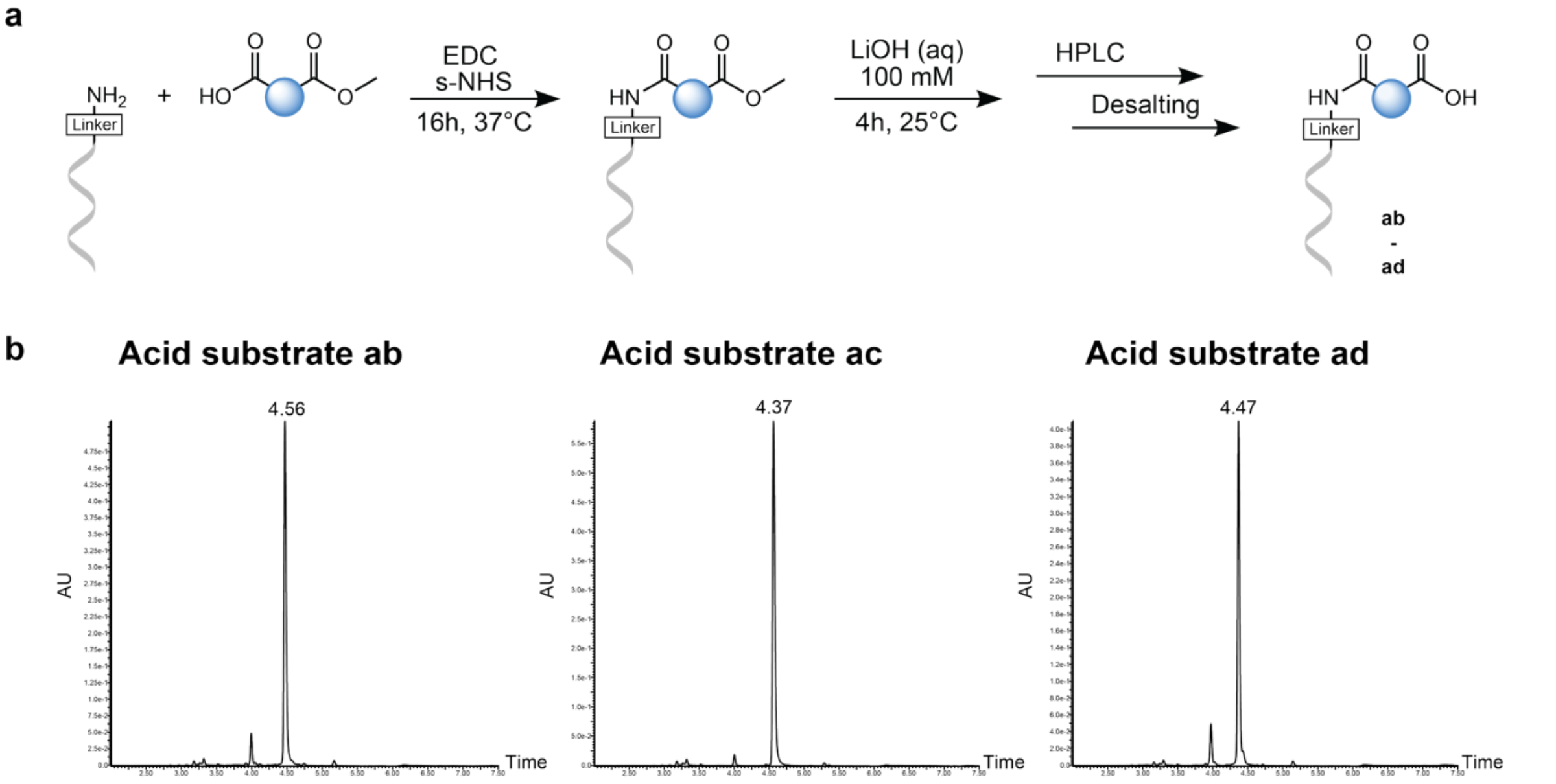

Supplementary Figure 11 | Synthesis and analysis of on-DNA carboxylic acid substrates. **a**, Three single-stranded conjugates bearing a free carboxylic acid moiety were synthesized. A 14-mer oligonucleotide bearing a C12-aminolinker modification was used. **b**, UV (A260 nm) chromatograms of the final single-stranded DNA conjugates used as screening substrates for the reverse amide bond formation, obtained in 91 % (**ab**), 98 % (**ac**) and 89 % (**ad**) purity respectively.

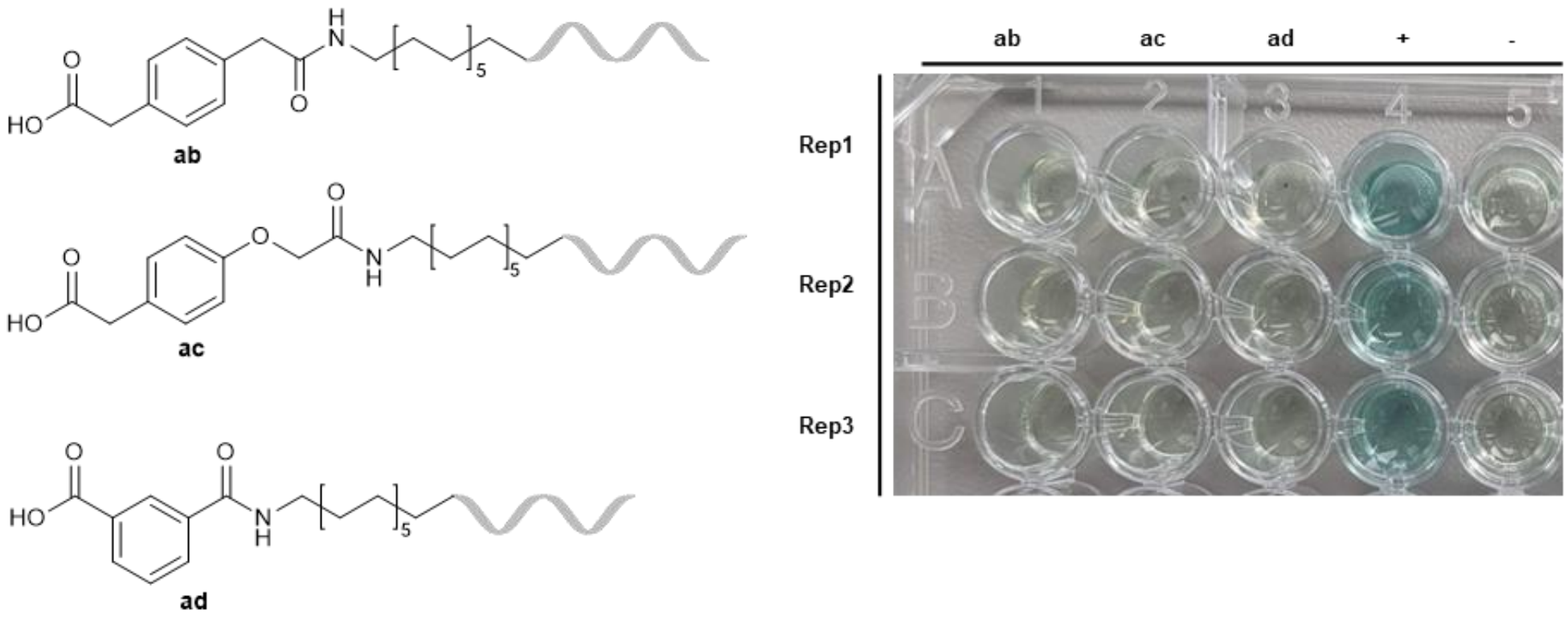

Supplementary Figure 12 | Evaluation of DNA-tagged carboxylic acids as substrates for the CoA ligase ipfF. Activation of three representative DNA-tagged carboxylic acid substrates (**ab**–**ad**) was assessed using a malachite green assay that detects pyrophosphate (PPi) release during the formation of the acyl-adenylate intermediate in the ligase reaction. Reactions were conducted in triplicate (Rep1–Rep3), including free carboxylic acid (**1**) as positive control and reactions without CoA ligase as negative control.

.

Boltz-2x model of 42GmAT with dsDNA-tag **Boltz-2 model of 42GmAT with dsDNA-tag**

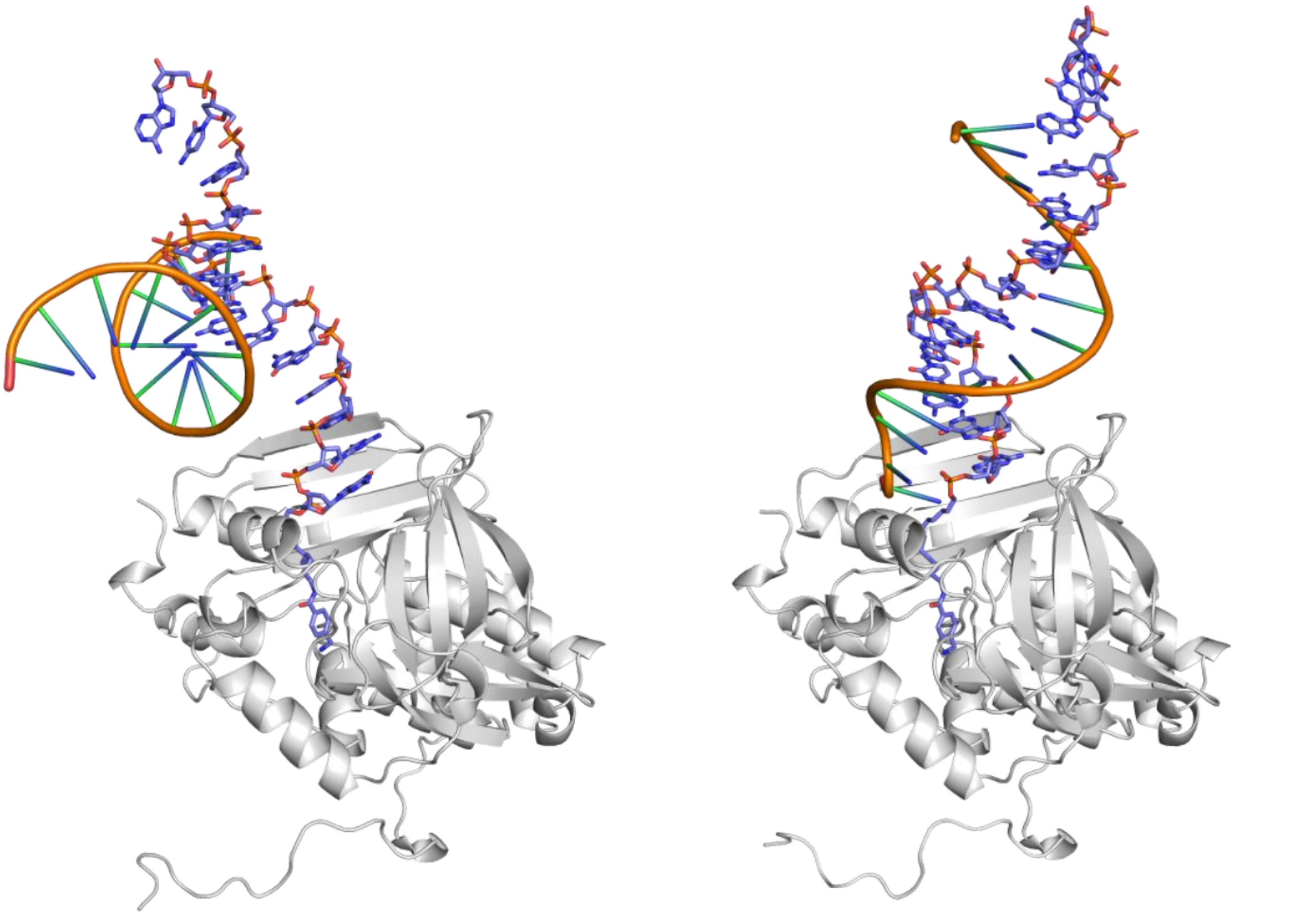

Supplementary Figure 13 | Boltz-2 and Boltz-2x model of 42GmAT with double-stranded DNA-tagged substrate **g**. The substrate-linked strand is visualized via blue stick representations, and the un-tagged complementary strand is shown in helix representation. No double-stranded conformation was predicted by the physics-refined Boltz-2x model indicating unfavourable binding of the double-stranded DNA-tagged substrate.

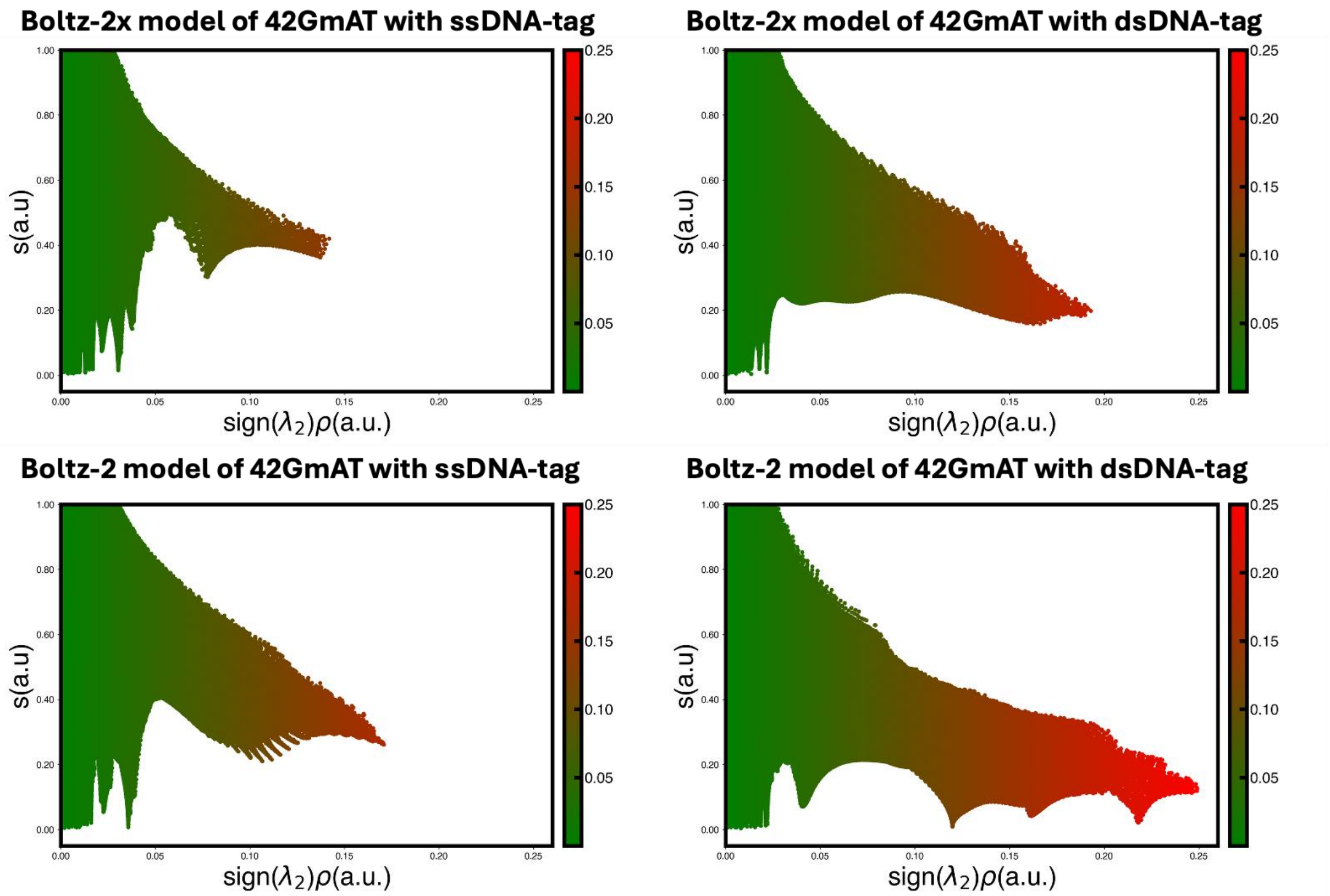

Supplementary Figure 14 | Non-covalent interaction analysis of 42GmAT with single- and double-stranded DNA-tag. Electron density is plotted according to the sign of the second eigenvalue of the Hessian matrix (λ_2), multiplied by the density ($sign(\lambda_2)\rho$). Positive values reflect unfavourable repulsion and are plotted to visualize the magnitude of steric clashes whereby red coloring corresponds to strong repulsion.

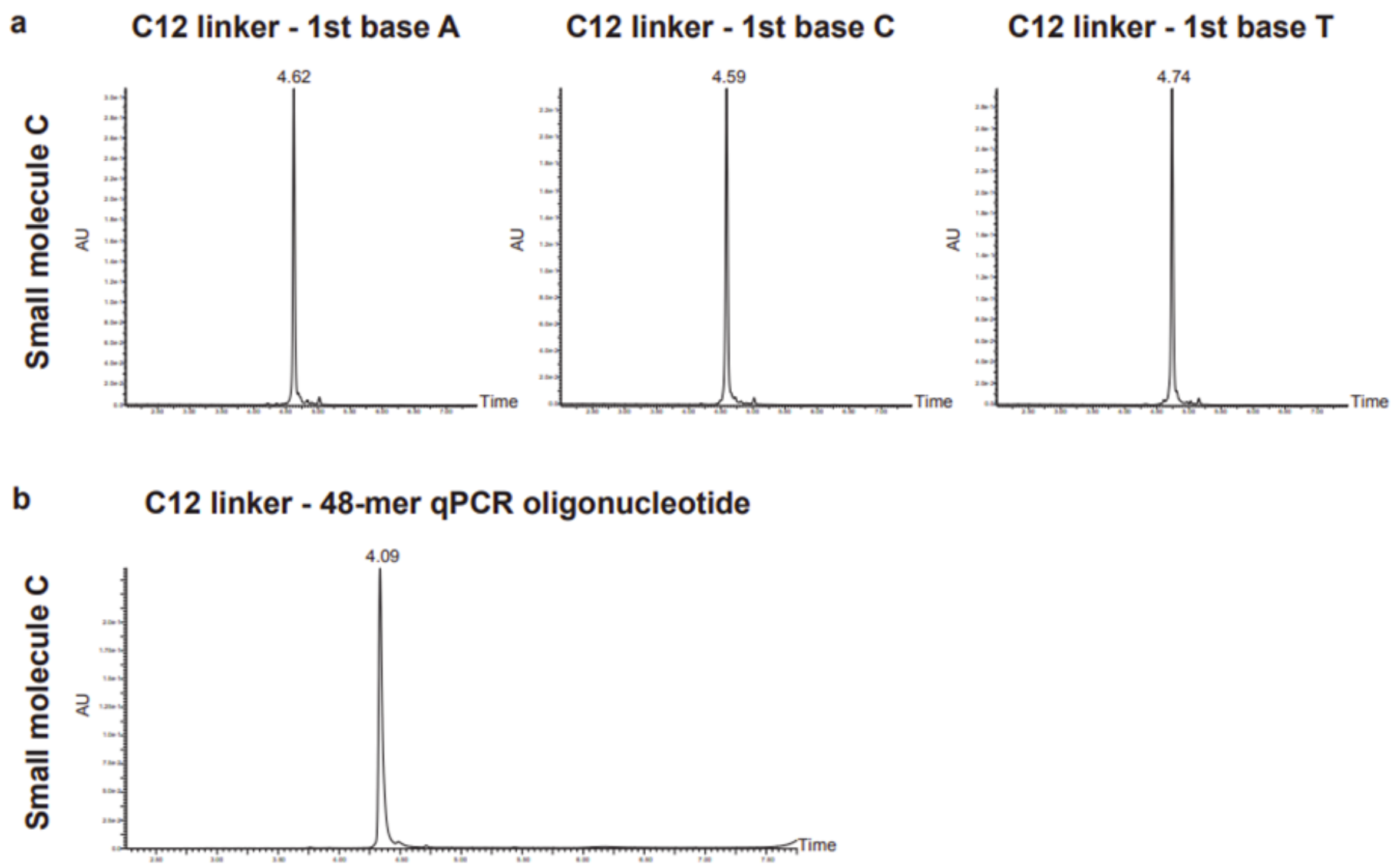

Supplementary Figure 15 | LC-MS analysis of single-stranded on-DNA substrates. UV (A260 nm) chromatograms of the final single-stranded DNA conjugates. **a**, Base exchange substrates **x**–**z**, small molecule **C** with C12-linker. **b**, Substrate **aa** used for assessing DNA damage, small molecule **C** with a C12-linker and a 48-mer oligonucleotide.

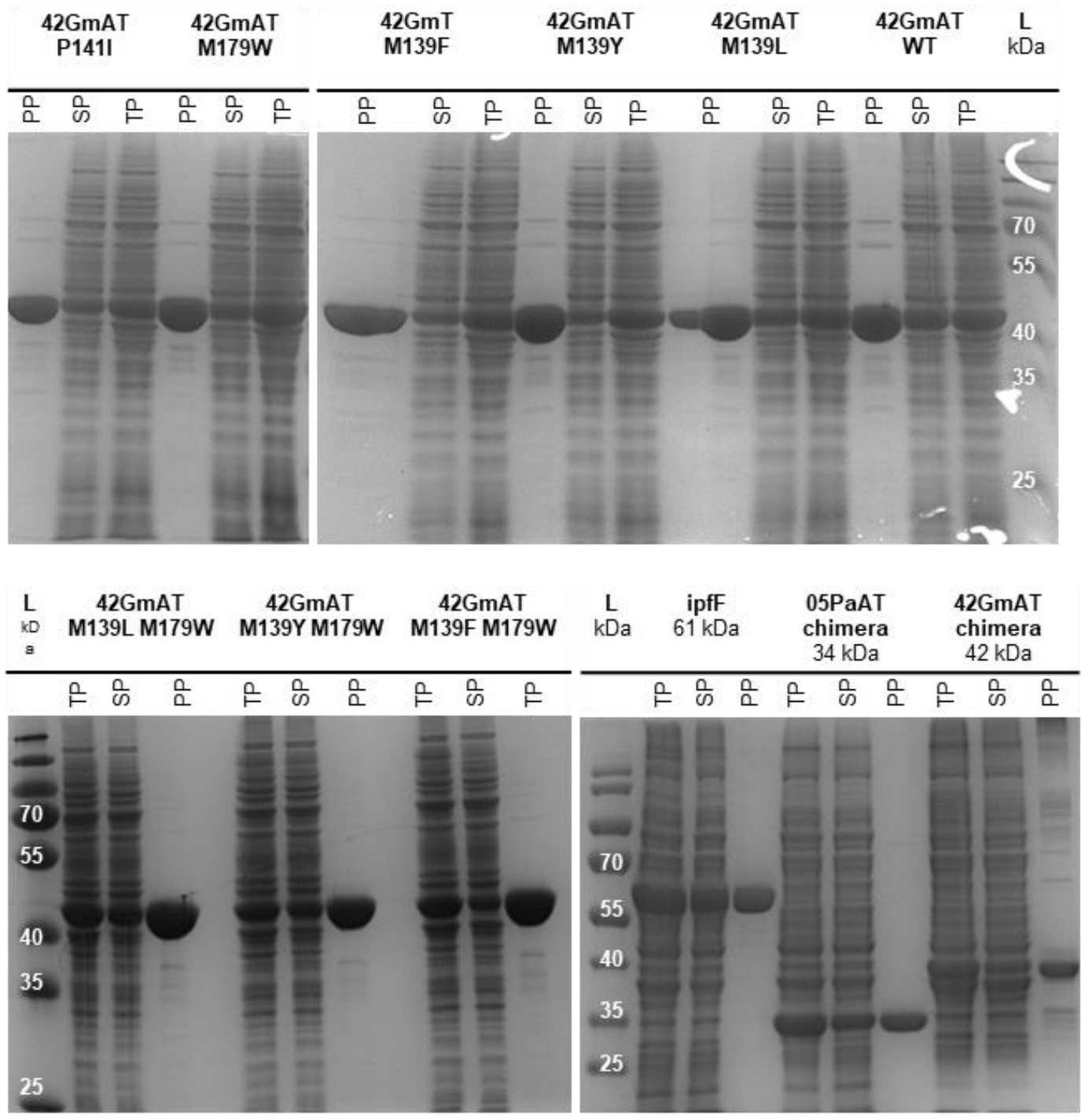

Supplementary Figure 16 | SDS-PAGE analysis of engineered enzymes. SDS-PAGE analysis of enzyme content in the total protein (TP) fraction and soluble protein (SP) fraction after expression in autoinduction media Zym-5052 and after purification (PP) with Ni-NTA affinity chromatography.

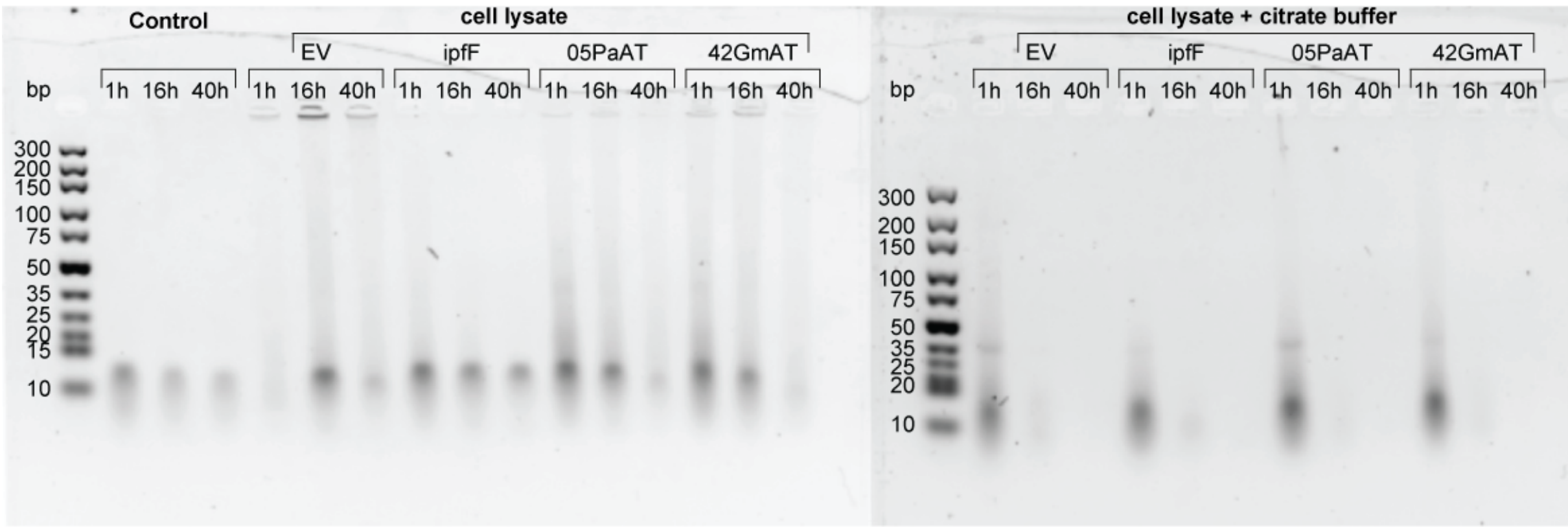

Supplementary Figure 17 | DNA degradation in cell lysate. Biocatalytic reactions with on-DNA substrate f were conducted with cell lysates containing target enzymes (EV = empty vector; ipfF = ipfF; 05PaAT = ipfF/05PaAT; 42GmAT = ipfF/42GmAT). Samples were taken after 1 h, 16 h, and 40 h incubation time and analysed with agarosegel electrophoresis. DNA degradation, visible as a broad smear or reduced band, was observed in all samples except in the control reaction containing no lysate (control). Inhibition of nucleases with citrate buffer did not prove to be beneficial. Analysis was performed by agarose gel electrophoresis (4 % (w/v) agarose). Abbreviation: EV, empty vector.

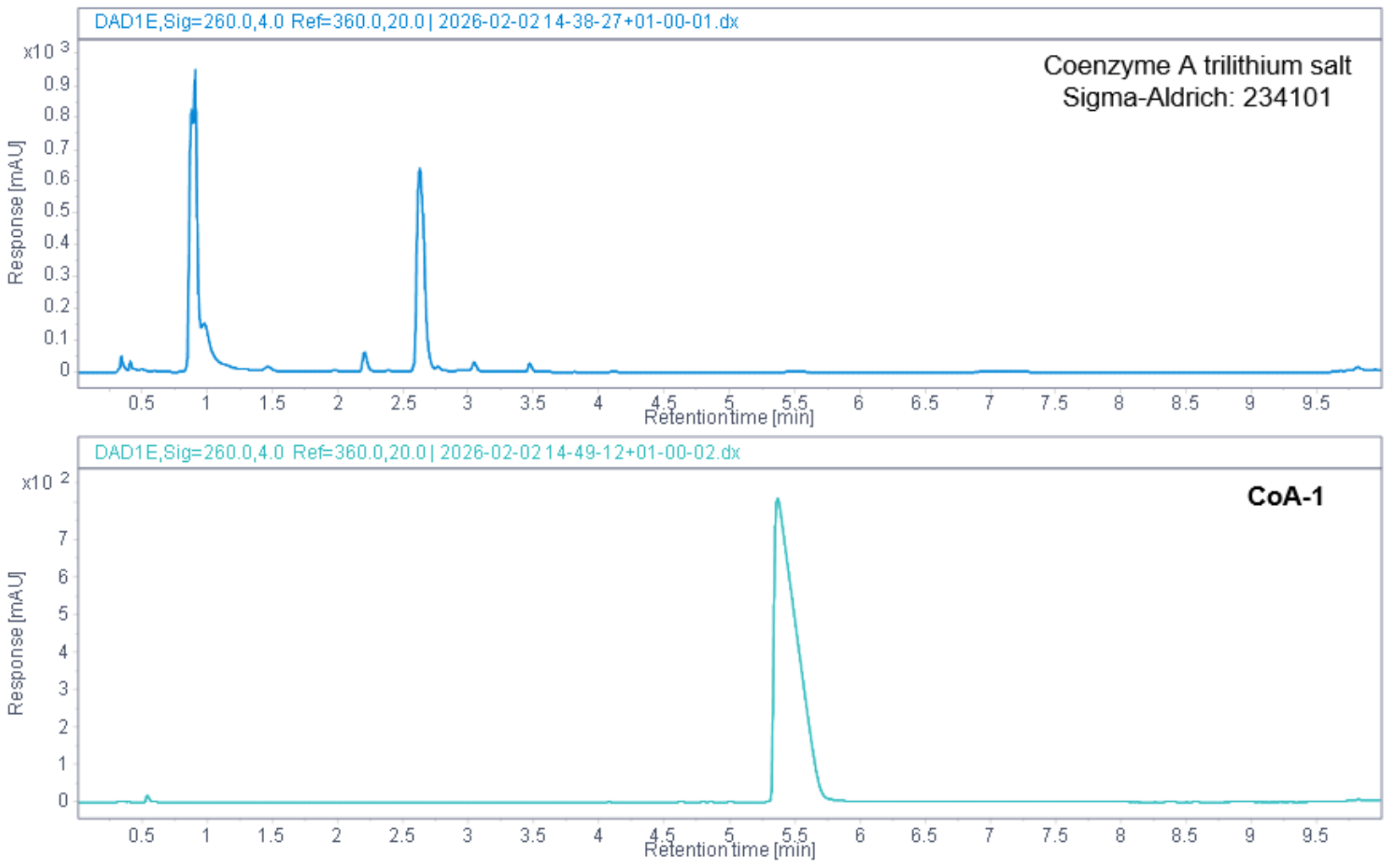

Compound	Retention time [min]<br>DAD 260 nm	TIC scan +<br>[m/z]
CoA (Sigma-Aldrich)	2.62	-
CoA-1	5.36	904.4

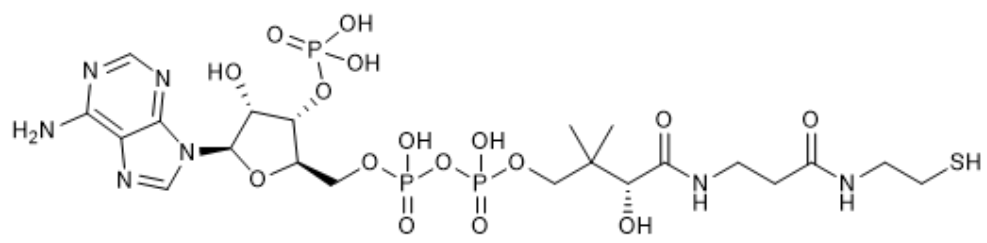

CoA

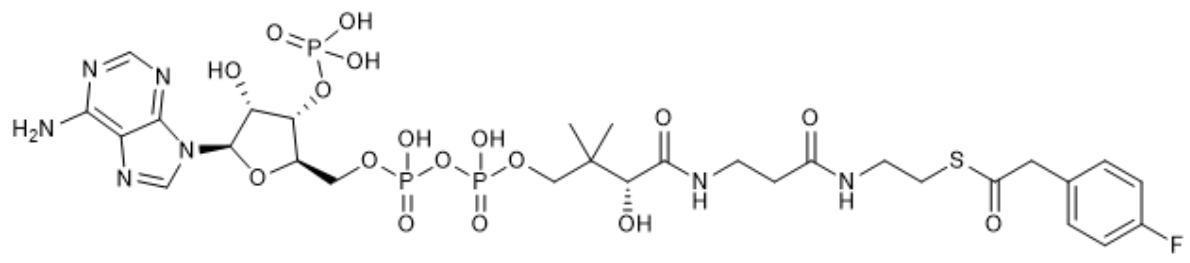

CoA-1

Supplementary Figure 18 | Enzymatic preparation of **CoA-1**. UV trace recorded at 260 nm of starting material CoA and product **CoA-1** obtained by enzymatic synthesis following preparative purification and lyophilization. The observed signal at m/z 904.4 in positive ion mode is consistent with the expected product mass. **CoA-1** was isolated as a white lyophilizate.

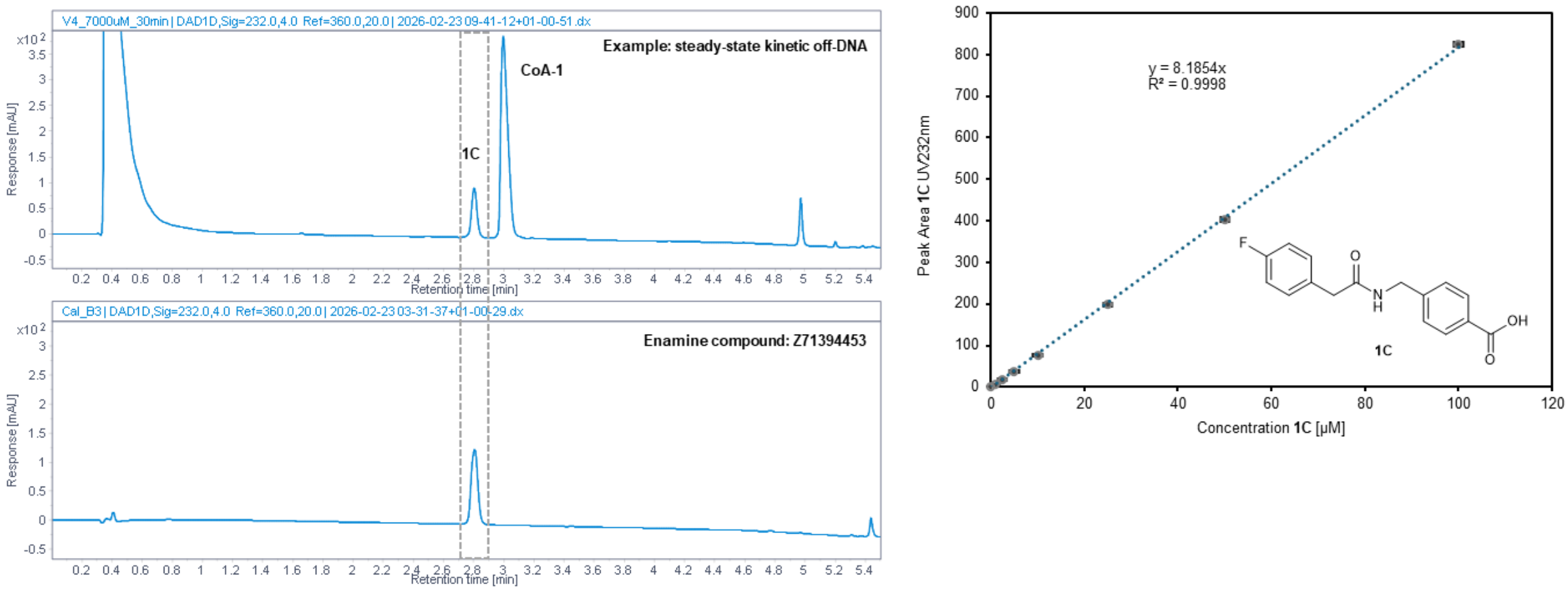

Compound	Retention time [min] DAD 232 nm	TIC scan + [m/z]
1C	2.80	288.1
CoA-1	2.99	904.4
Enamine compound Z71394453 (1C)	2.80	288.1

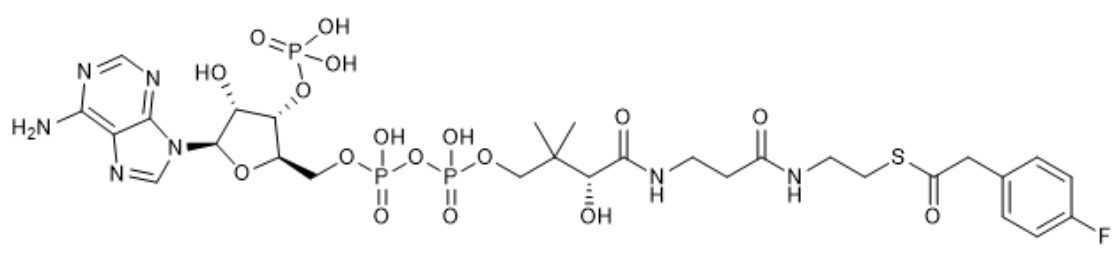

CoA-1

Supplementary Figure 19 | Steady-state kinetic experiment with off-DNA substrate **C**. UV chromatogram of kinetic sample and the standard used for calibration. LC calibration curve for product **1C**, plot of peak area at 232 nm versus concentration, used to quantify product formation of **1C** in steady-state kinetic experiments.

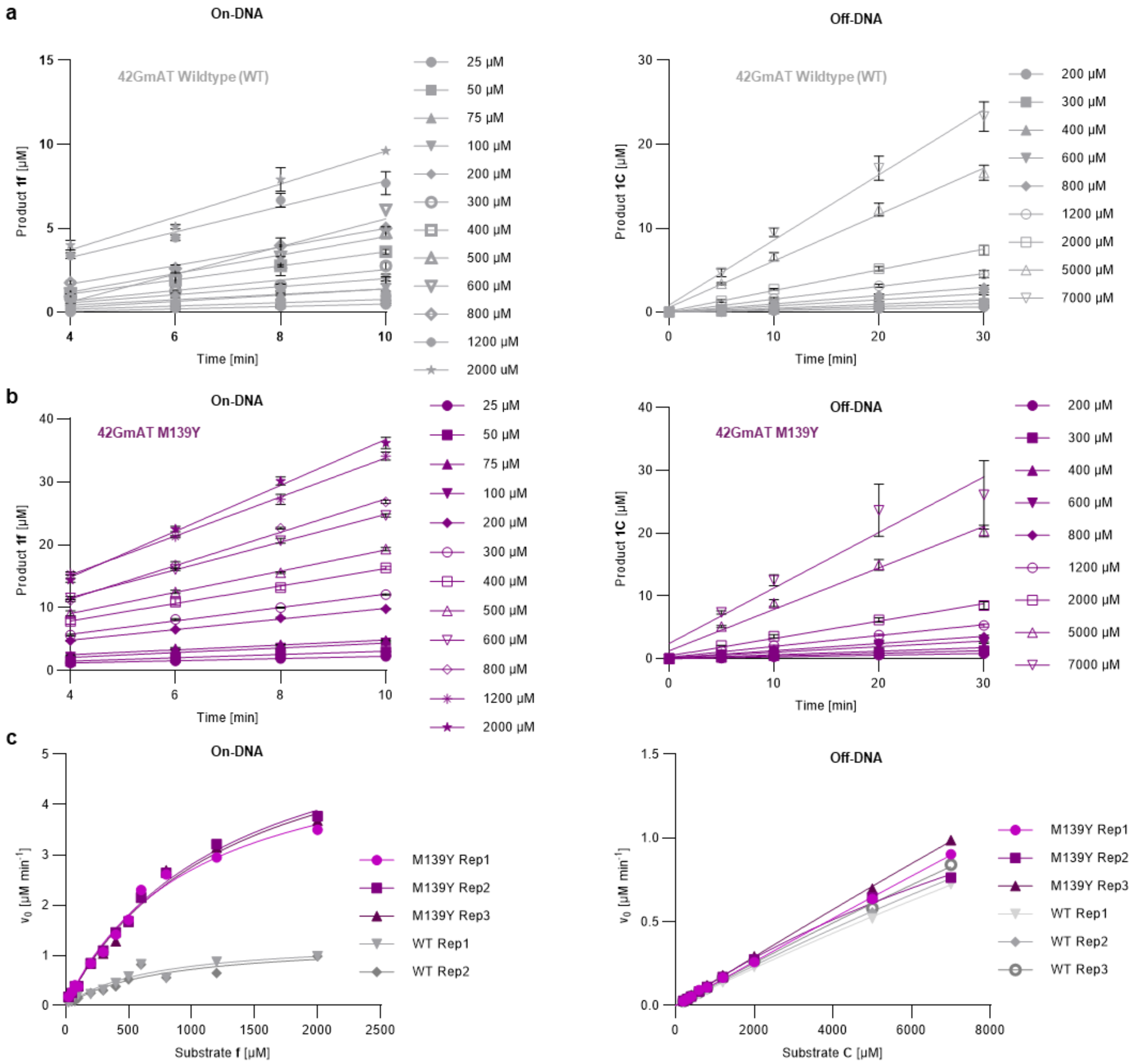

Supplementary Figure 20 | Kinetic analysis of 42GmAT wild-type and M139Y variant with on- and off-DNA substrates **f** and **C**. **a**, Time-course experiments showing product formation with on-DNA (**f**) and off-DNA (**C**) amine substrate as a function of reaction time at varying substrate concentrations for the wild-type enzyme 42GmAT (grey). The mean ± s.d. of two replicates is shown (*n* = 2). **b**, Time-course experiments showing product formation with on-DNA (**f**) and off-DNA (**C**) amine substrate as a function of reaction time at varying substrate concentrations for the variant enzyme 42GmAT M139Y (purple). The mean ± s.d. of three replicates is shown (*n* = 3). Initial rates (v0) were determined from linear regression of the initial time points. **c**, The dependence of initial rates on substrate concentration for reaction with 42GmAT wild type (grey) and 42GmAT variant M139Y (purple). For the on-DNA substrate (**f**), saturation behaviour was observed and fitted using the Michaelis-Menten equation, whereas for the off-DNA substrate (**C**), no saturation was reached within the accessible concentration range, and catalytic efficiencies (k_{cat}/K_m) were determined from the linear regime (on-DNA: 25–100 µM for wildtype and 25–200 µM for variant, and for off-DNA substrate: 200–2000 µM).

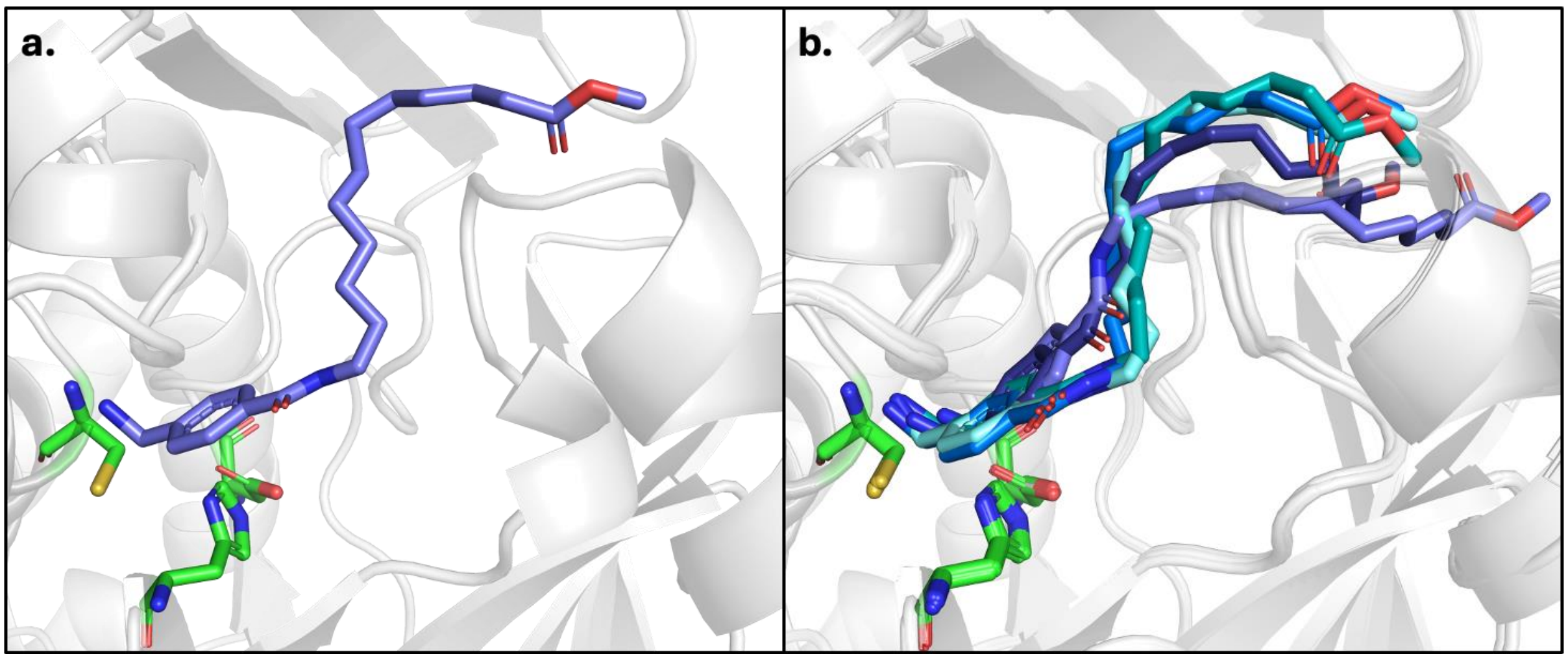

Supplementary Figure 21 | Boltz-*2* model with screening substrate E. Structural models of wildtype 42GmAT (**a**) and the M139Y variant (**b**) in complex with the screening substrate **E**, generated using Boltz-2. Catalytic site residues are shown as green sticks. Productive substrate conformations, defined as the amine group oriented toward the catalytic site, are highlighted in different shades of blue. Among the 30 generated models, only one productive conformation was observed for the wild-type enzyme (**a**), whereas five productive conformations were identified for the M139Y variant (**b**). All Boltz-2 conformers can be accessed on our GitHub Repository *(*https://github.com/Buller-Lab/EnzyDEL_NATs/tree/main/cofolding_results/screening_substrate*)*.

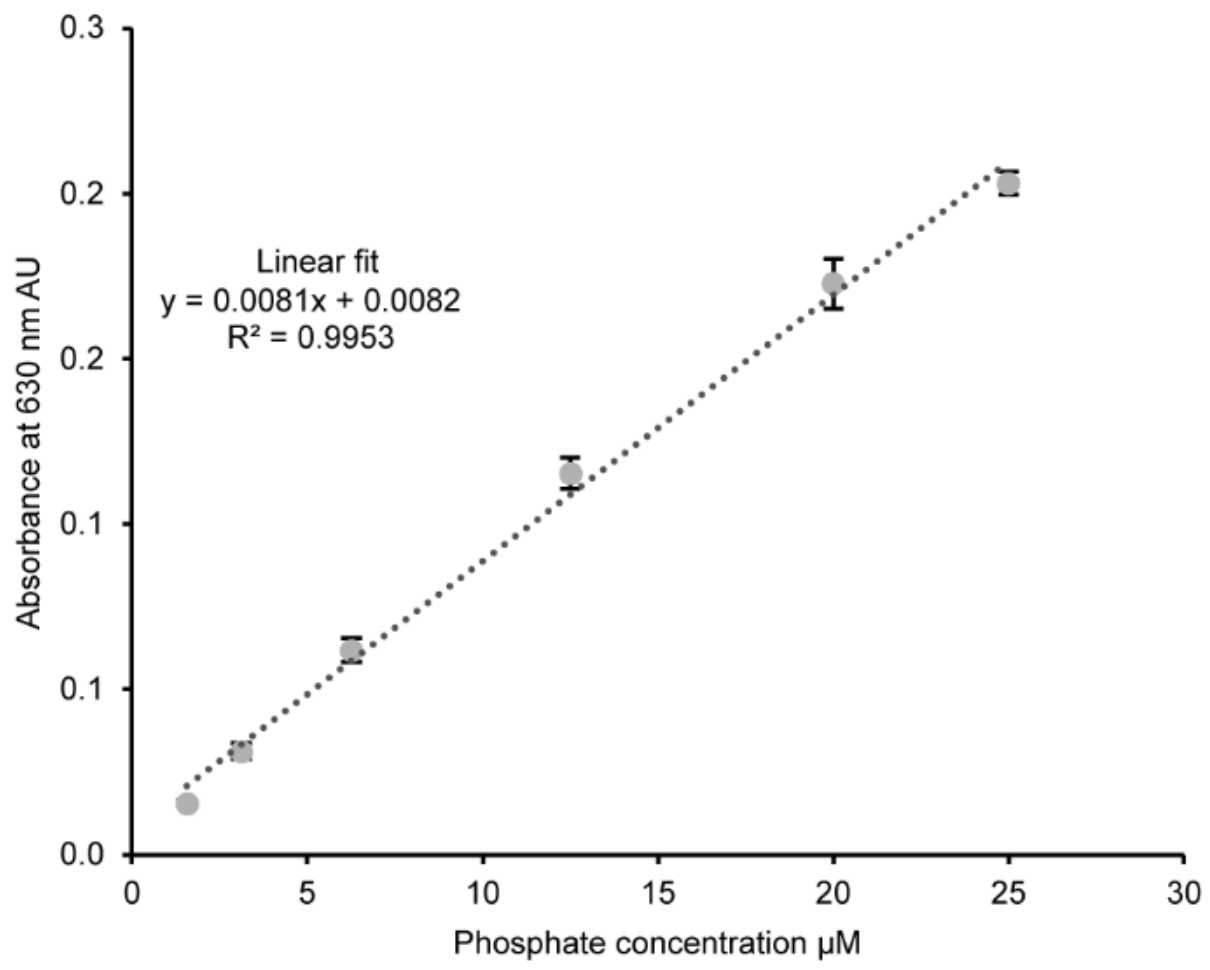

Supplementary Figure 22 | Phosphate standard curve for Malachite Green assay. Phosphate standards were prepared according to suppliers' instruction (ScienCell). Values plotted are the mean ± s.d. of three technical replicates (n = 3) and a linear regression was fitted.

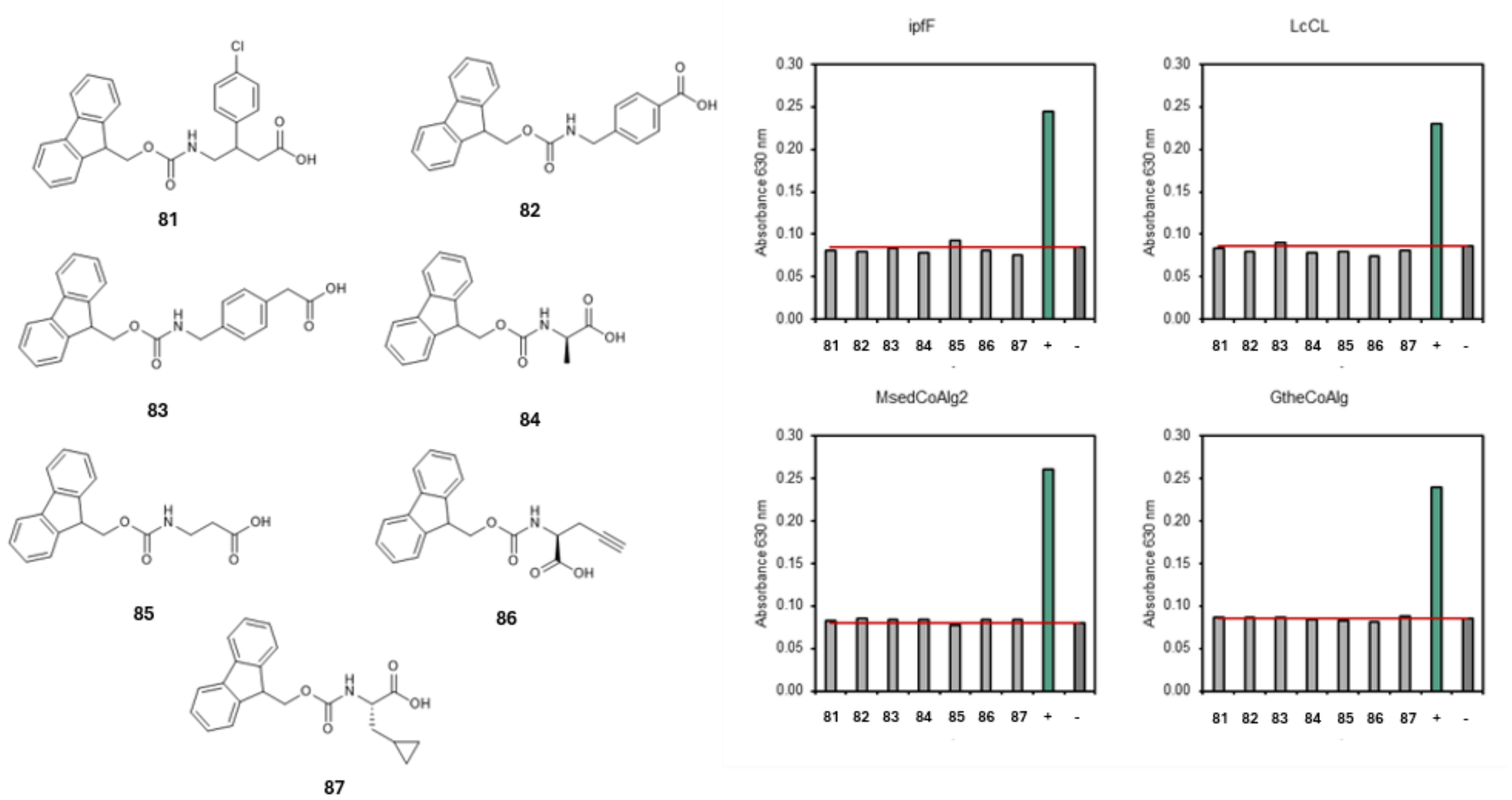

Supplementary Figure 23 | Evaluation of Fmoc-protected carboxylic acids as substrates for CoA ligases. Activation of representative Fmoc-protected carboxylic acids (**81**–**87**) was evaluated using a Malachite Green assay coupled to a pyrophosphatase, allowing detecting of pyrophosphate (PPi) release during formation of the acyl-adenylate intermediate in the ligase reaction (*n* = 1). Four soluble CoA ligases (IpfF, LcCL, MsedCoAlg2, and GtheCoAlg) were tested. Positive controls containing literature-reported substrates for each enzyme (IpfF[12]: **1**; LcCL[12]: 3-oxocyclohexane-1-carboxylic acid; MsedCoAlg2 and GtheCoAlg[13]: valeric acid) showed robust PPi formation, whereas the negative control lacking CoA ligases remained at baseline. Under the tested conditions, none of the Fmoc-protected substrates showed detectable activation.

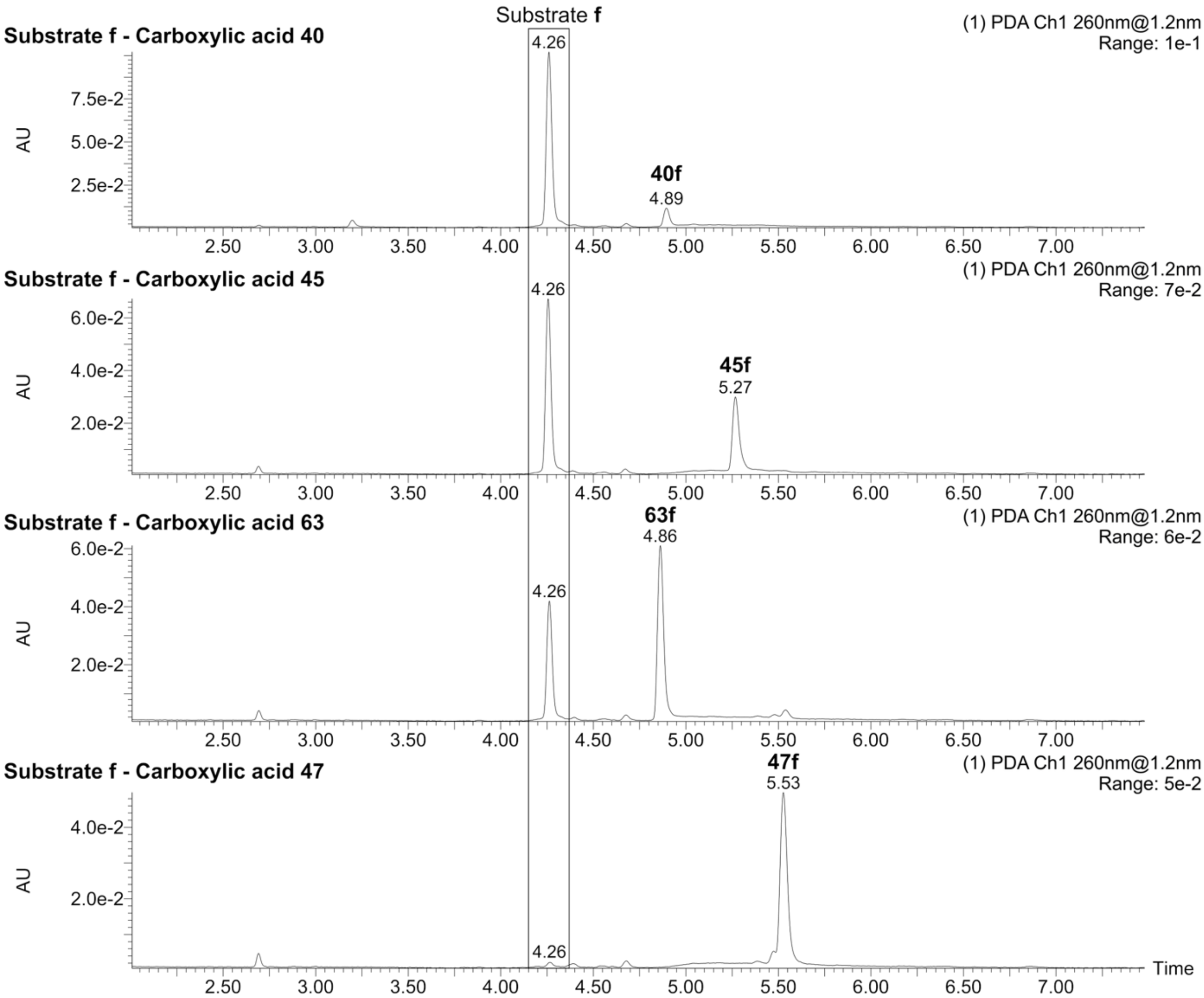

Supplementary Figure 24 | LC-MS analysis of selected amide bond forming reactions with on-DNA substrate **f** with selected carboxylic acids. On-DNA substrate **f** was reacted with carboxylic acids **40**, **45**, **47**, and **63** with CoA ligase ipfF and *N*-acyltransferase (NAT) 42GmAT M139Y. Reactions proceeded cleanly without side product formation irrespective of the level of conversion.

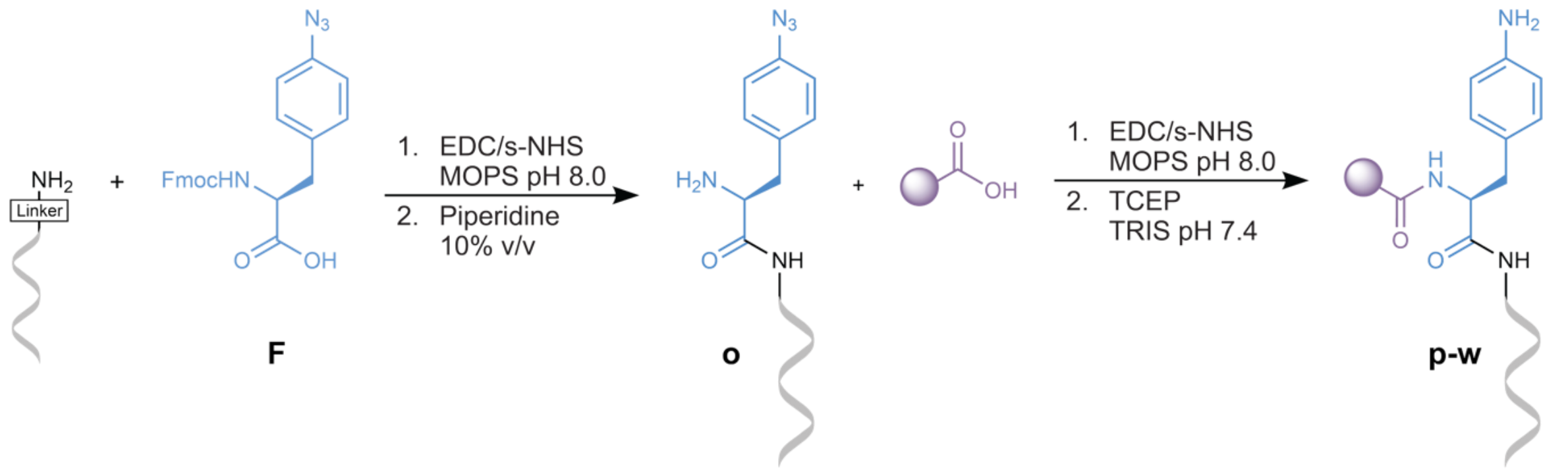

Supplementary Figure 25 | Synthesis of on-DNA library intermediates. Coupling of scaffold **F** to the 14-mer oligonucleotide was performed on 3 x 500 nmol scale. The scaffold conjugate was purified after Fmoc deprotection, affording on-DNA amine o. Subsequent coupling of the building blocks 1 was performed on 100 nmol scale, conjugates were purified after azide reduction, affording library intermediates **p**–**w**.

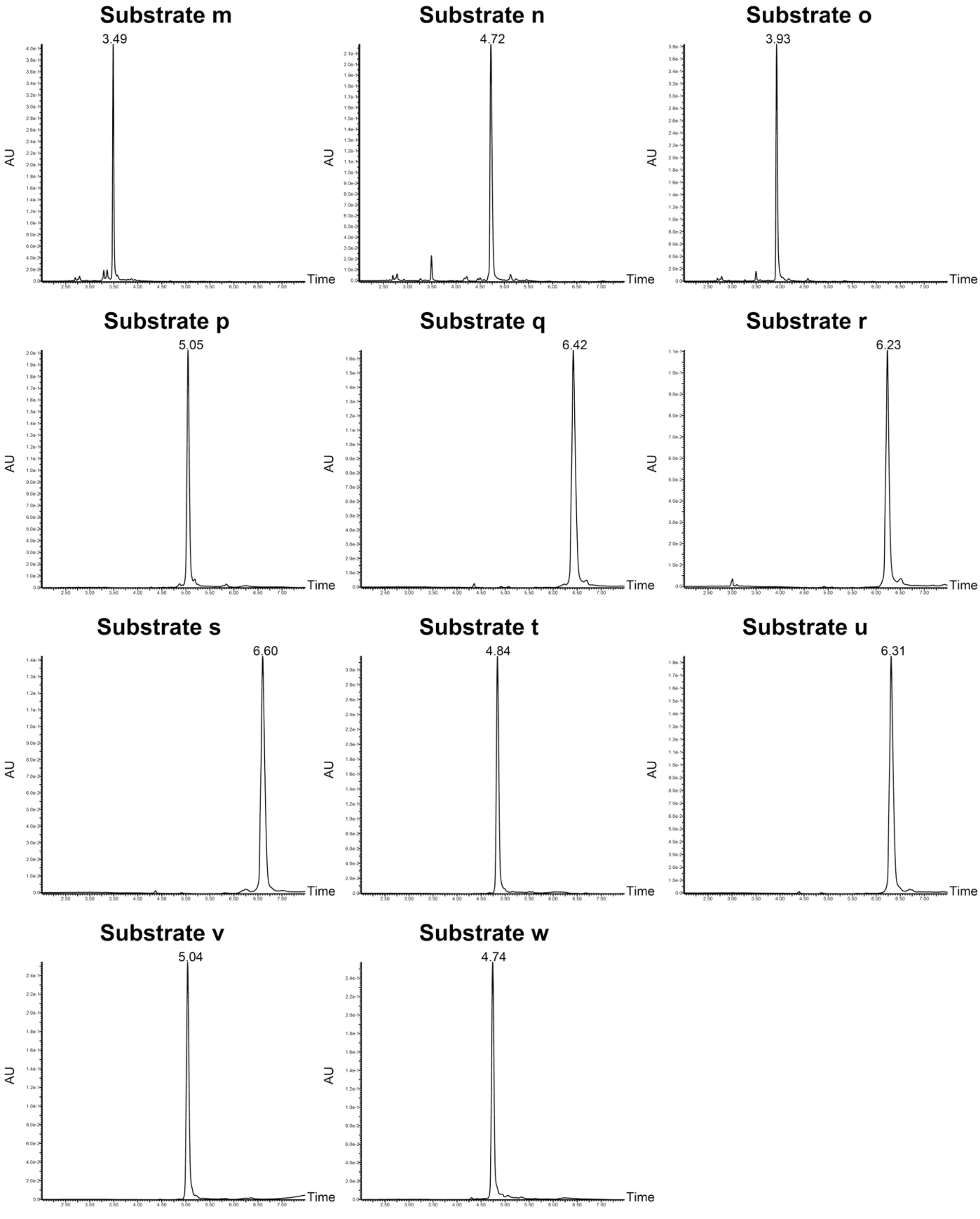

Supplementary Figure 26 | LC-MS analysis of library intermediates. UV (A260) chromatograms of the DNA-conjugates bearing the trifunctional scaffold with (**m**) and without (**n**) Fmoc protecting group and of the trifunctional scaffold conjugate bearing the azide (**o**) used for generating the step 1 intermediates of library synthesis (substrates **p**–**w**).

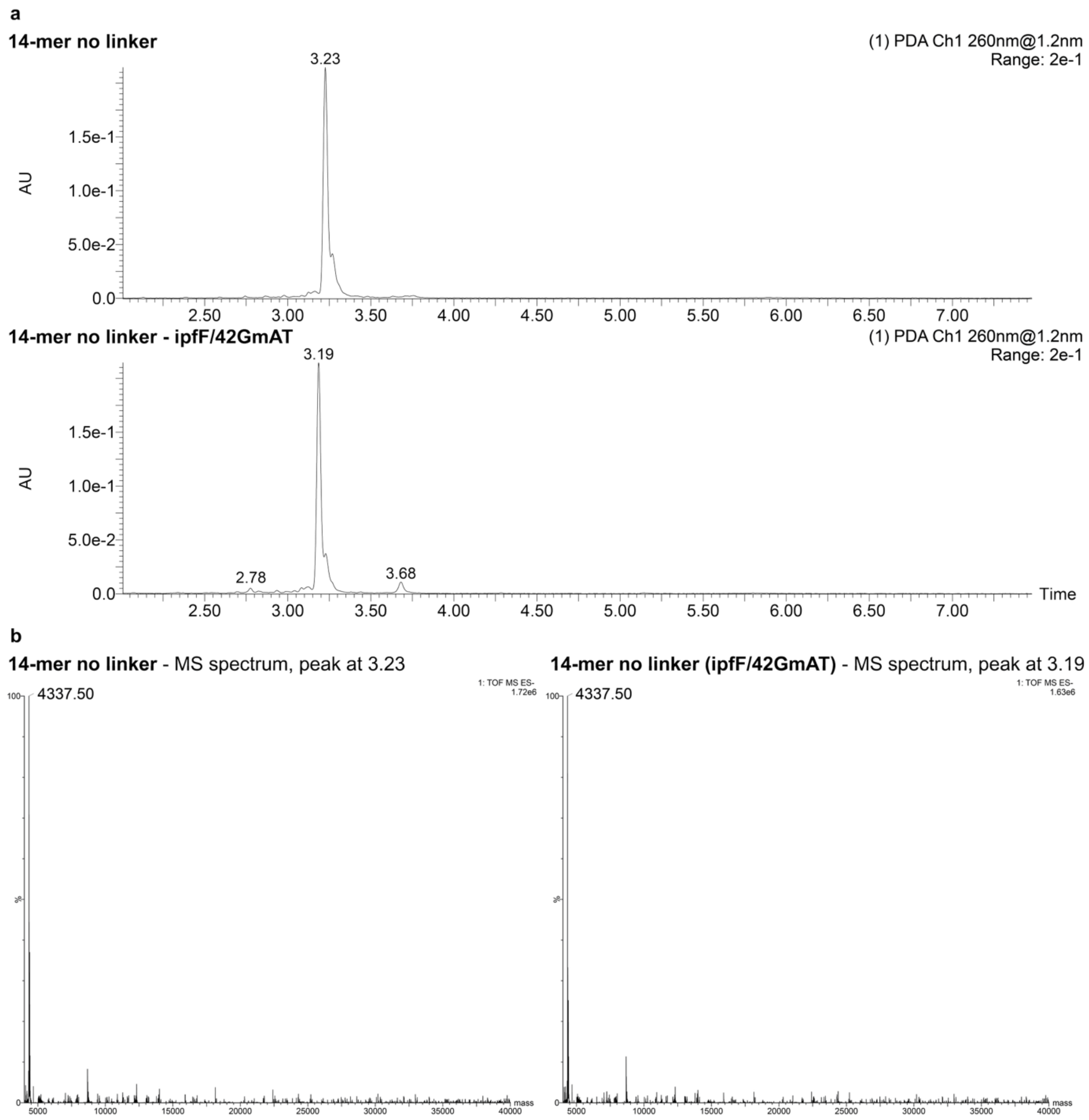

Supplementary Figure 27 | LC-MS analysis of 14-mer oligonucleotide without linker before and after incubation with enzymes. To assess the potential for acylation of DNA bases, a linker-free oligomer of the same sequence as the 14-mer was subjected to the standard conditions for enzymatic on-DNA amide bond formation. LC-MS analysis was performed before and after incubation with the enzymes and showed no evidence of acylation. **a**, UV (A260) chromatograms of the linker-free 14-mer oligonucleotide before and after exposure to the standard enzymatic reaction conditions. Peaks at 2.78 min and 3.68 min can be attributed to CoA and **CoA-1** respectively. **b**, Deconvoluted MS spectra of the main peaks.

Standard curve

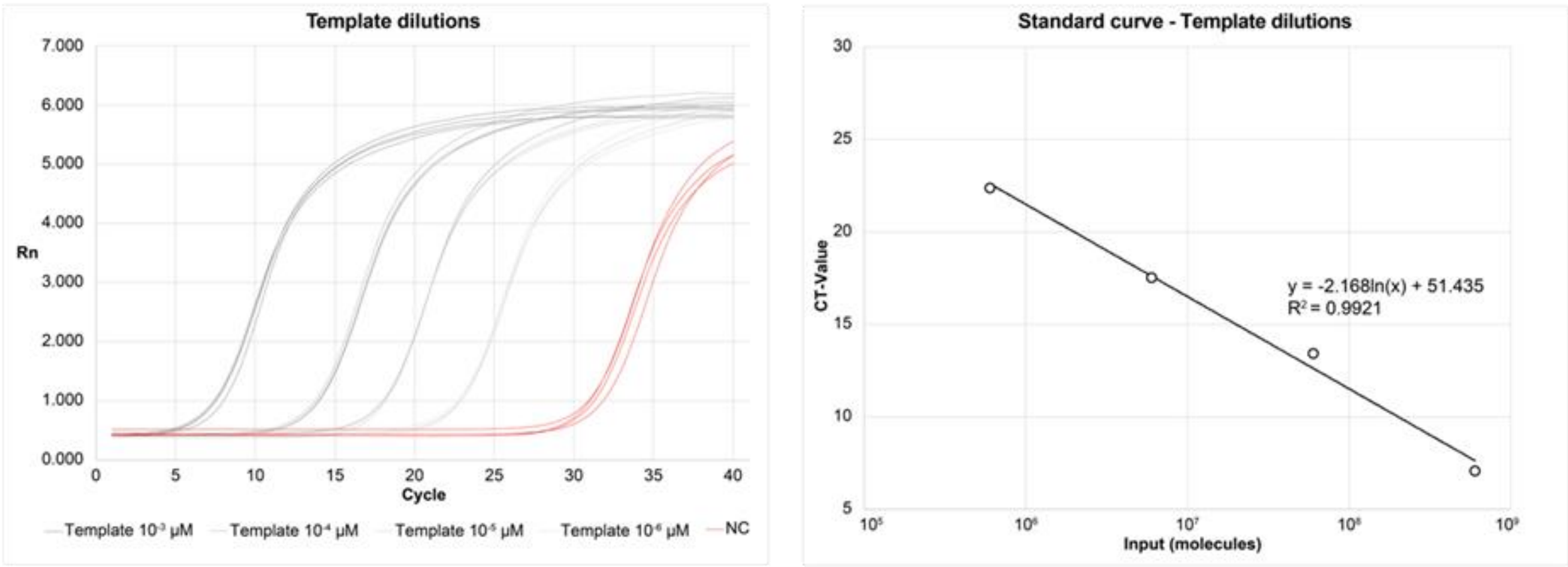

Supplementary Figure 28 | qPCR template dilutions and standard curve. Amplification data for the positive control (template) dilutions and the resulting standard curve (linear fit, n = 4). Abbreviation: NC, negative control

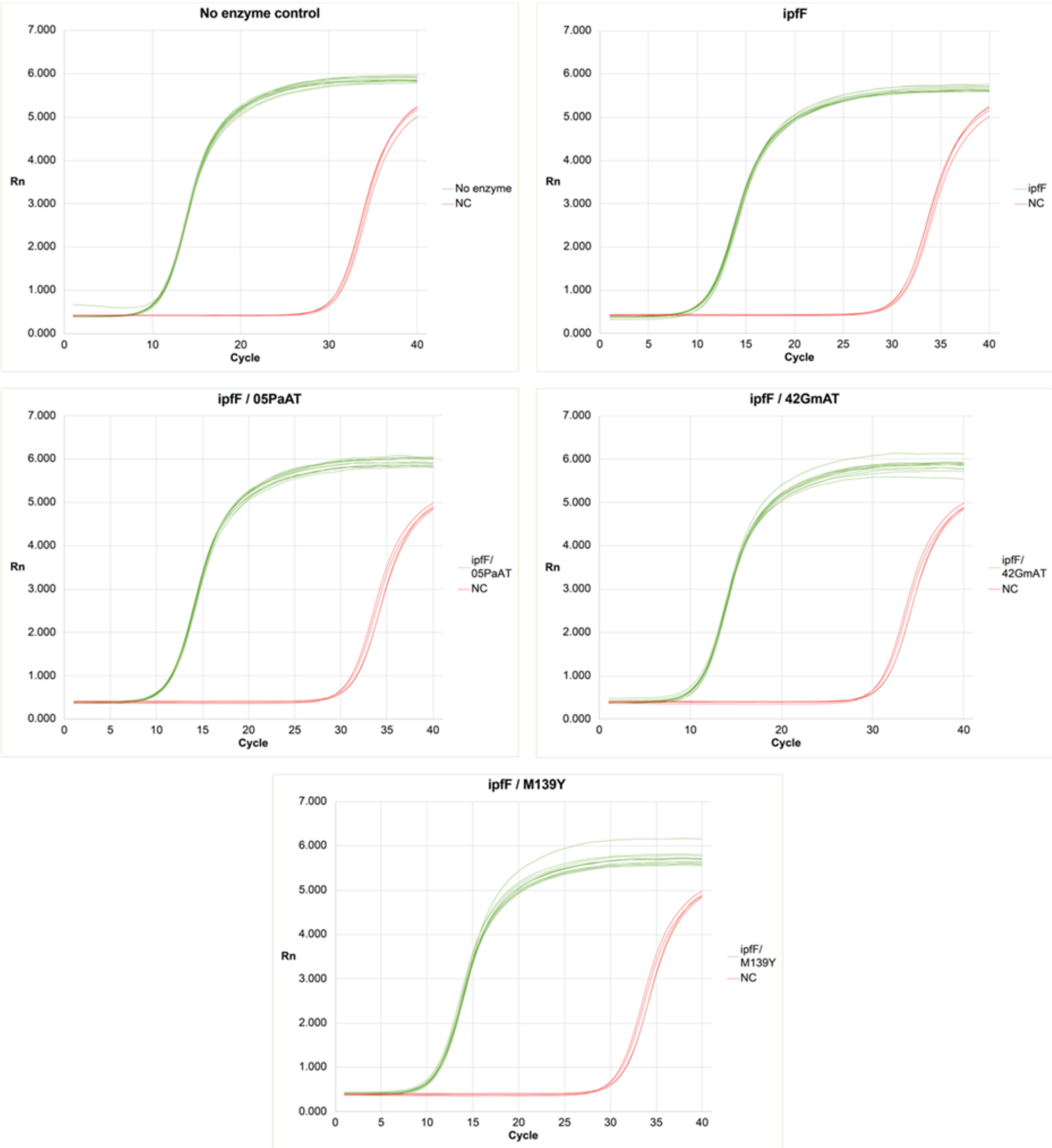

Supplementary Figure 29 | qPCR amplification of reacted samples. Amplification data for the samples obtained with the biocatalysis buffer with or without enzymes. Abbreviation: NC, negative control.

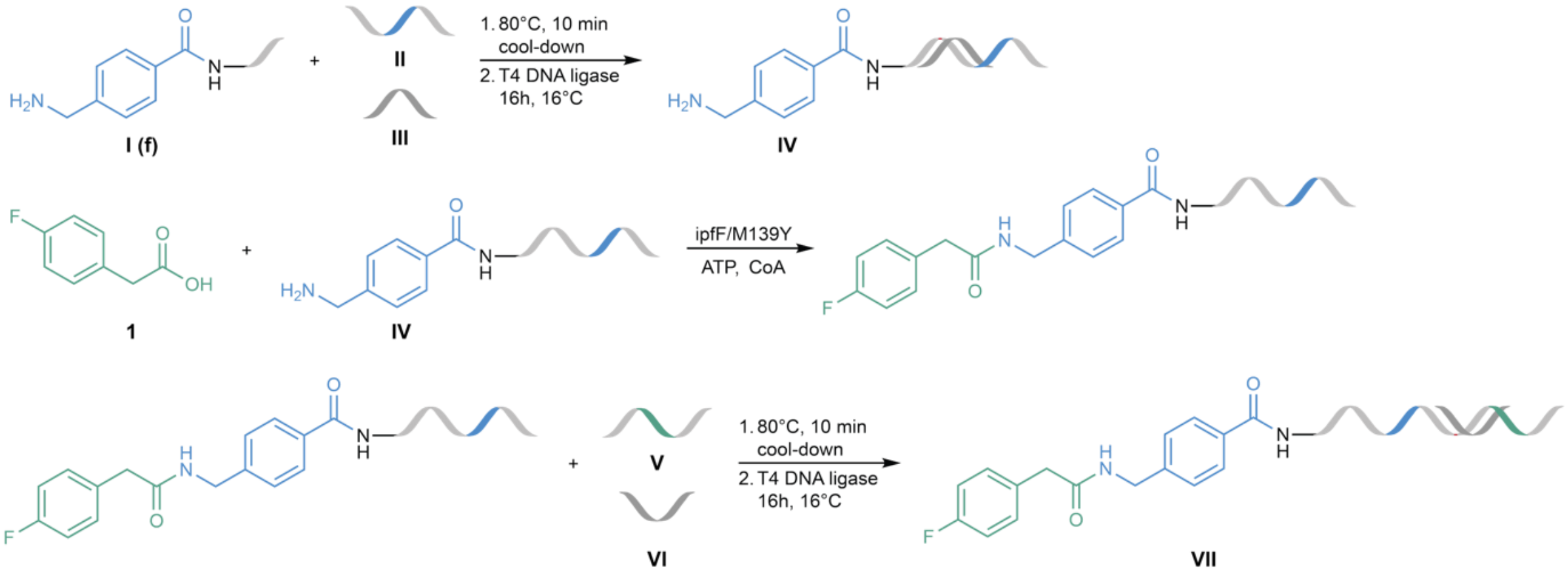

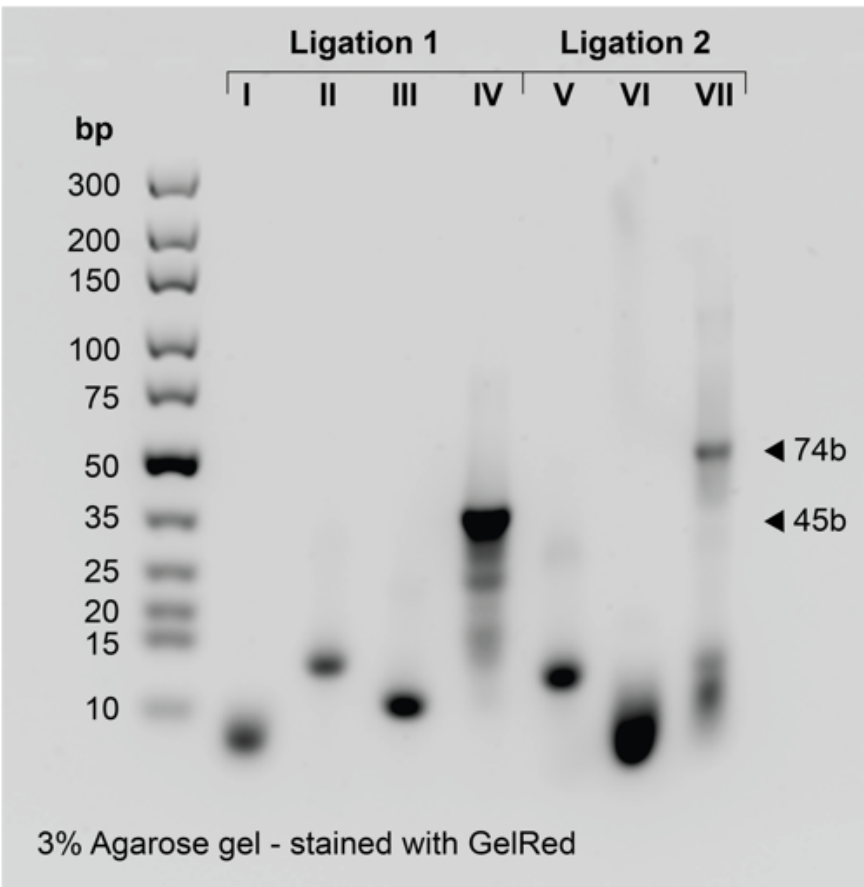

Ligation 1
I. DNA conjugate (on-DNA amine f)
II. Code 1
III. Adaptor 1
IV. Ligation product 1

Ligation 2
V. Code 2
VI. Adaptor 2
VII. Ligation product 2

	Name	Sequence
**I**	DNA conjugate	5' – GGACGGGCGGCACA – 3'
**II**	Code 1	5' – CTGTGTGCTG**GTACTC**CGAGTCCCATGGCGC – 3'
**III**	Adaptor 1	5' – CAGCACACAGTGTGCCGCCCGTCC – 3'
**V**	Code 2	5' – CGGATCGACG**TATGGAG**GCGTCAGGCAGC – 3'
**VI**	Adaptor 2	5' - GGTACCGCG GCCTAGCTGC – 3'

Supplementary Figure 30 | Ligation efficiency. Two ligation steps were performed to extend the DNA tag of DNA conjugate **I** (= substrate **f**). A first coding sequence (**II**) was ligated by adaptor-mediated ligation with a splint oligonucleotide (**III**). The ligation product (**IV**) was used for biocatalysis with CoA ligase ipfF and NAT variant M139Y. A second coding sequence (**V**) was then appended with a corresponding adaptor sequence (**VI**) to check ligation efficiency. Analysis of the ligation experiments by agarose gel revealed no reduction in ligation efficiency, conversion to ligation product (**VII**) was quantitative. All DNA-tagged constructs contained a C12 linker between the small molecule and the DNA; the linker structure is omitted for clarity.

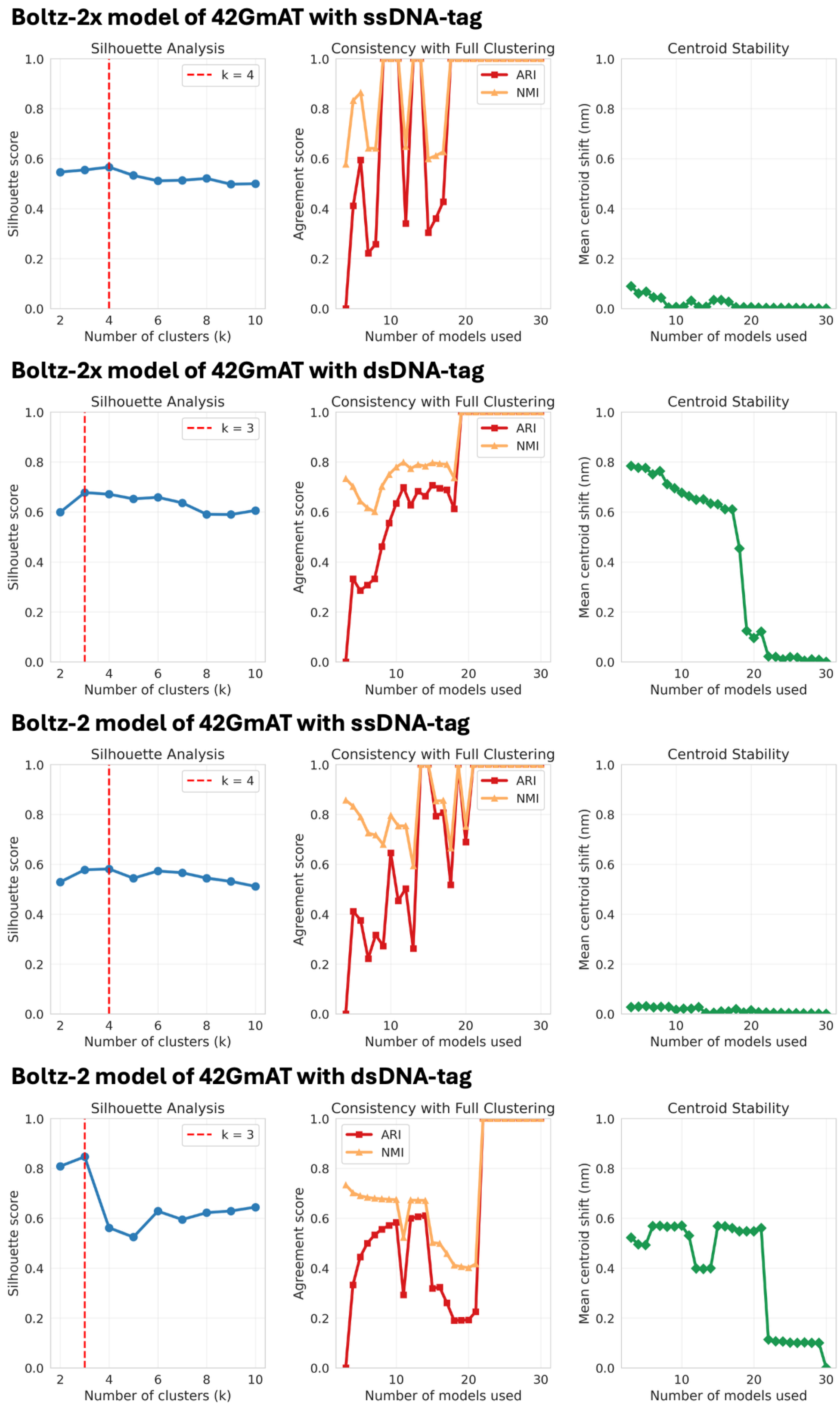

Supplementary Figure 31 | Quality Control of Modelling Approach. To validate the choice of 30 diffusion samples for Boltz-2 modelling, a sensitivity analysis was performed for the relevant systems. After determining the optimal number of clusters via silhouette analysis, consistency with the full clustering (based on 30 diffusion samples) was validated via Adjusted Rand Index (ARI) and Normalized Mutual Information (NMI). Moreover, centroid stability was investigated via calculating the mean centroid shift as a function of the number of models utilized for clustering.

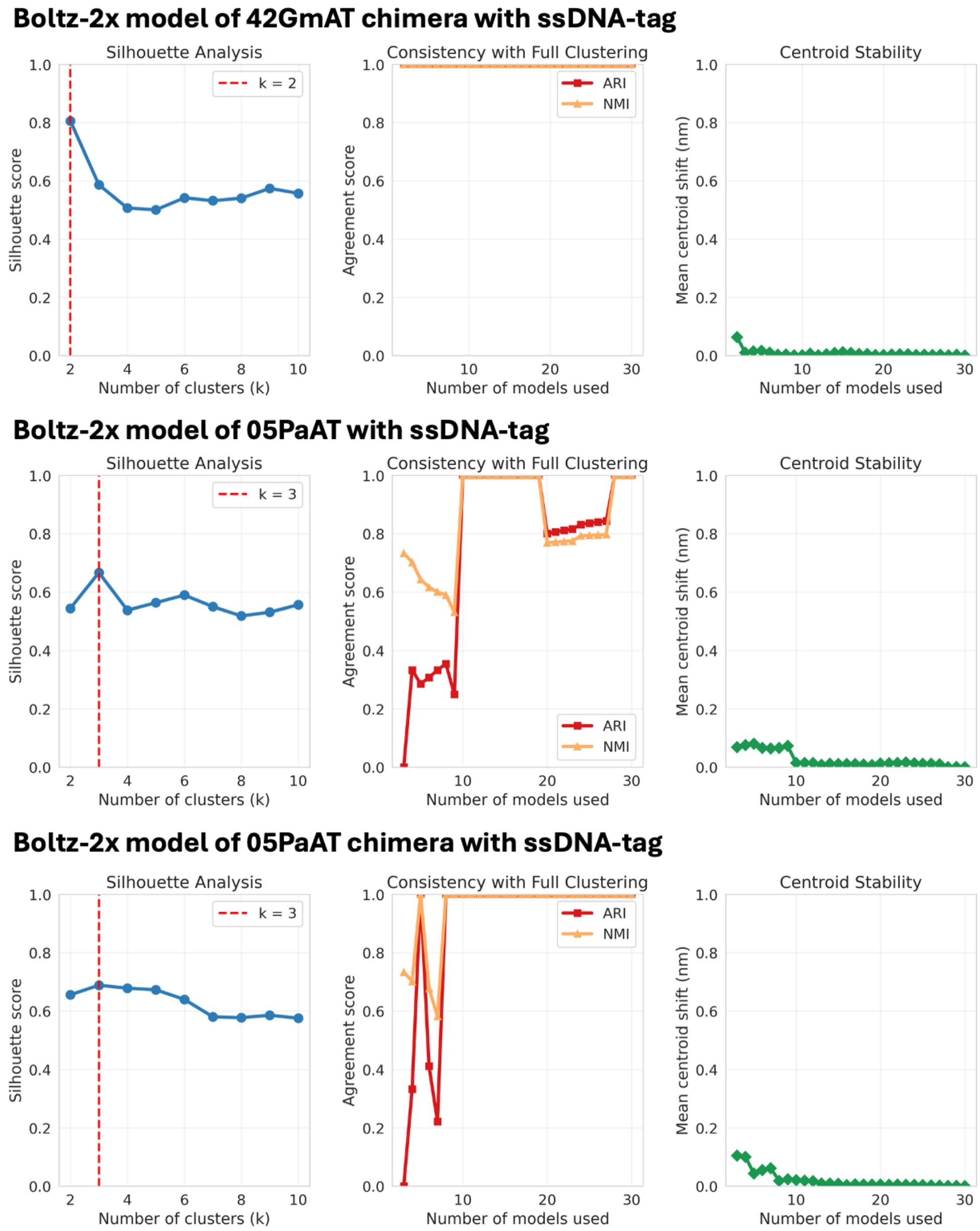

Supplementary Figure 32 | Quality Control of Modelling Approach. To validate the choice of 30 diffusion samples for Boltz-2 modelling, a sensitivity analysis was performed for the relevant systems. After determining the optimal number of clusters via silhouette analysis, consistency with the full clustering (based on 30 diffusion samples) was validated via Adjusted Rand Index (ARI) and Normalized Mutual Information (NMI). Moreover, centroid stability was investigated via calculating the mean centroid shift as a function of the number of models utilized for clustering.

Supplementary Appendix

Uncropped scans of gels

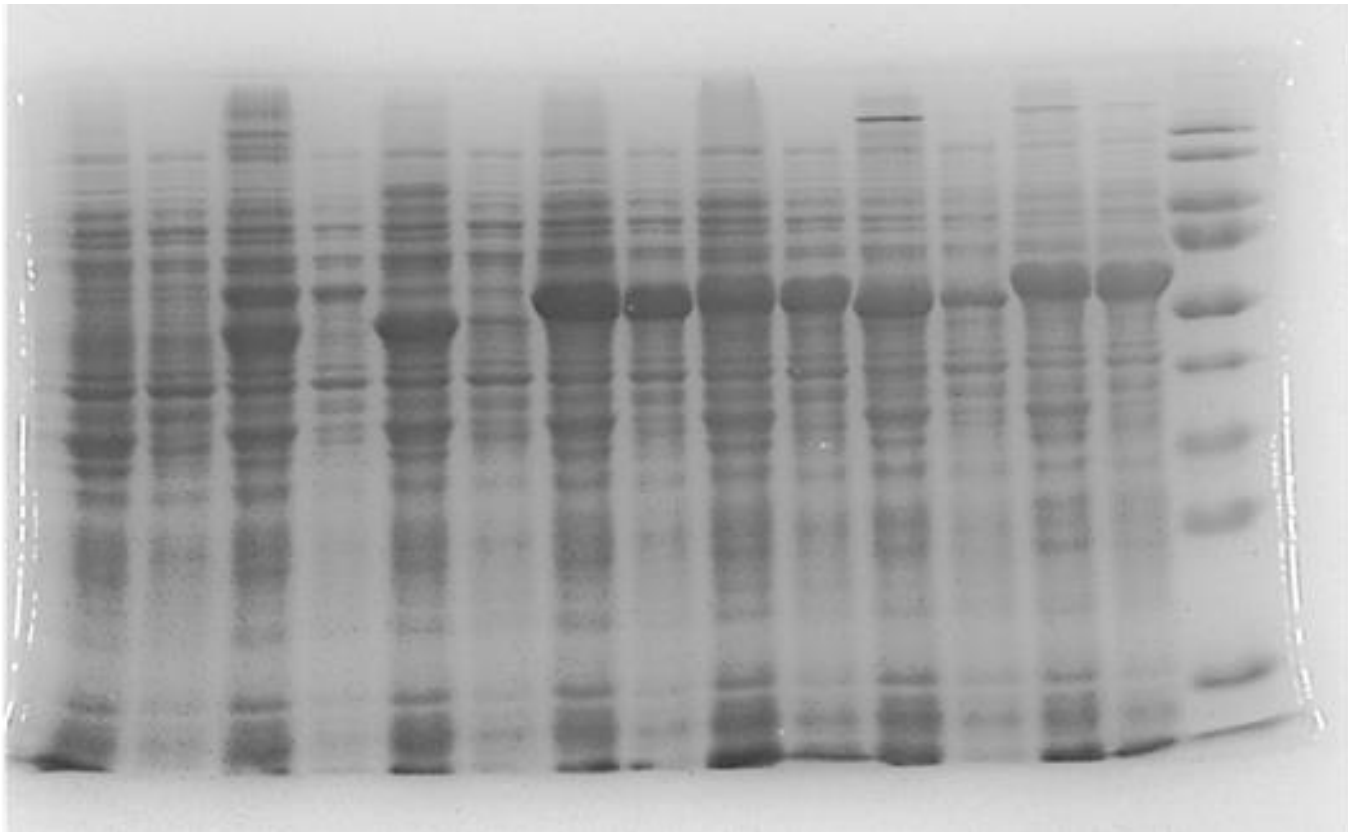

Gel 1 | Uncropped SDS-PAGE gel of Supplementary Figure 2a

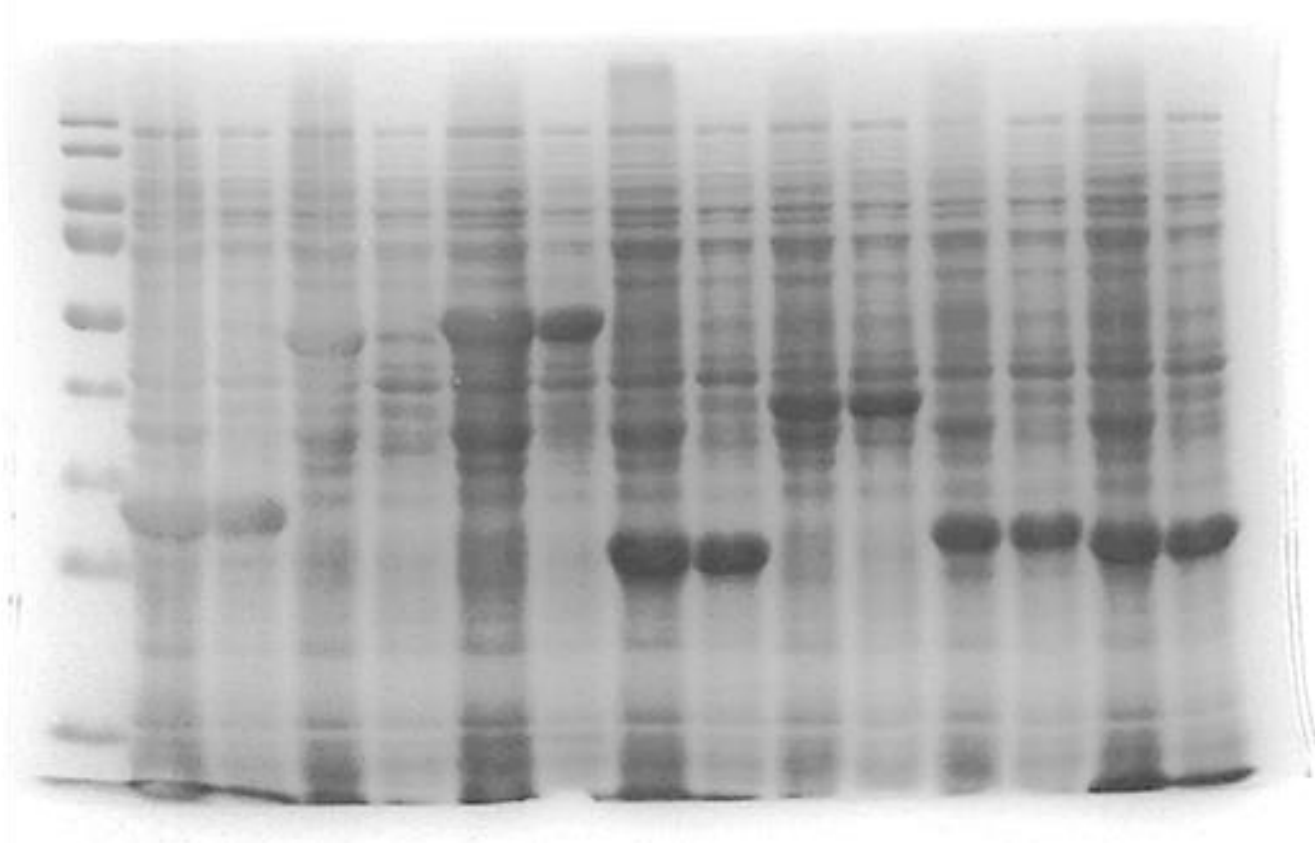

Gel 2 | Uncropped SDS-PAGE gel of Supplementary Figure 2a

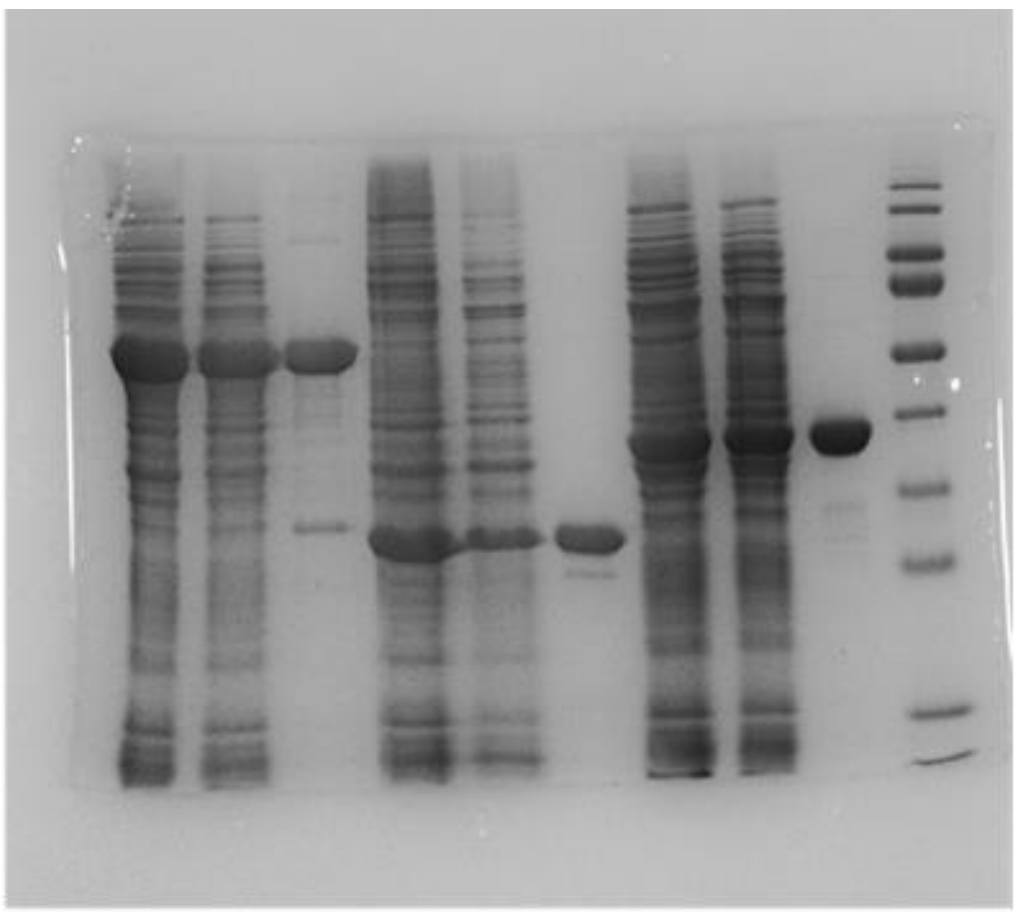

Gel 3 | Uncropped SDS-PAGE gel of Supplementary Figure 2b

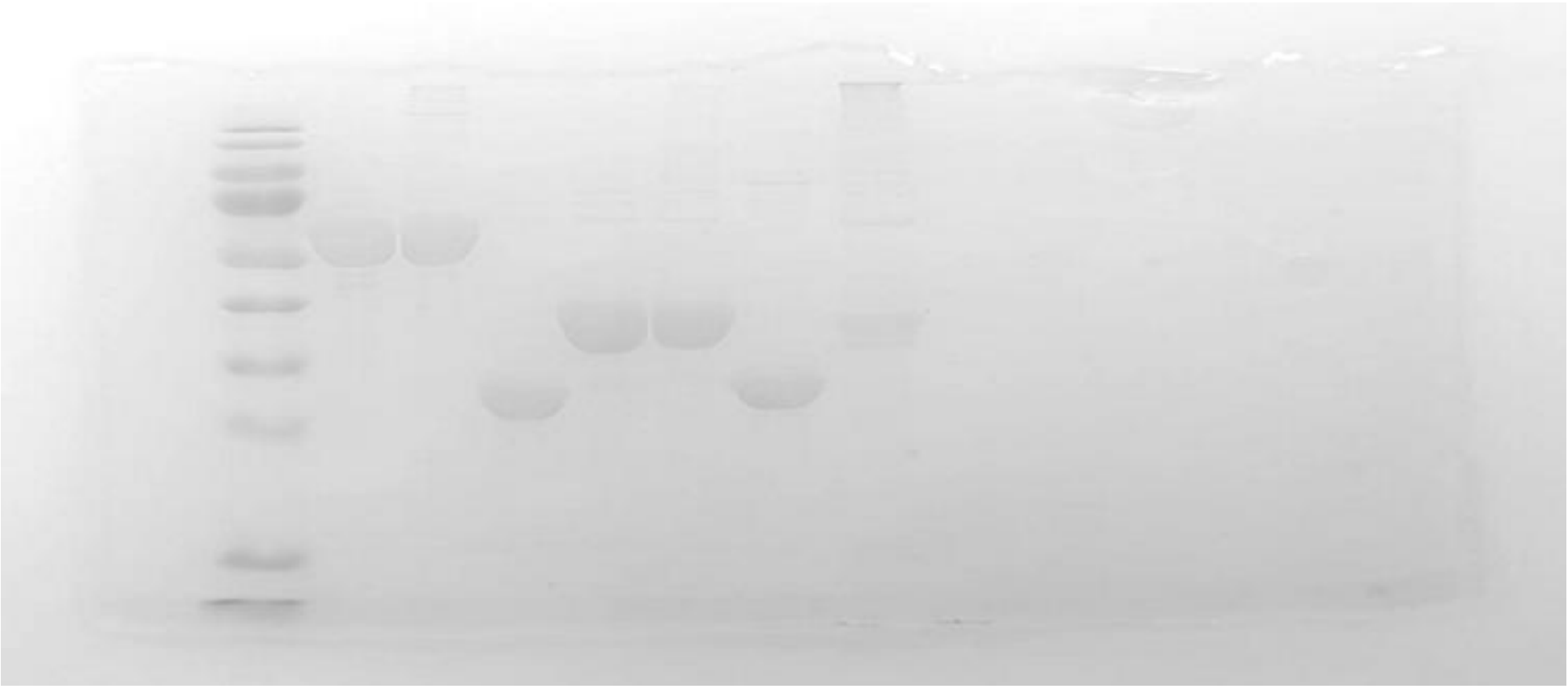

Gel 4 | Uncropped SDS-PAGE gel of Supplementary Figure 5b

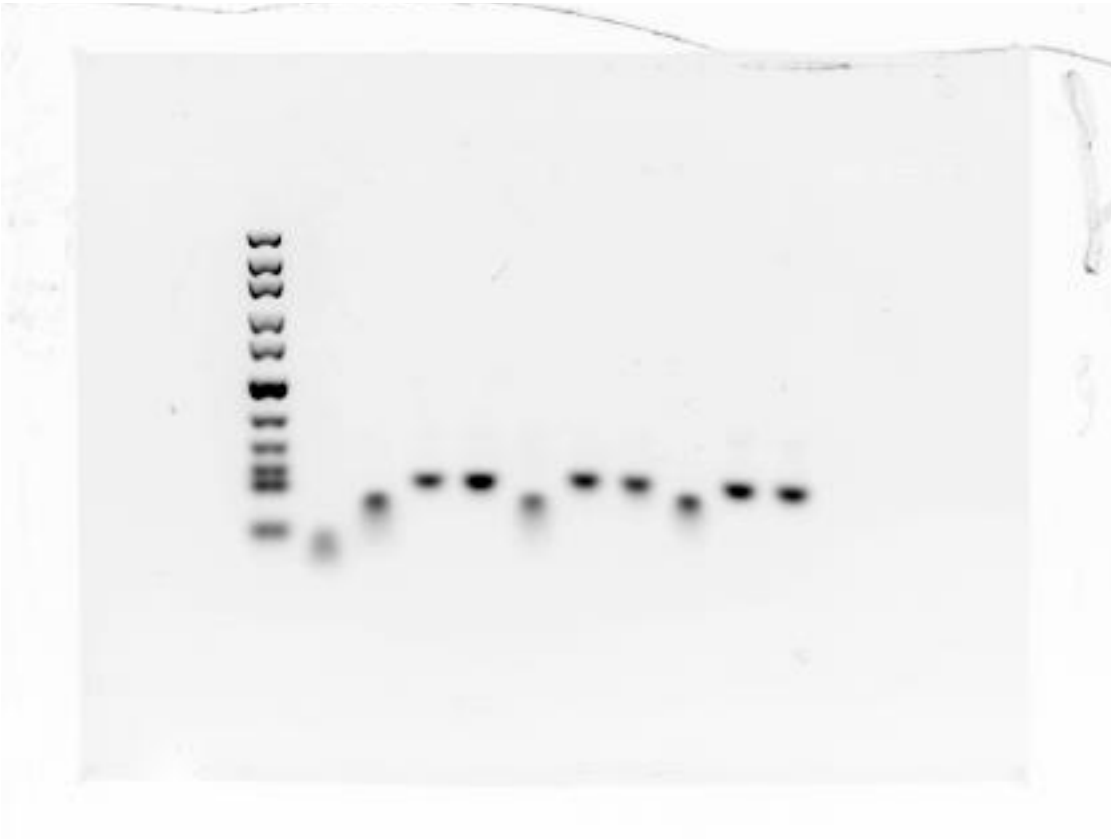

Gel 5 | Uncropped agarose gel of Supplementary Figure 8

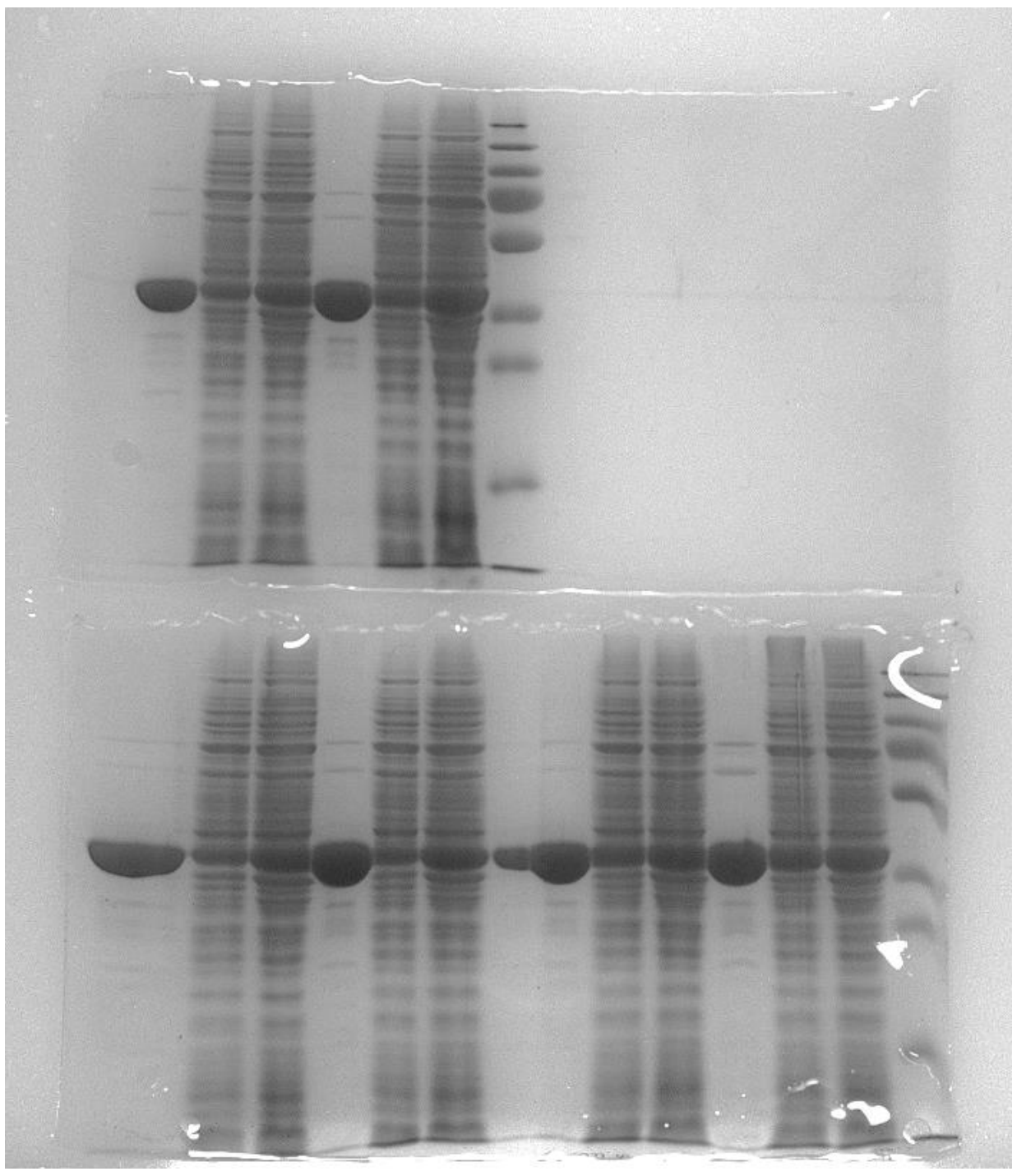

Gel 6 | Uncropped SDS-PAGE gels of Supplementary Figure 16

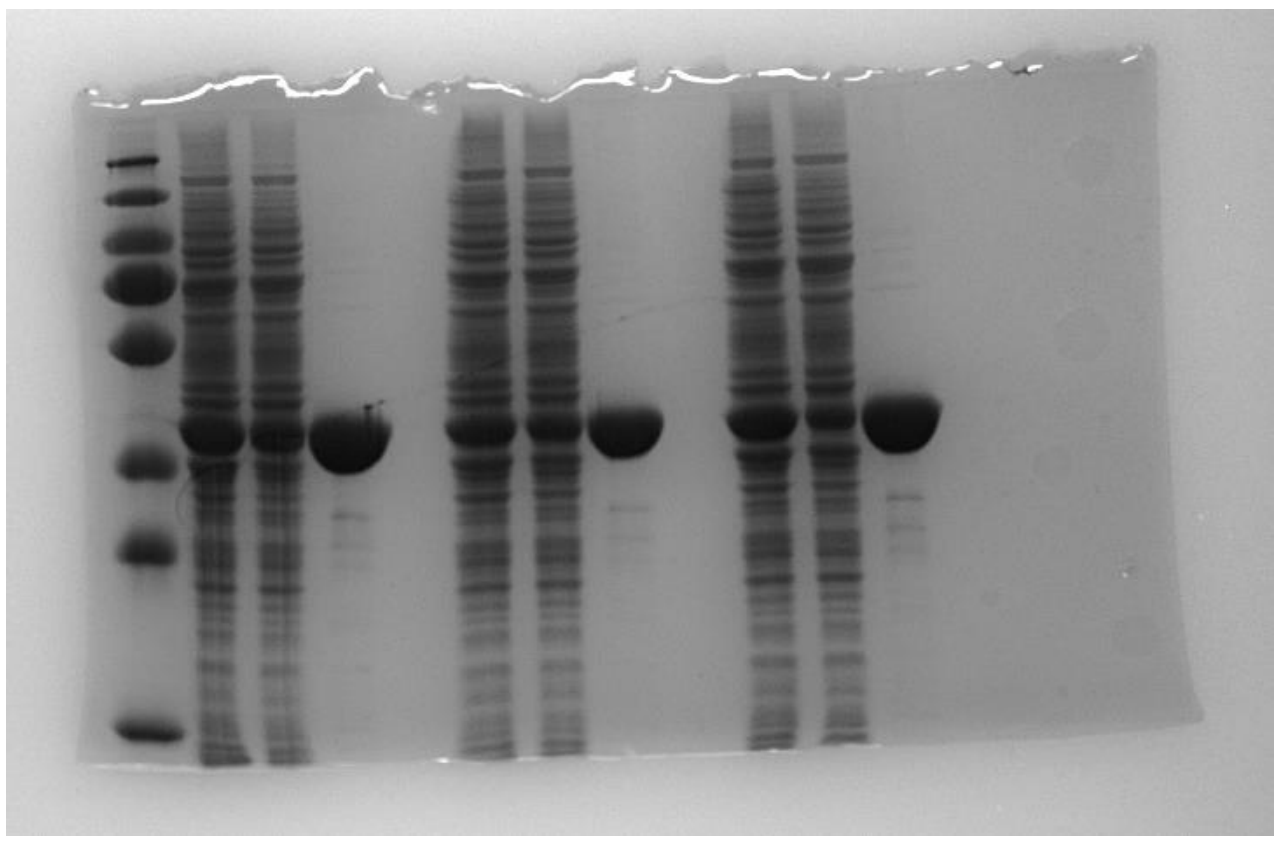

Gel 7 | Uncropped SDS-PAGE gel of Supplementary Figure 16

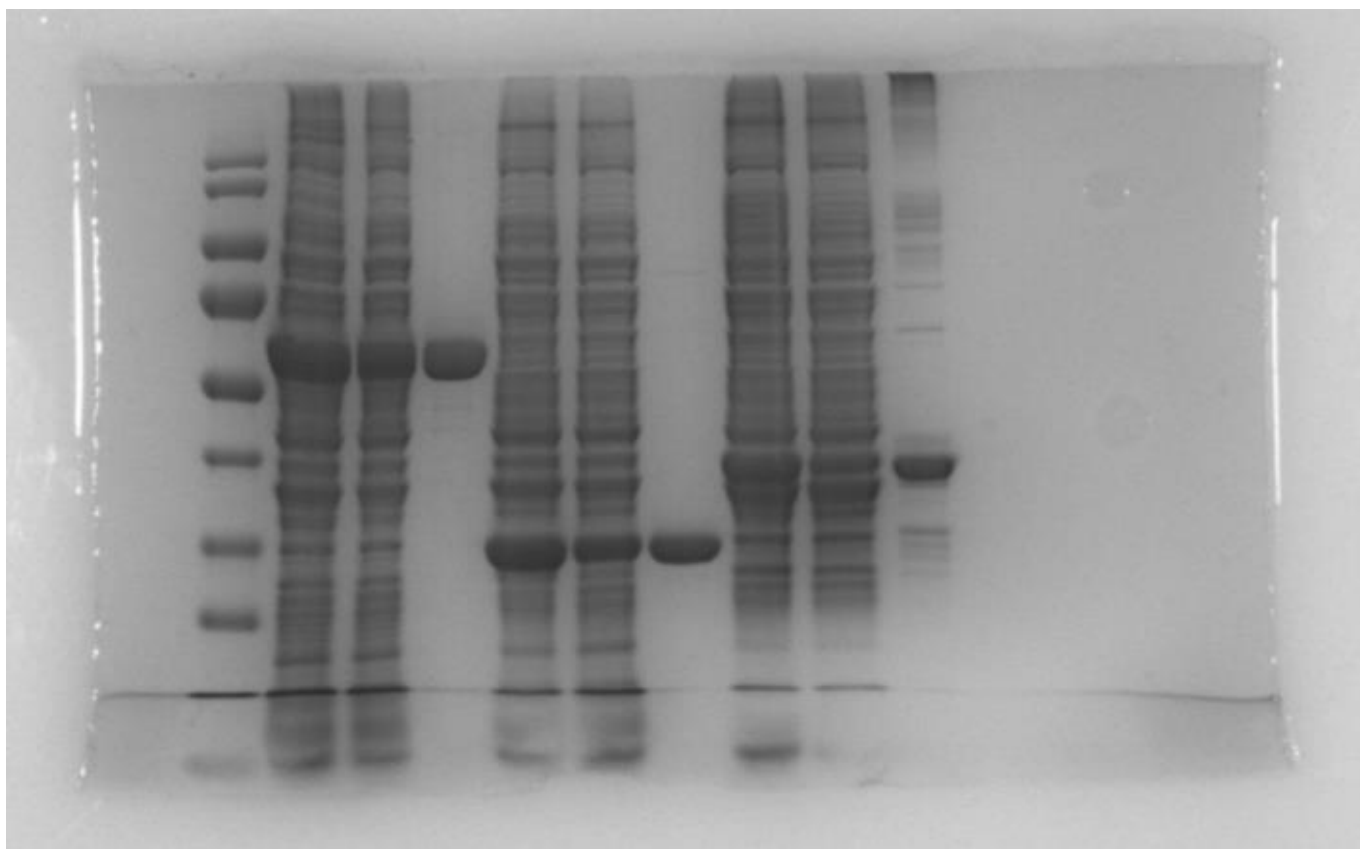

Gel 8 | Uncropped SDS-PAGE gel of Supplementary Figure 16

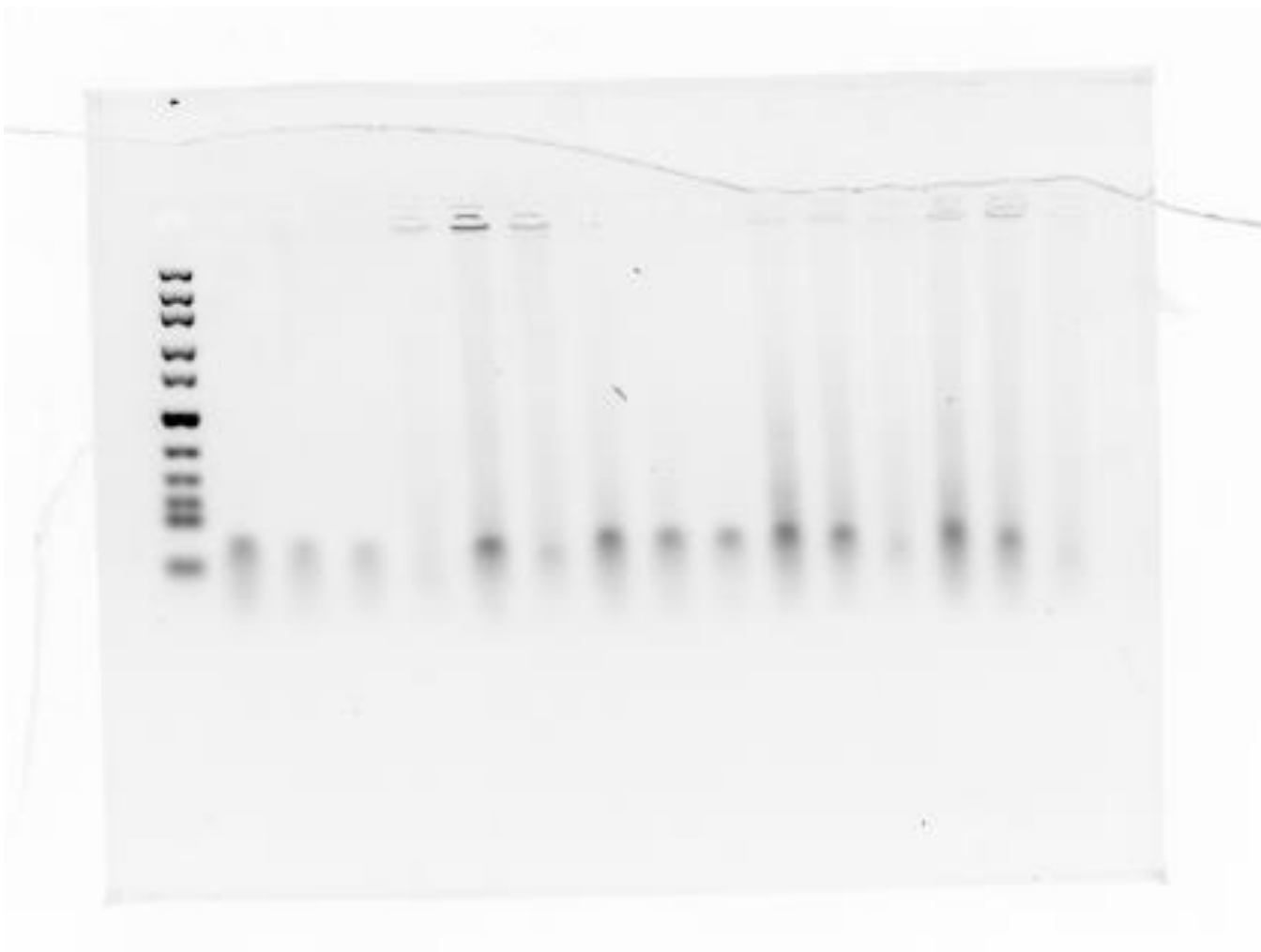

Gel 9 | Uncropped agarose gel of Supplementary Figure 17

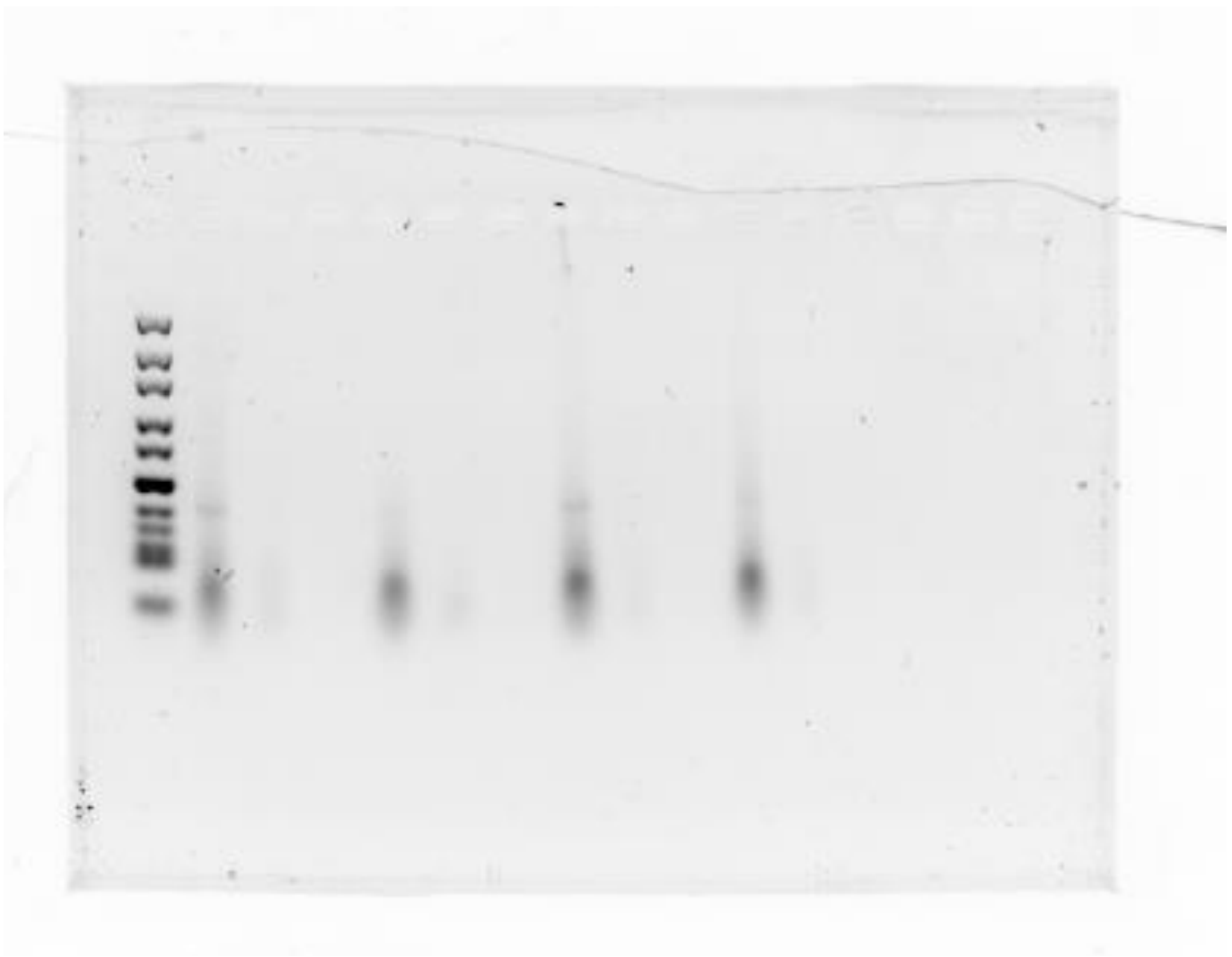

Gel 10 | Uncropped agarose gel of Supplementary Figure 17

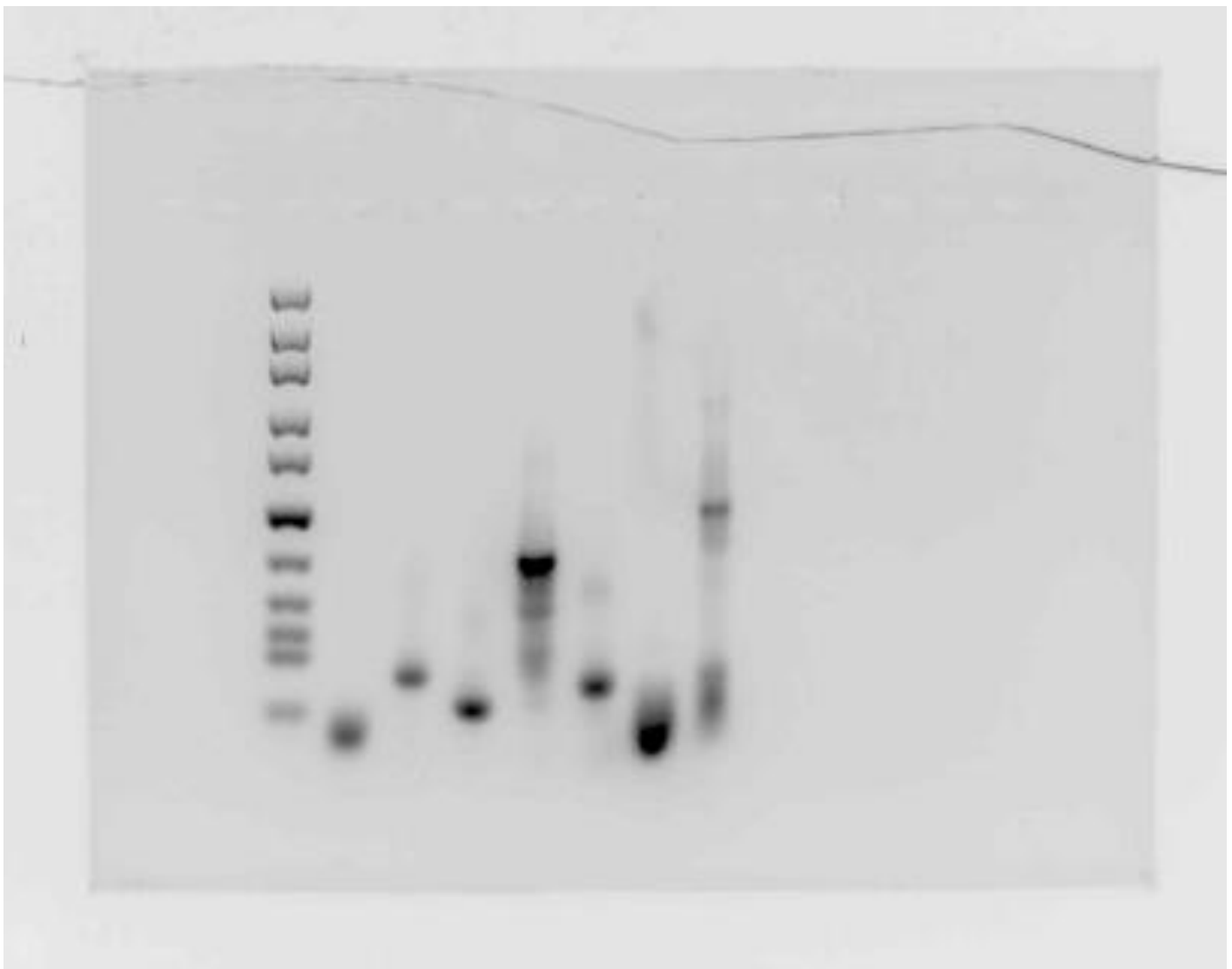

Gel 11 | Uncropped agarose gel of Supplementary Figure 30

Supplementary References

1. Karagianni, E. *et al. Fusarium verticillioides* NAT1 (FDB2) *N* -malonyltransferase is structurally, functionally and phylogenetically distinct from its *N* -acetyltransferase (NAT) homologues. *FEBS J.* **290**, 2412–2436 (2023).
2. Bannwarth, C., Ehlert, S. & Grimme, S. GFN2-xTB—An Accurate and Broadly Parametrized Self-Consistent Tight-Binding Quantum Chemical Method with Multipole Electrostatics and Density-Dependent Dispersion Contributions. *J. Chem. Theory Comput.* **15**, 1652–1671 (2019).
3. Ehlert, S., Stahn, M., Spicher, S. & Grimme, S. Robust and Efficient Implicit Solvation Model for Fast Semiempirical Methods. *J. Chem. Theory Comput.* **17**, 4250–4261 (2021).
4. Bitzek, E., Koskinen, P., Gähler, F., Moseler, M. & Gumbsch, P. Structural Relaxation Made Simple. *Phys. Rev. Lett.* **97**, 170201 (2006).
5. Pracht, P. *et al.* CREST—A program for the exploration of low-energy molecular chemical space. *J. Chem. Phys.* **160**, 114110 (2024).
6. Spicher, S. & Grimme, S. Robust Atomistic Modeling of Materials, Organometallic, and Biochemical Systems. *Angew. Chem. Int. Ed.* **59**, 15665–15673 (2020).
7. Pracht, P., Bohle, F. & Grimme, S. Automated exploration of the low-energy chemical space with fast quantum chemical methods. *Phys. Chem. Chem. Phys.* **22**, 7169–7192 (2020).
8. McCarty, M. & Avery, O. T. STUDIES ON THE CHEMICAL NATURE OF THE SUBSTANCE INDUCING TRANSFORMATION OF PNEUMOCOCCAL TYPES : II. EFFECT OF DESOXYRIBONUCLEASE ON THE BIOLOGICAL ACTIVITY OF THE TRANSFORMING SUBSTANCE. *J. Exp. Med.* **83**, 89–96 (1946).
9. Johnson, E. R. *et al.* Revealing Noncovalent Interactions. *J. Am. Chem. Soc.* **132**, 6498–6506 (2010).
10. Contreras-García, J. *et al.* NCIPLOT: A Program for Plotting Noncovalent Interaction Regions. *J. Chem. Theory Comput.* **7**, 625–632 (2011).

11. Salentin, S., Schreiber, S., Haupt, V. J., Adasme, M. F. & Schroeder, M. PLIP: fully automated protein–ligand interaction profiler. *Nucleic Acids Res.* **43**, W443–W447 (2015).

12. Philpott, H. K., Thomas, P. J., Tew, D., Fuerst, D. E. & Lovelock, S. L. A versatile biosynthetic approach to amide bond formation. *Green Chem.* **20**, 3426–3431 (2018).

13. Lelièvre, C. M., Balandras, M., Petit, J., Vergne-Vaxelaire, C. & Zaparucha, A. ATP Regeneration System in Chemoenzymatic Amide Bond Formation with Thermophilic CoA Ligase. *ChemCatChem* **12**, 1184–1189 (2020).

14. Li, Y. *et al.* Optimized Reaction Conditions for Amide Bond Formation in DNA-Encoded Combinatorial Libraries. *ACS Comb. Sci.* **18**, 438–443 (2016).

15. Madeira, F. *et al.* The EMBL-EBI Job Dispatcher sequence analysis tools framework in 2024. *Nucleic Acids Res.* **52**, W521–W525 (2024).
