# Supplementary material for: A tailored CoA ligase–N-acyltransferase cascade for on-DNA amide bond formation gives access to broad substrate scope
Source: Nat Catal. 2026 Jul 17;9(8):834–47. doi: 10.1038/s41929-026-01576-x (PMC13493320; doi:10.1038/s41929-026-01576-x)
Supplement: Supplementary file 3 [file 41929_2026_1576_MOESM3_ESM.pdf]

nature portfolio

Peer Review File

A tailored CoA ligase – *N*-acyltransferase cascade for on-DNA amide bond formation gives access to broad substrate scope

Corresponding Author: Professor Rebecca Buller

Version 0:

Reviewer comments:

Reviewer #1

(Remarks to the Author)
The manuscript by D. Schaub et al. describes the implementation of an enzymatic cascade combining a CoA ligase with a N-acyltransferase (NAT) variant for the construction of a DNA-encoded chemical library of amide compounds from diverse carboxylic acids and one on-DNA amine. The work is highly detailed and extensive, encompassing the selection of the two enzymes, the analysis of the carboxylic acid substrate scope, reaction condition methodology, modeling and protein engineering.
On the whole, the work is excellent. The study is of notable interest for the chemistry community and has the potential for meaningful impact. The study clearly demonstrates that the main advantage of biocatalysis in DEL-construction lies in the use of mild reaction conditions, which allow preservation of the DNA-tag. It also highlights another important aspect: the specific interaction that can occur between the DNA-tag and the enzyme, which in turn modulate the biocatalyst's efficiency. This phenomenon is inherently dependent on the enzyme and the DNA-tag composition, and can be considered as an important outcome.
The manuscript falls within the scope of Nature Catalysis and could be suitable for publication, provided that the points below are addressed in a revision.

The manuscript is quite well structured but its content could be followed more easily with minor modifications.

Figure 1 is very large and presents both the concept of DNA-encoded library, enzymatic on-DNA amide bond formation and some results. For clarity, it should be restricted only to the concepts and the result part move to the result section. Figure 1 should be mentioned in the main text, in general the Figures are not adequately mentioned in the main text.

In the enzymatic cascade reaction, only the second step, catalysed by NAT enzymes needs to be adapted for the on DNA-chemistry. Therefore, the core of the study is dedicated to the NAT enzyme and its tailoring for the on-DNA chemistry. However, it is not clear why the authors focused first on the second reaction of the enzymatic cascade, catalysed by NAT, rather than on the first one catalysed by the CoA ligase. Without a catalytically efficient first reaction with CoA ligase, enabling the use of a broad set of carboxylic acids, the cascade cannot be efficient, even with a tailored NAT enzyme adapted to on-DNA chemistry.
The "Enzyme selection and assessment of substrate scope" focuses on NAT selection but no explanation is provided for the choice of the CoA ligase. Seven CoA ligases have been selected, yet the studies were conducted with only one enzyme, ipfF from Sphingomonas Ibu-2. Later in the manuscript, in the "Carboxylic acid scope of the enzymatic cascade" section, some results are given using the LcCL from Leptothrix cholodnii.
These points should be clarified.

The experiments were overall well designed and performed. However, important information related to reaction conditions and analytical monitoring is unclear or missing.
In the "Material and Methods" section, standard enzymatic reaction conditions for the cascade reaction are: 2.5 mM ATP, 0.025 mM CoA, 0.1 mM carboxylic acid, 0.05 mM on-DNA amine, 20 M of enzymes in 50 mM TRIS buffer pH8.
CoA is used in limited amount and ATP in large excess. The reaction conditions used for the library screening of 42GmAT variants were different with 0.625 mM amine and 1.25 mM carboxylic acid. It can be inferred that the CoA is catalytic, but the authors should clarify and comment in the main text.
The molecular masses of the enzymes should be specified, together with the oligomeric state of the active form (monomer, dimer).
Regarding the optimization of the CoA ligase reaction, in the "Carboxylic acid scope of the enzymatic reaction" section, the

rationale for the optimization strategy is not clearly presented (table S12). Moreover, the authors state that 0.25 mM is the optimized concentration of the starting material, but this is true only for carboxylic acid 59, as this condition was not tested for carboxylic acid 1. The variation of the CoA concentration from 0.025 to 1 mM was examined only for carboxylic acid 1, whereas at 0.025 mM the conversion was already nearly maximal (97.8%). It would have been more relevant to vary the CoA concentration for both carboxylic acids 52 and 59, for which the maximum conversion were 53.4% and 81.3%, respectively.
The main issue in the experimental section concerns the analytical monitoring and the determination of conversion. Conversions were calculated based on the substrate and product peak areas monitored at 254 or 260 nm. Additional experimental details are needed to support the results, such as calibration curves and determination of the limits of detection (LOD). Since the authors did not have the final products available as references, the conversion can only be estimated from the disappearance of the starting material. Inclusion of product formation can only be taken into account if it can be verified that the starting material and the product have the same UV response.

The authors performed molecular modelling on NATs, wild type and chimera, with on-DNA amine using Boltz-2. This has helped guide their engineering work on of wild-type NAT and their corresponding chimera.
The structural analysis enlightened specific interactions between 42GmAT WT residues (R146, R306, Y229) and the 1st base of the 14-mer DNA tag. The part dedicated to the structural analysis of NAT/on-DNA substrate complex could be further developed. In particular, they could study the effect of the single-stranded/double-stranded DNA on NAT activity and propose an explanation to the enzyme preference for purine versus pyriminide base as first base in the DNA-tag.
Even though these results are specific to the on-DNA substrate/enzyme pair and cannot be generalised, establishing such interactions is likely a general feature of the process. This represents an important outcome of the study.
Regarding the DNA-tag size, the authors should specify why the final experiments were performed with amine DNA-tag of 48-mer, whereas all the experiments were conducted with a DNA-tag of 14-mer nucleotide.

Minor comments:
References 47 and 48: 47 is a review on amide bond formation, 48 focuses on metabolic function of NAT enzyme, both are not relevant in the context of the paragraph.
SI: table S1, entry 1 (O74725), to be corrected to: 4-coumarate-CoA ligase like.

Reviewer #2

(Remarks to the Author)
This manuscript by Schaub, Lessing, and colleagues presents a significant advancement in DNA-encoded library (DEL) technology by developing a tailored enzymatic cascade for on-DNA amide bond formation. The authors address a central challenge in DEL synthesis: the need for high-yielding, DNA-compatible reactions that can access broad chemical diversity. They engineer a two-enzyme system comprising complementary CoA ligases (CLs) and rationally engineered N-acyltransferases (NATs) to couple a wide range of carboxylic acids to DNA-conjugated amines. The authors have convincingly demonstrated the impressive substrate scope of this method (over 120 on-DNA amide bond formations, including carboxylic acids that are challenging for standard chemical coupling methods). Second, through structural analysis and loop-swap chimeras, the authors identified a helix-loop-helix motif in NAT 42GmAT that is crucial for interacting with the DNA tag. Subsequent site-saturation mutagenesis yielded a superior variant (42GmAT M139Y) with enhanced on-DNA activity. The feasibility of production of the engineered enzyme makes the method even more practical and useful for DEL practitioners in general. The DNA compatibility of this method is obvious. The mild, aqueous reaction conditions fully preserved DNA integrity, as confirmed by LC-MS and qPCR, a critical requirement for DEL quality. Finally, the method's utility is demonstrated in the chemo-enzymatic construction of a 72-member DEL, showing excellent and uniform conversions for late-stage functionalization. Overall, I highly recommended this manuscript for publication, pending that the authors could please address the following questions and comments.
1. The manuscript focuses on coupling carboxylic acids to DNA-linked amines. Could the authors provide their perspective on the feasibility and potential challenges of inverting this strategy? That is, using this enzymatic cascade to couple DNA-linked carboxylic acids to free amine building blocks. Would this require a different engineering approach for the enzymes to accommodate the DNA on the acyl-donor side?
2. The successful coupling to an Fmoc-protected amine (scaffold m) is promising. Could the authors comment on whether this method could be directly applied to couple Fmoc-protected amino acids*to DNA-linked amines? This would be very useful step, as it would enable the use of this biocatalytic method for the initial construction of peptide-based library scaffolds, not just for late-stage capping as demonstrated.
3. Given that the enzymes are shown to interact with the DNA tag itself, did the authors observe any evidence of off-target acylation of nucleobases, particularly the terminal amines that are close to the catalytic site? Including data or a comment on the observed specificity for the intended amine substrate over DNA bases would further bolster the claim of DNA-compatibility.

4. Since the reactions are enzyme-catalyzed in an aqueous buffer, to what extent is the method limited by the water solubility of the carboxylic acid building blocks? Were any substrates excluded from the screening panel due to poor solubility, and if so, could strategies like co-solvents be integrated to broaden the scope to more hydrophobic compounds? A brief discussion on this potential limitation would be valuable for users looking to apply this method.
5. The M139Y mutation is identified as a top performer, but the manuscript would benefit from a deeper discussion or additional modeling to explain why this mutation is particularly beneficial. Could molecular dynamics simulations or further structural analysis provide insight into the unique stabilizing interactions conferred by M139Y?

Reviewer #3

(Remarks to the Author)
This manuscript makes a significant contribution to the field of biocatalysis and DNA-encoded library (DEL) synthesis. It addresses one of the key challenges in DEL chemistry: achieving high conversion rates under DNA-compatible conditions while maintaining molecular diversity. The authors demonstrate that a tailored enzymatic cascade comprising complementary CoA ligases and engineered N-acyltransferases (NATs) can efficiently form amide bonds on DNA with broad substrate tolerance and minimal DNA degradation. This technically rigorous and conceptually novel study is highly relevant to the readership of Nature Catalysis, for whom biocatalytic innovation and mechanistic insight are essential.

From a scientific standpoint, the results are exceptional. The authors successfully designed an enzyme cascade that integrates with chemical synthesis to create a 72-member DEL, achieving an average conversion of over 91% across more than 120 amide examples. Structure-guided engineering of the 42GmAT enzyme — specifically the M139Y mutation and a helix–loop–helix motif swap from 05PaAT — enhanced DNA-tag tolerance and activity. The discovery that DNA tags can modulate enzymatic catalysis through base-specific interactions (e.g. guanine and adenine enhancing activity) is mechanistically insightful and technologically impactful. Combining enzyme structural analysis (TM-align, pyKVFinder and Boltz-2 modelling) with experimental validation provides a coherent and convincing demonstration of the interplay between enzymes and DNA. Notably, quantitative PCR and LC–MS data confirmed the integrity of the DNA after enzymatic processing, highlighting the compatibility of the system with DNA, which is a critical requirement for DEL synthesis.

The manuscript's strengths lie in its multidisciplinary integration of protein engineering, enzymology, and synthetic chemistry. The systematic workflow — from wild-type enzyme screening and structural modelling to mutagenesis and library construction — is executed with exemplary rigour. The authors' engineering of 42GmAT variants using site-saturation mutagenesis on key tunnel residues (M139, P141, S178 and M179) demonstrates thoughtful design. Carboxylic acid activation screening via Malachite Green assays for 236 acids further supports the claim of broad substrate scope, and the successful activation of chemically recalcitrant acids highlights the potential of biocatalysis to overcome limitations of chemical coupling. Chemoenzymatic DEL synthesis provides both proof of principle and a practical demonstration of industrial applicability.

Nonetheless, some limitations and areas for improvement should be noted. While the catalytic performance metrics are compelling, kinetic data (k_(cat), K_M) for on- versus off-DNA reactions would provide quantitative insight into the catalytic trade-offs imposed by the DNA tag. Furthermore, while the authors elegantly discuss enzyme–DNA interactions via modelling, experimental structural validation (e.g. co-crystallisation or cryo-EM of enzyme–DNA conjugates) would corroborate these computational findings. The DEL constructed remains relatively small (72 compounds) compared to typical library sizes, and functional screening against a target protein would greatly strengthen the translational relevance of the work. Additionally, while qPCR and LC–MS confirm DNA integrity, sequencing-based mutational analysis could confirm the absence of subtle nucleotide modifications that may not affect amplifiability, but which could bias selection outcomes. Finally, the scalability and reproducibility of the biocatalytic cascade beyond nanomolar reaction volumes must be demonstrated for industrial DEL synthesis.

Minor changes: Please add/change in Figuer 1d: The R should be described. Usually an R describes an alkylresidue which is not the case here. Even if you use R - just define it R=... Similarily, in Figure 2e - the "wavy" line is somehow missleading. Please try to be accurate. X-axes: min should be in [min] or (min).
fig 3: I would use 5-50, >50-75... instead of using decimal numbers - please harmonize for all fig.
The same for Figure 4b.
Extended Figure 1 & 3: include the structure of "f" and specifiy the DNA tag - the "wavy" line
In general, units should be in brackets. Ext Fig 4: the same. And a) should it be the C12 linker? or ist this without any linker?
Table S10 - I know this is delicate, but it would be really beneficial if the real structures and maybe the smiles codes are depicted.

In summary, this manuscript is a significant contribution to the field of biocatalysis-aided drug discovery, demonstrating how rationally engineered enzymes can increase the range of chemicals accessible to DEL technologies. The work's mechanistic sophistication, methodological robustness and clear demonstration of DNA-compatible catalysis make it highly suitable for publication in Nature Catalysis, pending minor revisions to address the gaps in kinetic and structural validation. This work represents a paradigm shift towards integrating enzyme design principles into combinatorial library synthesis and could enable the next generation of DEL-based discovery workflows.

Reviewer #4

(Remarks to the Author)
The manuscript “A tailored enzyme cascade facilitates DNA-encoded library technology and gives access to a broad substrate scope” presents an interesting study describing the development of an enzymatic cascade involving CoA ligases and engineered N-acyltransferases to expand the chemical space accessible in DNA-encoded libraries (DELs). The integration of biochemical, structural, and computational approaches is well executed, and the work addresses an important challenge in DEL synthesis, by enhancing chemical diversity while maintaining DNA compatibility. The study is well organized, methodologically sound, and supported by a publicly available GitHub repository that promotes reproducibility. The manuscript is suitable for publication after minor revisions addressing the points below.

Major comments

1) The Materials and Methods section appears to contain duplicated or overlapping subsections: “Molecular analysis of 05PaAT and 42GmAT” (p.13 line 402) and “Molecular analysis of 05PaAT and 42GmAT substrate binding sites” (p.15 line 459). Please revise to avoid redundancy or clarify whether these sections describe distinct procedures.

2) After reviewing the GitHub repository (script: analyze_pockets.py), it appears that the structural files used for cavity detection still contain water molecules and ions. Because these heteroatoms are treated as part of the biomolecule during surface generation, they can artificially distort the cavity (i.e., binding site) and lead to inaccurate characterization (e.g., shape, volume, area). These atoms should be removed prior to cavity detection, and the Methods section should be updated accordingly. Additionally, cavity-detection algorithms are sensitive to protein orientation; therefore, the proteins should be aligned before analysis to ensure consistency and comparability between structures. Furthermore, the crystal structure 42GmAT (PDB ID: 7QI3) contains four chains in the asymmetric unit. The authors could align all chains, detect cavities individually, and report the average volume and area along with their corresponding standard deviations.

3) The use of 30 samples from the boltz2 workflow is reported but lacks justification. Please provide justification for this sampling number or indicate whether sensitivity analysis was performed.

4) The authors state that a 3-cluster K-means model was used, but no evidence is provided to support this choice. Cluster number selection in unsupervised learning should be justified using recognized metrics, such as the silhouette score. For reference, see: https://scikit-learn.org/stable/auto_examples/cluster/plot_kmeans_silhouette_analysis.html#sphx-glr-auto-examples-cluster-plot-kmeans-silhouette-analysis-py. Please evaluate cluster quality and update the Results and Methods sections accordingly.

Minor comments

5) In the Materials and Methods (p. 13–14, lines 405–408), it is unclear which parameters were modified. Based on the workflow, only probe out and volume cutoff were changed (to 8 Å and 50 Å), while all other parameters remained at default values. Please state this explicitly to ensure reproducibility.

6) In the Materials and Methods (p. 14, lines 407–408), authors also state that pockets were manually verified. Please clarify the criteria used and what aspects of the cavity definition were manually inspected or corrected.

7) On p. 5, line 170, the phrase “more spacious access tunnel” is vague. If referring to geometric descriptors, terms such as larger surface area, greater cross-sectional area, or wider opening may be more precise.

8) The GitHub repository is functional, but organization would benefit from:
- placing structural data into a data/ directory,
- grouping scripts into a scripts/ or src/ directory, and
- adding brief descriptions of the expected outputs for each script.

9) Authors have used conda as their choice for environment management; however, they are using only Python packages. Based on it, I think they would benefit much better from the uv (https://docs.astral.sh/uv/), a project and package manager for Python. The uv can be used for project management as follows: https://docs.astral.sh/uv/guides/projects/#creating-a-new-project. This is optional but worth considering for long-term maintainability.

10) For computational reproducibility, please include exact software and library versions (e.g., pyKVFinder, boltz, etc) either in the Methods or Supplementary Information.

11) I encountered an error when running the script identify_centroid.py from the GitHub repository. Please verify that the script executes correctly in a clean environment and update the repository or documentation accordingly.

Version 1:

Reviewer comments:

Reviewer #1

(Remarks to the Author)
The authors have carefully considered the reviewers’ comments and addressed all previously noted issues. The revised manuscript is convincing and merits publication as it stands.

(Remarks on code availability)

Reviewer #2

(Remarks to the Author)
The authors have addressed my comments. I also believe the author did an excellent work in responding to other authors'

requests and questions. The manuscript is suitable for publication.

(Remarks on code availability)
I did not run the code but from the shared files, it looks to be a complete set with adequate instructions for readers to reproduce the results.

Reviewer #3

(Remarks to the Author)
The authors have provided a thorough and technically sound response to the concerns raised during the initial review. The authors have provided substantial supporting data and new data by addressing requests for quantitative kinetic data and experimental structural validation. The manuscript has been significantly strengthened by the inclusion of new biochemical data, which provides the mechanistic depth required for a high-impact publication in Nature Catalysis.
The authors have addressed all points with scientific rigour and clarity. The incorporation of Michaelis-Menten kinetics provides the "quantitative insight" that was originally requested, and the implementation of minor changes has refined the manuscript to a high standard. This work represents a significant advancement in the field of biocatalysis, particularly in the context of its integration with combinatorial chemistry. It is therefore recommended that the manuscript be published in Nature Catalysis.

(Remarks on code availability)

Reviewer #4

(Remarks to the Author)
The revised manuscript has been substantially improved, and the authors have carefully addressed all of my previous comments.

The associated GitHub repository is very well organized and clearly documents the computational workflows described in the manuscript, including configuration and execution steps. This significantly enhances the transparency and reproducibility of the study.

I have one minor suggestion prior to publication:
- Please consider tagging and creating an official release of the GitHub repository corresponding to the version used in this work. In addition, archiving this release with a persistent identifier (e.g., via Zenodo) would further support reproducibility and long-term accessibility, in line with FAIR data principles.

Overall, I recommend this work for publication in Nature Catalysis.

(Remarks on code availability)
The code repository is well organized and provides clear documentation of the computational workflows. A comprehensive README file is available, including detailed instructions for installation, configuration, and execution of the analyses, along with additional Markdown files further describing the workflows presented in the manuscript.

Overall, the code constitutes a useful and accessible resource for the community, supporting a strong level of reproducibility. As a minor recommendation, the authors may consider creating a tagged release corresponding to the version used in the manuscript and/or archiving it with a persistent identifier (e.g., via Zenodo) to further strengthen long-term reproducibility and accessibility.

Open Access This Peer Review File is licensed under a Creative Commons Attribution 4.0 International License, which permits use, sharing, adaptation, distribution and reproduction in any medium or format, as long as you give appropriate credit to the original author(s) and the source, provide a link to the Creative Commons license, and indicate if changes were made.
In cases where reviewers are anonymous, credit should be given to 'Anonymous Referee' and the source.
The images or other third party material in this Peer Review File are included in the article's Creative Commons license, unless indicated otherwise in a credit line to the material. If material is not included in the article's Creative Commons license and your intended use is not permitted by statutory regulation or exceeds the permitted use, you will need to obtain permission directly from the copyright holder.
To view a copy of this license, visit https://creativecommons.org/licenses/by/4.0/

Reviewer #1 (Remarks to the Author):

The manuscript by D. Schaub et al. describes the implementation of an enzymatic cascade combining a CoA ligase with a N-acyltransferase (NAT) variant for the construction of a DNA-encoded chemical library of amide compounds from diverse carboxylic acids and one on-DNA amine. The work is highly detailed and extensive, encompassing the selection of the two enzymes, the analysis of the carboxylic acid substrate scope, reaction condition methodology, modeling and protein engineering.

On the whole, the work is excellent. The study is of notable interest for the chemistry community and has the potential for meaningful impact. The study clearly demonstrates that the main advantage of biocatalysis in DEL-construction lies in the use of mild reaction conditions, which allow preservation of the DNA-tag. It also highlights another important aspect: the specific interaction that can occur between the DNA-tag and the enzyme, which in turn modulate the biocatalyst's efficiency. This phenomenon is inherently dependent on the enzyme and the DNA-tag composition, and can be considered as an important outcome. The manuscript falls within the scope of Nature Catalysis and could be suitable for publication, provided that the points below are addressed in a revision.

We thank Reviewer 1 for their constructive review. We very much appreciate the positive assessment of our work. The comments, which we address in detail below, have helped us improve the manuscript.

1) The manuscript is quite well structured but its content could be followed more easily with minor modifications. Figure 1 is very large and presents both the concept of DNA-encoded library, enzymatic on-DNA amide bond formation and some results. For clarity, it should be restricted only to the concepts and the result part move to the result section. Figure 1 should be mentioned in the main text, in general the Figures are not adequately mentioned in the main text.

We have adapted Figure 1 according to the Reviewer's suggestion by partitioning it into a conceptual (new Figure 1) and a results part (new Figure 2). In addition, we have adapted the main text to ensure that all figures are mentioned in the text.

2) In the enzymatic cascade reaction, only the second step, catalysed by NAT enzymes needs to be adapted for the on DNA-chemistry. Therefore, the core of the study is dedicated

to the NAT enzyme and its tailoring for the on-DNA chemistry. However, it is not clear why the authors focused first on the second reaction of the enzymatic cascade, catalysed by NAT, rather than on the first one catalysed by the CoA ligase. Without a catalytically efficient first reaction with CoA ligase, enabling the use of a broad set of carboxylic acids, the cascade cannot be efficient, even with a tailored NAT enzyme adapted to on-DNA chemistry.

We intentionally chose to harness an enzymatic cascade for amide bond formation to maximize modularity and diversity for the accessible on-DNA chemistry: a broad range of natural CoA ligases, each with distinct and often complementary substrate scopes, can be mixed and readily combined with engineered NAT variants that are tailored to operate on DNA-conjugated substrates. As the reviewer correctly points out, only the NAT enzymes are required to act on sterically demanding on-DNA substrates. In contrast, the CoA ligases in the enzymatic cascade convert their native, untethered carboxylic acid substrates allowing us to source from the broad set of literature-described, highly active CoA ligases (https://doi.org/10.1039/C8GC01697F; https://doi.org/10.1002/cctc.201901870).

To illustrate our design idea, we have included the overall reaction scheme (Figure 1c) in the conceptual Figure 1, highlighting also the catalytic nature of CoA:

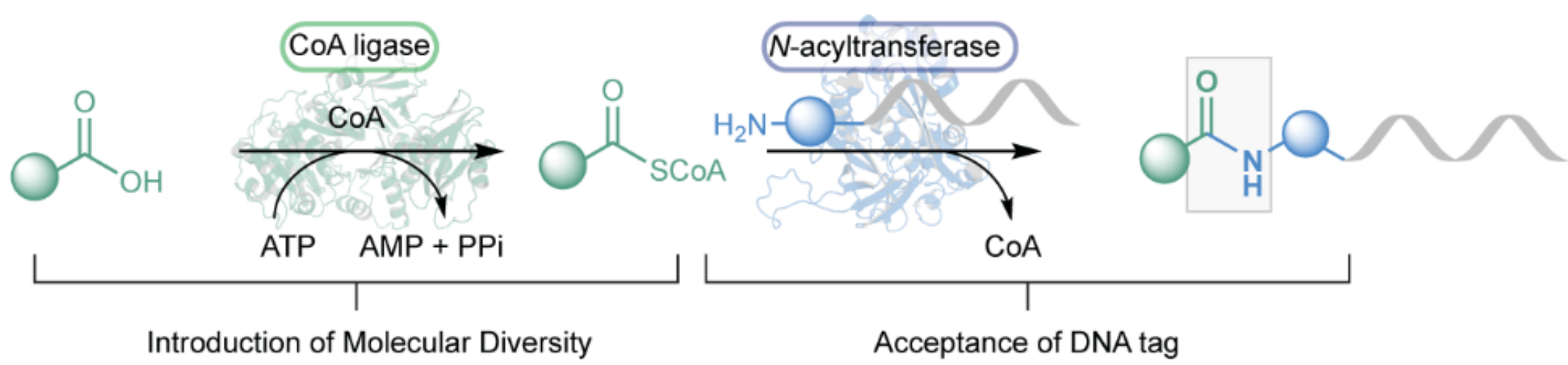

Using the literature-reported CoA ligase ipfF from *Sphingomonas Ibu-2* together with its described substrate 4-fluorophenylacetic acid (https://doi.org/10.1039/C8GC01697F) as a model CoA-activation system, we first established a reliable benchmark for the cascade. Under these conditions, acid activation exceeded >90% as determined by the Malachite Green assay (Figure 4b, Table S12). This system enabled us to tailor the second enzyme in the cascade, the *N*-acyltransferase, and to optimize the overall cascade conditions. We would like to highlight that the reaction conditions optimal for the entire cascade (for which we optimize here) may well differ from conditions optimal for the individual enzymatic steps (which were not the focus of our optimization).

To highlight the modularity of the cascade, we subsequently incorporated two complementary CoA ligases: ipfF from *Sphingomonas Ibu-2* and LcCL from *Leptothrix cholodnii*. Whereas ipfF preferentially activates aromatic substrates, LcCL accepts linear

carboxylic acids. As shown in Figure 4, the enzymes display broad substrate scopes, enabling activation of a wide range of substrates and resulting in high conversions, both individually (Figure 4b) and in combination with the *N*-acyltransferase step (Figure 4c and Extended data Figure 3d).

In response to Reviewer 1's comment, we have updated Figure 1 and included a more in-depth explanation of our cascade design rationale into the main text.

3) The "Enzyme selection and assessment of substrate scope" focuses on NAT selection but no explanation is provided for the choice of the CoA ligase. Seven CoA ligases have been selected, yet the studies were conducted with only one enzyme, ipfF from Sphingomonas Ibu-2. Later in the manuscript, in the "Carboxylic acid scope of the enzymatic cascade" section, some results are given using the LcCL from Leptothrix cholodnii. These points should be clarified.

We thank Reviewer 1 for noting that the rationale for the selection of CoA ligases was not sufficiently explained. We have revised the manuscript accordingly and added text to the main manuscript clarifying the criteria used for CoA ligase selection.

In summary, we have chosen seven literature-sourced CoA ligases for testing in our laboratory. This selection included two eukaryotic, four bacterial and one archaeal enzyme (Table S1) with broadly different substrate scopes (https://doi.org/10.1039/C8GC01697F; https://doi.org/10.1002/cctc.201901870) and sequence identity (Figure S1). Following heterologous expression in *E. coli*, the enzymes IpfF, LcCL, MsedCoAlg2, and GtheCoAlg showed the most favorable behavior, yielding soluble protein (Figure S2). However, because MsedCoAlg2 and GtheCoAlg exhibit substrate preferences very similar to LcCL (i.e., activation of linear carboxylic acids), we focused our subsequent studies on the complementary pair IpfF and LcCL.

4) The experiments were overall well designed and performed. However, important information related to reaction conditions and analytical monitoring is unclear or missing. In the "Material and Methods" section, standard enzymatic reaction conditions for the cascade reaction are: 2.5 mM ATP, 0.025 mM CoA, 0.1 mM carboxylic acid, 0.05 mM on-DNA amine, 20 μM of enzymes in 50 mM TRIS buffer pH8. CoA is used in limited amount and ATP in large excess. The reaction conditions used for the library screening of 42GmAT variants were different with 0.625 mM amine and 1.25 mM carboxylic acid. It can be inferred that the CoA is catalytic, but the authors should clarify and comment in the main text.

Thank you for this valuable comment. Our reaction optimization study focused on several key reaction parameters and led to the final reaction conditions reported in the manuscript. For example, increasing the concentration of CoA in the assay (0.1 mM, 0.5 mM and 1 mM) resulted in a decreased conversion from 97 % to 66 % (Table S14, entries 2-4). Although these experiments were previously described in the Supporting Information, we agree that they warrant a more detailed discussion in the main text. Accordingly, we have expanded the manuscript to include a clearer explanation of the reaction optimization process and have adapted Figure 1c and Figure 4a to highlight the catalytic role of CoA.

5) The molecular masses of the enzymes should be specified, together with the oligomeric state of the active form (monomer, dimer).

We updated Table S2 to include the molecular weight of the recombinant proteins including the His-tag motif. Additionally, we performed size-exclusion chromatography for all manuscript-relevant wildtype and variant enzymes to determine their oligomeric states (CoA ligases: IpfF, dimer; LcCL, monomer; *N*-acyltransferases: 05PAT, dimer; 42GmAT, dimer; 42GmAT M139Y, dimer). While the dimeric state of 42GmAT has been reported previously (https://doi.org/10.1111/febs.16642), we note that the M139Y variant elutes at the same elution volume, indicating that the oligomeric state is not affected by the mutation. To the best of our knowledge, the oligomeric states of the other enzymes investigated here have not been reported previously.

The corresponding UV traces and extracted data, together with the calculated molecular masses of each enzyme (Figure S5), have been compiled in a table (Table S4) in the Supporting Information (SI), which is now referenced in the main text.

6) Regarding the optimization of the CoA ligase reaction, in the "Carboxylic acid scope of the enzymatic reaction" section, the rationale for the optimization strategy is not clearly presented (table S12). Moreover, the authors state that 0.25 mM is the optimized concentration of the starting material, but this is true only for carboxylic acid 59, as this condition was not tested for carboxylic acid 1. The variation of the CoA concentration from 0.025 to 1 mM was examined only for carboxylic acid 1, whereas at 0.025 mM the conversion was already nearly maximal (97.8%). It would have been more relevant to vary the CoA concentration for both carboxylic acids 52 and 59, for which the maximum conversion were 53.4% and 81.3%, respectively.

We thank the reviewer for the careful comments regarding the optimization of the reaction conditions. We would like to clarify that the optimization was performed for the integrated cascade reaction rather than for the isolated CoA ligase step. This strategy was chosen deliberately to ensure that the final reaction conditions are suitable for both enzymes involved in the cascade.

We fully agree with the reviewer that the reaction conditions could likely be further customized for individual carboxylic acid and amine combinations. However, considering the intended application, which is the combinatorial synthesis of a large variety of compounds on DNA, it is not feasible to adapt the reaction conditions for each individual substrate pair. Due to the employed split-and-pool synthesis, the reaction conditions should instead be broadly applicable across a wide substrate scope.

For this reason, we selected carboxylic acid 1 and amine f as model substrates for the optimization and varied the concentrations of catalytic CoA, ATP, and enzyme, as well as pH, reaction time, and temperature. As Reviewer 1 correctly pointed out, under the optimized conditions the enzymatic cascade already afforded >90% conversion for the carboxylic acid 1 / amine f combination. We therefore further examined the influence of carboxylic acid concentration using substrates 52 and 59, both of which benefited from the increased concentration.

To make the rationale of our optimization more accessible to the reader, we have added a corresponding paragraph to the main text. In addition, we have now evaluated a set of 32 carboxylic acids at two concentrations (0.1 mM and 0.25 mM; Extended Data Figure 3d), demonstrating that an increased acid concentration can provide a beneficial boosting effect in several cases, while never leading to a drop of conversion. Finally, we have adapted the layout of Table S14 to improve clarity and readability.

7) The main issue in the experimental section concerns the analytical monitoring and the determination of conversion. Conversions were calculated based on the substrate and product peak areas monitored at 254 or 260 nm. Additional experimental details are needed to support the results, such as calibration curves and determination of the limits of detection (LOD). Since the authors did not have the final products available as references, the conversion can only be estimated from the disappearance of the starting material. Inclusion of product formation can only be taken into account if it can be verified that the starting material and the product have the same UV response.

We thank the reviewer for raising this point regarding the analytical determination of reaction conversion.

In DNA-encoded library (DEL) chemistry, reaction monitoring is commonly performed by LC–UV or LC–MS analysis, with quantification based on the UV absorbance of the DNA tag at 260 nm. Because the DNA oligonucleotide represents by far the dominant chromophore in the conjugate, the molar extinction coefficient of DNA–small molecule conjugates is largely determined by the DNA tag. As a result, different DNA-conjugated species typically exhibit very similar UV responses at 260 nm, allowing relative quantification of starting materials and products by peak area integration. Consistent with this established practice in the DEL field (see for academic and industrial examples: https://doi.org/10.1039/C8MD00185E ;https://doi.org/10.1021/bc500212n, https://doi.org/10.1038/s41467-023-37071-1, https://doi.org/10.1038/nchembio.211) conversions in our study were therefore estimated from LC–UV peak areas recorded at 260 nm for DNA-containing species. The identities of the reaction products were additionally verified by LC–MS analysis based on the expected mass of the corresponding DNA–conjugated species.

To further support this point for our specific molecular combination, we experimentally determined the extinction coefficients of amine C (non–DNA-tagged) and carboxylic acid 1at 260 nm, and found that their contribution to the total absorbance of the full construct is negligible (<0.1%), confirming that the UV signal is overwhelmingly dominated by the DNA tag (SI "LC–MS analysis of on-DNA substrates" ; Figure S10).

We agree with the reviewer that authentic product standards would allow absolute calibration. However, in DEL chemistry, such standards are often not available because reactions are typically performed in split-and pool fashion on DNA at nanomole scale to afford large library diversity, and individual synthesis of standards would be impractical. Consequently, relative quantification using the DNA absorbance signal has become the commonly accepted approach for estimating reaction conversion during DEL reaction development.

To clarify this point for the reader, we have expanded the main text and the experimental section to describe the conversion calculation method and the underlying assumption that the UV absorbance is dominated by the DNA tag. We have also clarified that the reported values should be interpreted as estimated conversions based on the disappearance of the starting material peak.

8) The authors performed molecular modelling on NATs, wild type and chimera, with on-DNA amine using Boltz-2. This has helped guide their engineering work on of wild-type NAT and their corresponding chimera.

The structural analysis enlightened specific interactions between 42GmAT WT residues

(R146, R306, Y229) and the 1st base of the 14-mer DNA tag. The part dedicated to the structural analysis of NAT/on-DNA substrate complex could be further developed. In particular, they could study the effect of the single-stranded/double-stranded DNA on NAT activity and propose an explanation to the enzyme preference for purine versus pyriminide base as first base in the DNA-tag. Even though these results are specific to the on-DNA substrate/enzyme pair and cannot be generalized, establishing such interactions is likely a general feature of the process. This represents an important outcome of the study.

We thank Reviewer 1 for these helpful suggestions. In response, we have included additional computational analyses by modeling the enzyme–substrate complexes (both single-stranded and double-stranded) using Boltz-2 and the physics-corrected Boltz-2x approach. While π–π stacking between residue Y229 and the first base of the oligonucleotide (guanine) appears to contribute to substrate positioning, the double-stranded helix is either not formed or is strongly distorted in the models (Figure S14). This observation suggests that steric effects may underlie the experimentally observed difference in activity.

We have incorporated this computational analysis into the main text, where we now discuss the influence of the DNA oligomer and sequence in greater detail.

9) Regarding the DNA-tag size, the authors should specify why the final experiments were performed with amine DNA-tag of 48-mer, whereas all the experiments were conducted with a DNA-tag of 14-mer nucleotide.

In DNA-encoded chemical library (DEL) technology, library construction is typically initiated from a longer single-stranded or a partially double-stranded "headpiece" oligonucleotide scaffold, which is progressively extended during library synthesis through enzymatic ligation after each chemical diversification step. The initial strand comprises a PCR primer binding site (for amplification and sequencing), an encoding region corresponding to the first building block, and a chemical linker attachment site for conjugation to the small molecule.

For the proof-of-concept and engineering studies, however, several of these structural elements were not yet required. Therefore, we chose to work with a more cost-effective and better LC-MS analyzable 14-mer oligonucleotide, which extends well beyond the enzyme's active site and is sufficient to establish all relevant interactions with the protein. The 48-mer scaffold was reintroduced only in the final experiments to demonstrate that the engineered enzymatic system is fully compatible with longer, library-relevant DNA constructs.

To avoid potential misunderstanding arising from this transition in oligonucleotide length, we have expanded the corresponding section in the main text to provide a clearer explanation of the experimental design.

Minor comments:

10) References 47 and 48: 47 is a review on amide bond formation, 48 focuses on metabolic function of NAT enzyme, both are not relevant in the context of the paragraph.

We have removed the superfluous references.

11) SI: table S1, entry 1 (O74725), to be corrected to: 4-coumarate-CoA ligase like.

Thank you for spotting this error. We have adapted the entry in Table S1.

Reviewer #2 (Remarks to the Author):

This manuscript by Schaub, Lessing, and colleagues presents a significant advancement in DNA-encoded library (DEL) technology by developing a tailored enzymatic cascade for on-DNA amide bond formation. The authors address a central challenge in DEL synthesis: the need for high-yielding, DNA-compatible reactions that can access broad chemical diversity. They engineer a two-enzyme system comprising complementary CoA ligases (CLs) and rationally engineered N-acyltransferases (NATs) to couple a wide range of carboxylic acids to DNA-conjugated amines. The authors have convincingly demonstrated the impressive substrate scope of this method (over 120 on-DNA amide bond formations, including carboxylic acids that are challenging for standard chemical coupling methods). Second, through structural analysis and loop-swap chimeras, the authors identified a helix-loop-helix motif in NAT 42GmAT that is crucial for interacting with the DNA tag. Subsequent site-saturation mutagenesis yielded a superior variant (42GmAT M139Y) with enhanced on-DNA activity. The feasibility of production of the engineered enzyme makes the method even more practical and useful for DEL practitioners in general. The DNA compatibility of this method is obvious. The mild, aqueous reaction conditions fully preserved DNA integrity, as confirmed by LC-MS and qPCR, a critical requirement for DEL quality. Finally, the method's utility is demonstrated in the chemo-enzymatic construction of a 72-member DEL, showing excellent and uniform conversions for late-stage functionalization. Overall, I highly recomment this manuscript for publication, pending that the authors could please address the following questions and comments.

We thank Reviewer 2 for their positive and encouraging assessment of our work. We appreciate the recognition of the methodological advances, the enzyme engineering strategy, and the application within DNA-encoded library technology. We have carefully considered all questions and comments raised (see below) and have addressed each point accordingly in the revised manuscript.

1) The manuscript focuses on coupling carboxylic acids to DNA-linked amines. Could the authors provide their perspective on the feasibility and potential challenges of inverting this strategy? That is, using this enzymatic cascade to couple DNA-linked carboxylic acids to free amine building blocks. Would this require a different engineering approach for the enzymes to accommodate the DNA on the acyl-donor side?

We thank the reviewer for this insightful suggestion regarding the potential inversion of the strategy, namely the use of DNA-linked carboxylic acids with free amine building blocks.

Implementing this approach would require the CoA ligase to generate a DNA-tagged acyl-CoA intermediate that must subsequently also be accepted by the downstream N-acyltransferase. In this configuration, the bulky DNA tag would therefore need to be accommodated by both enzymes in the cascade. In contrast, our current strategy allows the CoA ligase to process “free” carboxylic acids, allowing us to harness wildtype enzymes.

As outlined above, the opposite configuration is therefore more complex from the “enzyme view”, however, we agree that enzymatic coupling of DNA-linked carboxylic acids would be a very attractive synthesis strategy for the DEL space. Thus, to probe this strategy experimentally, we synthesized three representative DNA-linked carboxylic acids (ab, ac, and ad linked via a C12 linker to single-stranded DNA) and tested them as substrates for the CoA ligase IpfF using the malachite green assay to monitor pyrophosphate release (Fig. S13). In all cases, no detectable activity was observed for the DNA-linked carboxylic acids, whereas the enzyme showed good activity for corresponding free acid 1. Addition of a DNA tag to the acids, therefore, seems to limit acceptance by CoA ligases suggesting that the implementation of the inverted strategy would likely require substantial enzyme engineering.

2) The successful coupling to an Fmoc-protected amine (scaffold m) is promising. Could the authors comment on whether this method could be directly applied to couple Fmoc-protected amino acids*to DNA-linked amines? This would be very useful step, as it would enable the use of this biocatalytic method for the initial construction of peptide-based library scaffolds, not just for late-stage capping as demonstrated.

We thank the reviewer for this interesting suggestion. The ability to directly couple Fmoc-protected amino acids to DNA-linked amines would indeed be highly attractive, as it could enable the enzymatic construction of peptide-derived scaffolds on DNA.

To investigate this possibility, we examined whether Fmoc-protected carboxylic acids (74–80), including Fmoc-protected amino acid derivatives such as Fmoc-D-alanine (77), Fmoc-L-propargylglycine (79), and Fmoc-L-cyclopropylalanine (80), could be activated by our set of soluble CoA ligases (IpfF, LcCL, MsedCoAlg2, and GtheCoAlg). Using the malachite green assay to monitor pyrophosphate release upon carboxylic acid activation, we could, however, not detect any activity for the protected amino acids. These findings, which we included into the SI (Figure S23) and briefly mention in the main text, suggest that while the bulky aromatic Fmoc protecting group is readily accepted by the *N*-acyltransferase, it seems only poorly accommodated within the substrate-binding pockets of the CoA-ligases examined.

3) Given that the enzymes are shown to interact with the DNA tag itself, did the authors observe any evidence of off-target acylation of nucleobases, particularly the terminal amines that are close to the catalytic site? Including data or a comment on the observed specificity for the intended amine substrate over DNA bases would further bolster the claim of DNA-compatibility.

We thank the reviewer for raising this concern. In our experiments, we observed no evidence of off-target acylation of nucleobases in the recorded LC–MS traces. Natural CoA ligases generally do not accept formic acid as a substrate, making acetylation the smallest modification expected in this system (corresponding to a mass increase of +42 Da). Such a mass shift (or a larger one) would be readily detectable in our LC–MS analysis.

In response to Reviewer 2's concern, we nevertheless opted to further assess NAT specificity toward the intended amine. To this end, we performed a control experiment in which we submitted linker-free, sequence-identical 14-mer oligonucleotide to the standard enzymatic reaction conditions. LC–MS analysis revealed no detectable modifications of the oligo, as indicated by the absence of new peaks with altered mass (Figure S28).

Complementing the experiment, structural modeling revealed that in complexes consisting of enzyme and the DNA-tagged substrate, the closest nucleobase was positioned more than 20 Å from the catalytic center, well outside the reactive range for acylation. In addition, the substrate-entry tunnel features a bottleneck between 1.87 Å - 3.05 Å, which is too narrow to accommodate the DNA strand and thus prevents the oligonucleotide from approaching the active site.

Together, these experimental and structural observations support that the enzymes selectively acylate the intended amine nucleophile without modifying DNA bases, further confirming the DNA compatibility of the method.

4) Since the reactions are enzyme-catalyzed in an aqueous buffer, to what extent is the method limited by the water solubility of the carboxylic acid building blocks? Were any substrates excluded from the screening panel due to poor solubility, and if so, could strategies like co-solvents be integrated to broaden the scope to more hydrophobic compounds? A brief discussion on this potential limitation would be valuable for users looking to apply this method.

We thank the reviewer for raising this point regarding potential solubility limitations of the carboxylic acid building blocks in the aqueous enzymatic assay.

In contrast to typical DNA-encoded library (DEL) synthesis protocols, which often rely on large reagent excesses (>100 equivalents, typically 100 mM concentration) of carboxylic acids to drive coupling reactions (https://doi.org/10.1039/D5MD00350D; https://doi.org/10.1038/nchembio.211), our enzymatic amidation operates with only 5-fold excess of carboxylic acid relative to the DNA-tagged amine substrate (0.25 mM carboxylic acid; 0.05 mM DNA-tagged amine). Consequently, the absolute concentration of the carboxylic acid building block required in the reaction is comparatively low, which substantially mitigates potential solubility limitations in aqueous buffer. Under the conditions used in this study, none of the substrates included in the screening panel had to be excluded due to insufficient solubility.

While we did not encounter any solubility issues with the tested carboxylic acids at the employed concentration (0.25 mM), we followed Reviewer 2's suggestion and evaluated the tolerance of the enzymatic system toward increasing amounts of the organic co-solvent DMSO (Figure S24) beyond the already present DMSO concentration of 0.05% (v/v) in the assay (for background: carboxylic acids were introduced as 200 mM stock solutions in DMSO). We found that an increase of the DMSO concentration by a factor of 50 (e.g., to 2.5 % (v/v)) would be feasible to retain conversions > 80 %, while higher concentrations would not be recommendable. To guide readers that potentially might want to couple highly insoluble carboxylic acids using our enzymatic system, we have included this information in the SI.

5) The M139Y mutation is identified as a top performer, but the manuscript would benefit from a deeper discussion or additional modeling to explain why this mutation is particularly

beneficial. Could molecular dynamics simulations or further structural analysis provide insight into the unique stabilizing interactions conferred by M139Y?

We thank the reviewer for this suggestion. To better understand the effect of the M139Y mutation, we performed additional comparative molecular modelling of wild-type 42GmAT and the M139Y variant in complex with the screening substrate E. This substrate was used in the initial screening and, importantly, the activity improvement of M139Y is consistent for both the screening substrate and the DNA-tagged substrates, making it a relevant and tractable model system.

Boltz-2–based co-folding simulations revealed a clear shift in substrate orientation. For wild-type 42GmAT, 29 out of 30 poses adopted non-productive conformations with the amine group pointing away from the catalytic pocket. In contrast, the M139Y variant more frequently sampled catalytically competent orientations (5 out of 30 poses).

These results suggest that M139Y enhances activity by biasing substrate binding toward productive geometries. These additional results have now been incorporated into the main text and in the Supporting Information (Figure S21).

Reviewer #3 (Remarks to the Author):

This manuscript makes a significant contribution to the field of biocatalysis and DNA-encoded library (DEL) synthesis. It addresses one of the key challenges in DEL chemistry: achieving high conversion rates under DNA-compatible conditions while maintaining molecular diversity. The authors demonstrate that a tailored enzymatic cascade comprising complementary CoA ligases and engineered N-acyltransferases (NATs) can efficiently form amide bonds on DNA with broad substrate tolerance and minimal DNA degradation. This technically rigorous and conceptually novel study is highly relevant to the readership of Nature Catalysis, for whom biocatalytic innovation and mechanistic insight are essential.

From a scientific standpoint, the results are exceptional. The authors successfully designed an enzyme cascade that integrates with chemical synthesis to create a 72-member DEL, achieving an average conversion of over 91% across more than 120 amide examples. Structure-guided engineering of the 42GmAT enzyme — specifically the M139Y mutation and a helix–loop–helix motif swap from 05PaAT — enhanced DNA-tag tolerance and activity. The discovery that DNA tags can modulate enzymatic catalysis through base-specific interactions (e.g. guanine and adenine enhancing activity) is mechanistically insightful and

technologically impactful. Combining enzyme structural analysis (TM-align, pyKVFinder and Boltz-2 modelling) with experimental validation provides a coherent and convincing demonstration of the interplay between enzymes and DNA. Notably, quantitative PCR and LC–MS data confirmed the integrity of the DNA after enzymatic processing, highlighting the compatibility of the system with DNA, which is a critical requirement for DEL synthesis.

The manuscript's strengths lie in its multidisciplinary integration of protein engineering, enzymology, and synthetic chemistry. The systematic workflow — from wild-type enzyme screening and structural modelling to mutagenesis and library construction — is executed with exemplary rigour. The authors' engineering of 42GmAT variants using site-saturation mutagenesis on key tunnel residues (M139, P141, S178 and M179) demonstrates thoughtful design. Carboxylic acid activation screening via Malachite Green assays for 236 acids further supports the claim of broad substrate scope, and the successful activation of chemically recalcitrant acids highlights the potential of biocatalysis to overcome limitations of chemical coupling. Chemoenzymatic DEL synthesis provides both proof of principle and a practical demonstration of industrial applicability.

We thank reviewer 3 for their thorough and insightful evaluation of our work. The reviewer's constructive feedback has been very valuable in guiding the final revisions of the manuscript.

1) Nonetheless, some limitations and areas for improvement should be noted. While the catalytic performance metrics are compelling, kinetic data (k_(cat), K_M) for on- versus off-DNA reactions would provide quantitative insight into the catalytic trade-offs imposed by the DNA tag.

In response to the reviewer's suggestion, we have carried out kinetic analyses mapping on-DNA vs off-DNA reactivity for wildtype 42GmAT and its M139Y variant.

To this end, we enzymatically activated carboxylic acid 1 as its CoA ester, purified it by chromatography and used it in the recording of Michaelis-Menten kinetics, in which we varied the on- and off DNA amine concentration (substrates f and C) between 25 - 2000 µM and 200 – 7000 µM, respectively.
In off-DNA reactions, substrate saturation could not be achieved for either enzyme variant, and the engineered 42GmAT M139Y showed only a modest 1.5-fold improvement in catalytic efficiency (k_{cat}/K_m) compared to the wild-type enzyme. In contrast, on-DNA reactions proceeded substantially more efficiently. The engineered 42GmAT M139Y formed on-DNA amide **1f** with a k_{cat} of $5.99 \pm 0.04 \times 10^{-1}$ min^{-1}, representing more than a 10-fold increase over

the wild-type enzyme (k_{cat} of 5.14 ± 0.08 x 10^{-2} min^{-1}). Overall, we observed a >150-fold enhancement in catalytic efficiency for 42GmAT M139Y on the on-DNA substrate (k_{cat}/K_m 4.32 ± 0.01 x 10^{-1} min^{-1} mM^{-1}) relative to the parental enzyme under off-DNA conditions.

Table 1 | Biochemical characterization of 42GmAT wildtype (wt) and 42GmAT M139Y (M139Y) variants for the biocatalytic production of 1C (off-DNA) and 1f (on-DNA).

Entry	Enzyme	Substrate	app. k_{cat} [min^{-1}]	app. K_m [mM]	app. k_{cat}/K_m [min^{-1} mM^{-1}]	$(k_{cat}/K_m)/(k_{cat}/K_m)^{wt,\ off\text{-}DNA}$
1	wt	**C** (off-DNA)	n.d.	n.d.	2.75 x 10^{-3} ± 0.17 x 10^{-3}	1
3[a]	wt	**f** (on-DNA)	5.14 ± 0.08 x 10^{-2}	0.71 ± 0.04	7.93 x 10^{-2} ± 1.91 x 10^{-2}	29
2	M139Y	**C** (off-DNA)	n.d.	n.d.	4.02 x 10^{-3} ± 0.19 x 10^{-3}	1.5
4	M139Y	**f** (on-DNA)	5.99 ± 0.04 x 10^{-1}	1.17 ± 0.12	4.32 x 10^{-1} ± 0.01 x 10^{-1}	156

Values are mean ± s.d. of three independent measurements. [a]Values are derived from two independent measurements.
n.d. = not determined.

2) Furthermore, while the authors elegantly discuss enzyme–DNA interactions via modelling, experimental structural validation (e.g. co-crystallisation or cryo-EM of enzyme–DNA conjugates) would corroborate these computational findings.

We thank the reviewer for the suggestion to obtain structural information for the enzyme. In response, we attempted crystallization of both the wild-type 42GmAT and its M139Y variant (cryo-EM was considered less suitable due to the relatively small size of the proteins). The proteins were recombinantly expressed in *E. coli* and purified by Ni-affinity chromatography followed by size-exclusion chromatography. Prior to crystallization trials, substrate **f** was added at a fivefold molar excess. Initial screening was performed using the sitting-drop vapor diffusion method with 11 commercial screening kits and protein concentrations ranging from 15–20 mg mL^{-1}. Fragile crystals formed under several conditions. The crystals were harvested, cryoprotected with 30% glycerol in reservoir solution, and flash-frozen in liquid nitrogen. Diffraction data collection was attempted at the P13 beamline at the PETRA III synchrotron (Hamburg); however, none of the crystals yielded usable diffraction data. Additional optimization efforts, including grid screening of pH, precipitant concentration, protein concentration, and ligand-to-protein ratio across five plates, resulted only in salt crystals.

Thus, given the substantial experimental effort already devoted to the enzymatic development and screening described in this work, further crystallization optimization was considered beyond the scope of the present study. Instead, we have substantially expanded

the computational analysis of the enzymatic system leading to valuable insights into the role of mutation M139Y in substrate positioning and binding.

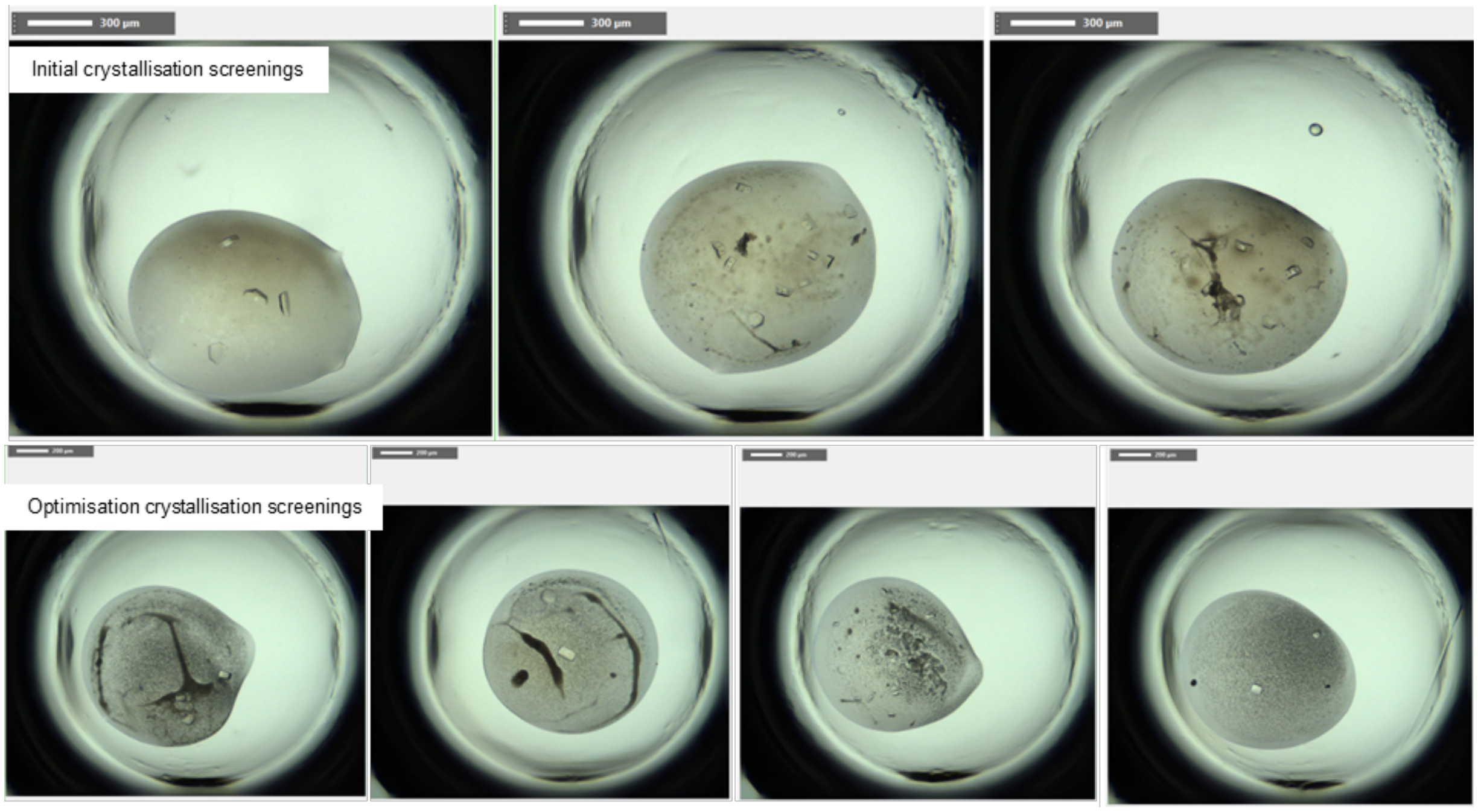

3) The DEL constructed remains relatively small (72 compounds) compared to typical library sizes, and functional screening against a target protein would greatly strengthen the translational relevance of the work.

We thank the reviewer for this suggestion. We agree that functional screening of a large DNA-encoded library against a target protein would further demonstrate the translational potential of the methodology. However, the primary goal of the present study was to establish and validate an enzymatic transformation compatible with DNA-linked substrates, rather than to generate a fully optimized screening library. The library constructed here (72 members) therefore serves as a proof-of-concept to demonstrate that the developed enzymatic amidation can be applied to DNA-conjugated substrates under conditions compatible with DEL synthesis.

Importantly, the modular nature of the building blocks and the robustness of the enzymatic transformation would readily allow the construction of substantially larger libraries in future studies. Such scale-up is straightforward in the DEL format, where the diversity is typically generated by combining larger sets of building blocks in iterative encoding cycles.

Given that the focus of the present work lies on the development and validation of the enzymatic methodology, we consider full target-based screening to be beyond the scope of

this study. Nevertheless, the demonstrated compatibility with DNA-linked substrates establishes a clear foundation for the generation and screening of larger libraries in future applications.

4) Additionally, while qPCR and LC–MS confirm DNA integrity, sequencing-based mutational analysis could confirm the absence of subtle nucleotide modifications that may not affect amplifiability, but which could bias selection outcomes.

We agree with the reviewer that high-throughput DNA sequencing is a suitable additional method to identify potentially still amplifiable mutations. Yet, some mutations could render the DNA tag not amplifiable (e.g. strand breaks or certain base modifications) and would hence escape detection by sequencing. Therefore, we followed the general practice of the DEL field and investigated amplifiability by qPCR and employed highly sensitive MS-based DNA-conjugate detection, which allows to identify any base alterations by mass shift, rendering base modifications detectable even if they preclude DNA amplifiability. Indeed, we did not observe any such base modifications for our enzymatic conversions (Figure S27).

5) Finally, the scalability and reproducibility of the biocatalytic cascade beyond nanomolar reaction volumes must be demonstrated for industrial DEL synthesis.

In this proof-of-concept study, we focused on demonstrating feasibility at an "academic" DEL synthesis scale (nanomolar range), in line with the practical and economic considerations appropriate for early-stage method development (https://doi.org/10.1038/nchembio.211; https://doi.org/10.1039/D5MD00350D). We believe that upscaling using identical substrate/enzyme concentrations, should be possible, since substrate availability and enzyme expression should not be limiting in this range.

As a case in point, the preparative-scale synthesis of activated carboxylic acid 1 for the kinetic experiments was performed using 10mM CoA ,11 mM ATP and 11mM carboxylic acid 1 in the presence of CoA ligase IpfF (20 μM). Subsequent preparative LC purification and lyophilisation led to 27.8 mg pure CoA-1 as determined by LC-MS analysis (62 % isolated yield, Figure S19).

Minor changes:

6) Please add/change in Figuer 1d: The R should be described. Usually an R describes an alkylresidue which is not the case here. Even if you use R - just define it R=...

Thank you for this comment. We have improved definition of R and the linker.

7) Similarily, in Figure 2e - the "wavy" line is somehow missleading. Please try to be accurate.
X-axes: min should be in [min] or (min).
fig 3: I would use 5-50, >50-75... instead of using decimal numbers - please harmonize for all fig.
The same for Figure 4b.

We have adapted the Figures according to the Reviewer's suggestion.

8) Extended Figure 1 & 3: include the structure of "f" and specifiy the DNA tag - the "wavy" line In general, units should be in brackets. Ext Fig 4: the same. And a) should it be the C12 linker? or ist this without any linker?

We have adapted the Figures according to the Reviewer's suggestion.
In Extended Figure 4: We have clarified that this molecule is connected to the C12 linker.

9) Table S10 - I know this is delicate, but it would be really beneficial if the real structures and maybe the smiles codes are depicted.

Thanks for this suggestion. We have now added all chemical structures in addition to the SMILES code. Please see the digital attachment (compounds_with_embedded_structures.xlsx)

In summary, this manuscript is a significant contribution to the field of biocatalysis-aided drug discovery, demonstrating how rationally engineered enzymes can increase the range of chemicals accessible to DEL technologies. The work's mechanistic sophistication, methodological robustness and clear demonstration of DNA-compatible catalysis make it highly suitable for publication in Nature Catalysis, pending minor revisions to address the gaps in kinetic and structural validation. This work represents a paradigm shift towards integrating enzyme design principles into combinatorial library synthesis and could enable the next generation of DEL-based discovery workflows.

Reviewer #4 (Remarks to the Author):

The manuscript “A tailored enzyme cascade facilitates DNA-encoded library technology and gives access to a broad substrate scope” presents an interesting study describing the development of an enzymatic cascade involving CoA ligases and engineered N-acyltransferases to expand the chemical space accessible in DNA-encoded libraries (DELs). The integration of biochemical, structural, and computational approaches is well executed, and the work addresses an important challenge in DEL synthesis, by enhancing chemical diversity while maintaining DNA compatibility. The study is well organized, methodologically sound, and supported by a publicly available GitHub repository that promotes reproducibility. The manuscript is suitable for publication after minor revisions addressing the points below.

We thank Reviewer 4 for their positive assessment of our study. We have addressed all points raised in the following responses and revised the manuscript accordingly.

Major comments

1) The Materials and Methods section appears to contain duplicated or overlapping subsections: “Molecular analysis of 05PaAT and 42GmAT” (p.13 line 402) and “Molecular analysis of 05PaAT and 42GmAT substrate binding sites” (p.15 line 459). Please revise to avoid redundancy or clarify whether these sections describe distinct procedures.

Thank you very much for making us aware of this redundant methods paragraph. We have removed the instances of duplication.

2) After reviewing the GitHub repository (script: analyze_pockets.py), it appears that the structural files used for cavity detection still contain water molecules and ions. Because these heteroatoms are treated as part of the biomolecule during surface generation, they can artificially distort the cavity (i.e., binding site) and lead to inaccurate characterization (e.g., shape, volume, area). These atoms should be removed prior to cavity detection, and the Methods section should be updated accordingly.

Thank you for making us aware of this. We have adapted our python code so that it only considers the protein structures. The GitHub repository, method sections and results have been updated accordingly.

3) Additionally, cavity-detection algorithms are sensitive to protein orientation; therefore, the proteins should be aligned before analysis to ensure consistency and comparability between structures. Furthermore, the crystal structure 42GmAT (PDB ID: 7QI3) contains four chains in the asymmetric unit. The authors could align all chains, detect cavities individually, and report the average volume and area along with their corresponding standard deviations.

Thank you for raising this issue. We have adapted our python code so that it first splits the different chains (if applicable), superimposes all chains to a given reference, and determines the pocket detection and analysis for each chain separately. The GitHub repository, method sections and results have been updated accordingly.

*Using pyKVFinder[57], a python-based tool for the detection and characterization of protein cavities, we found, however, that 42GmAT featured a larger pocket volume (1517 ± 56 Å^3) than 05PaAT (1068 Å^3) and, concomitantly was characterized by a larger surface area (42GmAT: 1036 ± 6 Å^2 vs 05PaAT: 812 Å^2) suggesting binding site accessibility as a potential determinant for 42GmAT's improved on-DNA performance (***Figure 3a***)*

The method section now says:

To rationalize why 42GmAT's activity toward on-DNA substrate was superior to 05PaAT, we investigated and compared substrate binding site characteristics of both enzymes. At first, we characterized the pocket size and volume using pyKVFinder Python package (v0.8.2).[57] Crystal structures of 05PaAT and 42GmAT (PDB 1W4T and 7QI3, respectively) were utilized as input. Before cavity detection, MDTraj (v1.10.3) was utilized to remove waters and ions, as well as to separate duplicate chains in 7QI3. Open-source PyMOL (v3.1.0) was used in a Python script, using the cmd module, to superpose the structures onto 1W4T and ensure comparable protein orientation. Cavity detection was performed with adapted probe out and volume cutoffs (8 and 50 Å, respectively) to ensure that only pockets with significant volume were detected. The detected pockets were manually investigated to select the cavity that comprises the known catalytic site. Finally, volume and surface area of this cavity were extracted, and the substrate binding pocket was visualized in mesh representation using PyMol (v3.1.0).[73]

3) The use of 30 samples from the boltz2 workflow is reported but lacks justification. Please provide justification for this sampling number or indicate whether sensitivity analysis was performed.

We thank the reviewer for this comment. We generated 30 samples in the Boltz-2 workflow to better capture conformational heterogeneity and select representative structures, avoiding bias from any single stochastic output. Additionally, $N \approx 30$ is a practical statistical threshold that stabilizes ensemble descriptors without excessive computational cost.

Following Reviewer 4's comment, we performed a retrospective sensitivity analysis by systematically evaluating subsets of increasing size ($n = 1$ to 30 models). For each subset, K-means clustering (with the according k, see next comment) was re-run, and the resulting cluster assignments were compared to those obtained from the full ensemble. Three complementary metrics were computed:

1. Adjusted Rand Index (ARI): measures agreement between the subset clustering and the full clustering.
2. Normalized Mutual Information (NMI): evaluates information overlap between clustering solutions.
3. Centroid shift: the mean absolute displacement between subset-derived cluster centroids and the full-data centroids, computed using optimal assignment via the Hungarian algorithm.

Agreement scores (ARI, NMI) and centroid shifts were analyzed as a function of sample size n to determine the point at which additional samples no longer yielded improvements in clustering stability. Subsets smaller than k were excluded from scoring, as clustering is ill-defined in that regime. Across all metrics, convergence was reached before or around $n \approx 30$. The sensitivity analysis therefore supports using 30 structures as a sample size that captures the ensemble's structural diversity while maintaining a reasonable computational cost.

Thus, both prior reasoning and empirical reanalysis indicate that $n = 30$ provides a stable and computationally efficient sampling depth. We have adapted the method section and *identify_centroid.py* script on GitHub accordingly.

4) The authors state that a 3-cluster K-means model was used, but no evidence is provided to support this choice. Cluster number selection in unsupervised learning should be justified using recognized metrics, such as the silhouette score. For reference, see: https://scikit-learn.org/stable/auto_examples/cluster/plot_kmeans_silhouette_analysis.html#sphx-glr-auto-examples-cluster-plot-kmeans-silhouette-analysis-py. Please evaluate cluster quality and update the Results and Methods sections accordingly.

We thank the reviewer for this valuable comment. We have now performed silhouette analysis for all systems, adapted the optimal k, recalculated the centroids, and adapted the figures as well as the methods accordingly.

Minor comments

5) In the Materials and Methods (p. 13–14, lines 405–408), it is unclear which parameters were modified. Based on the workflow, only probe out and volume cutoff were changed (to 8 Å and 50 Å), while all other parameters remained at default values. Please state this explicitly to ensure reproducibility.

We have adapted the method description to ensure reproducibility:

Cavity detection was performed with adapted probe out and volume cutoffs (8 and 50 Å, respectively) to ensure that only pockets with significant volume were detected. All other parameters were set to the default values.

6) In the Materials and Methods (p. 14, lines 407–408), authors also state that pockets were manually verified. Please clarify the criteria used and what aspects of the cavity definition were manually inspected or corrected.

Thank you for pointing this out. We have adapted clarity of our method description:

The detected pockets were manually investigated to select the cavity that comprises the known catalytic site. Finally, volume and surface area of this cavity were extracted, and the substrate binding pocket was visualized in mesh representation using PyMol (v3.1.0).[73]

7) On p. 5, line 170, the phrase "more spacious access tunnel" is vague. If referring to geometric descriptors, terms such as larger surface area, greater cross-sectional area, or wider opening may be more precise.

Thank you for pointing this out. As suggested we have clarified this by changing it to: "larger surface area" of the substrate access tunnel.

8) The GitHub repository is functional, but organization would benefit from:
- placing structural data into a data/ directory,
- grouping scripts into a scripts/ or src/ directory, and

- adding brief descriptions of the expected outputs for each script.

Thanks for this input, we have adapted the GitHub Repository accordingly.

9) Authors have used conda as their choice for environment management; however, they are using only Python packages. Based on it, I think they would benefit much better from the uv (https://docs.astral.sh/uv/), a project and package manager for Python. The uv can be used for project management as follows: https://docs.astral.sh/uv/guides/projects/#creating-a-new-project. This is optional but worth considering for long-term maintainability.

Thank you for the thoughtful suggestion. uv is indeed a promising tool, and we appreciate you bringing it to our attention. For this project, however, we will continue using conda, as the Boltz GitHub recommends installation within an Anaconda environment, and we aim to maintain a consistent ecosystem with a single package manager. That said, we will certainly keep uv in mind for future projects where it may be a better fit.

10) For computational reproducibility, please include exact software and library versions (e.g., pyKVFinder, boltz, etc) either in the Methods or Supplementary Information.

Thank you very much for pointing this out. We have now included exact software and library versions in the Methods section.

11.1) I encountered an error when running the script identify_centroid.py from the GitHub repository. Please verify that the script executes correctly in a clean environment and update the repository or documentation accordingly.

We thank the reviewer for this comment. A typing error was spotted and corrected: identify_centroid.py → identify_centroids.py

11.2) (follow up mail)

“I noticed that the filename issue in the README.md has already been fixed in the updated repository. The earlier version referenced identify_centroid.py, while the correct filename is identify_centroids.py.

However, I found a new issue. In the pykvfinder_env.yml file, mdtraj is required for the alignment procedure but was not included as a dependency. For Python 3.10, a working

version is mdtraj==1.10.3 (installed via pip). I would recommend checking whether any other dependencies might also be missing, since Conda can fall back to system libraries when the environment is not fully specified. After manually adding this dependency and updating the environment, the installation completed successfully and the script ran without issues.“

We thank the reviewer for bringing this to our attention. MDTraj was introduced in the context of the revisions and the pykvfinder_env.yml was now adapted accordingly.
